# Supplementary material for: Evaluating comparative effectiveness of psychosocial interventions adjunctive to opioid agonist therapy for opioid use disorder: A systematic review with network meta-analyses
Source: PLoS One. 2020 Dec 28;15(12):e0244401. doi: 10.1371/journal.pone.0244401 (PMC7769275; doi:10.1371/journal.pone.0244401)
Supplement: S2 Text — (DOCX) [file pone.0244401.s003.docx]

**S2 Text: Literature Search Strategy**

Database: Embase Classic+Embase <1947 to 2020 June 23>, Ovid MEDLINE(R) ALL <1946 to June 23, 2020>, APA PsycInfo <1806 to June Week 3 2020>, EBM Reviews - Cochrane Central Register of Controlled Trials <May 2020>

Search Strategy:

--------------------------------------------------------------------------------

1 Opiate Substitution Treatment/ (5609)

2 ((narcotic* or opioid* or opiate*) adj3 (maintenance* or substitut* or replac*)).tw,kf. (8908)

3 ((narcotic* or opioid* or opiate*) adj3 (decreas* or detoxif* or discontinu* or reduc* or taper* or wean* or withdraw*)).tw,kf. (34171)

4 ((opiate* agonist* or opioid* agonist* or opiate* receptor* or opioid* receptor*) adj3 (maintenance* or substitut* or replac* or rehabilit* or detox* or therap* or pharmacotherap* or pharmaco-therap* or treatment* or abuse-deterren*)).tw,kf. (3370)

5 ((opiate* agonist* or opioid* agonist* or opiate* receptor* or opioid* receptor*) adj3 (decreas* or detoxif* or discontinu* or reduc* or taper* or wean* or withdraw*)).tw,kf. (2344)

6 or/1-5 [OPIATE SUBSTITUTION TREATMENT] (47789)

7 Substance-Related Disorders/ (124899)

8 Substance Abuse, Intravenous/ (69073)

9 Substance Abuse, Oral/ [NEW MESH 2018] (53563)

10 exp Opioid-Related Disorders/ (48205)

11 ((narcotic* or opioid* or opiate*) adj3 (abus* or addict* or dependen* or disorder? or habituat* or misus* or mis-us* or non-medical* or nonmedical* or non-prescrib* or nonprescrib* or non-prescription* or nonprescription* or withdrawal* or (("use" or used or uses or using) adj2 (illicit* or illegal*)))).tw,kf. (62523)

12 ((drug? or substance?) adj3 (abus* or addict* or dependen* or disorder? or habituat* or misus* or mis-us* or "non-medical use?" or "nonmedical use?" or "non-prescribed use?" or "nonprescribed use?" or "non-prescription use?" or "nonprescription use?" or (("use" or used or uses or using) adj2 (illicit* or illegal*)))).tw,kf. (362235)

13 (heroin* adj3 (abus* or addict* or dependen* or disorder? or habituat* or misus* or mis-us* or non-medical* or nonmedical* or non-prescrib* or nonprescrib* or non-prescription* or nonprescription* or withdrawal* or (("use" or used or uses or using) adj2 (illicit* or illegal*)))).tw,kf. (18281)

14 ((hydrocodone or bekadid$2 or codinovo$2 or dico$2 or dicodid$2 or dihydrocodeinone$2 or hycodan$2 or hycon$2 or hydrocodeinonebitartrate$2 or hydrocodon$2 or hydrocon$2 or hydrocodonum$2 or robidone$2) adj3 (abus* or addict* or dependen* or disorder? or habituat* or misus* or mis-us* or non-medical* or nonmedical* or non-prescrib* or nonprescrib* or non-prescription* or nonprescription* or withdrawal* or (("use" or used or uses or using) adj2 (illicit* or illegal*)))).tw,kf. (192)

15 ((fentanyl or alfentanil$2 or alfenta$2 or alfentanyl$2 or beta hydroxymefentanyl or brifentanil$2 or carfentanil$2 or duragesic$2 or fanaxal$2 or fentanest$2 or fentora$2 or hypnorm$2 or limifen$2 or lofentanil$2 or mefentanyl$2 or mirfentanil$2 or ocfentanil$2 or phentanyl$2 or R-39209 or R-4263 or rapifen$2 or remifentanil$2 or sublimaze$2 or sufenta$2 or sufentanil$2 or sulfentanyl$2 or trefentanil$2) adj3 (abus* or addict* or dependen* or disorder? or habituat* or misus* or mis-us* or non-medical* or nonmedical* or non-prescrib* or nonprescrib* or non-prescription* or nonprescription* or withdrawal* or (("use" or used or uses or using) adj2 (illicit* or illegal*)))).tw,kf. (972)

16 ((morphine or anpec$2 or duramorph$2 or epimorph$2 or miro$2 or morfin$2 or morfine$2 or morphin$2 or morphinium$2 or morphium$2 or MS contin or morphia$2 or opso$2 or oramorph$2 or SDZ 202-250 or SDZ202-250 or skenan$2 or transmorphine$2 or trans-morphine$2) adj3 (abus* or addict* or dependen* or disorder? or habituat* or misus* or mis-us* or non-medical* or nonmedical* or non-prescrib* or nonprescrib* or non-prescription* or nonprescription* or withdrawal* or (("use" or used or uses or using) adj2 (illicit* or illegal*)))).tw,kf. (12896)

17 ((oxycodone or bionine$2 or bionone$2 or bolodorm$2 or broncodal$2 or bucodal$2 or cafacodal$2 or cardanon$2 or codenon$2 or codix 5 or "col 003" or col003 or DETERx$2 or dihydrohydroxycodeinone or dihydrohydroxydodeinone or dihydrone$2 or dinarkon$2 or endone$2 or eubine$2 or eucodal$2 or eucodale$2 or eucodalum$2 or eudin$2 or eukdin$2 or eukodal$2 or eumorphal$2 or eurodamine$2 or eutagen$2 or hydrocodal$2 or hydroxycodeinoma$2 or ludonal$2 or m-oxy or medicodal$2 or narcobasina$2 or narcobasine$2 or narcosin$2 or nargenol$2 or narodal$2 or nsc 19043 or nucodan$2 or opton$2 or ossicodone$2 or oxanest$2 or oxaydo$2 or oxecta$2 or oxicone$2 or oxicontin$2 or oxiconum$2 or oxikon$2 or oxy ir or oxycod$2 or oxycodeinon$2 or oxycodeinonhydrochloride or oxycodone hydrochloride or oxycodonhydrochlorid or oxycodyl$2 or oxycone$2 or oxycontin$2 or oxydose$2 or oxyfast$2 or oxygesic$2 or oxyir$2 or oxykon$2 or oxynorm$2 or pancodine$2 or pavinal$2 or percolone$2 or pronarcin$2 or remoxy$2 or roxicodone$2 or roxycodone$2 or sinthiodal$2 or stupenal$2 or supeudol$2 or tebodal$2 or tekodin$2 or thecodin$2 or theocodin$2 or xtampa$2 or xtampza$2) adj3 (abus* or addict* or dependen* or disorder? or habituat* or misus* or mis-us* or non-medical* or nonmedical* or non-prescrib* or nonprescrib* or non-prescription* or nonprescription* or withdrawal* or (("use" or used or uses or using) adj2 (illicit* or illegal*)))).tw,kf. (798)

18 or/7-17 [OPIOID USE DISORDERS] (535012)

19 Substance-Related Disorders/dt [drug therapy] (3761)

20 Substance-Related Disorders/rh [rehabilitation] (10368)

21 Substance Abuse, Intravenous/dt [drug therapy] (388)

22 Substance Abuse, Intravenous/rh [rehabilitation] (1454)

23 Substance Abuse, Oral/dt [drug therapy] (40)

24 Substance Abuse, Oral/rh [rehabilitation] (206)

25 exp Opioid-Related Disorders/dt [drug therapy] (10749)

26 exp Opioid-Related Disorders/rh [rehabilitation] (6677)

27 Analgesics, Opioid/tu [therapeutic use] (16704)

28 (OAT or OATs).tw,kf. (23617)

29 exp Buprenorphine/tu [therapeutic use] (2501)

30 Heroin/tu [therapeutic use] (410)

31 Hydromorphone/tu [therapeutic use] (319)

32 exp Methadone/tu [therapeutic use] (6983)

33 Morphine/tu [therapeutic use] (5467)

34 exp Naloxone/tu [therapeutic use] (5006)

35 ((buprenorphine or anorfin$2 or belbuca$2 or buprenex$2 or buprex$2 or buprine$2 or butrans$2 or finibron$2 or lepetan$2 or nih 8805 or nih8805 or norphin$2 or pentorel$2 or prefin$2 or probuphine$2 or rx 6029 m or rx 6029m or rx6029m or subutex$2 or temgesic$2 or transtec$2 or um 952 or um952) adj3 (maintenance* or substitut* or replac* or rehabilit* or detox* or therap* or pharmacotherap* or pharmaco-therap* or treatment* or abuse-deterren*)).tw,kf. (6672)

36 ((buprenorphine-naloxone or naloxone-buprenorphine or suboxone$2 or zubsolv$2) adj3 (maintenance* or substitut* or replac* or rehabilit* or detox* or therap* or pharmacotherap* or pharmaco-therap* or treatment* or abuse-deterren*)).tw,kf. (1116)

37 ((heroin* or diacetyl morphine or diacetylmorphine or diamorphine or diaphorin$2 or morphacetin$2) adj3 (maintenance* or substitut* or replac* or rehabilit* or detox* or therap* or pharmacotherap* or pharmaco-therap* or treatment* or abuse-deterren*)).tw,kf. (6376)

38 ((hydromorphon* or biomorphyl$2 or cofalaudid$2 or dihydromorphinon* or dihydromorphon* or diladid$2 or dilaudid$2 or dimorphon* or dolonovag$2 or exalgo$2 or hydal$2 or hydromorph contin$2 or hydromorphinone$2 or hydrostat$2 or hymorphan$2 or jurnista$2 or laudaconum$2 or novolaudon$2 or opidol$2 or paliadon retardkaps$2 or palladon$2 or rexaphon$2 or semcox$2 or sophidone$2) adj3 (maintenance* or substitut* or replac* or rehabilit* or detox* or therap* or pharmacotherap* or pharmaco-therap* or treatment* or abuse-deterren*)).tw,kf. (1095)

39 ((methadone or adanon$2 or adanon hydrochloride$2 or algidon$2 or algolysin$2 or algoxale$2 or althose$2 or althose hydrochloride$2 or amidon$2 or amidona$2 or amidone$2 or amidosan$2 or an 148 or an148 or anadon$2 or biodone$2 or butalgin$2 or deamin$2 or depridol$2 or diaminon$2 or dianone$2 or dolafin$2 or dolamid$2 or dolesone$2 or dolmed$2 or dolophine$2 or dolophine hydrochloride$2 or dorex$2 or dorexol$2 or eptadone$2 or fenadon$2 or gobbidona$2 or heptadon$2 or heptanon$2 or hoe 10820 or hoe10820 or ketalgin$2 or l polamidon$2 or mecodin$2 or mepecton$2 or mephenon$2 or metadol$2 or metadon$2 or metasedin$2 or methaddict$2 or methadose$2 or methaforte mix$2 or miadone$2 or moheptan$2 or pallidone$2 or phenadon$2 or physepton$2 or physeptone$2 or polamidon$2 or polamivet$2 or polamivit$2 or sinalgin$2 or symoron$2 or westadone$2) adj3 (maintenance* or substitut* or replac* or rehabilit* or detox* or therap* or pharmacotherap* or pharmaco-therap* or treatment* or abuse-deterren*)).tw,kf. (22594)

40 ((morphine or anpec$2 or duramorph$2 or epimorph$2 or miro$2 or morfin$2 or morfine$2 or morphin$2 or morphinium$2 or morphium$2 or MS contin or morphia$2 or opso$2 or oramorph$2 or SDZ 202-250 or SDZ202-250 or skenan$2 or transmorphine$2 or trans-morphine$2) adj3 (maintenance* or substitut* or replac* or rehabilit* or detox* or therap* or pharmacotherap* or pharmaco-therap* or treatment* or abuse-deterren*)).tw,kf. (10980)

41 ((naloxone or antioplaz$2 or en 1530 or en 15304 or en1530 or en15304 or evzio$2 or maloxone$2 or mapin$2 or MRZ 2593-Br or MRZ-2593 or nalone$2 or nalonee$2 or nalone$2 or naloxon curamed$2 or naloxon-ratiopharm$2 or naloxona$2 or narcan$2 or narcanti$2 or narcon$2 or narvcam$2 or naxone$2 or zynox$2) adj3 (maintenance* or substitut* or replac* or rehabilit* or detox* or therap* or pharmacotherap* or pharmaco-therap* or treatment* or abuse-deterren*)).tw,kf. (4039)

42 ((naltrexone or antaxon$2 or antaxone$2 or celupan$2 or en 1639a or en1639a or nalerona$2 or nalorex$2 or naltrel$2 or naltrexone hydrochloride$2 or nemexin$2 or nodict$2 or nutrexon$2 or phaltrexia$2 or re-via$2 or regental$2 or revez$2 or revia$2 or trexan$2 or vivitrex$2 or vivitrol$2) adj3 (maintenance* or substitut* or replac* or rehabilit* or detox* or therap* or pharmacotherap* or pharmaco-therap* or treatment* or abuse-deterren*)).tw,kf. (4791)

43 (((drug? adj2 dependen*) or (drug? adj2 addict*) or (narcotic* adj2 dependen*) or (narcotic* adj2 addict*) or (opioid* adj2 dependen*) or (opioid* adj2 addict*) or (opiate* adj2 dependen*) or (opiate* adj2 addict*) or (substance? adj2 dependen*) or (substance? adj2 addict*)) adj3 (treatment* or therap* or pharmacotherap* or pharmaco-therap*)).tw,kf. (17064)

44 (maintenance* adj2 (treatment* or therap* or pharmacotherap* or pharmaco-therap*)).tw,kf. (85403)

45 detoxif*.tw,kf. (96295)

46 or/19-45 [OAT] (290990)

47 18 and 46 (71181)

48 6 or 47 (103915)

49 exp Child/ not (Adolescent/ or exp Adult/) (2692233)

50 exp Infant/ not (Adolescent/ or exp Adult/) (1557993)

51 48 not (49 or 50) [CHILD-/INFANT-ONLY REMOVED] (101840)

52 exp Animals/ not (exp Animals/ and Humans/) (17708444)

53 51 not 52 [ANIMAL-ONLY REMOVED] (80234)

54 (editorial or news or newspaper article).pt. (1408450)

55 53 not 54 [OPINION PIECES REMOVED] (78912)

56 (controlled clinical trial or randomized controlled trial or pragmatic clinical trial).pt. (1180876)

57 clinical trials as topic.sh. (224959)

58 exp Randomized Controlled Trials as Topic/ (325917)

59 (randomi#ed or randomly or RCT? or placebo*).tw,kf. (3546331)

60 ((singl* or doubl* or trebl* or tripl*) adj (mask* or blind* or dumm*)).tw,kf. (716538)

61 trial.ti. (877801)

62 or/56-61 (4411328)

63 55 and 62 [RCTS] (17417)

64 controlled clinical trial.pt. (185264)

65 Controlled Clinical Trial/ or Controlled Clinical Trials as Topic/ (572792)

66 (control* adj2 trial*).tw,kf. (963140)

67 Non-Randomized Controlled Trials as Topic/ (11547)

68 (nonrandom* or non-random* or quasi-random* or quasi-experiment*).tw,kf. (159174)

69 (nRCT or nRCTs or non-RCT?).tw,kf. (2419)

70 Controlled Before-After Studies/ (218544)

71 (control* adj3 ("before and after" or "before after")).tw,kf. (739181)

72 Interrupted Time Series Analysis/ (213496)

73 time series.tw,kf. (75493)

74 (pre- adj3 post-).tw,kf. (290558)

75 (pretest adj3 posttest).tw,kf. (22426)

76 Historically Controlled Study/ (229024)

77 (control* adj2 stud$3).tw,kf. (697436)

78 Control Groups/ (125869)

79 (control* adj2 group$1).tw,kf. (1541444)

80 trial.ti. (877801)

81 or/64-80 (4580257)

82 55 and 81 [NON-RCTS] (14480)

83 63 or 82 [RCTs, NON-RCTs] (20953)

84 83 use medall [MEDLINE RECORDS] (7316)

85 drug dependence treatment/ (8490)

86 opiate substitution treatment/ (5609)

87 ((narcotic* or opioid* or opiate*) adj3 (maintenance* or substitut* or replac*)).tw,kw. (9045)

88 ((narcotic* or opioid* or opiate*) adj3 (decreas* or detoxif* or discontinu* or reduc* or taper* or wean* or withdraw*)).tw,kw. (34342)

89 ((opiate* agonist* or opioid* agonist* or opiate* receptor* or opioid* receptor*) adj3 (maintenance* or substitut* or replac* or rehabilit* or detox* or therap* or pharmacotherap* or pharmaco-therap* or treatment* or abuse-deterren*)).tw,kw. (3404)

90 ((opiate* agonist* or opioid* agonist* or opiate* receptor* or opioid* receptor*) adj3 (decreas* or detoxif* or discontinu* or reduc* or taper* or wean* or withdraw*)).tw,kw. (2352)

91 methadone treatment/ (4640)

92 or/85-91 [OPIATE SUBSTITUTION TREATMENT] (59481)

93 drug dependence/ (156749)

94 substance abuse/ (195453)

95 exp narcotic dependence/ (61179)

96 ((narcotic* or opioid* or opiate*) adj3 (abus* or addict* or dependen* or disorder? or habituat* or misus* or mis-us* or non-medical* or nonmedical* or non-prescrib* or nonprescrib* or non-prescription* or nonprescription* or withdrawal* or (("use" or used or uses or using) adj2 (illicit* or illegal*)))).tw,kw. (63846)

97 ((drug? or substance?) adj3 (abus* or addict* or dependen* or disorder? or habituat* or misus* or mis-us* or "non-medical use?" or "nonmedical use?" or "non-prescribed use?" or "nonprescribed use?" or "non-prescription use?" or "nonprescription use?" or (("use" or used or uses or using) adj2 (illicit* or illegal*)))).tw,kw. (372390)

98 (heroin* adj3 (abus* or addict* or dependen* or disorder? or habituat* or misus* or mis-us* or non-medical* or nonmedical* or non-prescrib* or nonprescrib* or non-prescription* or nonprescription* or withdrawal* or (("use" or used or uses or using) adj2 (illicit* or illegal*)))).tw,kw. (18582)

99 ((hydrocodone or bekadid$2 or codinovo$2 or dico$2 or dicodid$2 or dihydrocodeinone$2 or hycodan$2 or hycon$2 or hydrocodeinonebitartrate$2 or hydrocodon$2 or hydrocon$2 or hydrocodonum$2 or robidone$2) adj3 (abus* or addict* or dependen* or disorder? or habituat* or misus* or mis-us* or non-medical* or nonmedical* or non-prescrib* or nonprescrib* or non-prescription* or nonprescription* or withdrawal* or (("use" or used or uses or using) adj2 (illicit* or illegal*)))).tw,kw. (205)

100 ((fentanyl or alfentanil$2 or alfenta$2 or alfentanyl$2 or beta hydroxymefentanyl or brifentanil$2 or carfentanil$2 or duragesic$2 or fanaxal$2 or fentanest$2 or fentora$2 or hypnorm$2 or limifen$2 or lofentanil$2 or mefentanyl$2 or mirfentanil$2 or ocfentanil$2 or phentanyl$2 or R-39209 or R-4263 or rapifen$2 or remifentanil$2 or sublimaze$2 or sufenta$2 or sufentanil$2 or sulfentanyl$2 or trefentanil$2) adj3 (abus* or addict* or dependen* or disorder? or habituat* or misus* or mis-us* or non-medical* or nonmedical* or non-prescrib* or nonprescrib* or non-prescription* or nonprescription* or withdrawal* or (("use" or used or uses or using) adj2 (illicit* or illegal*)))).tw,kw. (999)

101 ((morphine or anpec$2 or duramorph$2 or epimorph$2 or miro$2 or morfin$2 or morfine$2 or morphin$2 or morphinium$2 or morphium$2 or MS contin or morphia$2 or opso$2 or oramorph$2 or SDZ 202-250 or SDZ202-250 or skenan$2 or transmorphine$2 or trans-morphine$2) adj3 (abus* or addict* or dependen* or disorder? or habituat* or misus* or mis-us* or non-medical* or nonmedical* or non-prescrib* or nonprescrib* or non-prescription* or nonprescription* or withdrawal* or (("use" or used or uses or using) adj2 (illicit* or illegal*)))).tw,kw. (13180)

102 ((oxycodone or bionine$2 or bionone$2 or bolodorm$2 or broncodal$2 or bucodal$2 or cafacodal$2 or cardanon$2 or codenon$2 or codix 5 or "col 003" or col003 or DETERx$2 or dihydrohydroxycodeinone or dihydrohydroxydodeinone or dihydrone$2 or dinarkon$2 or endone$2 or eubine$2 or eucodal$2 or eucodale$2 or eucodalum$2 or eudin$2 or eukdin$2 or eukodal$2 or eumorphal$2 or eurodamine$2 or eutagen$2 or hydrocodal$2 or hydroxycodeinoma$2 or ludonal$2 or m-oxy or medicodal$2 or narcobasina$2 or narcobasine$2 or narcosin$2 or nargenol$2 or narodal$2 or nsc 19043 or nucodan$2 or opton$2 or ossicodone$2 or oxanest$2 or oxaydo$2 or oxecta$2 or oxicone$2 or oxicontin$2 or oxiconum$2 or oxikon$2 or oxy ir or oxycod$2 or oxycodeinon$2 or oxycodeinonhydrochloride or oxycodone hydrochloride or oxycodonhydrochlorid or oxycodyl$2 or oxycone$2 or oxycontin$2 or oxydose$2 or oxyfast$2 or oxygesic$2 or oxyir$2 or oxykon$2 or oxynorm$2 or pancodine$2 or pavinal$2 or percolone$2 or pronarcin$2 or remoxy$2 or roxicodone$2 or roxycodone$2 or sinthiodal$2 or stupenal$2 or supeudol$2 or tebodal$2 or tekodin$2 or thecodin$2 or theocodin$2 or xtampa$2 or xtampza$2) adj3 (abus* or addict* or dependen* or disorder? or habituat* or misus* or mis-us* or non-medical* or nonmedical* or non-prescrib* or nonprescrib* or non-prescription* or nonprescription* or withdrawal* or (("use" or used or uses or using) adj2 (illicit* or illegal*)))).tw,kw. (835)

103 or/93-102 [OPIOID USE DISORDERS] (561396)

104 drug dependence/dt [Drug Therapy] (6769)

105 drug dependence/rh [Rehabilitation] (10538)

106 substance abuse/dt [Drug Therapy] (2824)

107 substance abuse/rh [Rehabilitation] (10120)

108 exp narcotic dependence/dt (13402)

109 exp narcotic dependence/rh (7427)

110 (OAT or OATs).tw,kw. (23812)

111 ((buprenorphine or anorfin$2 or belbuca$2 or buprenex$2 or buprex$2 or buprine$2 or butrans$2 or finibron$2 or lepetan$2 or nih 8805 or nih8805 or norphin$2 or pentorel$2 or prefin$2 or probuphine$2 or rx 6029 m or rx 6029m or rx6029m or subutex$2 or temgesic$2 or transtec$2 or um 952 or um952) adj3 (maintenance* or substitut* or replac* or rehabilit* or detox* or therap* or pharmacotherap* or pharmaco-therap* or treatment* or abuse-deterren*)).tw,kw. (6760)

112 ((buprenorphine-naloxone or naloxone-buprenorphine or suboxone$2 or zubsolv$2) adj3 (maintenance* or substitut* or replac* or rehabilit* or detox* or therap* or pharmacotherap* or pharmaco-therap* or treatment* or abuse-deterren*)).tw,kw. (1123)

113 ((hydromorphon* or biomorphyl$2 or cofalaudid$2 or dihydromorphinon* or dihydromorphon* or diladid$2 or dilaudid$2 or dimorphon* or dolonovag$2 or exalgo$2 or hydal$2 or hydromorph contin$2 or hydromorphinone$2 or hydrostat$2 or hymorphan$2 or jurnista$2 or laudaconum$2 or novolaudon$2 or opidol$2 or paliadon retardkaps$2 or palladon$2 or rexaphon$2 or semcox$2 or sophidone$2) adj3 (maintenance* or substitut* or replac* or rehabilit* or detox* or therap* or pharmacotherap* or pharmaco-therap* or treatment* or abuse-deterren*)).tw,kw. (1101)

114 ((heroin* or diacetyl morphine or diacetylmorphine or diamorphine or diaphorin$2 or morphacetin$2) adj3 (maintenance* or substitut* or replac* or rehabilit* or detox* or therap* or pharmacotherap* or pharmaco-therap* or treatment* or abuse-deterren*)).tw,kw. (6490)

115 ((methadone or adanon$2 or adanon hydrochloride$2 or algidon$2 or algolysin$2 or algoxale$2 or althose$2 or althose hydrochloride$2 or amidon$2 or amidona$2 or amidone$2 or amidosan$2 or an 148 or an148 or anadon$2 or biodone$2 or butalgin$2 or deamin$2 or depridol$2 or diaminon$2 or dianone$2 or dolafin$2 or dolamid$2 or dolesone$2 or dolmed$2 or dolophine$2 or dolophine hydrochloride$2 or dorex$2 or dorexol$2 or eptadone$2 or fenadon$2 or gobbidona$2 or heptadon$2 or heptanon$2 or hoe 10820 or hoe10820 or ketalgin$2 or l polamidon$2 or mecodin$2 or mepecton$2 or mephenon$2 or metadol$2 or metadon$2 or metasedin$2 or methaddict$2 or methadose$2 or methaforte mix$2 or miadone$2 or moheptan$2 or pallidone$2 or phenadon$2 or physepton$2 or physeptone$2 or polamidon$2 or polamivet$2 or polamivit$2 or sinalgin$2 or symoron$2 or westadone$2) adj3 (maintenance* or substitut* or replac* or rehabilit* or detox* or therap* or pharmacotherap* or pharmaco-therap* or treatment* or abuse-deterren*)).tw,kw. (22786)

116 ((morphine or anpec$2 or duramorph$2 or epimorph$2 or miro$2 or morfin$2 or morfine$2 or morphin$2 or morphinium$2 or morphium$2 or MS contin or morphia$2 or opso$2 or oramorph$2 or SDZ 202-250 or SDZ202-250 or skenan$2 or transmorphine$2 or trans-morphine$2) adj3 (maintenance* or substitut* or replac* or rehabilit* or detox* or therap* or pharmacotherap* or pharmaco-therap* or treatment* or abuse-deterren*)).tw,kw. (11024)

117 ((naloxone or antioplaz$2 or en 1530 or en 15304 or en1530 or en15304 or evzio$2 or maloxone$2 or mapin$2 or MRZ 2593-Br or MRZ-2593 or nalone$2 or nalonee$2 or nalone$2 or naloxon curamed$2 or naloxon-ratiopharm$2 or naloxona$2 or narcan$2 or narcanti$2 or narcon$2 or narvcam$2 or naxone$2 or zynox$2) adj3 (maintenance* or substitut* or replac* or rehabilit* or detox* or therap* or pharmacotherap* or pharmaco-therap* or treatment* or abuse-deterren*)).tw,kw. (4066)

118 ((naltrexone or antaxon$2 or antaxone$2 or celupan$2 or en 1639a or en1639a or nalerona$2 or nalorex$2 or naltrel$2 or naltrexone hydrochloride$2 or nemexin$2 or nodict$2 or nutrexon$2 or phaltrexia$2 or re-via$2 or regental$2 or revez$2 or revia$2 or trexan$2 or vivitrex$2 or vivitrol$2) adj3 (maintenance* or substitut* or replac* or rehabilit* or detox* or therap* or pharmacotherap* or pharmaco-therap* or treatment* or abuse-deterren*)).tw,kw. (4847)

119 maintenance therapy/ (41003)

120 (maintenance* adj2 (treatment* or therap* or pharmacotherap* or pharmaco-therap*)).tw,kw. (86302)

121 (((drug? adj2 dependen*) or (drug? adj2 addict*) or (narcotic* adj2 dependen*) or (narcotic* adj2 addict*) or (opioid* adj2 dependen*) or (opioid* adj2 addict*) or (opiate* adj2 dependen*) or (opiate* adj2 addict*) or (substance? adj2 dependen*) or (substance? adj2 addict*)) adj3 (treatment* or therap* or pharmacotherap* or pharmaco-therap*)).tw,kw. (17953)

122 detoxif*.tw,kw. (97370)

123 or/104-122 [OAT] (292546)

124 103 and 123 (73190)

125 92 or 124 [OPIATE SUBSTITUTION TREATMENT/OAT] (113295)

126 exp child/ not (exp adult/ or adolescent/) (2692233)

127 exp infant/ not (exp adult/ or adolescent/) (1557993)

128 fetus/ not (exp adult/ or adolescent/) (246288)

129 or/126-128 (3353458)

130 125 not 129 [CHILD-/INFANT-/FETUS-ONLY REMOVED] (111044)

131 exp animal experimentation/ or exp animal model/ or exp animal experiment/ or nonhuman/ or exp vertebrate/ (52590644)

132 exp human/ or exp human experimentation/ or exp human experiment/ (41489512)

133 131 not 132 (11102908)

134 130 not 133 [ANIMAL-ONLY REMOVED] (99949)

135 editorial.pt. (1189843)

136 134 not 135 [OPINION PIECES REMOVED] (98647)

137 randomized controlled trial/ or controlled clinical trial/ (1394366)

138 exp "clinical trial (topic)"/ (324882)

139 (randomi#ed or randomly or RCT? or placebo*).tw,kw. (3608193)

140 ((singl* or doubl* or trebl* or tripl*) adj (mask* or blind* or dumm*)).tw,kw. (743075)

141 trial.ti. (877801)

142 or/137-141 (4498154)

143 136 and 142 [RCTs] (21583)

144 controlled clinical trial/ (558111)

145 "controlled clinical trial (topic)"/ (10812)

146 (control* adj2 trial*).tw,kw. (1261078)

147 (nonrandom* or non-random* or quasi-random* or quasi-experiment*).tw,kw. (160073)

148 (nRCT or nRCTs or non-RCT?).tw,kw. (2420)

149 (control* adj3 ("before and after" or "before after")).tw,kw. (739186)

150 time series analysis/ (26180)

151 time series.tw,kw. (76464)

152 pretest posttest control group design/ (470)

153 (pre- adj3 post-).tw,kw. (290599)

154 (pretest adj3 posttest).tw,kw. (25192)

155 controlled study/ (7483962)

156 (control* adj2 stud$3).tw,kw. (1053871)

157 control group/ (125772)

158 (control* adj2 group$1).tw,kw. (1542483)

159 trial.ti. (877801)

160 or/144-159 (10810919)

161 136 and 160 [NON-RCTs] (25701)

162 143 or 161 [RCTs, NON-RCTs] (31611)

163 conference abstract.pt. (3830098)

164 limit 163 to yr="2016-current" (1559051)

165 162 and 164 (2337)

166 162 not 163 [ALL CONFERENCE ABSTRACTS REMOVED] (28323)

167 165 or 166 [CONFERENCE ABSTRACTS PRIOR TO 2016 REMOVED] (30660)

168 167 use emczd [EMBASE RECORDS] (14504)

169 Drug Rehabilitation/ (21122)

170 ((narcotic* or opioid* or opiate*) adj3 (maintenance* or substitut* or replac*)).tw. (8781)

171 ((narcotic* or opioid* or opiate*) adj3 (decreas* or detoxif* or discontinu* or reduc* or taper* or wean* or withdraw*)).tw. (34125)

172 ((opiate* agonist* or opioid* agonist* or opiate* receptor* or opioid* receptor*) adj3 (maintenance* or substitut* or replac* or rehabilit* or detox* or therap* or pharmacotherap* or pharmaco-therap* or treatment* or abuse-deterren*)).tw. (3326)

173 ((opiate* agonist* or opioid* agonist* or opiate* receptor* or opioid* receptor*) adj3 (decreas* or detoxif* or discontinu* or reduc* or taper* or wean* or withdraw*)).tw. (2344)

174 methadone maintenance/ (8262)

175 or/169-174 [OPIATE SUBSTITUTION TREATMENT] (70855)

176 exp drug dependency/ (264338)

177 drug abuse/ (198912)

178 "substance use disorder"/ (151356)

179 Intravenous Drug Usage/ (3955)

180 ((narcotic* or opioid* or opiate*) adj3 (abus* or addict* or dependen* or disorder? or habituat* or misus* or mis-us* or non-medical* or nonmedical* or non-prescrib* or nonprescrib* or non-prescription* or nonprescription* or withdrawal* or (("use" or used or uses or using) adj2 (illicit* or illegal*)))).tw. (62051)

181 ((drug? or substance?) adj3 (abus* or addict* or dependen* or disorder? or habituat* or misus* or mis-us* or "non-medical use?" or "nonmedical use?" or "non-prescribed use?" or "nonprescribed use?" or "non-prescription use?" or "nonprescription use?" or (("use" or used or uses or using) adj2 (illicit* or illegal*)))).tw. (358695)

182 (heroin* adj3 (abus* or addict* or dependen* or disorder? or habituat* or misus* or mis-us* or non-medical* or nonmedical* or non-prescrib* or nonprescrib* or non-prescription* or nonprescription* or withdrawal* or (("use" or used or uses or using) adj2 (illicit* or illegal*)))).tw. (18240)

183 ((hydrocodone or bekadid$2 or codinovo$2 or dico$2 or dicodid$2 or dihydrocodeinone$2 or hycodan$2 or hycon$2 or hydrocodeinonebitartrate$2 or hydrocodon$2 or hydrocon$2 or hydrocodonum$2 or robidone$2) adj3 (abus* or addict* or dependen* or disorder? or habituat* or misus* or mis-us* or non-medical* or nonmedical* or non-prescrib* or nonprescrib* or non-prescription* or nonprescription* or withdrawal* or (("use" or used or uses or using) adj2 (illicit* or illegal*)))).tw. (192)

184 ((fentanyl or alfentanil$2 or alfenta$2 or alfentanyl$2 or beta hydroxymefentanyl or brifentanil$2 or carfentanil$2 or duragesic$2 or fanaxal$2 or fentanest$2 or fentora$2 or hypnorm$2 or limifen$2 or lofentanil$2 or mefentanyl$2 or mirfentanil$2 or ocfentanil$2 or phentanyl$2 or R-39209 or R-4263 or rapifen$2 or remifentanil$2 or sublimaze$2 or sufenta$2 or sufentanil$2 or sulfentanyl$2 or trefentanil$2) adj3 (abus* or addict* or dependen* or disorder? or habituat* or misus* or mis-us* or non-medical* or nonmedical* or non-prescrib* or nonprescrib* or non-prescription* or nonprescription* or withdrawal* or (("use" or used or uses or using) adj2 (illicit* or illegal*)))).tw. (971)

185 ((morphine or anpec$2 or duramorph$2 or epimorph$2 or miro$2 or morfin$2 or morfine$2 or morphin$2 or morphinium$2 or morphium$2 or MS contin or morphia$2 or opso$2 or oramorph$2 or SDZ 202-250 or SDZ202-250 or skenan$2 or transmorphine$2 or trans-morphine$2) adj3 (abus* or addict* or dependen* or disorder? or habituat* or misus* or mis-us* or non-medical* or nonmedical* or non-prescrib* or nonprescrib* or non-prescription* or nonprescription* or withdrawal* or (("use" or used or uses or using) adj2 (illicit* or illegal*)))).tw. (12799)

186 ((oxycodone or bionine$2 or bionone$2 or bolodorm$2 or broncodal$2 or bucodal$2 or cafacodal$2 or cardanon$2 or codenon$2 or codix 5 or "col 003" or col003 or DETERx$2 or dihydrohydroxycodeinone or dihydrohydroxydodeinone or dihydrone$2 or dinarkon$2 or endone$2 or eubine$2 or eucodal$2 or eucodale$2 or eucodalum$2 or eudin$2 or eukdin$2 or eukodal$2 or eumorphal$2 or eurodamine$2 or eutagen$2 or hydrocodal$2 or hydroxycodeinoma$2 or ludonal$2 or m-oxy or medicodal$2 or narcobasina$2 or narcobasine$2 or narcosin$2 or nargenol$2 or narodal$2 or nsc 19043 or nucodan$2 or opton$2 or ossicodone$2 or oxanest$2 or oxaydo$2 or oxecta$2 or oxicone$2 or oxicontin$2 or oxiconum$2 or oxikon$2 or oxy ir or oxycod$2 or oxycodeinon$2 or oxycodeinonhydrochloride or oxycodone hydrochloride or oxycodonhydrochlorid or oxycodyl$2 or oxycone$2 or oxycontin$2 or oxydose$2 or oxyfast$2 or oxygesic$2 or oxyir$2 or oxykon$2 or oxynorm$2 or pancodine$2 or pavinal$2 or percolone$2 or pronarcin$2 or remoxy$2 or roxicodone$2 or roxycodone$2 or sinthiodal$2 or stupenal$2 or supeudol$2 or tebodal$2 or tekodin$2 or thecodin$2 or theocodin$2 or xtampa$2 or xtampza$2) adj3 (abus* or addict* or dependen* or disorder? or habituat* or misus* or mis-us* or non-medical* or nonmedical* or non-prescrib* or nonprescrib* or non-prescription* or nonprescription* or withdrawal* or (("use" or used or uses or using) adj2 (illicit* or illegal*)))).tw. (797)

187 or/176-186 [OPIOID USE DISORDERS] (697764)

188 ((buprenorphine or anorfin$2 or belbuca$2 or buprenex$2 or buprex$2 or buprine$2 or butrans$2 or finibron$2 or lepetan$2 or nih 8805 or nih8805 or norphin$2 or pentorel$2 or prefin$2 or probuphine$2 or rx 6029 m or rx 6029m or rx6029m or subutex$2 or temgesic$2 or transtec$2 or um 952 or um952) adj3 (maintenance* or substitut* or replac* or rehabilit* or detox* or therap* or pharmacotherap* or pharmaco-therap* or treatment* or abuse-deterren*)).tw. (6669)

189 ((buprenorphine-naloxone or naloxone-buprenorphine or suboxone$2 or zubsolv$2) adj3 (maintenance* or substitut* or replac* or rehabilit* or detox* or therap* or pharmacotherap* or pharmaco-therap* or treatment* or abuse-deterren*)).tw. (1115)

190 ((hydromorphon* or biomorphyl$2 or cofalaudid$2 or dihydromorphinon* or dihydromorphon* or diladid$2 or dilaudid$2 or dimorphon* or dolonovag$2 or exalgo$2 or hydal$2 or hydromorph contin$2 or hydromorphinone$2 or hydrostat$2 or hymorphan$2 or jurnista$2 or laudaconum$2 or novolaudon$2 or opidol$2 or paliadon retardkaps$2 or palladon$2 or rexaphon$2 or semcox$2 or sophidone$2) adj3 (maintenance* or substitut* or replac* or rehabilit* or detox* or therap* or pharmacotherap* or pharmaco-therap* or treatment* or abuse-deterren*)).tw. (1088)

191 ((heroin* or diacetyl morphine or diacetylmorphine or diamorphine or diaphorin$2 or heroine$2 or morphacetin$2) adj3 (maintenance* or substitut* or replac* or rehabilit* or detox* or therap* or pharmacotherap* or pharmaco-therap* or treatment* or abuse-deterren*)).tw. (6367)

192 ((methadone or adanon$2 or adanon hydrochloride$2 or algidon$2 or algolysin$2 or algoxale$2 or althose$2 or althose hydrochloride$2 or amidon$2 or amidona$2 or amidone$2 or amidosan$2 or an 148 or an148 or anadon$2 or biodone$2 or butalgin$2 or deamin$2 or depridol$2 or diaminon$2 or dianone$2 or dolafin$2 or dolamid$2 or dolesone$2 or dolmed$2 or dolophine$2 or dolophine hydrochloride$2 or dorex$2 or dorexol$2 or eptadone$2 or fenadon$2 or gobbidona$2 or heptadon$2 or heptanon$2 or hoe 10820 or hoe10820 or ketalgin$2 or l polamidon$2 or mecodin$2 or mepecton$2 or mephenon$2 or metadol$2 or metadon$2 or metasedin$2 or methaddict$2 or methadose$2 or methaforte mix$2 or miadone$2 or moheptan$2 or pallidone$2 or phenadon$2 or physepton$2 or physeptone$2 or polamidon$2 or polamivet$2 or polamivit$2 or sinalgin$2 or symoron$2 or westadone$2) adj3 (maintenance* or substitut* or replac* or rehabilit* or detox* or therap* or pharmacotherap* or pharmaco-therap* or treatment* or abuse-deterren*)).tw. (22552)

193 ((morphine or anpec$2 or duramorph$2 or epimorph$2 or miro$2 or morfin$2 or morfine$2 or morphin$2 or morphinium$2 or morphium$2 or MS contin or morphia$2 or opso$2 or oramorph$2 or SDZ 202-250 or SDZ202-250 or skenan$2 or transmorphine$2 or trans-morphine$2) adj3 (maintenance* or substitut* or replac* or rehabilit* or detox* or therap* or pharmacotherap* or pharmaco-therap* or treatment* or abuse-deterren*)).tw. (10935)

194 ((naloxone or antioplaz$2 or en 1530 or en 15304 or en1530 or en15304 or evzio$2 or maloxone$2 or mapin$2 or MRZ 2593-Br or MRZ-2593 or nalone$2 or nalonee$2 or nalone$2 or naloxon curamed$2 or naloxon-ratiopharm$2 or naloxona$2 or narcan$2 or narcanti$2 or narcon$2 or narvcam$2 or naxone$2 or zynox$2) adj3 (maintenance* or substitut* or replac* or rehabilit* or detox* or therap* or pharmacotherap* or pharmaco-therap* or treatment* or abuse-deterren*)).tw. (4039)

195 ((naltrexone or antaxon$2 or antaxone$2 or celupan$2 or en 1639a or en1639a or nalerona$2 or nalorex$2 or naltrel$2 or naltrexone hydrochloride$2 or nemexin$2 or nodict$2 or nutrexon$2 or phaltrexia$2 or re-via$2 or regental$2 or revez$2 or revia$2 or trexan$2 or vivitrex$2 or vivitrol$2) adj3 (maintenance* or substitut* or replac* or rehabilit* or detox* or therap* or pharmacotherap* or pharmaco-therap* or treatment* or abuse-deterren*)).tw. (4790)

196 maintenance therapy/ (41003)

197 (maintenance adj2 (treatment* or therap* or pharmacotherap* or pharmaco-therap*)).tw. (85211)

198 (OAT or OATs).tw. (23539)

199 (((drug? adj2 dependen*) or (drug? adj2 addict*) or (narcotic* adj2 dependen*) or (narcotic* adj2 addict*) or (opioid* adj2 dependen*) or (opioid* adj2 addict*) or (opiate* adj2 dependen*) or (opiate* adj2 addict*) or (substance? adj2 dependen*) or (substance? adj2 addict*)) adj3 (treatment* or therap* or pharmacotherap* or pharmaco-therap*)).tw. (16998)

200 Detoxification/ (28041)

201 detoxif*.tw. (95857)

202 or/196-201 [OAT] (242367)

203 187 and 202 (37861)

204 175 or 203 (95457)

205 exp Animals/ not (exp Animals/ and Humans/) (17708444)

206 204 not 205 [ANIMAL-ONLY REMOVED] (76426)

207 limit 206 to dissertation [Limit not valid in Embase,Ovid MEDLINE(R),Ovid MEDLINE(R) Daily Update,Ovid MEDLINE(R) In-Process,Ovid MEDLINE(R) Publisher,CCTR; records were retained] (48755)

208 206 not 207 [DISSERTATIONS REMOVED] (27671)

209 clinical trials/ (105309)

210 (randomi#ed or randomly or RCT$1 or placebo*).tw. (3544036)

211 ((singl* or doubl* or trebl* or tripl*) adj (mask* or blind* or dumm*)).tw. (716427)

212 trial.ti. (877801)

213 or/209-212 (3898160)

214 208 and 213 [RCTS] (3147)

215 (control* adj2 trial*).tw. (960095)

216 (nonrandom* or non-random* or quasi-random* or quasi-experiment*).tw. (158985)

217 (nRCT or nRCTs or non-RCT?).tw. (2419)

218 (control* adj3 ("before and after" or "before after")).tw. (739172)

219 time series/ (26676)

220 time series.tw. (74898)

221 (pretest adj3 posttest).tw. (22420)

222 (control* adj2 stud$3).tw. (696298)

223 Experiment Controls/ (918)

224 (control* adj2 group$1).tw. (1541076)

225 trial.ti. (877801)

226 or/215-225 (3701205)

227 208 and 226 [NON-RCTS] (2248)

228 214 or 227 [RCTs, NON-RCTs] (3860)

229 228 use medall,emczd (0)

230 228 not 229 [PSYCINFO RECORDS] (3860)

231 Opiate Substitution Treatment/ (5609)

232 ((narcotic* or opioid* or opiate*) adj3 (maintenance* or substitut* or replac*)).ti,ab,kw. (8871)

233 ((narcotic* or opioid* or opiate*) adj3 (decreas* or detoxif* or discontinu* or reduc* or taper* or wean* or withdraw*)).ti,ab,kw. (34251)

234 ((opiate* agonist* or opioid* agonist* or opiate* receptor* or opioid* receptor*) adj3 (maintenance* or substitut* or replac* or rehabilit* or detox* or therap* or pharmacotherap* or pharmaco-therap* or treatment* or abuse-deterren*)).ti,ab,kw. (3362)

235 ((opiate* agonist* or opioid* agonist* or opiate* receptor* or opioid* receptor*) adj3 (decreas* or detoxif* or discontinu* or reduc* or taper* or wean* or withdraw*)).ti,ab,kw. (2347)

236 or/231-235 [OPIATE SUBSTITUTION TREATMENT] (47808)

237 Substance-Related Disorders/ (124899)

238 Substance Abuse, Intravenous/ (69073)

239 Substance Abuse, Oral/ [NEW MESH 2018] (53563)

240 exp Opioid-Related Disorders/ (48205)

241 ((narcotic* or opioid* or opiate*) adj3 (abus* or addict* or dependen* or disorder? or habituat* or misus* or mis-us* or non-medical* or nonmedical* or non-prescrib* or nonprescrib* or non-prescription* or nonprescription* or withdrawal* or (("use" or used or uses or using) adj2 (illicit* or illegal*)))).ti,ab,kw. (63254)

242 ((drug? or substance?) adj3 (abus* or addict* or dependen* or disorder? or habituat* or misus* or mis-us* or "non-medical use?" or "nonmedical use?" or "non-prescribed use?" or "nonprescribed use?" or "non-prescription use?" or "nonprescription use?" or (("use" or used or uses or using) adj2 (illicit* or illegal*)))).ti,ab,kw. (363956)

243 (heroin* adj3 (abus* or addict* or dependen* or disorder? or habituat* or misus* or mis-us* or non-medical* or nonmedical* or non-prescrib* or nonprescrib* or non-prescription* or nonprescription* or withdrawal* or (("use" or used or uses or using) adj2 (illicit* or illegal*)))).ti,ab,kw. (18266)

244 ((hydrocodone or bekadid$2 or codinovo$2 or dico$2 or dicodid$2 or dihydrocodeinone$2 or hycodan$2 or hycon$2 or hydrocodeinonebitartrate$2 or hydrocodon$2 or hydrocon$2 or hydrocodonum$2 or robidone$2) adj3 (abus* or addict* or dependen* or disorder? or habituat* or misus* or mis-us* or non-medical* or nonmedical* or non-prescrib* or nonprescrib* or non-prescription* or nonprescription* or withdrawal* or (("use" or used or uses or using) adj2 (illicit* or illegal*)))).ti,ab,kw. (205)

245 ((fentanyl or alfentanil$2 or alfenta$2 or alfentanyl$2 or beta hydroxymefentanyl or brifentanil$2 or carfentanil$2 or duragesic$2 or fanaxal$2 or fentanest$2 or fentora$2 or hypnorm$2 or limifen$2 or lofentanil$2 or mefentanyl$2 or mirfentanil$2 or ocfentanil$2 or phentanyl$2 or R-39209 or R-4263 or rapifen$2 or remifentanil$2 or sublimaze$2 or sufenta$2 or sufentanil$2 or sulfentanyl$2 or trefentanil$2) adj3 (abus* or addict* or dependen* or disorder? or habituat* or misus* or mis-us* or non-medical* or nonmedical* or non-prescrib* or nonprescrib* or non-prescription* or nonprescription* or withdrawal* or (("use" or used or uses or using) adj2 (illicit* or illegal*)))).ti,ab,kw. (992)

246 ((morphine or anpec$2 or duramorph$2 or epimorph$2 or miro$2 or morfin$2 or morfine$2 or morphin$2 or morphinium$2 or morphium$2 or MS contin or morphia$2 or opso$2 or oramorph$2 or SDZ 202-250 or SDZ202-250 or skenan$2 or transmorphine$2 or trans-morphine$2) adj3 (abus* or addict* or dependen* or disorder? or habituat* or misus* or mis-us* or non-medical* or nonmedical* or non-prescrib* or nonprescrib* or non-prescription* or nonprescription* or withdrawal* or (("use" or used or uses or using) adj2 (illicit* or illegal*)))).ti,ab,kw. (13094)

247 ((oxycodone or bionine$2 or bionone$2 or bolodorm$2 or broncodal$2 or bucodal$2 or cafacodal$2 or cardanon$2 or codenon$2 or codix 5 or "col 003" or col003 or DETERx$2 or dihydrohydroxycodeinone or dihydrohydroxydodeinone or dihydrone$2 or dinarkon$2 or endone$2 or eubine$2 or eucodal$2 or eucodale$2 or eucodalum$2 or eudin$2 or eukdin$2 or eukodal$2 or eumorphal$2 or eurodamine$2 or eutagen$2 or hydrocodal$2 or hydroxycodeinoma$2 or ludonal$2 or m-oxy or medicodal$2 or narcobasina$2 or narcobasine$2 or narcosin$2 or nargenol$2 or narodal$2 or nsc 19043 or nucodan$2 or opton$2 or ossicodone$2 or oxanest$2 or oxaydo$2 or oxecta$2 or oxicone$2 or oxicontin$2 or oxiconum$2 or oxikon$2 or oxy ir or oxycod$2 or oxycodeinon$2 or oxycodeinonhydrochloride or oxycodone hydrochloride or oxycodonhydrochlorid or oxycodyl$2 or oxycone$2 or oxycontin$2 or oxydose$2 or oxyfast$2 or oxygesic$2 or oxyir$2 or oxykon$2 or oxynorm$2 or pancodine$2 or pavinal$2 or percolone$2 or pronarcin$2 or remoxy$2 or roxicodone$2 or roxycodone$2 or sinthiodal$2 or stupenal$2 or supeudol$2 or tebodal$2 or tekodin$2 or thecodin$2 or theocodin$2 or xtampa$2 or xtampza$2) adj3 (abus* or addict* or dependen* or disorder? or habituat* or misus* or mis-us* or non-medical* or nonmedical* or non-prescrib* or nonprescrib* or non-prescription* or nonprescription* or withdrawal* or (("use" or used or uses or using) adj2 (illicit* or illegal*)))).ti,ab,kw. (834)

248 or/237-247 [OPIOID USE DISORDERS] (537073)

249 Substance-Related Disorders/dt [drug therapy] (3761)

250 Substance-Related Disorders/rh [rehabilitation] (10368)

251 Substance Abuse, Intravenous/dt [drug therapy] (388)

252 Substance Abuse, Intravenous/rh [rehabilitation] (1454)

253 Substance Abuse, Oral/dt [drug therapy] (40)

254 Substance Abuse, Oral/rh [rehabilitation] (206)

255 exp Opioid-Related Disorders/dt [drug therapy] (10749)

256 exp Opioid-Related Disorders/rh [rehabilitation] (6677)

257 Analgesics, Opioid/tu [therapeutic use] (16704)

258 (OAT or OATs).ti,ab,kw. (23803)

259 exp Buprenorphine/tu [therapeutic use] (2501)

260 Heroin/tu [therapeutic use] (410)

261 Hydromorphone/tu [therapeutic use] (319)

262 exp Methadone/tu [therapeutic use] (6983)

263 Morphine/tu [therapeutic use] (5467)

264 exp Naloxone/tu [therapeutic use] (5006)

265 ((buprenorphine or anorfin$2 or belbuca$2 or buprenex$2 or buprex$2 or buprine$2 or butrans$2 or finibron$2 or lepetan$2 or nih 8805 or nih8805 or norphin$2 or pentorel$2 or prefin$2 or probuphine$2 or rx 6029 m or rx 6029m or rx6029m or subutex$2 or temgesic$2 or transtec$2 or um 952 or um952) adj3 (maintenance* or substitut* or replac* or rehabilit* or detox* or therap* or pharmacotherap* or pharmaco-therap* or treatment* or abuse-deterren*)).ti,ab,kw. (6697)

266 ((buprenorphine-naloxone or naloxone-buprenorphine or suboxone$2 or zubsolv$2) adj3 (maintenance* or substitut* or replac* or rehabilit* or detox* or therap* or pharmacotherap* or pharmaco-therap* or treatment* or abuse-deterren*)).ti,ab,kw. (1118)

267 ((heroin* or diacetyl morphine or diacetylmorphine or diamorphine or diaphorin$2 or morphacetin$2) adj3 (maintenance* or substitut* or replac* or rehabilit* or detox* or therap* or pharmacotherap* or pharmaco-therap* or treatment* or abuse-deterren*)).ti,ab,kw. (6303)

268 ((hydromorphon* or biomorphyl$2 or cofalaudid$2 or dihydromorphinon* or dihydromorphon* or diladid$2 or dilaudid$2 or dimorphon* or dolonovag$2 or exalgo$2 or hydal$2 or hydromorph contin$2 or hydromorphinone$2 or hydrostat$2 or hymorphan$2 or jurnista$2 or laudaconum$2 or novolaudon$2 or opidol$2 or paliadon retardkaps$2 or palladon$2 or rexaphon$2 or semcox$2 or sophidone$2) adj3 (maintenance* or substitut* or replac* or rehabilit* or detox* or therap* or pharmacotherap* or pharmaco-therap* or treatment* or abuse-deterren*)).ti,ab,kw. (1100)

269 ((methadone or adanon$2 or adanon hydrochloride$2 or algidon$2 or algolysin$2 or algoxale$2 or althose$2 or althose hydrochloride$2 or amidon$2 or amidona$2 or amidone$2 or amidosan$2 or an 148 or an148 or anadon$2 or biodone$2 or butalgin$2 or deamin$2 or depridol$2 or diaminon$2 or dianone$2 or dolafin$2 or dolamid$2 or dolesone$2 or dolmed$2 or dolophine$2 or dolophine hydrochloride$2 or dorex$2 or dorexol$2 or eptadone$2 or fenadon$2 or gobbidona$2 or heptadon$2 or heptanon$2 or hoe 10820 or hoe10820 or ketalgin$2 or l polamidon$2 or mecodin$2 or mepecton$2 or mephenon$2 or metadol$2 or metadon$2 or metasedin$2 or methaddict$2 or methadose$2 or methaforte mix$2 or miadone$2 or moheptan$2 or pallidone$2 or phenadon$2 or physepton$2 or physeptone$2 or polamidon$2 or polamivet$2 or polamivit$2 or sinalgin$2 or symoron$2 or westadone$2) adj3 (maintenance* or substitut* or replac* or rehabilit* or detox* or therap* or pharmacotherap* or pharmaco-therap* or treatment* or abuse-deterren*)).ti,ab,kw. (22444)

270 ((morphine or anpec$2 or duramorph$2 or epimorph$2 or miro$2 or morfin$2 or morfine$2 or morphin$2 or morphinium$2 or morphium$2 or MS contin or morphia$2 or opso$2 or oramorph$2 or SDZ 202-250 or SDZ202-250 or skenan$2 or transmorphine$2 or trans-morphine$2) adj3 (maintenance* or substitut* or replac* or rehabilit* or detox* or therap* or pharmacotherap* or pharmaco-therap* or treatment* or abuse-deterren*)).ti,ab,kw. (10983)

271 ((naloxone or antioplaz$2 or en 1530 or en 15304 or en1530 or en15304 or evzio$2 or maloxone$2 or mapin$2 or MRZ 2593-Br or MRZ-2593 or nalone$2 or nalonee$2 or nalone$2 or naloxon curamed$2 or naloxon-ratiopharm$2 or naloxona$2 or narcan$2 or narcanti$2 or narcon$2 or narvcam$2 or naxone$2 or zynox$2) adj3 (maintenance* or substitut* or replac* or rehabilit* or detox* or therap* or pharmacotherap* or pharmaco-therap* or treatment* or abuse-deterren*)).ti,ab,kw. (4037)

272 ((naltrexone or antaxon$2 or antaxone$2 or celupan$2 or en 1639a or en1639a or nalerona$2 or nalorex$2 or naltrel$2 or naltrexone hydrochloride$2 or nemexin$2 or nodict$2 or nutrexon$2 or phaltrexia$2 or re-via$2 or regental$2 or revez$2 or revia$2 or trexan$2 or vivitrex$2 or vivitrol$2) adj3 (maintenance* or substitut* or replac* or rehabilit* or detox* or therap* or pharmacotherap* or pharmaco-therap* or treatment* or abuse-deterren*)).ti,ab,kw. (4808)

273 (((drug? adj2 dependen*) or (drug? adj2 addict*) or (narcotic* adj2 dependen*) or (narcotic* adj2 addict*) or (opioid* adj2 dependen*) or (opioid* adj2 addict*) or (opiate* adj2 dependen*) or (opiate* adj2 addict*) or (substance? adj2 dependen*) or (substance? adj2 addict*)) adj3 (treatment* or therap* or pharmacotherap* or pharmaco-therap*)).ti,ab,kw. (17393)

274 (maintenance* adj2 (treatment* or therap* or pharmacotherap* or pharmaco-therap*)).ti,ab,kw. (85926)

275 detoxif*.ti,ab,kw. (97215)

276 or/249-275 [OAT] (292670)

277 248 and 276 (71286)

278 236 or 277 (104041)

279 exp Child/ not (Adolescent/ or exp Adult/) (2692233)

280 exp Infant/ not (Adolescent/ or exp Adult/) (1557993)

281 278 not (279 or 280) [CHILD-/INFANT-ONLY REMOVED] (101948)

282 281 use cctr [CENTRAL RECORDS] (8730)

283 84 or 168 or 230 or 282 [ALL DATABASES] (34410)

284 283 use medall [MEDLINE RECORDS] (7316)

285 (201901* or 201902* or 201903* or 201904* or 201905* or 201906* or 201907* or 201908* or "20190901").dt. (893626)

286 284 and 285 [MEDLINE RECORDS - UPDATE PERIOD] (348)

287 283 use emczd [EMBASE RECORDS] (14504)

288 (201901* or 201902* or 201903* or 201904* or 201905* or 201906* or 201907* or 201908* or "20190901").dc. (1241097)

289 287 and 288 [EMBASE RECORDS - UPDATE PERIOD] (1180)

290 283 use cctr [CENTRAL RECORDS] (8730)

291 (201901* or 201902* or 201903* or 201904* or 201905* or 201906* or 201907* or 201908* or "20190901").up. (745456)

292 290 and 291 [CENTRAL RECORDS - UPDATE PERIOD] (3481)

293 283 not (284 or 287 or 290) [PSYCINFO RECORDS] (3860)

294 291 and 293 [PSYCINFO RECORDS - UPDATE PERIOD] (114)

295 286 or 289 or 292 or 294 [ALL DATABASES - UPDATE PERIOD] (5123)

296 remove duplicates from 295 (4755) [TOTAL UNIQUE RECORDS]

297 296 use medall [MEDLINE UNIQUE RECORDS] (335)

298 296 use emczd [EMBASE UNIQUE RECORDS] (990)

299 296 use cctr [CENTRAL UNIQUE RECORDS] (3350)

300 296 not (297 or 298 or 299) [PSYCINFO UNIQUE RECORDS] (80)
