# Supplementary material for: Evaluating comparative effectiveness of psychosocial interventions adjunctive to opioid agonist therapy for opioid use disorder: A systematic review with network meta-analyses
Source: PLoS One. 2020 Dec 28;15(12):e0244401. doi: 10.1371/journal.pone.0244401 (PMC7769275; doi:10.1371/journal.pone.0244401)
Supplement: S3 Text — (DOCX) [file pone.0244401.s004.docx]

**S3 Text: A Priori List of Eligible Interventions**

Provided below is a list that was developed a priori list of psychosocial interventions for which we anticipated finding study data and which were considered of clinical relevance for the planned knowledge syntheses.

1. ***Standard Care***
2. ***Medical Management***
3. ***Acceptance and Commitment Therapy***
4. ***Cognitive Behavioral Therapy***
5. ***Contingency Management (i.e., medication, prize, or monetary equivalent reinforcement)***
6. ***Community Reinforcement***
7. ***Dialectical Behavior Therapy***
8. ***Drug Counselling, General Counselling, and Supportive Counselling***
9. ***Motivational Interviewing***
10. ***Behavioural Couples Therapy***
11. ***Cognitive-Behavioural Couples Therapy***
12. ***Emotion- Focused Couples Therapy***
13. ***Family Therapy***
14. ***Behavioural Family Therapy***
15. ***Cognitive-Behavioural Family Therapy***
16. ***Emotion- Focused Family Therapy***
17. ***Multi-Dimensional Family Therapy***
18. ***12-Step Facilitation Group***
19. ***Acceptance and Commitment Therapy***
20. ***Cognitive Behavioural Group Therapy***
21. ***Dialectical Behavior Group Therapy***
22. ***Mindfulness Group Therapy***
23. ***Group Motivational Interviewing***
