# Supplementary material for: Evaluating comparative effectiveness of psychosocial interventions adjunctive to opioid agonist therapy for opioid use disorder: A systematic review with network meta-analyses
Source: PLoS One. 2020 Dec 28;15(12):e0244401. doi: 10.1371/journal.pone.0244401 (PMC7769275; doi:10.1371/journal.pone.0244401)
Supplement: S4 Text — (DOCX) [file pone.0244401.s005.docx]

**S4 Text: Additional Details, Statistical Methods for NMA**

**Primary Analyses**

We planned a priori to conduct separate NMAs of available direct and indirect evidence using a Bayesian framework for outcomes with sufficient data for analysis in cases where well-connected evidence networks existed, and the transitivity assumption was judged appropriate. Exploration of potential clinical and methodological heterogeneity between studies as well as variability in outcome reporting formats was performed using tabular and graphical approaches to evaluate differences in participant demographics, methods, and outcome formats between studies. Intervention characteristics including the content of psychosocial interventions, comparators, frequency, and intensity of interventions were also compared prior to conducting analyses. Substantial methodologic and clinical heterogeneity were apparent between studies, specifically for outcome reporting formats, whereby outcomes were reported using a variety of approaches (e.g., urinalysis, self-report, oral swab). Due to the high heterogeneity between studies, only treatment retention reported as the number of individuals per arm retained in treatment at the latest study timepoint was amenable to synthesis using NMA. A random effects NMA model was used for this outcome assuming a common heterogeneity parameter across all comparisons, accounting for correlations in multi-arm studies and using vague (non-informative) prior distributions for all treatment effects.^1^ Results are reported as posterior median odds ratios (ORs) with corresponding 95% credible intervals (CrIs), which are the Bayesian analogue of 95% confidence intervals. Surface under the cumulative ranking area (SUCRA) values and median treatment rankings are also presented for all treatments in the network. NMA was based on a total of 50,000 iterations with a burn-in of 25,000 iterations. We assessed convergence using Brooks–Gelman–Rubin plots.^2^ The consistency assumption for NMA was assessed by comparing the DIC of the consistency model with that of unrelated means model and by inspection of a scatterplot of residuals from the two analyses. All NMAs were performed using WinBUGS Software (WinBUGS version 1.4.3, Imperial College and Medical Research Council (MRC) Biostatistics Unit, UK) and R Software (R version 3.5.2, The R Foundation for Statistical Computing). Network geometry was evaluated graphically through preparation of network diagrams, and through inspection of the patterns of treatment comparisons (versus control therapies such as counselling/OAT only, as well as comparisons between different forms of intervention).

**Subgroup and Sensitivity Analyses**

Primary analyses were planned to be unadjusted. To explore whether primary findings were affected by specific traits of the included studies, sensitivity analyses (including subgroup analyses and network meta-regression) were planned to be conducted where sufficient data were available. A priori plans to explore subgroup analyses related to level of psychosocial intervention (e.g., individual, family, couple, and group) and within specific groups defined by age (i.e. youths aged 12-17 years and adults aged 18-25 years) were not feasible due to insufficient data. Analyses involving NMA meta-regression adjusting for sex (percent of males), average duration of OUD, most common method of administration (proportion intravenous use), type of opioid use (proportion illicit), percent with physical health comorbidities, percent with other substance/alcohol use, extent of comorbid pain, and percent of participants with mental health comorbidities were also planned, however, sufficient data was only available for a meta-regression adjusted for sex. In addition to the a priori planned subgroup and sensitivity analyses, we also conducted meta-regressions adjusting for average age, study duration (number of study weeks), and control group event rates. We also conducted a subgroup analysis excluding any articles that were published in potentially predatory journals (i.e., journals that accept articles for publication and collect fees from authors, without having performed quality checks for issues such as plagiarism or ethical approval) (see **S5 Text** for protocol deviations).^3^ Univariate meta-regressions adjusting for study duration and for incorporating baseline risk adjustment were both performed using a non-informative prior distribution for the slope parameter (i.e. Uniform(-1,1)). The importance of meta-regression adjustments was assessed by considering impact on DIC, magnitude of the between-study heterogeneity measure and whether the 95% credible interval for the regression parameter excluded.

References

1. Philippo D, Ades A, Dias S, Palmer S, Abrams K, Welton N. Nice Dsu Technical Support Document 18: Methods For Population-Adjusted Indirect Comparisons In Submissions To Nice. 2016.
2. Gelman A, Rubin DB. Inference from iterative simulation using multiple sequences. Statistical science. 1992;7(4):457-472.
3. Grudniewicz A, Moher D, Cobey KD, et al. Predatory journals: no definition, no defence. In: Nature Publishing Group; 2019.
