# Supplementary material for: Evaluating comparative effectiveness of psychosocial interventions adjunctive to opioid agonist therapy for opioid use disorder: A systematic review with network meta-analyses
Source: PLoS One. 2020 Dec 28;15(12):e0244401. doi: 10.1371/journal.pone.0244401 (PMC7769275; doi:10.1371/journal.pone.0244401)
Supplement: S5 Text — (DOCX) [file pone.0244401.s006.docx]

**S5 Text: Protocol Deviations**

While conducting risk of bias assessments for included articles, we noticed substantive errors in one article and discrepancies between values reported in tables and text in a second article. We reviewed the journals where these articles had been published and noticed that they appeared to be published in potentially predatory journals. To systematically assess articles published in potentially predatory journals, the types of journal where articles were published were extracted (i.e., subscription, hybrid, and exclusively open access journals). One reviewer then cross-checked all open access journals against the Directory of Open Access Journals (DOAJ) in addition to reviewing journal websites for key characteristics of potentially predatory journals. We conducted a secondary sensitivity analysis which excluded articles published in potentially predatory journals. We also conducted three meta-regressions for our NMA that had not been planned in our protocol in order to adjust for other key characteristics including average age of participants, number of study weeks, and control group risk.
