# Supplementary material for: Evaluating comparative effectiveness of psychosocial interventions adjunctive to opioid agonist therapy for opioid use disorder: A systematic review with network meta-analyses
Source: PLoS One. 2020 Dec 28;15(12):e0244401. doi: 10.1371/journal.pone.0244401 (PMC7769275; doi:10.1371/journal.pone.0244401)
Supplement: S6 Text — (DOCX) [file pone.0244401.s007.docx]

**S6 Text: Outcomes Reported by Study**

The summary table below provides a detailed account of the reporting of outcomes across the set of 71 included studies. Readers should note that, as described in the review’s main text, there was considerable variability in assessment methods, reporting format and follow-up duration amongst many of the studies that reported common endpoints. For these reasons, in many cases meta-analyses were considered to be unreliable and descriptive summaries were instead used to synthesize available data.

| **First Author** | **Year** | **Treatment Retention** | **Opioid use** | **Changes in Quality of Life** | **HIV/HCV Risk Behaviour** | **Abstinence from illicit drugs** | **Drug use** | **Mental health symptoms** | **Drop-outs/Attrition** | **Adverse events** | **Adherence to OAT** | **Withdrawal Symptoms** | **Alcohol** | **Measure of Craving** | **Relapse prevention** | **Abstinence from Opioid drugs** |
| --- | --- | --- | --- | --- | --- | --- | --- | --- | --- | --- | --- | --- | --- | --- | --- | --- |
| Nyamathi | 2011 |  |  |  |  |  | x |  |  |  |  |  |  |  |  |  |
| Day | 2018 | x |  |  |  |  |  | x |  |  |  | x |  |  |  | x |
| Pashaei | 2013 |  |  |  |  |  |  |  |  |  |  |  |  |  | x |  |
| Jiang | 2012 | x | x |  |  |  | x | x |  |  | x |  | x |  |  | x |
| Pan | 2015 | x |  |  |  |  |  | x |  |  | x |  | x |  |  | x |
| Stein | 2015 | x | x |  |  |  |  |  |  |  |  |  |  |  |  | x |
| Hosseinzadeh Asl | 2014 |  |  |  |  |  |  | x |  |  |  |  |  |  |  |  |
| Otto | 2014 | x |  |  |  |  | x |  | x |  |  |  |  |  |  |  |
| Christensen | 2014 | x |  |  |  | x | x |  |  |  |  |  |  |  |  |  |
| Marsch | 2014 | x |  |  |  |  |  |  |  |  |  |  |  |  |  | x |
| Jaffray | 2014 | x | x |  |  |  | x |  |  |  |  |  |  |  |  |  |
| Chen | 2013 | x |  |  |  |  |  |  |  |  | x |  |  |  |  | x |
| Ling | 2013 | x | x |  |  |  | x |  |  | x | x | x |  | x |  | x |
| Gu | 2013 | x |  |  |  |  |  |  |  |  | x |  |  |  |  |  |
| Moore | 2013 | x | x |  |  | x |  |  |  |  |  |  |  |  |  | x |
| Fiellin | 2013 | x | x |  |  | x |  |  |  |  | x |  |  |  |  | x |
| Tetrault | 2012 | x |  |  | x | x |  |  |  |  | x |  |  |  |  | x |
| Tuten | 2012 | x |  |  |  | x |  |  |  |  |  |  |  |  |  | x |
| Kelly | 2012 | x |  |  | x |  |  |  |  |  |  |  |  |  |  |  |
| Miotto | 2012 | x |  |  |  |  |  |  |  |  |  |  |  |  |  | x |
| Schwartz | 2012 | x | x |  |  |  | x |  |  | x |  |  | x |  |  |  |
| Hser | 2011 | x | x |  |  |  |  |  |  |  |  |  |  |  |  | x |
| Chawarski | 2011 |  | x |  | x |  |  |  |  |  | x |  |  |  |  |  |
| Karow | 2010 |  |  | x |  |  |  |  |  |  |  |  |  |  |  |  |
| Czuchry | 2009 |  | x |  |  |  | x |  |  |  |  |  |  |  |  |  |
| Chopra | 2009 | x |  |  |  | x | x |  |  |  |  |  |  |  |  | x |
| Epstein | 2009 | x |  |  |  | x |  |  |  |  |  | x |  |  |  | x |
| Bickel | 2008 | x |  |  |  | x |  |  |  |  |  |  |  |  |  |  |
| Chawarski | 2008 | x |  |  | x |  |  |  |  |  |  |  |  |  |  | x |
| Ghitza | 2008 | x |  |  | x | x |  |  |  |  |  |  |  |  |  |  |
| Ball | 2007 | x |  |  |  |  | x | x |  |  |  |  |  |  |  |  |
| Brooner | 2007 | x |  |  |  | x |  |  |  |  |  |  |  |  |  | x |
| Sullivan | 2006 | x | x |  |  |  |  |  |  |  |  |  |  |  |  |  |
| Fiellin | 2006 | x | x |  |  | x | x |  |  |  |  |  |  |  |  | x |
| Gross | 2006 | x |  |  |  | x | x |  |  |  |  |  | x |  |  |  |
| Poling | 2006 | x | x |  |  | x | x | x |  |  |  |  |  |  |  | x |
| Scherbaum | 2005 | x | x |  |  |  | x |  |  |  | x |  |  |  |  |  |
| Oliveto | 2005 | x |  |  |  | x |  | x |  |  |  | x |  |  |  | x |
| Schottenfeld | 2005 | x |  |  |  | x |  |  |  |  |  |  |  |  |  | x |
| Silverman | 2004 | x |  |  |  | x | x |  |  | x |  |  |  |  |  | x |
| Kosten | 2003 | x |  |  |  | x |  | x |  |  |  | x |  |  |  | x |
| Pollack | 2002 | x |  |  |  | x | x | x |  |  |  |  |  |  |  |  |
| Preston | 2002 | x | x |  |  | x |  |  |  |  |  |  |  |  |  | x |
| Linehan | 2002 | x | x |  |  | x | x |  |  |  |  |  |  |  |  | x |
| Petry | 2002 | x |  |  |  | x | x |  |  |  |  |  | x |  |  | x |
| Downey | 2000 | x |  |  |  | x | x |  |  |  |  |  | x |  |  | x |
| Preston | 2000 | x | x |  |  | x |  |  |  |  |  |  | x | x |  | x |
| Catalano | 1999 |  | x |  |  |  | x |  |  |  |  |  |  |  |  |  |
| Chutuape | 1999 |  |  |  |  | x | x |  |  |  |  |  |  |  |  |  |
| Avants | 1999 | x |  |  |  | x |  |  |  |  | x |  |  |  |  | x |
| O'Connor | 1998 | x | x |  |  |  | x |  |  |  |  | x |  |  |  | x |
| Abbott | 1998 |  |  |  | x |  | x | x |  |  |  |  | x |  |  | x |
| Joe | 1997 | x | x |  | x |  | x |  |  |  |  |  |  |  |  |  |
| Iguchi | 1997 | x |  |  |  | x |  |  |  |  |  |  |  |  |  |  |
| O'Neill | 1996 | x |  |  | x |  | x |  |  |  |  |  |  |  |  |  |
| Woody | 1995 | x | x |  |  |  | x | x |  |  |  |  | x |  |  |  |
| McLellan | 1993 |  | x |  |  | x | x |  |  |  |  |  |  |  |  | x |
| Woody | 1987 |  | x |  |  |  | x |  |  |  |  |  |  |  |  |  |
| Rounsaville | 1983 | x |  |  |  |  | x | x | x |  |  |  | x |  |  |  |
| Amini-Lari | 2017 | x | x |  |  |  |  |  |  |  |  | x |  |  |  |  |
| Abrahms | 1979 | x |  |  |  |  | x | x |  |  |  |  |  |  |  |  |
| Carroll | 1995 | x | x |  |  |  | x |  |  |  |  |  |  |  |  |  |
| Fals-Stewart | 2001 | x | x |  |  |  | x |  |  |  |  |  |  |  |  |  |
| Milby | 1979 |  |  |  |  | x |  |  |  |  |  |  |  |  |  |  |
| Rowan-Szal | 1997 | x | x |  |  |  | x |  |  |  |  |  |  |  |  |  |
| Salehi | 2018 | x |  |  | x | x |  |  |  |  |  |  |  |  |  |  |
| Moore | 2019 | x |  |  |  | x |  |  |  |  |  |  |  |  | x |  |
| Barry | 2019 | x |  |  |  |  |  |  |  |  |  |  |  |  |  | x |
| Kidorf | 2018 | x | x |  |  |  | x |  |  |  |  |  | x |  |  |  |
| Liu | 2018 | x |  |  |  |  |  |  |  | x | x |  |  |  |  | x |
| Yaghubi | 2017 |  |  |  |  |  |  |  |  |  |  |  |  |  |  |  |
| Shi | 2020 | x |  |  |  | x |  |  |  |  | x |  |  |  |  | x |
