# Supplementary material for: Evaluating comparative effectiveness of psychosocial interventions adjunctive to opioid agonist therapy for opioid use disorder: A systematic review with network meta-analyses
Source: PLoS One. 2020 Dec 28;15(12):e0244401. doi: 10.1371/journal.pone.0244401 (PMC7769275; doi:10.1371/journal.pone.0244401)
Supplement: S7 Text — (DOCX) [file pone.0244401.s008.docx]

**S7 Text: Risk of Bias Evaluations**

Findings from risk of bias evaluations of the included studies using the Cochrane Risk of Bias Scale are presented below, by clinical outcome.

| Author name, year | Sequence generation (selection bias) | | Allocation concealment (selection bias) | Blinding of participants and personnel | Blinding of outcome assessors (detection bias) | Incomplete outcome data addressed (attrition bias) | Selective outcome reporting (reporting bias) | Other sources of bias |
| --- | --- | --- | --- | --- | --- | --- | --- | --- |
| **Critical Outcome 1: Treatment Retention** | | | | | | | | |
| **Abrahms, 1979** | | ? | ? | - | + | N/A | ? | + |
| **Amini-Lari, 2017** | | + | ? | - | + | N/A | ? | + |
| **Avants, 1999** | | + | ? | - | + | N/A | ? | ? |
| **Ball, 2007** | | ? | ? | - | + | N/A | ? | + |
| **Barry, 2019** | | ? | + | - | + | N/A | + | + |
| **Bickel, 2008** | | + | ? | - | + | N/A | ? | + |
| **Brooner, 2007** | | ? | ? | - | + | N/A | ? | ? |
| **Carroll, 1995** | | ? | ? | - | + | N/A | - | ? |
| **Chawarski, 2008** | | + | ? | - | + | N/A | ? | + |
| **Chen, 2013** | | ? | ? | - | + | N/A | ? | + |
| **Chopra, 2009** | | + | ? | - | + | N/A | - | - |
| **Christensen, 2014** | | + | - | - | + | N/A | ? | - |
| **Day, 2018** | | + | + | - | + | N/A | - | ? |
| **Downey, 2000** | | - | - | - | + | N/A | - | - |
| **Epstein, 2009** | | + | - | - | + | N/A | ? | - |
| **Fals-Stewart, 2001** | | ? | ? | - | + | N/A | - | + |
| **Fiellin, 2013** | | + | + | - | + | N/A | + | - |
| **Fiellin, 2006** | | + | ? | - | + | N/A | - | + |
| **Ghitza, 2008** | | ? | ? | - | + | N/A | ? | - |
| **Groß (Gross), 2006** | | + | ? | - | + | N/A | ? | + |
| **Gu, 2013** | | + | + | - | + | N/A | ? | + |
| **Hser, 2011** | | + | ? | - | + | N/A | - | + |
| **Iguchi, 1997** | | ? | ? | - | + | N/A | - | + |
| **Jaffray, 2014** | | ? | ? | - | + | N/A | ? | - |
| **Jiang, 2012** | | + | ? | - | + | N/A | ? | - |
| **Joe, 1997** | | ? | ? | - | + | N/A | ? | - |
| **Kelly, 2012** | | ? | + | - | + | N/A | - | - |
| **Kidorf, 2018** | | + | ? | - | + | N/A | + | + |
| **Kosten, 2003** | | ? | ? | - | + | N/A | - | + |
| **Linehan, 2002** | | + | ? | - | + | N/A | - | - |
| **Ling, 2013** | | + | ? | - | + | N/A | ? | ? |
| **Liu, 2018** | | + | ? | - | + | N/A | + | ? |
| **Marsch, 2014** | | ? | ? | - | + | N/A | ? | ? |
| **Miotto, 2012** | | - | ? | - | + | N/A | ? | - |
| **Moore, 2013** | | ? | ? | - | + | N/A | - | - |
| **Moore, 2019** | | ? | ? | - | + | N/A | + | + |
| **O’Connor, 1998** | | ? | ? | - | + | N/A | ? | ? |
| **O’Neil, 1996** | | ? | ? | - | - | N/A | ? | ? |
| **Oliveto, 2005** | | ? | ? | - | + | N/A | - | - |
| **Otto, 2014** | | ? | ? | - | + | N/A | - | + |
| **Pan, 2015** | | + | - | - | + | N/A | + | - |
| **Petry, 2002** | | + | ? | - | + | N/A | - | + |
| **Poling, 2006** | | + | ? | - | + | N/A | ? | + |
| **Pollack, 2002** | | + | ? | - | + | N/A | - | ? |
| **Preston, 2002** | | ? | ? | - | + | N/A | ? | - |
| **Preston, 2000** | | + | - | - | + | N/A | ? | + |
| **Rounsaville, 1983** | | ? | ? | - | + | N/A | ? | + |
| **Rowan-Szal, 1997** | | ? | ? | - | + | N/A | ? | ? |
| **Salehi, 2018** | | + | - | - | + | N/A | ? | - |
| **Scherbaum, 2005** | | + | ? | - | + | N/A | - | + |
| **Schottenfeld, 2005** | | + | ? | - | + | N/A | - | - |
| **Schwartz, 2012** | | + | + | - | + | N/A | + | - |
| **Shi, 2020** | | + | ? | - | + | N/A | ? | + |
| **Silverman, 2004** | | + | - | - | + | N/A | ? | + |
| **Stein. 2015** | | + | ? | - | + | N/A | - | ? |
| **Sullivan, 2006** | | ? | ? | - | + | N/A | ? | - |
| **Tetrault, 2012** | | ? | ? | - | + | N/A | - | ? |
| **Tuten, 2012** | | ? | ? | - | + | N/A | - | + |
| **Woody, 1995** | | ? | ? | - | + | N/A | ? | + |
| **Critical Outcome 2: Adherence to OAT** | | | | | | | | |
| **Avants, 1999** | + | | ? | - | ? | ? | - | ? |
| **Chawarski, 2011** | + | | + | - | + | N/A^*^ | + | - |
| **Chen, 2013** | ? | | ? | - | + | + | ? | + |
| **Gu, 2013** | + | | + | - | + | + | ? | + |
| **Fiellin, 2006** | + | | ? | - | + | + | - | + |
| **Jiang, 2012** | + | | ? | - | + | N/A* | ? | - |
| **Ling, 2013** | + | | ? | - | + | - | ? | ? |
| **Moore, 2019** | ? | | ? | - | + | ? | + | + |
| **Pan, 2015** | + | | - | - | + | ? | + | - |
| **Scherbaum, 2005** | + | | ? | - | + | + | - | + |
| **Shi, 2020** | + | | ? | - | + | - | ? | + |
| **Tetrault, 2012** | ? | | ? | - | + | + | - | ? |
| **Critical Outcome 3: Opioid Use** | | | | | | | | |
| **Abbott, 1998** | + | | ? | - | + | + | ? | - |
| **Amini-Lari, 2017** | + | | ? | - | - | + | + | + |
| **Avants, 1999** | + | | ? | - | + | ? | ? | ? |
| **Barry, 2019** | ? | | + | - | + | ? | + | + |
| **Brooner, 2007** | ? | | ? | - | + | + | ? | ? |
| **Carroll, 1995** | ? | | ? | - | + | - | ? | ? |
| **Catalano, 1999** | + | | ? | - | - | + | ? | - |
| **Chawarski, 2011** | + | | + | - | + | + | - | - |
| **Chawarski, 2008** | + | | ? | - | + | + | ? | + |
| **Chen, 2013** | ? | | ? | - | + | + | ? | + |
| **Chopra, 2009** | + | | ? | - | + | ? | - | - |
| **Czuchry, 2009** | ? | | ? | - | + | - | - | - |
| **Day, 2018** | + | | + | - | ? | + | - | ? |
| **Downey, 2000** | - | | - | - | + | + | ? | - |
| **Epstein, 2009** | + | | - | - | + | + | - | - |
| **Fals-Stewart, 2001** | ? | | ? | - | + | - | ? | + |
| **Fiellin, 2013** | + | | + | - | + | + | + | - |
| **Fiellin, 2006** | + | | ? | - | + | + | + | + |
| **Hser, 2011** | + | | ? | - | + | + | - | + |
| **Jaffray, 2014** | ? | | ? | - | - | + | ? | - |
| **Jiang, 2012** | + | | ? | - | + | + | - | - |
| **Joe, 1997** | ? | | ? | - | + | - | ? | - |
| **Kidorf, 2018** | + | | ? | - | + | + | + | + |
| **Kosten, 2003** | ? | | ? | - | + | + | - | + |
| **Linehan, 2002** | + | | ? | - | + | + | ? | - |
| **Ling, 2013** | + | | ? | - | + | + | ? | ? |
| **Liu, 2018** | + | | ? | - | + | + | + | ? |
| **Marsch, 2014** | ? | | ? | - | + | + | ? | ? |
| **McLellan, 1993** | ? | | ? | - | + | - | ? | ? |
| **Miotto, 2012** | - | | ? | - | + | - | ? | - |
| **Moore, 2013** | ? | | ? | - | + | + | - | - |
| **O’Connor, 1998** | ? | | ? | - | + | + | - | ? |
| **Oliveto, 2005** | ? | | ? | - | + | + | - | - |
| **Pan, 2015** | + | | - | - | + | + | - | - |
| **Petry, 2002** | + | | ? | - | + | ? | ? | + |
| **Poling, 2006** | + | | ? | - | + | + | ? | + |
| **Preston, 2002** | ? | | ? | - | + | - | - | - |
| **Preston, 2000** | + | | - | - | + | + | ? | + |
| **Rowan-Szal, 1997** | ? | | ? | - | + | + | ? | ? |
| **Salehi, 2018** | + | | - | - | + | - | ? | ? |
| **Scherbaum, 2005** | + | | ? | - | + | + | - | + |
| **Schottenfeld, 2005** | + | | ? | - | + | + | + | - |
| **Schwartz, 2012** | + | | + | - | + | - | + | - |
| **Shi, 2020** | + | | ? | - | + | - | ? | + |
| **Silverman, 2004** | + | | - | - | + | + | ? | + |
| **Stein, 2015** | + | | ? | - | + | + | - | ? |
| **Sullivan, 2006** | ? | | ? | - | + | + | ? | - |
| **Tetrault, 2012** | ? | | ? | - | + | + | - | ? |
| **Tuten, 2012** | ? | | ? | - | + | - | - | + |
| **Woody, 1995** | ? | | ? | - | + | + | - | + |
| **Woody, 1987** | ? | | ? | - | - | - | ? | ? |
| **Critical Outcome 4: Adverse Events** | | | | | | | | |
| **Ling, 2013** | + | | ? | - | ? | + | ? | ? |
| **Moore, 2019** | ? | | ? | - | ? | ? | ? | + |
| **Silverman, 2004** | + | | - | - | ? | ? | ? | + |
| **Schwartz, 2012** | + | | + | - | + | + | ? | - |
| **No Critical Outcomes** | | | | | | | | |
| **Chutuape, 1999** | ? | | ? | - | N/A | N/A | N/A | - |
| **Hosseinzadeh, 2014** | ? | | ? | - | N/A | N/A | N/A | ? |
| **Karow, 2010** | ? | | ? | - | N/A | N/A | N/A | + |
| **Milby, 1978** | + | | ? | - | N/A | N/A | N/A | ? |
| **Nyamathi, 2011** | ? | | ? | - | N/A | N/A | N/A | ? |
| **Pashaei, 2013** | ? | | ? | - | N/A | N/A | N/A | ? |
| **Yaghubi, 2017** | + | | ? | - | N/A | N/A | N/A | - |

*Note:* * = Outcome was not applicable as it was measuring attrition (e.g., adherence to opioid agonist therapy as measured by participants that continued to receive treatment for 6 months).
