# Supplementary material for: Evaluating comparative effectiveness of psychosocial interventions adjunctive to opioid agonist therapy for opioid use disorder: A systematic review with network meta-analyses
Source: PLoS One. 2020 Dec 28;15(12):e0244401. doi: 10.1371/journal.pone.0244401 (PMC7769275; doi:10.1371/journal.pone.0244401)
Supplement: S8 Text — (DOCX) [file pone.0244401.s009.docx]

| **S8 Text: Overview of Findings by Study, *Treatment Retention (not included in network meta-analyses)*** | | | | | | | |  |
| --- | --- | --- | --- | --- | --- | --- | --- | --- |
| **Author, Year** | **Outcome Description** | | **Control Group:** N | **Control Group:** Frequency of Individuals N (%) | **Intervention Group:** N | **Intervention Group:** Frequency of Individuals N (%) | **Author Reported Conclusions** | **Final Timepoint (Weeks)** |
| *Number of Individuals Retained at Final Timepoint* | | | | | | | | |
| Stein, 2015* | Number of individuals retained at final study timepoint. | | C+Ed: 25 | 19 (76.0%) | C+ACT: 24 | 18 (75.0%) | Not Reported | 12 |
| Kosten, 2003 | Number of individuals retained at final study timepoint. | | C: 40 | N/A | C+CM: 40 | N/A | No significant differences for treatment retention were found between groups (p>.05). | 12 |
| *Number of Patients Retained in OAT* | | |  | | |  |  |  |
| Shi, 2020 | | Number of individuals retained in OAT. | OAT: 10 | 8 (80.0%) | CBT: 10 | 9 (90.0%) | Not Reported | 12 |
| *Number of Individuals Achieving Minimum Participation* | | | | | |  |  |  |
| Pollack, 2002 | Patients who achieved at least minimal participation in treatment (attending greater than 50% of the 12 weekly sessions). | | C + CM: 11 | 11 (100.0%) | CM + CBT: 12 | 9 (75.0%) | Not Reported | 12 |
| *Rates of Group Therapy Attendance* | | |  | | |  |  |  |
| Abrahms, 1979 | Rates of attendance for group therapy. | | C: 7 | 69.6% | CBT: 7 | 93.7% | No significant differences for treatment retention were found between groups (p>.05). | 10 |
| **Author, Year** | **Outcome Description** | | **Control Group:** N | **Control Group:** Mean (SD) | **Intervention Group:** N | **Intervention Group:** Mean (SD) | **Author Reported Conclusions** | **Final Timepoint (Weeks)** |
| *Average Number of Days in Study* | | |  | | |  |  |  |
| Preston, 2002 | Average number of total days in treatment. | | C: 55 | Mean (SEM): 29.1 (1.5) | C +CM: 55 | Mean (SEM): 32.9 (0.8) | No significant differences for treatment retention were found between groups (p>.05). | 52 |
| Rowan-Szal, 1997 | Average number of days maintained in treatment. | | C: 22 | 165.1 (91.7) | C + CM: 24 | 184.6 (74.4) | No significant differences for treatment retention were found between groups (p>.05). | 12 |
| *Average Number of Weeks in Study* | | | | | | |  |  |
| Ball, 2007 | Average number of session weeks attended. | | C + 12 Step Facilitation Therapy: NR | N/A | C + DFST: NR | N/A | No significant differences for treatment retention were found between groups (p>.05). | 24 |
| Carroll, 1995 | Average number of weeks retained in study. | | C: 7 | 22.3 (7.1) | C + CM: 7 | 25.1 (6.8) | No significant differences for treatment retention were found between groups (p>.05). | 13-31 |
| *Average Number of Sessions Attended* | | | | | |  |  |  |
| Joe, 1997 | Average number of sessions attended. | | C: 99 | N/A | NLM: 81 | N/A | No significant differences for treatment retention were found between groups (p>.05). | Not reported |
| Woody, 1995 | Average number of sessions attended. | | C: 27 | 26.0 (6.0) | C + PSEP: 57 | 23.0 (6.0) | No significant differences for treatment retention were found between groups (p>.05). | 24 |

*Note.* *Study was eligible for network meta-analysis but was disconnected from the rest of the network. C = Counselling, CM = Contingency Management, DFST = Dual Focus Schema Therapy, NLM = Node-Link Mapping, NR = Not Reported, PSEP = Psychoanalytic Supportive-Expressive Psychotherapy, SEM = Standard Error of the Mean.
