# Supplementary material for: Evaluating comparative effectiveness of psychosocial interventions adjunctive to opioid agonist therapy for opioid use disorder: A systematic review with network meta-analyses
Source: PLoS One. 2020 Dec 28;15(12):e0244401. doi: 10.1371/journal.pone.0244401 (PMC7769275; doi:10.1371/journal.pone.0244401)
Supplement: S9 Text — (DOCX) [file pone.0244401.s010.docx]

**S9 Text: Overview of Findings by Study, Abstinence from Opioids**

| **Author, Year** | **Outcome Description** | **Control Group:** N | **Control Group:** Frequency of Urinalysis N (%) | **Intervention Group:** N | **Intervention Group:** Frequency of Urinalysis N (%) | **Author Reported Conclusions** | **Final Timepoint (Weeks)** | |
| --- | --- | --- | --- | --- | --- | --- | --- | --- |
| *Abstinence throughout Study* | | | | | | | |  |
| Stein, 2015 | Number of participants with no specimens positive for opioids during months 1-,2- or 3. | C + Ed: 25 | 4 participants (16.0%) | C + ACT: 24 | 6 participants (25.0%) | Not Reported | | 12 |
| *Number of Urinalysis Negative for Opioid (percent values are at final timepoint)* | | | | | | | |  |
| Epstein, 2009 | Percentage of urine specimens negative for opiates. | C: 31 | 15 (48.4%) | C + CM: 47 | 22 (46.8%) | No significant differences between groups were found (p>.05). | | 12 |
| Hser, 2011 | Percentage of negative samples. | OAT Only: 159 | 63 (39.6%) | CM: 160 | 73 (45.6%) | No significant differences between groups were found (p>.05). | | 12 |
| Chawarski, 2008 | Percentage of opiate negative urine toxicology screens final timepoint. | C: 12 | 9 (75.0%) | C + EMM: 12 | 11 (91.7%) | The C+EMM group had a significantly greater number of negative urine tests than the C group (p < 0.05). | | 12 |
| *Mean Percent of Urinalysis Negative for Opioids Over Study (no N or SD)* | | | | | | | | |
| Silverman, 2004 | Percentage of opiate-negative urine samples. | C: 26 | 36.0% | C + CM: 26 | 42.0% | The C+CM group had a significantly greater number of negative urine tests than the C group (p < 0.05).* | | 52 |
| Brooner, 2007 | Opioid-negative urine samples. | C: 59 | 74.0% | CM: 59 | 74.4% | No significant differences between groups were found (p>.05). | | 36 |
| Avants, 1999 | Patients with opiate-free urine samples. | C: 145 | 53.0% | C + CBT: 146 | 62.0% | No significant differences between groups were found (p>.05). | | 36 |
| Fiellin, 2006 | Percentage of opioid-negative urine specimens. | C: 56 | 40.0% | C + EMM: 56 | 40.0% | No significant differences between groups were found (p>.05). | | 24 |
| Oliveto, 2005 | Percentage of opioid-negative urine tests. | C: 35 | 52.0% | C + CM: 35 | 51.0% | No significant differences between groups were found (p>.05). | | 12 |
| Kosten, 2003 | Percent of opiate-free urine. | C + CBT: 40 | 54.0% | C + CBT + CM: 40 | 65.0% | No significant differences between groups were found (p>.05). | | 12 |
| Chopra, 2009 | Percent of opioid-free urine samples over the 12 weeks of the trial. | C: 37 | 72.0% | CM + CRA: 41 | 84.0% | The CM+CRA group had a significantly greater number of negative urine tests than the C group (p < 0.05). | | 12 |
| Chen, 2013 | The proportion of urine samples that were negative for morphine among all samples taken at the clinic. | OAT Only: 120 | 57.6% | CM: 126 | 68.3% | The CM group had a significantly greater number of negative urine tests than the OAT Only group (p < 0.05). | | 12 |
| Petry, 2002 | Percentage of urine samples that were negative for opioids, total at final timepoint. | C: 23 | 15 (65.2%) | C + CM: 19 | 15 (78.9%) | No significant differences between groups were found (p>.05). | | 12 |
| Moore, 2013 | Percent of urine screens negative for opioids. | C: 18 | 75.0% | C + CBT: 18 | 79.0% | No significant differences between groups were found (p>.05). | | 4 |
| *Percent of Study Weeks Abstinent from Opioids* | | | | | | | |  |
| Marsch, 2014 | Percentage of study weeks abstinent from opioids (opioid abstinence was based on urinalysis result for opiates, propoxyphene, and oxycodone such that all three tests needed to be negative for a designation of an opioid-negative result). | C: 80 | 37.0% | C+CBT: 80 | 48.0% | The C+CBT group had a significantly greater number of negative urine tests than the C group (p < 0.05). | | 52 |
| *Percent of Participants At least One Urinalysis Negative for Opioids* | | | | | | | |  |
| Downey, 2000 | Percentage of participants who produced at least one negative urine for heroin. | CBT: 21 | 16 (76.2%) | CBT + CM: 20 | 13 (65.0%) | No significant differences between groups were found (p>.05). | | 17 |

| **Author, Year** | **Outcome Description** | **Control Group:** N | **Control Group:** Frequency of Individuals N (%) | **Intervention Group 1:** N | **Intervention Group 1:** Frequency of Individuals N (%) | **Intervention Group 2:** N | **Intervention Group 2:** Frequency of Individuals N (%) | **Author Reported Conclusions** | **Final Timepoint (Weeks)** |
| --- | --- | --- | --- | --- | --- | --- | --- | --- | --- |
| *Consecutive Urines Abstinent from Opioids* | | | | | | | | |  |
| McLellan, 1993 | 16 or more consecutive opiate-free weeks. | OAT Only: 10 | 0 (0.0%) | BT: 29 | 8 (27.6%) | BT + FT + ES: 31 | 17 (54.8%) | The BT and BT+FT+ES groups resulted in greater urines negative for opioids as compared to OAT only (p<.05). | 24 |
| O’Connor, 1998 | Proportion of patients who achieved 3 or more consecutive weeks of abstinence from opioids, as determined by thrice weekly urine toxicology testing. | C: 23 | 10 (43.5%) | CBT: 23 | 3 (13.0%) | N/A | N/A | The C group had a significantly greater number of negative urine tests than the CBT group (p < 0.05). | 12 |
|  |  |  |  |  |  |  |  |  |  |
|  |  |  |  |  |  |  |  |  |  |

| **Author, Year** | **Outcome Description** | **Control Group:** N | **Control Group:** Mean (SD) | | **Intervention Group 1:** N | | **Intervention Group 1:** Mean (SD) | **Intervention Group 2:** N | **Intervention Group 2:** Mean (SD) | **Intervention Group 3:** N | **Intervention Group 3:** Mean (SD) | **Author Reported Conclusions** | **Final Timepoint (Weeks)** |
| --- | --- | --- | --- | --- | --- | --- | --- | --- | --- | --- | --- | --- | --- |
| *Consecutive Urines Negative for Opioids* | | | | | | | | | | |  |  |  |
| Ling, 2013 | Mean number of consecutive opioid-negative urine results over the number of tests possible. | C: 51 | 10.9 (10.7) | | C + CBT: 53 | | 10.0 (11.1) | C + CM: 49 | 14.0 (12.3) | CBT + CM: 49 | 14.1 (12.7) | No significant differences between groups were found (p>.05). | 52 |
| *Urinalysis Tests Negative for Opioids* | | | | | | | |  |  |  |  |  |  |
| Tuten, 2012 | Number of opiate-negative urine tests. | OAT Only: 43 | Least square mean (standard error): 18.1 (2.2) | | CM: 38 | | Least square mean (standard error): 17.4 (2.0) | N/A | N/A | N/A | N/A | No significant differences between groups were found (p>.05). | 13 |
| *Mean Maximum Consecutive Weeks Abstinent from Opioids* | | | | | | | | | | | |  |  |
| Poling, 2006 | Maximum number of consecutive weeks of continued opioid abstinence. | CBT: 24 | 3.4 | | | CBT+CM: 25 | 4.6 | N/A | N/A | N/A | N/A | The CBT+CM group had a significantly greater number of negative urine tests than the CBT group (p < 0.05). | 25 |
| Jiang, 2012 | Longest continuous period (in weeks) of negative urine tests over the study. | C: 80 | 13.0 (8.1) | | | C+ CM + MI: 80 | 15.4 (8.1) | N/A | N/A | N/A | N/A | No significant differences between groups were found (p>.05). | 24 |
| Barry, 2019 | Maximum number of consecutive weeks abstinent from non medical opioid use. Patients were classified as abstinent during weeks 1–4, 5–8, and 9–12 if they had four successive opioid negative weekly urine tests and no positive tests during each 4-week period. | C: 19 | 3.9 (3.3) | | | CBT: 21 | 6.1 (4.2) | N/A | N/A | N/A | N/A | No significant differences between groups were found (p>.05). | 16 |
| *Days Abstinence from Opioids* | | | | | | | | | | | |  |  |
| Day, 2018 | Median days abstinent from heroin. | C: 30 | Median (IQR): 25 (14,27) | | PGS: 27 | | 18 (8,27) | Median (IQR): C + BSBNT + NLM: 26 | 17 (11,23) | N/A | N/A | No significant differences between groups were found (p>.05). | 52 |
| *Mean Percent of Urinalysis Negative for Opioids Over Study* | | | | | | | | | | | | |  |
| Preston, 2002 | Percentage of opiate-negative urine specimens. | C: 55 | 45 (24.5) | | C + CM: 55 | | 50.5 (26.3) | N/A | N/A | N/A | N/A | No significant differences between groups were found (p>.05). | 52 |
| Linehan, 2002 | Percentage of clean urinalyses from heroin. | CVT: 12 | 53.3 (83.4) | | DBT: 11 | | 46.4 (68.3) | N/A | N/A | N/A | N/A | Not Reported | 52 |
| Pan, 2015 | Proportion of opiate-negative urine samples. | OAT Only: 120 | 63 (37) | | C + CBT: 120 | | 73 (29) | N/A | N/A | N/A | N/A | The C+CBT group had a significantly greater number of negative urine tests than the OAT only group (p < 0.05). | 26 |
| Abbott, 1998 | Percentage of negative opiate screens. | C: 63 | 20.9 (4.0) | | CRA: 100 | | 22.1 (5.6) | N/A | N/A | N/A | N/A | No significant differences between groups were found (p>.05). | 24 |
| Fiellin, 2013 | Percentage of opioid-negative urines. | C: 71 | N/A | | C + CBT: 70 | | N/A | N/A | N/A | N/A | N/A | No significant differences between groups were found (p>.05). | 24 |
| Schottenfeld, 2005 | Percentage of opiate-free urine tests. | CRA: 40 | 50.3 (33.5) | | CM + CRA: 40 | | 54.7 (32.4) | N/A | N/A | N/A | N/A | No significant differences between groups were found (p>.05). | 24 |
| Miotto, 2012 | Percent of opioid-negative urine tests. Proportion of negative urine tests over all tests possible. | C: 33 | N/A | | CBT: 33 | | N/A | N/A | N/A | N/A | N/A | No significant difference for the percent of urinalysis negative for opioids was found between groups (p>.05). | 20 |
| Liu, 2018 | Percentage of negative urine test results, drug abstinence was defined as the number and percentage of opioid-negative urine specimens during the 16-week trial. | OAT Only: 63 | | 67.4 (95% confidence interval, 60.1-74.2) | C+Ed: 62 | | 76.4 (95% confidence interval, 70.8-82.5) | N/A | N/A | N/A | N/A | No significant differences between groups were found (p>.05). | 16 |
| Tetrault, 2012 | Percentage of opioid-negative urines. | C: 25 | 63.3 (33.8) | | C + EMM: 22 | | 69 (30.6) | N/A | N/A | N/A | N/A | No significant differences between groups were found (p>.05). | 12 |
| Shi, 2020 | Percentage of opioid-negative urines. | OAT: 10 | 91 (20.8) | | CBT:10 | | 64 (36.6) | N/A | N/A | N/A | N/A | No significant differences between groups were found (p=.05). | 12 |
| Preston, 2000 | Percentage of opiate-negative urine specimens. | C: 28 | 33.8 (29.1) | | C + CM: 29 | | 47.4 (42.5) | N/A | N/A | N/A | N/A | The C+CM group had a significantly greater number of negative urine tests than the C (p < 0.05). | 8 |

*Note.* C = Counselling, CBT = Cognitive Behavioural Therapy, CM = Contingency Management, Ed= Education, OAT = Opioid Agonist Treatment, BT = Behavioural Therapy, FT = Family Therapy, ES = Employment Services, EMM = Enhanced Medical Management, CVT = Comprehensive Validation Therapy, DBT = Dialectical Behaviour Therapy, CRA = Community Reinforcement Approach, Ed = Education, ACT = Acceptance and Commitment Therapy, PGS = Personal Goal Setting, IQR = Interquartile Range. *The in-text results are described differently than the table results, we have used the table results as the values are present.
