# Supplementary material for: Evaluating comparative effectiveness of psychosocial interventions adjunctive to opioid agonist therapy for opioid use disorder: A systematic review with network meta-analyses
Source: PLoS One. 2020 Dec 28;15(12):e0244401. doi: 10.1371/journal.pone.0244401 (PMC7769275; doi:10.1371/journal.pone.0244401)
Supplement: S10 Text — (DOCX) [file pone.0244401.s011.docx]

| **S10 Text: Overview of Findings by Study, *Opioid Use – Urinalysis*** | | | | | | | | | | | | | | | | | | | |  | |  | | |  |  |
| --- | --- | --- | --- | --- | --- | --- | --- | --- | --- | --- | --- | --- | --- | --- | --- | --- | --- | --- | --- | --- | --- | --- | --- | --- | --- | --- |
| **Author, Year** | | | **Outcome Description** | | | | | **Control Group:** N | **Control Group:**  Opioid Use N (%) | | | **Intervention Group 1:** N | | **Intervention Group:** Opioid Use N (%) | | **Intervention Group 2:** N | | **Intervention Group:** Opioid Use (%) | | **Author Reported Conclusions** | | **Final Timepoint (Weeks)** | | |  |  |
| *Number of Urinalysis Positive for Opioid (percent values are at final timepoint)* | | | | | | | | | | | | | | | | | | | | | | | | |  |  |
| Linehan, 2002 | | | Opiate use across the treatment year. Each point represents the crude rate of opiate-positive tests defined as the ratio of the total number of opiate-positive tests divided by the total number of available tests (summing over the 3 measurements per subject and summing over subjects) for each treatment condition. These ratios were then converted to percentages. Results from the final time point are presented. | | | | | CVT: 12 | 4 (33%) | | | DBT: 11 | | 3 (27%) | | N/A | | N/A | | The DBT group had a significantly greater reduction in frequency of use than the CVT group (p < 0.05). | | 64 | | |  |  |
| Woody, 1995 | | | Percentage of opiate-positive urine samples. Results from the final time point are presented. | | | | | C: 27 | 12 (44.4%) | | | C + PSEP: 57 | | 21 (36.8%) | | N/A | | N/A | | No significant differences between groups were found (p>.05). | | 24 | | |  |  |
|  | | |  | | | | |  |  | | |  | |  | |  | |  | |  | |  | | |  |  |
| *Percent of Urinalysis Positive for Opioids Over Study* | | | | | | | | | | | | | | | | | | | | | | | | |  |  |
| Kidorf, 2018 | | | Urine samples were obtained under direct observation (through a one-way mirror) and tested at a certified laboratory that employed TLC and EMIT testing for the presence of opioids. | | | | | C: 69 | 742 (29%) | | | C+CM: 72 | | 772 (32%) | | N/A | | N/A | | No significant differences between groups were found (p>.05). | | 26 | | |  |  |
| Stein, 2015 | | | Urine toxicologic testing for opioids including morphine, heroin, oxycodone, and methadone was done at each assessment and physician visit. Measured as percentage with any self-reported opioid use or positive urine toxicology test. | | | | | C + Ed: 25 | 18 (72.0%) | | | C + ACT: 24 | | 15 (62.5%) | | N/A | | N/A | | No significant differences between groups were found (p>.05). | | 12 | | |  |  |
| Sullivan, 2006 | | | Urine toxicology test results were considered to be positive for opioids if the specimen was positive for morphine metabolites, methadone, or oxycodone. Cutoff levels were 300 ng/mL for opioids and 1000 ng/mL for methadone and oxycodone. Percent of opioid positive urinalysis. | | | | | C: 8 | N/A | | | C+ EMM: 8 | | N/A | | N/A | | N/A | | No significant differences between groups were found (p>.05). | | 12 | | |  |  |
|  | | |  | | | | |  |  | | |  | |  | |  | |  | |  | |  | | |  |  |
| *Mean Number of Urinalysis Positive for Opioids* | | | | | | | | | | | | | | | | | | | | | | | | |  |  |
| Fals-Stewart, 2001 | | | Mean number of opiate-positive urines per month. | | | | | CBT:17 | N/A | | | CBT + BCT: 19 | | N/A | | N/A | | N/A | | The CBT+BCT group had a significantly greater reduction in opiate positive tests than the CBT only group (p < 0.05). | | 16 | | |  |  |
| **Author, Year** | | **Outcome Description** | | **Control Group:** N | | **Control Group:** Mean (SD) | | **Intervention Group 1:** N | | | **Intervention Group 1:** Mean (SD) | | **Intervention Group 2:** N | | **Intervention Group 2:** Mean (SD) | | **Intervention Group 3:** N | | **Intervention Group 3:** Mean (SD) | | **Author Reported Conclusions** | | **Final Timepoint (Weeks)** |  |  |  |
| *Mean Proportion of Urine Samples Positive for Opioids* | | | | | | | | | | | | | | | | | | | |  | |  | |  | | |
| Ling, 2013 | | Mean percentage of opioid use - The TES calculated opioid use (opiate, oxycodone, propoxyphene, and/or methadone) as a percentage of the number of opioid-negative urine tests over the number of tests possible. | | | | C: 51 | | 0.6 (0.5) | C + CBT: 53 | | 0.6 (0.5) | | | C + CM: 49 | | 0.6 (0.5) | | CBT + CM: 49 | | 0.7 (0.4) | | No significant differences between groups were found (p>.05). | | 52 | | |
| Schwartz, 2012 | | Percentage of opiate-positive urine samples. | | | | OAT Only: 104 | | 0.46 (0.50) | C + CM: 99 | | 0.48 (0.51) | | | N/A | | N/A | | N/A | | N/A | | No significant differences between groups were found (p>.05). | | 52 | | |
| Joe, 1997 | | Opioid positive sample among individuals in treatment for at least 6 months. Tested for cocaine metabolites (using the enzyme multiplicating immunoassay technique) obtained at follow-up. Mean percentage of opioid use. | | | | C: 52 | | 0.44 (0.50) | NLM: 26 | | 0.54 (0.51) | | | N/A | | N/A | | N/A | | N/A | | The NLM group had a significantly greater reduction in opiate positive tests than the C only group (p < 0.05). | | 52 | | |
| Carroll, 1995 | | Percentage of opiate-positive urine samples. | | | | C: 7 | | 0.25 (0.23) | C + CM: 7 | | 0.31 (0.33) | | | N/A | | N/A | | N/A | | N/A | | No significant differences between groups were found (p>.05). | | Range: 13-31 | | |
|  | |  | | | |  | |  |  | |  | | |  | |  | |  | |  | |  | |  | | |
| Chawarski, 2011 | | Percentage of opiate (morphine) positive urine tests. | | | | OAT Only: 17 | | N/A | EMM: 20 | | N/A | | | N/A | | N/A | | N/A | | N/A | | The EMM group had a significantly greater reduction in opiate positive tests than the OAT only group (p < 0.001). | | 24 | | |
| Czuchry, 2009 | | Percentage of opiate-positive urine samples. | | | | C: 27 | | 0.85 (0.36) | C + NLM: 33 | | 0.76 (0.44) | | | N/A | | N/A | | N/A | | N/A | | No significant differences between groups were found (p>.05). | | 24 | | |
|  | |  | | | |  | |  |  | |  | | |  | |  | |  | |  | |  | |  | | |
| Poling, 2006 | | Rate of opiate-positive urine samples. | | | | CBT: 24 | | N/A | CBT+CM: 25 | | N/A | | | N/A | | N/A | | N/A | | N/A | | No significant differences between groups were found (p>.05). | | 25 | | |
| Rowan-Szal, 1997 | | Percentage of opiate-positive urine samples. | | | | C: 22 | | 0.6 (0.4) | C + CM: 24 | | 0.6 (0.5) | | | N/A | | N/A | | N/A | | N/A | | No significant differences between groups were found (p>.05). | | 24 | | |
| Scherbaum, 2005 | | Rate of opiate-positive urine samples. | | | | C: 32 | | 0.2 (0.3) | CBT: 41 | | 0.15 (0.32) | | | N/A | | N/A | | N/A | | N/A | | No significant differences between groups were found (p>.05). | | 24 | | |
| McLellan, 1993 | | Percentage of opiate-positive urine samples. | | | | OAT Only: 10 | | N/A | BT: 29 | | N/A | | | BT + FT + ES: 31 | | N/A | | N/A | | N/A | | The BT+FT+ES group showed significantly greater opiate use than the OAT only group (p<.05). | | 12 | | |
|  | |  | | | |  | |  |  | |  | | |  | |  | |  | |  | |  | |  | | |
| O’Connor, 1998 | | Rates of opioid-positive urine samples. | | | | C: 23 | | N/A | CBT: 23 | | N/A | | | N/A | | N/A | | N/A | | N/A | | The CBT group had a significantly greater reduction in opiate positive tests than the C group (p < 0.05). | | 12 | | |

*Note.* CVT = Comprehensive Validation Therapy, DBT = Dialectical Behaviour Therapy, C = Counselling, PSEP = Psychoanalytic Supportive-Expressive Psychotherapy, Ed = Education, ACT = Acceptance and Commitment Therapy, EMM = Enhanced Medical Management, CBT = Cognitive Behavioural Therapy, BCT = Behavioural Couples Therapy, OAT = Opioid Agonist Treatment, CM = Contingency Management, NLM = Node-Link Mapping, BT = Behavioural Therapy, FT = Family Therapy, ES = Employment Services
