# Supplementary material for: Evaluating comparative effectiveness of psychosocial interventions adjunctive to opioid agonist therapy for opioid use disorder: A systematic review with network meta-analyses
Source: PLoS One. 2020 Dec 28;15(12):e0244401. doi: 10.1371/journal.pone.0244401 (PMC7769275; doi:10.1371/journal.pone.0244401)
Supplement: S11 Text — (DOCX) [file pone.0244401.s012.docx]

| **S11 Text: Overview of Findings by Study, *Drug Use*** | | | | | | | | | | | | |  |  | | |  |  |
| --- | --- | --- | --- | --- | --- | --- | --- | --- | --- | --- | --- | --- | --- | --- | --- | --- | --- | --- |
| **Author, Year** | **Outcome Description** | | **Control Group:** N | **Control Group:** N (%) | | **Intervention Group 1:** N | | **Intervention Group 1:** N (%) | | | **Intervention Group 2:** N | | | **Intervention Group 2:** N (%) | | **Author Reported Conclusions** | | **Final Timepoint (Weeks)** |
| *Number of Urinalysis Positive for Cocaine (percent values are at final timepoint)* | | | | | | | | | | | | | |  | | |  |  |
| Woody, 1995 | Percentage of cocaine-positive urine samples. | | C: 27 | 6 (22.2%) | | C + PSEP: 57 | | | 7 (12.3%) | | N/A | | | N/A | | The C+PSEP group had significantly fewer results positive for drugs as compared to the C group (p<.05). | | 24 |
| McLellan, 1993 | Percentage of cocaine-positive urine samples. | | OAT Only: 10 | 6 (60.0%) | | BT: 29 | | | 10 (34.5%) | | BT + FT + ES: 31 | | | 8 (25.8%) | | No significant differences between groups were found (p>.05). | | 24 |
| *At Least One Urine Sample Positive for Illicit Drugs* | | | | | | | | | | | | | | | | | |  |
| Fiellin, 2006 | Proportion of patients with at least one cocaine-positive urine specimen. | | C: 56 | 50% | | C + EMM: 56 | | | 55% | | N/A | | | N/A | | No significant differences between groups were found (p>.05). | | 24 |
| Chutuape, 1999 | Percentage of patients that submitted a urine positive for opiates, cocaine, or benzodiazepines. | | C: 7 | 7 (100.0%) | | C + CM: 7 | | | 6 (85.7%) | | N/A | | | N/A | | Not Reported | | 12 |
| *Percent of Urinalysis Positive for Illicit Drugs (No N)* | | | | | | | | | | | | | | | |  | |  |
| Linehan, 2002 | Other drug use across the treatment year. Each point represents the crude rate of non-opioid positive tests defined as the ratio of the total number of positive tests for non-opioid drugs divided by the total number of available tests (summing over subjects) for each treatment condition. These ratios were then converted to percentages. The temporal trend for each group is characterized using a smoothing spline with 4 degrees of freedom. | | CVT: 12 | N/A | | DBT: 11 | | | N/A | | N/A | | | N/A | | No significant differences between groups were found (p>.05). | | 52 |
| Abrahms, 1979 | Percent of contaminated urines. | | C: 7 | | 9.0% | CBT: 7 | | | | 4.5% | | N/A | | N/A | No significant differences between groups were found (p>.05). | | | 10 |
| *Percent of Urine Samples Positive for Cocaine (No N)* | | | | | |  | | | |  | |  | |  |  | | |  |
| Poling, 2006 | The observed probability of a cocaine-positive sample. | | CBT: 24 | | N/A | CBT+CM: 25 | | | | N/A | | N/A | | N/A | The CBT+CM group had significantly fewer results positive for drugs as compared to the CBT group (p<.001). | | | 25 |
| O’Connor, 1998 | Proportion of urine toxicology screens positive for cocaine. | | C: 23 | 30.5% | | CBT: 23 | | | | 38.5% | | N/A | | N/A | Not Reported | | | 12 |
|  |  | |  |  | |  | | | |  | |  | |  |  | | |  |
| *Number of Urine Samples Positive for Illicit Drugs* | | | | | |  | | | |  | |  | |  |  | | |  |
| Kidorf, 2018 | Proportion of positive urine samples (positive for any drug). | | C: 69 | 1778 (63%) | | C+CM: 72 | | | | 1329 (52%) | | N/A | | N/A | The C+CM group had significantly fewer urine samples positive for drugs as compared to the C group (p<.01). | | | 26 |
| Rounsaville, 1983 | Number of urine specimens positive for illicit substances in past 3 months. | | C: 28 | N/A | | IPT: 22 | | | | N/A | | N/A | | N/A | No significant differences between groups were found (p>.05). | | | 24 |
| *Number of Urine Samples Positive for Cocaine* | | | | | | | | | | | | | | | | | |  |
| Rowan-Szal, 1997 | Cocaine positive urines. | | C: 22 | 65 (40) | | C + CM: 24 | | | | 38 (46) | | N/A | | N/A | The C+CM group had significantly fewer urine samples positive for cocaine as compared to the C group (p<.05). | | | 24 |
| *Number of Patients Who Used Illicit Drugs* | | | | | | |  | | |  | |  | |  |  | | |  |
| Jaffray, 2014 | Proportion of patients who used illicit drugs. | OAT Only: 153 | | 101 (67.8%)* | | MI: 182 | | | | 123 (68.3%)* | | N/A | | N/A | No significant differences between groups were found (p>.05). | | | 24 |
| Petry, 2002 | Number of patients who were unable to achieve a week of abstinence. | C: 23 | | 11 (47.8%) | | C + CM: 19 | | | | 3 (15.8%) | | N/A | | N/A | Not Reported | | | 52 |

| **Author, Year** | **Outcome Description** | | **Control Group:** N | **Control Group:** Mean (SD) | | **Intervention Group:** N | **Intervention Group:** Mean (SD) | | | | **Author Reported Conclusions** | | **Final Timepoint (Weeks)** |  |  |
| --- | --- | --- | --- | --- | --- | --- | --- | --- | --- | --- | --- | --- | --- | --- | --- |
| *Mean Percent of Urines Positive for Cocaine* | | | | |  | | | |  |  | | |  |  |  |
| Joe, 1997 | | Percentage of cocaine-positive urine samples at last time point. | C: 99 | | 0.38 (0.5) | NLM: 81 | 0.54 (0.5) | | | No significant differences between groups were found (p>.05). | | | 52 |  |  |
| Schwartz, 2012 | | Cocaine positive tests. | OAT Only: 104 | | 0.36 (0.5) | C + CM: 99 | 0.39 (0.5) | | | No significant differences between groups were found (p>.05). | | | 52 |  |  |
| Czuchry, 2009 | | Mean proportions and standard deviations for urinalysis of cocaine. | C: 27 | | 0.4 (0.5) | C + NLM: 33 | 0.4 (0.5) | | | No significant differences between groups were found (p>.05). | | | 24 |  |  |
| Scherbaum, 2005 | | Rate of cocaine-positive urine samples; measured by 5 randomised urine screens per month. | C: 32 | | 0.4 (0.4) | CBT: 41 | 0.16 (0.3) | | | The CBT group had significantly fewer urine samples positive for cocaine as compared to the C group (p<.05). | | | 24 |  |  |
| Carroll, 1995 | | Percentage of cocaine positive urinalysis results. | C: 7 | | 0.4 (0.36) | C + CM: 7 | 0.17 (0.36) | | | No significant differences between groups were found (p>.05). | | | NR |  |  |

*Note. **Numbers and percentages provided by authors do not align with calculated percentages, data taken directly from study. BT = Behavioural Therapy, CBT = Cognitive Behavioural Therapy, CVT = Comprehensive Validation Therapy, CM = Contingency Management, C = Counselling, NLM = Node-Link Mapping, DBT = Dialectical Behaviour Therapy, EMM = Enhanced Medical Management, ES = Employment Services, FT = Family Therapy, MI = Motivational Interviewing, IPT = Interpersonal Psychotherapy, NR = Not Reported, OAT = Opioid Agonist Treatment, PSEP = Psychoanalytic Supportive-Expressive Psychotherapy
