# Supplementary material for: Evaluating comparative effectiveness of psychosocial interventions adjunctive to opioid agonist therapy for opioid use disorder: A systematic review with network meta-analyses
Source: PLoS One. 2020 Dec 28;15(12):e0244401. doi: 10.1371/journal.pone.0244401 (PMC7769275; doi:10.1371/journal.pone.0244401)
Supplement: S12 Text — (DOCX) [file pone.0244401.s013.docx]

| **S12 Text: Overview of Findings by Study, *Self-Reported Illicit Drug Use*** | | | | | | | |  |  |  |  |
| --- | --- | --- | --- | --- | --- | --- | --- | --- | --- | --- | --- |
| **Author, Year** | **Outcome Description** | **Control Group:** N | **Control Group:** Mean (SD) | **Intervention Group 1:** N | **Intervention Group 1:** Mean (SD) | **Intervention Group 2:** N | **Intervention Group 2:** Mean (SD) | **Intervention Group 3:** N | **Intervention Group 3:** Mean (SD) | **Author Reported Conclusions** | **Final Timepoint (Weeks)** |
| *Addiction Severity Index* | | | | | |  |  |  |  |  |  |
| Ling, 2013 | Addiction severity index (ASI) - drug dimension score - specific to cocaine. Composite scores are calculated (between 0 and 1, with higher scores representing more severe addiction) for each of the seven dimensions. | C: 51 | N/A | C+ CBT: 53 | N/A | C + CM: 49 | N/A | CBT + CM: 49 | N/A | No significant differences between groups were found (p>.05). | 52 |
| Silverman, 2004 | ASI – composite drug dimension score. Higher scores represent more severe addiction. | C: 26 | N/A | C + CM: 26 | N/A | N/A | N/A | N/A | N/A | No significant differences between groups were found (p>.05). | 52 |
| Woody, 1987 | ASI – composite drug dimension score. Higher scores represent more severe addiction. | C: 31 | N/A | C + PSEP: 28 | N/A | C + CBT: 34 | N/A | N/A | N/A | No significant differences between groups were found (p>.05). | 52 |
| Nyamathi, 2011 | Addiction severity index (ASI) - Lite - drug dimension score. The drugs considered in this instrument were: heroin, methadone, opiates/analgesics, barbiturates, cocaine, amphetamines, cannabis, hallucinogens and inhalants. Higher scores represent more severe addiction. | Ed: 87 | 0.1 (1.5) | MI: 90 | 0.3 (1.7) | N/A | N/A | N/A | N/A | No significant differences between groups were found (p>.05). | 26 |
| Ball, 2007 | ASI – composite drug dimension score. Higher scores represent more severe addiction. | C + 12-Step Facilitation Therapy: Not Reported | N/A | C + DFST: NR | N/A | N/A | N/A | N/A | N/A | No significant differences between groups were found (p>.05). | 24 |
| Jiang, 2012 | ASI – composite drug dimension score. Higher scores represent more severe addiction. | C: 62 | 0.05 (0.08) | C + MI + CM: 63 | 0.03 (0.08) | N/A | N/A | N/A | N/A | No significant differences between groups were found (p>.05). | 24 |
| Abbott, 1998 | ASI – composite drug dimension score. Higher scores represent more severe addiction. | C: 55 | 0.16 (0.11) | CRA: 96 | 0.13 (0.09) | N/A | N/A | N/A | N/A | The CRA group had a significantly greater reduction in drug use than the C group (p < 0.05). | 24 |
| Pollack, 2002 | ASI – composite drug dimension score. Higher scores represent more severe addiction. | C+CM: 11 | N/A | CM+CBT: 12 | N/A | N/A | N/A | N/A | N/A | No significant differences between groups were found (p>.05). | 24 |
| Downey, 2000 | ASI – composite drug dimension score. Higher scores represent more severe addiction. | CBT: 21 | N/A | CM + CBT: 20 | N/A | N/A | N/A | N/A | N/A | No significant differences between groups were found (p>.05). | 17 |
| Fals-Stewart, 2001 | ASI – composite drug dimension score. Higher scores represent more severe addiction. | CBT: 17 | 0.28 (0.08) | CBT + BCT: 19 | 0.16 (0.09) | N/A | N/A | N/A | N/A | The CBT+BCT group had a significantly greater reduction in drug use than the CBT group (p < 0.05). | 16 |
| Otto, 2014 | ASI – composite drug dimension score. Higher scores represent more severe addiction. | C: 37 | N/A | CBT: 41 | N/A | N/A | N/A | N/A | N/A | No significant differences between groups were found (p>.05). | 12 |
| Christensen, 2014 | ASI – composite drug dimension score. Higher scores represent more severe addiction. | C + CM: 78 | N/A | C + CM + CRA: 92 | N/A | N/A | N/A | N/A | N/A | No significant differences between groups were found (p>.05). | 12 |
| Chopra, 2009 | ASI – composite drug dimension score. Higher scores represent more severe addiction. | C: 37 | N/A | CM + CRA: 41 | N/A | N/A | N/A | N/A | N/A | No significant differences between groups were found (p>.05). | 12 |
| Groß, 2006 | ASI – composite drug dimension score. Higher scores represent more severe addiction. | C: 20 | N/A | C + CM: 20 | N/A | N/A | N/A | N/A | N/A | No significant differences between groups were found (p>.05). | 12 |
|  |  |  |  |  |  |  |  |  |  |  |  |
| *Use of Cocaine in Past Month* | | | | | | | | | | |  |
| Catalano, 1999 | Mean times used cocaine in the previous month. | C: 55 | 12.2 (45.7) | C + SBPT: 75 | 1.8 (7.4) | N/A | N/A | N/A | N/A | The C+SBPT group had a significantly greater reduction in drug use than the C group (p < 0.05). | 52 |
| O'Neill, 1996 | The drug use subscale of the opioid treatment index. This scale assesses the subject’s use of 11 classes of drug in the month prior to the day of interview and provides an estimate of the number of episodes of use per day. | C: 40 | N/A | C + CBT: 40 | N/A | N/A | N/A | N/A | N/A | No significant differences between groups were found (p>.05). | 36 |

*Note.*  NR = Not Reported, Ed = Education, MI = Motivational Interviewing, C = Counselling, CM = Contingency Management, CBT = Cognitive Behavioural Therapy, CRA = Community Reinforcement Approach, DFST = Dual Focus Schema Therapy, PSEP = Psychoanalytic Supportive-Expressive Psychotherapy, BCT = Behavioural Couples Therapy, SBP = Skills Based Parental Training
