# Supplementary material for: Evaluating comparative effectiveness of psychosocial interventions adjunctive to opioid agonist therapy for opioid use disorder: A systematic review with network meta-analyses
Source: PLoS One. 2020 Dec 28;15(12):e0244401. doi: 10.1371/journal.pone.0244401 (PMC7769275; doi:10.1371/journal.pone.0244401)
Supplement: S13 Text — (DOCX) [file pone.0244401.s014.docx]

| **S13 Text: Overview of Findings by Study, *Abstinence from Illicit Drugs*** | | | |  |  |  |  |  |  |
| --- | --- | --- | --- | --- | --- | --- | --- | --- | --- |
| **Author, Year** | **Outcome Description** | **Control Group:** N | **Control Group:** Number of Urines Negative for Illicit Drugs N (%) | **Intervention Group 1:** N | **Intervention Group 1:** Number of Urines Negative for Illicit Drugs N (%) | **Intervention Group 2:** N | **Intervention Group 2:** Number of Urines Negative for Illicit Drugs N (%) | **Author Reported Conclusions** | **Final Timepoint (Weeks)** |
| *Percent of Urinalysis Negative for Illicit Drugs at Final Timepoint* | | | |  |  |  |  |  |  |
| Salehi, 2018 | Percent of negative urine test results at the end of the follow-up (drugs tested not specified). | C: 25 | 12 (48%) | C+CBT: 25 | 21 (84%) | N/A | N/A | The C+CBT group had a significantly greater number of negative urine tests than the C group (p < 0.05). | 25 |
| *Number of Participants with Consecutive Urinalysis Negative for Illicit Drugs* | | | |  |  |  |  |  |  |
| Milby, 1978 | Number of subjects who met the seven consecutive “clean” urine criteria. Specimens were collected on a random schedule once per week, and analyzed for amphetamines. quinine, methadone, barbiturates, methaqualone and diazepam. | OAT Only: 18 | 6 (33.3%) | CM: 51 | 24 (47.1%) | N/A | N/A | The CM group had a significantly greater number of negative urine tests than the OAT only group (p < 0.05). | 7 |
| *Proportion of Urine Samples Negative for Cocaine* | | |  |  |  |  |  |  |  |
| Oliveto, 2005 | Proportion of cocaine-negative urine tests. | C: 35 | 33.7% | C + CM: 35 | 51.0% | N/A | N/A | The C+CM group had a significantly greater number of negative urine tests than the C group (p < 0.05). | 12 |
| Moore, 2013 | Proportion of urine screen negative for cocaine. | C: 72 | 27.8% | C + CBT: 72 | 44.4% | N/A | N/A | No significant differences between groups were found (p>.05). | 4 |
| *Number of Urine Samples Negative for Cocaine (percent values represent the final timepoint)t* | | | | |  |  |  |  |  |
| Iguchi, 1997 | Percentage of drug-free urine samples. | C: 35 | 8 (22.9%) | C + CM: 27 | 7 (26.0%) | C + CRA: 41 | 14 (34.1%) | No significant differences between groups were found (p>.05). | 24 |
| McLellan, 1993 | Percentage abstinent from opiates and cocaine. | OAT Only: 10 | 3 (30.0%) | BT: 29 | 16 (55.2%) | BT + FT + ES: 31 | 21 (67.7%) | No significant differences between groups were found (p>.05). | 24 |
| *Percent of Urinalysis Negative for Cocaine at Final Timepoint* | | | |  |  |  |  |  |  |
| Avants, 1999 | Patients with cocaine-free urine samples; twice-weekly urine samples were analyzed for the presence of opiates (≥200 ng/ml) and the cocaine. | C: 145 | 75 (51.7%) | C +CBT: 146 | 79 (54.1%) | NA | NA | No significant differences between groups were found (p>.05). | 36 |
| *Percent of Urinalysis Negative from Drugs (No N)* | | | | |  |  |  |  |  |
| Brooner, 2007 | Percent of any drug-negative urine samples. Samples were tested for opiates, barbiturates, cocaine, alcohol, and some benzodiazepines. | C: 59 | 32.9% | CM: 59 | 45.8% | N/A | N/A | The CM group had a significantly greater number of negative urine tests than the C group (p < 0.05). | 36 |
| Pollack, 2002 | Percentage of negative illicit drug screens; The study utilized the ETS Plus EMIT system (Syva Co., Palo Alto, CA) for urine toxicology screens for opiates, methadone, cocaine, propoxyphene, benzodiazepines, amphetamines, THC, and barbiturates. One time point after 12 weeks was selected for this outcome. | C+CM: 11 | Men: 12.9%; Women: 16.2% | CM+CBT: 12 | Men: 5.3%; Women: 30.7% | N/A | N/A | The CM+CBT group had a significantly greater number of negative urine tests than the C+CM group for women (p < 0.05) but not men (p>.05). | 24 |
| Downey, 2000 | Cocaine abstinence rates for participants who achieved one or more poly-drug-free urine test results. Percent of drug free urines. | CBT: 21 | 38.3% | CM + CBT: 20 | 60.0% | N/A | N/A | No significant differences between groups were found (p>.05). | 17 |
| Petry, 2002 | Percentage of urine samples that were negative for opioids and cocaine. | C: 23 | 34% | C + CM: 19 | 52% | N/A | N/A | The C+CM group had a significantly greater number of negative urine tests than the C group (p < 0.05). | 12 |
| Ghitza, 2008 | Urine specimens negative for both cocaine and opiates. | C: 40 | 17.5% | C + CM: 76 | 23.0% | N/A | N/A | Not Reported* | 12 |
| *Consecutive Weeks Abstinent from Cocaine* | |  |  |  |  |  |  |  |  |
| Poling, 2006 | Number of weeks of consecutive cocaine abstinence based on urinalysis. | CBT: 24 | N/A | CBT+CM: 25 | N/A | N/A | N/A | No significant difference was found between groups (p>.05). | 25 |

| **Author, Year** | **Outcome Description** | **Control Group:** N | | **Control Group:** Mean (SD) | | **Intervention Group:** N | **Intervention Group:** Mean (SD) | **Author Reported Conclusions** | | **Final Timepoint (Weeks)** | |
| --- | --- | --- | --- | --- | --- | --- | --- | --- | --- | --- | --- |
| *Mean Longest Number of Consecutive Weeks Abstinent from Drugs* | | | | | | | |  | |  | |
| Silverman, 2004 | Longest duration of sustained abstinence from cocaine. | C: 26 | | 6.3 (6.9) | | C + CM: 26 | 18.8 (16) | The C+CM group resulted in significantly more drug-free weeks as compared to C (p<.05). | | 52 | |
| Fiellin, 2013 | Maximum number of weeks abstinent from cocaine use (verified by urinalysis); Urinalyses were conducted with the use of a semiquantitative homogeneous enzyme immunoassay for opioids, cocaine, oxycodone, methadone, marijuana, and benzodiazepines. | C: 71 | | N/A | | C + CBT: 70 | N/A | No significant difference between groups (p>.05). | | 24 | |
| Schottenfeld, 2005 | Mean maximum consecutive weeks of abstinence from cocaine and opiates. | CRA: 40 | | 4.3 (6.1) | | CM + CRA: 40 | 4.8 (6.1) | No significant difference between groups (p>.05). | | 24 | |
| Bickel, 2008 | Mean number of weeks continuous abstinence from opiates and cocaine | C: 34 | | 4.7 (5.4) | | CM + CRA: 30 | 7.8 (6) | The CM+CRA group resulted in significantly more drug-free weeks as compared to C (p<.05). | | 23 | |
| Epstein, 2009 | Longest weeks of simultaneous abstinence (abstinence from cocaine, opiates, benzodiazepines, and marijuana). | C: 31 | | 0.7 (1.3) | | C + CM: 47 | 2.7 (4.2) | The C+CM group resulted in significantly more drug-free weeks as compared to C (p<.05). | | 20 | |
| Chopra, 2009 | Longest continuous weeks abstinent from opioids and cocaine. Urine samples were screened for methadone, opiates, propoxyphene, and cocaine. | C: 37 | | 7.1 (4.4) | | CM + CRA: 41 | 7.8 (3.7) | The C+CM group resulted in significantly more drug-free weeks as compared to C (p<.05). | | 12 | |
| Groß, 2006 | Durations of continuous abstinence (weeks) from opioids and cocaine. Urine specimens screened for methadone, opiates, propoxyphene, and cocaine. | C: 20 | | 4 (3.2) | | C + CM: 20 | 2.9 (3.3) | No significant difference between groups (p>.05). | | 12 | |
| Chutuape, 1999 | Longest duration of drug-free weeks (presence of methadone, opiates, cocaine and benzodiazepines.) | C: 7 | | 1 (2.1) | | C + CM: 7 | 8.4 (3.2) | The C+CM group resulted in significantly more drug-free weeks as compared to C (p<.05). | | 12 | |
| *Mean Number of Days Abstinent from Drugs* | |  | |  | |  |  |  | |  | |
| Christensen, 2014 | Mean number of days abstinent from drugs. | C+CM: 78 | | 57.4 (28.0) | | C + CM + CRA: 92 | 67.1 (19.3) | The C+CM+CRA group resulted in significantly more drug-free weeks as compared to C (p<.05). | | 12 | |
| *Longest Number of Time Points Abstinent from Drugs* | |  | |  | |  |  |  | |  | |
| Tuten, 2012 | Longest number of time points with drug negative urine results. Urine samples were tested three times a week on Monday, Wednesday, and Friday. Urine samples were tested on Monday/Wednesday/Friday on-site under observed conditions for opioids and cocaine. | OAT Only: 43 | | Least square mean(standard error): 8.4 (1.5) | | CM: 38 | Least square mean(standard error): 8.5 (1.4) | No significant difference between groups (p>.05). | | 13 | |
| *Mean Percent Urine Samples Negative for Illicit Drugs* | | | | | | |  |  | |  | |
| Moore, 2019 | Percent of negative urine tests (urine samples collected at baseline and twice each week during treatment were screened for opiates, methadone, oxycodone, THC, cocaine metabolite, and benzodiazepines using RediTest (Redwood Toxicology Laboratories, Santa Rosa, CA). Cut off values established by the manufacturer were > 200 ng/ml for opiates and benzodiazepines, > 300 ng/ml for methadone and cocaine, > 100 ng/ml oxycodone, and > 50 ng/ml for THC). | C: 42 | 16.8 (23.0) | | C + CBT: 40 | | 16.7 (25.6) | | No significant difference between groups (p>.05). | 12 |  |
| Shi, 2020 | Percentage of urine toxicology screens negative for all drugs tested (iCup DX Drug Test Cup with Specimen Validity Test): amphetamines; barbiturates;  benzodiazepines; cocaine; methamphetamine; opiates; oxycodone; tetrahydrocannabinol) | OAT Only: 10 | 30 (38.1) | | CBT: 10 | | 82 (29.7) | | The CBT group had a significantly greater percentage of negative urine tests than the OAT Only (p < 0.05). | 12 |  |
| *Average Weeks of Continuous Abstinence* | |  | |  | |  |  |  | |  | |
| Kosten, 2003 | Average number of weeks of continuous abstinence from cocaine. | C + CBT: 40 | | 1.2 (2.5) | | C+CBT + CM: 40 | 1.2 (2.6) | No significant difference between groups (p>.05). | | 12 | |
| Linehan, 2002 | Percentage of clean urinalyses from cocaine. | CVT: 12 | | 72.3 (78.6) | | DBT: 11 | 70.9 (80.7) | Not Reported | | 52 | |
| Fiellin, 2006 | Percentage of cocaine-negative urine specimens. | C: 56 | | 71% (Confidence interval: 62.3-79.9) | | C + EMM: 56 | 73.6% (Confidence interval: 64.8-82.3) | No significant differences between groups were found (p>.05). | | 24 | |
| Tetrault, 2012 | Percent of cocaine negative urines. | C: 25 | | 62.4 (40.7) | | C + EMM: 22 | 55.6 (44.1) | No significant differences between groups were found (p>.05). | | 12 | |
| Preston, 2002 | Mean percentage of cocaine negative urine test. | C: 55 | | 36.6 (37.8) | | C + CM: 55 | 38.7 (39.3) | No significant differences between groups were found (p>.05). | | 12 | |
| Preston, 2000 | Percentage of cocaine-negative urine specimens. | C: 28 | | 34.8 (31.2) | | C + CM: 29 | 35.4 (39.9) | The C+CM group had a significantly greater number of negative urine tests than the C (p < 0.05). | | 13 | |
|  |  |  | |  | |  |  |  | |  | |

*Note.* C = Counselling, CM = Contingency Management, MI = Motivational Interviewing, CRA = Community Reinforcement Approach, CBT = Cognitive Behavioural Therapy, OAT = Opioid Agonist Treatment, EMM = Enhanced Medical Management, CVT = Comprehensive Validation Therapy, DBT = Dialectical Behaviour Therapy, BT = Behavioural Therapy, FT = Family Therapy, ES = Employment Services, *Only adjusted model significance values were reported.
