# Supplementary material for: Evaluating comparative effectiveness of psychosocial interventions adjunctive to opioid agonist therapy for opioid use disorder: A systematic review with network meta-analyses
Source: PLoS One. 2020 Dec 28;15(12):e0244401. doi: 10.1371/journal.pone.0244401 (PMC7769275; doi:10.1371/journal.pone.0244401)
Supplement: S14 Text — (DOCX) [file pone.0244401.s015.docx]

| **S14 Text: Overview of Findings by Study, *Mental Health*** | | | | |  |  |  |  |  |
| --- | --- | --- | --- | --- | --- | --- | --- | --- | --- |
| **Author, Year** | **Outcome Description** | **Control Group:** N | **Control Group:** Mean (SD) | **Intervention Group 1:** N | **Intervention Group 1:** Mean (SD) | **Intervention Group 2:** N | **Intervention Group 2:** Mean (SD) | **Author Reported Conclusions** | **Final Timepoint (Weeks)** |
| *Depression* |  |  |  |  |  |  |  |  |  |
| Woody, 1987 | Beck Depression Inventory. Greater scores represent greater endorsement of depressive symptoms. | C: 31 | 11.0 (3.0) | C + PSEP: 28 | 9.0 (2.0) | C + CBT: 34 | 9.0 (2.0) | The C+PSEP and C+CBT groups had a significantly greater reduction in depression scores as compared to the C group (p<.05). | 52 |
| Pollack, 2002 | Hamilton Rating Scale for Depression (HRSD) - Greater scores represent greater endorsement of depressive symptoms. | C + CM: 11 | N/A | CM + CBT: 12 | N/A | N/A | N/A | No significant differences between groups were found (p>.05). | 26 |
| Poling, 2006 | The Center for Epidemiological Studies Depression Scale (CES-D). Greater scores represent greater endorsement of depressive symptoms. | CBT: 24 | 0.61 (1.42) | CBT+CM: 25 | 0.25 (0.78) | N/A | N/A | No significant differences between groups were found (p>.05). | 25 |
| Abbott, 1998 | Beck Depression Inventory. Greater scores represent greater endorsement of depressive symptoms. | C: 55 | 8.0 (8.1) | CRA: 96 | 7.0 (7.9) | N/A | N/A | No significant differences between groups were found (p>.05). | 24 |
| Rounsaville, 1983 | Raskin Depression Scale (range 3-15, higher score = higher severity of depression) | C: 28 | N/A | IPT: 22 | N/A | N/A | N/A | No significant differences between groups were found (p>.05). | 24 |
| Kosten, 2003 | The Center for Epidemiological Studies Depression Scale (CES-D). Greater scores represent greater endorsement of depressive symptoms. | CM: 40 | N/A | C + CBT + CM: 40 | N/A | N/A | N/A | No significant differences between groups were found (p>.05). | 12 |
| Abrahms, 1979 | Zung Depression Scale. Greater scores represent greater endorsement of depressive symptoms. | C: 7 | N/A | CBT: 7 | N/A | N/A | N/A | The CBT group had a significantly greater reduction in depression scores as compared to the C group (p<.05) | 10 |
| Hosseinzadeh Asl, 2014 | Beck Depression Inventory-II. Greater scores represent greater endorsement of depressive symptoms. | OAT Only: 17 | 23.65 (8.05) | MBCT: 16 | 16.94 (7.64) | N/A | N/A | No significant differences between groups were found (p>.05). | 8 |
| Oliveto, 2005 | The Center for Epidemiological Studies Depression Scale (CES-D). Greater scores represent greater endorsement of depressive symptoms. | C: 35 | N/A | C + CM: 35 | N/A | N/A | N/A | No significant differences between groups were found (p>.05). | Not reported |
| *Distress* |  |  |  |  |  |  |  |  |  |
| Ball, 2007 | Brief symptom inventory is a self-report measure of psychiatric symptoms that asks participants to rate items on a five point scale of distress. Includes nine primary symptom dimensions, global severity index was analyzed for this study as a measure of change in psychiatric symptom severity. | C + 12-Step Facilitation Therapy: NR | N/A | C + DFST: NR | N/A | N/A | N/A | No significant differences between groups were found (p>.05). | 24 |
| *General Psychological Functioning & Psychiatric Symptoms* | | | | | | |  |  |  |
| Pan, 2015 | Addiction severity index - mental health score. A 55-item scale that assesses seven dimensions of addiction: physical condition, employment status, drug usage, alcohol abuse, criminal activities, family relationships, and mental status. Greater scores represent greater mental health concerns. | OAT Only: 120 | 0.01 (0.1) | C + CBT: 120 | 0.1 (0.1) | N/A | N/A | No significant differences between groups were found (p>.05). | 26 |
| Jiang, 2012 | Addiction Severity Index – Mental Health Score | C: 62 | 0.02 (0.1) | C + CM + MI: 63 | 0.03 (0.2) | N/A | N/A | No significant differences between groups were found (p>.05). | 24 |
| Day, 2018 | Psychological functioning: measured using three domains of the Clinical Outcome in Routine Evaluation (CORE-OM) scale (a) well-being; (b) symptoms; (c) functioning. Greater scores represent greater mental health concerns. Scores represent the **symptoms** subscale. | C: 30 | Median (IQR): 1.6 (0.8, 2.3) | PGS: 27 | Median (IQR): 1.5 (0.8, 2.0) | C + BSBNT + NLM: 26 | 2.0 (1.0, 2.0) | No significant differences between groups were found (p>.05). | 12 |

*Note.* BSBNT = Brief Social Behaviour and Network Therapy, CBT = Cognitive Behavioural Therapy, CRA = Community Reinforcement Approach, CM = Contingency Management, C = Counselling, DFST = Dual Focus Schema Therapy, IPT = Interpersonal Psychotherapy, MBCT = Mindfulness Based Cognitive Therapy, NLM = Node-Link Mapping, NR = Not Reported, OAT = Opioid Agonist Treatment, PGS = Personal Goal Setting, PSEP = Psychoanalytic Supportive-Expressive Psychotherapy
