# Supplementary material for: Evaluating comparative effectiveness of psychosocial interventions adjunctive to opioid agonist therapy for opioid use disorder: A systematic review with network meta-analyses
Source: PLoS One. 2020 Dec 28;15(12):e0244401. doi: 10.1371/journal.pone.0244401 (PMC7769275; doi:10.1371/journal.pone.0244401)
Supplement: S15 Text — (DOCX) [file pone.0244401.s016.docx]

| **S15 Text: Overview of Findings by Study, *Alcohol Use*** | | | |  |  |  |  |  |  |
| --- | --- | --- | --- | --- | --- | --- | --- | --- | --- |
| **Author, Year** | **Outcome Description** | **Control Group:** N | **Control Group:** Mean (SD) | **Intervention Group 1:** N | **Intervention Group 1:** Mean (SD) | **Intervention Group 2:** N | **Intervention Group 2:** Mean (SD) | **Author Reported Conclusions** | **Final Timepoint (Weeks)** |
| *Abstinence from Alcohol* | |  |  |  |  |  |  |  |  |
| Petry, 2002 | Days of self-reported abstinence from alcohol. The timeline follow back was used to report alcohol use. | C: 23 | 26.5 (8.2) | C + CM: 19 | 25.5 (7) | N/A | N/A | No significant differences between groups were found (p>.05). | 24 |
| Preston, 2000 | Percentage of alcohol-negative breath specimens; Testing was conducted using an enzyme multiplied immunoassay technique. | C: 28 | 99.7 (1.1) | C + CM: 29 | 99.7 (1.1) | N/A | N/A | No significant differences between groups were found (p>.05). | 8 |
| *Addiction Severity Index – Alcohol Composite Score* | | |  |  |  |  |  |  |  |
| Woody, 1987 | The alcohol severity index (ASI) is a 55-item scale that assesses seven dimensions of addiction: physical condition, employment status, drug usage, alcohol abuse, criminal activities, family relationships, and mental status. An algorithm is applied to the scores of the 55 items in the scale to compute composite scores (between 0 and 1, with higher scores representing more severe addiction) for each of the seven dimensions. The factor alcohol score was used for this study. | C: 31 | 59 (7) | C + PSEP: 28 | 64 (12) | C + CBT: 34 | 59 (7) | The C+CBT and C+PSEP groups had significantly greater reductions in alcohol use as compared to the C group (p<.05). | 52 |
| Kidorf, 2018 | The ASI composite alcohol score. | C: 69 | N/A | C+CM: 72 | N/A | N/A | N/A | No significant differences between groups were found (p>.05). | 26 |
| Pan, 2015 | The ASI composite alcohol score. | OAT Only: 120 | 0.03 (0.06) | C + CBT: 120 | 0.02 (0.05) | N/A | N/A | No significant differences between groups were found (p>.05). | 26 |
| Jiang, 2012 | The ASI composite alcohol score. | C: 62 | 0.02 (0.08) | C + CM + MI: 63 | 0.02 (0.08) | N/A | N/A | No significant differences between groups were found (p>.05). | 24 |
| Abbott, 1998 | The ASI composite alcohol score. | C: 55 | 0.04 (0.11) | CRA: 96 | 0.04 (0.13) | N/A | N/A | No significant differences between groups were found (p>.05). | 24 |
| Downey, 2000 | The ASI composite alcohol score. | CBT: 21 | N/A | CBT + CM: 20 | N/A | N/A | N/A | No significant differences between groups were found (p>.05). | 17 |
| Schwartz, 2012 | The ASI composite alcohol score. | OAT Only: 104 | 0.05 (0.10) | C + CM: 99 | 0.0 (0.16) | N/A | N/A | No significant differences between groups were found (p>.05). | 16 |
| Groß 2006 | The ASI composite alcohol score. | C: 20 | N/A | C + CM: 20 | N/A | N/A | N/A | No significant differences between groups were found (p>.05). | 12 |
| *Reported Drinking Use* | | | | | | |  |  |  |
| Rounsaville, 1983 | Current drinking pattern (range 1-5, 1 = none, 5 = chronic excessive). | C: 28 | N/A | IPT: 22 | N/A | N/A | N/A | No significant differences between groups were found (p>.05). | 24 |
| *Note.* CBT = Cognitive Behavioural Therapy, CRA = Community Reinforcement Approach, CM = Contingency Management, C = Counselling, IPT = Interpersonal Psychotherapy, MI = Motivational Interviewing, OAT = Opioid Agonist Treatment, PSEP = Psychoanalytic Supportive-Expressive Psychotherapy | | | | | | | | |  |
