# Supplementary material for: Evaluating comparative effectiveness of psychosocial interventions adjunctive to opioid agonist therapy for opioid use disorder: A systematic review with network meta-analyses
Source: PLoS One. 2020 Dec 28;15(12):e0244401. doi: 10.1371/journal.pone.0244401 (PMC7769275; doi:10.1371/journal.pone.0244401)
Supplement: S16 Text — (DOCX) [file pone.0244401.s017.docx]

| **S16 Text: Overview of Findings by Study, *Adherence to OAT*** | | | | | |  |  |  |  |  | |  |
| --- | --- | --- | --- | --- | --- | --- | --- | --- | --- | --- | --- | --- |
| **Author, Year** | **Outcome Description** | **Control Group:** N | **Control Group:** Adherence to OAT N (%) | **Intervention Group 1:** N | **Intervention Group 1:** Adherence to OAT N (%) | **Intervention Group 2:** N | **Intervention Group 2:** Adherence to OAT N (%) | **Intervention Group 3: N** | **Intervention Group 3:** Adherence to OAT N (%) | | **Author Reported Conclusion** | **Final Timepoint (Weeks)** |
| *Continuing to Receive OAT* | | | | | | | | | | | | |
| Ling, 2013 | Percentage of dose that was taken as prescribed. | C: 51 | 45 (88.2%) | C + CBT: 53 | 45 (85.0%) | C + CM: 49 | 42 (85.7%) | CBT + CM: 49 | 42 (85.7%) | No significant differences between groups were found (p>.05). | | 52 |
| Pan, 2015 | Regular attendance in MMT clinics defined as attending MMT clinics to take daily oral methadone under supervision. Participants were permitted to have gaps of no more than consecutive 7 days with appropriate excuses in the study period. | OAT Only: 120 | 67 (55.8%) | C + CBT: 120 | 77 (64.2%) | N/A | N/A | N/A | N/A | No significant differences between groups were found (p>.05). | | 26 |
| Chawarski, 2011 | Continuing to receive methadone treatment at 6 months. | OAT Only: 17 | 13 (76.5%) | EMM: 20 | 16 (80.0%) | N/A | N/A | N/A | N/A | No significant differences between groups were found (p>.05). | | 24 |
| Scherbaum, 2005 | Percentage of patients continuously in MMT in the same setting. | C: 32 | 19 (59.4%) | CBT: 41 | 27 (63%)* | N/A | N/A | N/A | N/A | No significant differences between groups were found (p>.05). | | 24 |

| **Author, Year** | **Outcome Description** | **Control Group:** N | **Control Group:** Mean (SD) | **Intervention Group:** N | **Intervention Group:** Mean (SD) | **Author Reported Conclusions** | **Final Timepoint (Weeks)** |
| --- | --- | --- | --- | --- | --- | --- | --- |
| *Days receiving OAT* | |  |  |  |  |  |  |
| Avants, 1999 | Number of days methadone was received out of 84 possible days. | C: 145 | 81.7 (3.2) | C + CBT: 146 | 81.5 (3.4) | No significant differences between groups were found (p>.05). | 24 |
| Gu, 2013 | Number of days attending the MMT clinics. | OAT Only: 146 | N/A | C: 142 | N/A | The C group attended the MMT clinics significantly more days than the OAT only group (p<.05). | 24 |

| Jiang, 2012 | The number of days over the first 12 weeks of treatment that the subject attended the clinic and received methadone. | C: 80 | 62 | C + CM + MI: 80 | 63 | No significant differences between groups were found (p>.05). | 24 |
| --- | --- | --- | --- | --- | --- | --- | --- |

|  |  |  |  |  |  |  |  |
| --- | --- | --- | --- | --- | --- | --- | --- |
| Fiellin, 2006 | Mean percentage of days which patients adherence to buprenorphine/naloxone. Buprenorphine/naloxone medication adherence was measured by the use of computerized caps placed on medication bottles. | C: 56 | 73 (95% confidence interval: 67-79) | C+EMM: 56 | 69 (95% confidence interval: 63-74) | No significant differences between groups were found (p>.05). | 24 |
| Chen, 2013 | The number of days participants took the required daily dosage of methadone. | OAT Only: 120 | 58 (24.2) | CM: 126 | 65.3 (17.4) | The CM group took the required methadone dose significantly more days than the C group (p<.05). | 12 |
| Moore, 2019 | Number of days methadone was taken based on administrative data. | C: 42 | 93.0 (11.9) | C+CBT: 40 | 86.1 (19.7) | No significant differences between groups were found (p>.05). | 12 |
| Tetrault, 2012 | Percent of days of buprenorphine/naloxone adherence. Buprenorphine/naloxone medication adherence was measured by the use of computerized caps placed on medication bottles. These record the date and time at which each bottle is opened. Patients were classified as adherent to buprenorphine or naloxone for a given day if the cap recorded that the bottle was opened on that day. | C: 25 | 75.3 (19.8) | CM + EMM: 22 | 78.3 (20.6) | No significant differences between groups were found (p>.05). | 12 |
| Shi, 2020 | Mean days of OAT completed. | OAT Only: 10 | 68.6 (32.6) | CBT:10 | 82.6 (4.4) | Not Reported | 12 |

*Note. **The number of participants and percent provided in text do not total as expected. 27 of 41 participants are described as having adhered to opioid agonist treatment and this is noted as 63% of participants in the intervention group, however, 27/41= 65.8% as opposed to 63%. CBT = Cognitive Behavioural Therapy, CM = Contingency Management, C = Counselling, EMM = Enhanced Medical Management, MMT = Methadone Maintenance Therapy, MI = Motivational Interviewing, OAT = Opioid Agonist Treatment
