# Supplementary material for: Evaluating comparative effectiveness of psychosocial interventions adjunctive to opioid agonist therapy for opioid use disorder: A systematic review with network meta-analyses
Source: PLoS One. 2020 Dec 28;15(12):e0244401. doi: 10.1371/journal.pone.0244401 (PMC7769275; doi:10.1371/journal.pone.0244401)
Supplement: S17 Text — (DOCX) [file pone.0244401.s018.docx]

| **S17 Text: Overview of Findings by Study, *Self-Reported Opioid Use*** | | | | | | | | | | | |  | | | | | | |  | |  |  |
| --- | --- | --- | --- | --- | --- | --- | --- | --- | --- | --- | --- | --- | --- | --- | --- | --- | --- | --- | --- | --- | --- | --- |
| **Author, Year** | | **Outcome Description** | | **Control Group:** N | | | | **Control Group:** N (%) | | | | **Intervention Group:** N | | | | **Intervention Group:** N (%) | | | | | **Author Reported Conclusions** | **Final Timepoint (Weeks)** |
| *Percent of Patients Reporting Opioid Use in the Previous Month* | | | | | | | | | | | | | | | |  | | | | |  |  |
| Jaffray, 2014 | | Proportion of patients using illicit heroin in the last 30 days. | | OAT Only: 153 | | | | 48 (31.4%) | | | | MI: 182 | | | | 59 (32.4%) | | | | | No significant differences between groups were found (p>.05). | 26 |
|  | |  | |  | | | |  | | | |  | | | |  | | | | |  |  |
| **Author, Year** | **Outcome Description** | | | **Control Group:** N | | **Control Group:** Mean (SD) | | | | **Intervention Group 1:** N | | | **Intervention Group 1:** Mean (SD) | | **Intervention Group 2:** N | | | **Intervention Group 2:** Mean (SD) | | | **Author Reported Conclusions** | **Final Timepoint (Weeks)** |
| *Reported Opioids Use in the Previous Month* | | | | | | | | | | | | | | | | | | | | | |  |
| Woody, 1987 | Self-reported use (number of days) of heroin in the past 30 days. | | C: 31 | | | 2 (1) | | | | C + PSEP: 28 | | | 3 (2) | | C + CBT: 34 | | | 2 (2) | | | Significant differences were found which favoured the two psychotherapy groups (p<.05). | 52 |
| Catalano, 1999 | Mean times using heroin in the previous month. | | C: 55 | | | 19.7 (36.8) | | | | C + SBPT: 75 | | | 6.9 (15.8) | | N/A | | | N/A | | | No significant differences between groups were found (p>.05). | 52 |
| Hser, 2011 | Self-reported opiate use in past 30 days. | | OAT Only: 159 | | | 3.1 (7.1) | | | | CM: 160 | | | 4.2 (7.5) | | N/A | | | N/A | | | No significant differences between groups were found (p>.05). | 24 |
| Jiang, 2012 | Self-reported use (number of days) of heroin in the past 30 days. | | C: 62 | | | 0.2 (0.1) | | | | C + CM + MI: 63 | | | 0.3 (1.7) | | N/A | | | N/A | | | No significant differences between groups were found (p>.05). | 24 |
| *Reported Opioids Use in the Previous Week* | | | | | | | | | | | |  | | | | | | |  | |  |  |
| Fiellin, 2006 | | Mean self-reported frequency of illicit opioid use per week. | | C: 56 | | | N/A | | C + EMM: 56 | | | | N/A | N/A | | | N/A | | | | No significant differences between groups were found (p>.05). | 24 |
| Fiellin, 2013 | | Mean self-reported frequency of illicit opioid use per week. | | C: 71 | | | N/A | | C + CBT: 70 | | | | N/A | N/A | | | N/A | | | | No significant differences between groups were found (p>.05). | 24 |
| *Reported Opioids Use Per Day* | | | | | | | |  | | | |  | | | | | | |  | |  |  |
| Preston, 2000 | | Mean frequency of opioid use per day. | | C: 28 | 0.39 (0.6) | | | | | | C + CM: 29 | | 0.26 (0.4) | N/A | | | N/A | | | | No significant differences between groups were found (p>.05). | 28 |
| Preston, 2002 | | Mean self-reported frequency of heroin use per day. | | C: 55 | N/A | | | | | | C + CM: 55 | | N/A | N/A | | | N/A | | | | The C+CM group had a significantly greater reduction in frequency of use than the counselling group (p < 0.05). | 12 |
| Moore, 2013 | | Mean frequency of self-reported opioid use per day. | | C: 18 | N/A | | | | | | C + CBT: 18 | | N/A | N/A | | | N/A | | | | No significant differences between groups were found (p>.05). | 4 |
| *Opioid Treatment Index* | | | | |  | | | | | |  | |  | | | | | | | |  |  |
| Amini-Lari, 2017 | | The Persian-validated version of the opioid treatment index was administered. A score of at least 0.14 is needed to diagnose regular opiate use (i.e. at least weekly use). In this study, the OTI was modified to assess demographics and opiate use in the last 30 days. | | OAT Only: 59 | 0.8 (95% Confidence Interval: 0.4-1.3) | | | | | | CBT: 59 | | 0.2 (95% Confidence Interval: 0.1-0.3) | N/A | | | | | N/A | The CBT group had a significantly greater reduction in frequency of use than the OAT only group (p < 0.05). | | 12 |

*Note.* CBT = Cognitive Behavioural Therapy, CM = Contingency Management, C = Counselling, EMM = Enhanced Medical Management, MI = Motivational Interviewing, OAT = Opioid Agonist Treatment, PSEP = Psychoanalytic Supportive-Expressive Psychotherapy, SBPT = Skills Based Parental Training
