# Supplementary material for: Evaluating comparative effectiveness of psychosocial interventions adjunctive to opioid agonist therapy for opioid use disorder: A systematic review with network meta-analyses
Source: PLoS One. 2020 Dec 28;15(12):e0244401. doi: 10.1371/journal.pone.0244401 (PMC7769275; doi:10.1371/journal.pone.0244401)
Supplement: S18 Text — (DOCX) [file pone.0244401.s019.docx]

| **S18 Text: Overview of Findings by Study, *HIV/HCV-Risk Taking Behaviours*** | | | |  |  |  |  |
| --- | --- | --- | --- | --- | --- | --- | --- |
| **Author, Year** | **Outcome Description** | **Control Group:** N | **Control Group:** Mean (SD) | **Intervention Group:** N | **Intervention Group:** Mean (SD) | **Author Reported Conclusions** | **Final Timepoint (Weeks)** |
| ***Drug Risk*** | | | | | | | |
| O'Neill, 1996 | HIV Risk-Taking Behavioural Scale (a subscale of the Opiate Treatment Scale). This scale covers items related to injecting and sexual behaviour in the month prior to the interview. ***Needle Use Risk*** - higher score represent a higher risk. | C: 36 | 4.3 (5.3) | C + CBT: 37 | 1.7 (3.4) | No significant differences between groups were found (p>.05). | 36 |
| Ghitza, 2008 | HIV Risk-taking Behaviour Scale (HRBS) - ***Needle Use Risk*** - higher score represent a higher risk. | C: 40 | N/A | C + CM: 76 | N/A | No significant differences between groups were found (p>.05). | 24 |
|  |  |  |  |  |  |  |  |
| Joe, 1997 | Participants who reported any dirty needle use in the last month and number of needle injections in the past month. | C: 99 | N/A | NLM: 81 | N/A | No significant differences between groups were found (p>.05). | N/A |
| ***Sexual Risk*** |  |  |  |  |  |  |  |
| O'Neill, 1996 | HIV Risk-Taking Behavioural Scale (a subscale of the Opiate Treatment Scale). This scale consists of 11 items covering injecting and sexual behaviour in the month prior to the interview. ***Sexual Risk*** - higher score represent a higher risk. | C: 36 | 4.3 (3.5) | C + CBT: 37 | 4.0 (2.9) | No significant differences between groups were found (p>.05). | 36 |
| Ghitza, 2008 | HIV Risk-taking Behaviour Scale (HRBS) - ***Sexual Risk*** - higher score represent a higher risk. | C: 40 | N/A | C + CM: 76 | N/A | The C+CM group had a significantly greater reduction in risk than the C group (p < 0.05). | 24 |
| ***Sexual & Drug Risk Combined*** | |  |  |  |  |  |  |
| Chawarski, 2011 | The short version of AIDS Risk Inventory, assessing drug-related and sexual risk behaviours associated with HIV transmission. A higher score represents a higher risk. | OAT Only: 17 | N/A | EMM: 20 | N/A | The EMM group had a significantly greater reduction in risk than the OAT only group (p < 0.05) | 24 |
| Abbott, 1998 | The Risk Assessment Battery is self-administered by the patient. It provides an overall score and individual risk subscale scores for injection drug use and sexual behavior. A higher score represents a higher risk. | C: 55 | 0.1 (0.1) | CRA: 96 | 0.1 (0.1) | No significant differences between groups were found (p>.05). | 24 |
| Kelly, 2012 | AIDS Risk Assessment - inquires about sex- and injection- risk behaviours in the past 30 days. Six items represented sex risk, including for example, frequency of sex without a condom, and frequency of sex without a condom: with someone who is not the participant’s usual. A higher score represents a higher risk. | OAT Only: 97 | N/A | C + CM: 119 | N/A | No significant differences between groups were found for sexual or drug risk (p>.05). | 16 |
| Chawarski, 2008 | The AIDS risk inventory, assessing drug-related and sexual risk behaviors associated with HIV transmission. A higher score represents a higher risk. | C: 12 | N/A | C + EMM: 12 | N/A | No significant differences between groups were found (p>.05). | 12 |
|  |  |  |  |  |  |  |  |
|  |  |  |  |  |  |  |  |
| Tetrault, 2012 | The AIDS Risk Inventory (ARI) was used to assess HIV risk behaviors. The ARI is a structured interview that assesses both frequency and recency of both sex-related and drug use-related behaviors associated with HIV transmission. A higher score represents a higher risk. | C: 25 | 67.7 (29.5) | C + EMM: 22 | 63.7 (44.1) | The C+EMM group had a significantly greater reduction in risk than the C group (p < 0.05) | 12 |
| **Author, Year** | **Outcome description** | **Control Group:** N | **Control Group:** Percent of Participants (95% confidence interval) | **Intervention Group:** N | **Intervention Control Group:** Percent of Participants (95% confidence interval) | **Author Reported Conclusions** | **Final Timepoint (Weeks)** |
| Liu, 2018 | Self-reported HIV risk-taking behaviour – percent of participants sharing needles or other equipment. | OAT Only: 63 | 13.2 (9.9-17.9) | C + Ed: 62 | 13.9 (9.3-17.4) | No significant differences between groups were found (p>.05). | 16 |
|  |  |  |  |  |  |  |  |

*Note.* CBT = Cognitive Behavioural Therapy, CRA = Community Reinforcement Approach, C = Counselling, CM = Contingency Management, Ed = Education, EMM = Enhanced Medical Management, NLM = Node-link Mapping, OAT = Opioid Agonist Treatment
