# Supplementary material for: Evaluating comparative effectiveness of psychosocial interventions adjunctive to opioid agonist therapy for opioid use disorder: A systematic review with network meta-analyses
Source: PLoS One. 2020 Dec 28;15(12):e0244401. doi: 10.1371/journal.pone.0244401 (PMC7769275; doi:10.1371/journal.pone.0244401)
Supplement: S19 Text — (DOCX) [file pone.0244401.s020.docx]

| **S19 Text: Overview of Findings by Study, *Withdrawal Symptoms*** | | | | | | | |  |  |  |  |
| --- | --- | --- | --- | --- | --- | --- | --- | --- | --- | --- | --- |
| **Author, Year** | **Outcome Description** | **Control Group:** N | **Control Group:** Mean (SD) | **Intervention Group 1:** N | **Intervention Group 1:** Mean (SD) | **Intervention Group 2:** N | **Intervention Group 2:** Mean (SD) | **Intervention Group 3:** N | **Intervention Group 3:** Mean (SD) | **Author Reported Conclusions** | **Final Timepoint (Weeks)** |
| Epstein, 2009 | Substance-Dependent Severity Scale (Heroin / Cocaine). | C: 31 | N/A | C + CM: 47 | N/A | N/A | N/A | N/A | N/A | No significant differences between groups were found (p>.05). | 20 |
| Ling, 2013 | Clinical Opioid Withdrawal Scale (COWS). Higher scores represent greater withdrawal. | C: 51 | 1.2 (1.2) | C + CBT: 53 | 1.3 (1.1) | C + CM: 49 | 1.0 (1.2) | CBT + CM: 49 | 1.2 (1.2) | No significant differences between groups were found (p>.05). | 16 |
| Day, 2018 | Leeds Dependence Questionnaire. Maximum score of 30. A higher score indicates greater dependence. | C: 30 | Median (IQR): 5 (2,13) | PGS: 27 | Median (IQR): 7 (4, 10) | C + BSBNT + NLM: 26 | Median (IQR): 10 (7, 16) | N/A | N/A | No significant differences between groups were found (p>.05). | 12 |
| Amini-Lari, 2017 | The Persian version of the substance dependent severity scale (SDS). The SDS was modified to assess the severity of opiate dependence in the last 30 days. Higher scores indicate higher severity of opiate dependence. | OAT Only: 59 | 10.6 (2.5) | CBT: 59 | 5.3 (2.6) | N/A | N/A | N/A | N/A | Not reported (no between group differences calculated). | 12 |
| O’Connor, 1998 | Self-reported opioid withdrawal symptoms on a 0 to 3 scale (none, mild, moderate, severe). Higher scores indicate higher withdrawal. | C: 23 | N/A | CBT: 23 | N/A | N/A | N/A | N/A | N/A | No significant differences between groups were found (p>.05). | 12 |
| Oliveto, 2005 | Weekly mean opiate withdrawal symptoms checklist. | C: 35 | N/A | C + CM: 35 | N/A | N/A | N/A | N/A | N/A | No significant differences between groups were found (p>.05). | 6 |
| Kosten, 2003 | Weekly mean opiate withdrawal symptoms checklist. | C + CBT: 40 | N/A | C + CBT + CM: 40 | N/A | N/A | N/A | N/A | N/A | No significant differences between groups were found (p>.05). | 4 |

*Note.* BSBNT = Brief Social Behaviour and Network Therapy, C = Counselling, CBT = Cognitive Behavioural Therapy, CM = Contingency Management, IQR = Interquartile range, NLM = Node-Link Mapping, PGS = Personal Goal Setting, OAT= Opioid Agonist Treatment
