# Supplementary material for: Evaluating comparative effectiveness of psychosocial interventions adjunctive to opioid agonist therapy for opioid use disorder: A systematic review with network meta-analyses
Source: PLoS One. 2020 Dec 28;15(12):e0244401. doi: 10.1371/journal.pone.0244401 (PMC7769275; doi:10.1371/journal.pone.0244401)
Supplement: S20 Text — (DOCX) [file pone.0244401.s021.docx]

| **S20 Text. Overview of Findings by Study, *Adverse Events*** | | | | | |  | |  |  | | |  |
| --- | --- | --- | --- | --- | --- | --- | --- | --- | --- | --- | --- | --- |
| **Author, Year** | **Outcome description** | **Control Group:** N | **Control Group:** Frequency of Adverse Events N | **Intervention Group 1:** N | **Intervention Group 1:** Frequency of Adverse Events N | **Intervention Group 2:** N | **Intervention Group 2:** Frequency of Adverse Events N | **Intervention Group 3:** N | **Intervention Group 3:** Frequency of Adverse Events N | **Author Reported Conclusions** | **Final Timepoint (Weeks)** |  |
| Schwartz, 2012 | Total number of serious adverse events (on an intent-to-treat basis). | OAT Only: 104 | 12 | C + CM: 99 | 6 | N/A | N/A | N/A | N/A | Not Reported | 52 |  |
| Ling, 2013 | Total number of adverse events deemed possibly- or definitely-related to study drug. Number of adverse events that occurred in each group. | C: 51 | 60 | C + CBT: 53 | 74 | C + CM: 49 | 62 | CBT + CM: 49 | 61 | Not Reported | 52 |  |
| Silverman, 2004 | Hospitalizations were considered potential adverse events. Number of hospitalizations. | C: 26 | 7 | C+CM: 26 | 12 | N/A | N/A | N/A | N/A | Not Reported | 26 |  |
| Moore, 2019 | Number of unanticipated negative events. | C: 42 | 5 | C+CBT: 40 | 7 | N/A | N/A | N/A | N/A | Not Reported | 12 |  |

*Note.* CM = Contingency Management, C = Counselling, OAT = Opioid Agonist Treatment
