# Supplementary material for: Evaluating comparative effectiveness of psychosocial interventions adjunctive to opioid agonist therapy for opioid use disorder: A systematic review with network meta-analyses
Source: PLoS One. 2020 Dec 28;15(12):e0244401. doi: 10.1371/journal.pone.0244401 (PMC7769275; doi:10.1371/journal.pone.0244401)
Supplement: S21 Text — (DOCX) [file pone.0244401.s022.docx]

| **S21 Text: Overview of Findings by Study, *Dropouts from Psychotherapy*** | | | | |  |  |  |
| --- | --- | --- | --- | --- | --- | --- | --- |
| **Author, Year** | **Outcome Description** | **Control Group:** N | **Control Group:** Frequency of Dropout N (%) | **Intervention Group:** N | **Intervention Group:** Frequency of Dropout N (%) | **Author Reported Conclusions** | **Final Timepoint (Weeks)** |
| Rounsaville, 1983 | Voluntary withdrawals were those who did not continue to participate in the psychotherapy study but who continued to remain in good standing in the methadone maintenance program, at least up to the point of withdrawing from the study. In the IPT group, voluntary withdrawals most typically did not make or keep any subsequent appointments with the psychotherapist. If the patient missed all sessions after random assignment, they were a voluntary withdrawal at week 1. If they made it to any session but then missed six sessions in a row or informed the therapist that they were terminating, then they were considered a voluntary withdrawal at the point of the last session kept and their clinical rating at that point was included in the endpoint analysis. In the low-contact cell, voluntary withdrawals were those who did not keep any further appointments for clinical ratings. | C: 28 | 8 (28.6%) | IPT: 22 | 19 (86.4%) | The IPT group had significantly greater dropouts from therapy relapses than the C group (p<0.05). | 24 |
| Otto, 2014 | Percent of non-completers based on a failure to complete at least 12 of 15 CBT sessions. | C: 37 | 9 (24.3%) | CBT: 41 | 9 (22.0%) | No significant differences between groups were found (p>.05). | 20 |
| *Note.* CBT = Cognitive Behavioural Therapy, C = Counselling, IPT= Interpersonal Psychotherapy | | | | | | | |
