# Supplementary material for: Evaluating comparative effectiveness of psychosocial interventions adjunctive to opioid agonist therapy for opioid use disorder: A systematic review with network meta-analyses
Source: PLoS One. 2020 Dec 28;15(12):e0244401. doi: 10.1371/journal.pone.0244401 (PMC7769275; doi:10.1371/journal.pone.0244401)
Supplement: S22 Text — (DOCX) [file pone.0244401.s023.docx]

| **S22 Text: Overview of Findings by Study, C*ravings*** | | | | |  |  |  |  |  |  |  |
| --- | --- | --- | --- | --- | --- | --- | --- | --- | --- | --- | --- |
| **Author, Year** | **Outcome Description** | **Control Group:** N | **Control Group:** Mean (SD) | **Intervention Group 1:** N | **Intervention Group 1:** Mean (SD) | **Intervention Group 2:** N | **Intervention Group 2** Mean (SD) | **Intervention Group 3:** N | **Intervention Group 3:** Mean (SD) | **Author Reported Conclusions** | **Final Timepoint (Weeks)** |
| Ling, 2013 | Craving by Visual Analogue Scale higher scores represent more cravings). | C: 51 | 19.3 (18.2) | C + CBT: 53 | 26.6 (25.2) | C + CM: 49 | 19.7 (21.9) | CBT + CM: 49 | 19.9 (21.1) | No significant differences between groups were found (p>.05). | 18 |
| Preston, 2000 | Patients rated how much they had wanted cocaine and heroin during the past week (higher scores represent more cravings). | C: 28 | 1.3 (1.0) | C + CM: 29 | 1.5 (0.9) | N/A | N/A | N/A | N/A | No significant differences between groups were found (p>.05). | 8 |
|  | | | | | | | | | | | |

*Note.* CBT = Cognitive Behavioural Therapy, CM = Contingency Management, C = Counselling
