# Supplementary material for: Evaluating comparative effectiveness of psychosocial interventions adjunctive to opioid agonist therapy for opioid use disorder: A systematic review with network meta-analyses
Source: PLoS One. 2020 Dec 28;15(12):e0244401. doi: 10.1371/journal.pone.0244401 (PMC7769275; doi:10.1371/journal.pone.0244401)
Supplement: S23 Text — (DOCX) [file pone.0244401.s024.docx]

| **S23 Text. Overview of Findings by Study, *Relapse*** | | | | | | |  |
| --- | --- | --- | --- | --- | --- | --- | --- |
| **Author, Year** | **Outcome description** | **Control Group:** N | **Control Group:** Frequency of Individuals that Relapsed N (%) | **Intervention Group:** N | **Intervention Control Group:** Frequency of Individuals that Relapsed N (%) | **Author Reported Conclusions** | **Final Timepoint (Weeks)** |
| Pashaei, 2013 | Relapse criteria was defined as not showing-up for methadone maintenance treatment, confirmation of return to drug use for at least five continuous days and positive urinary morphine test. | OAT Only: 46 | 29 (63.0%) | CBT: 46 | 17 (37.0%) | The CBT group had significantly fewer relapses than the OAT only group (p<0.05). | 28 |
| Yaghubi, 2017 | Relapse criteria was defined as the percent of participants that had any morphine in their urine. | OAT Only: 30 | 11 (36.7%) | MBSR: 30 | 4 (13.3%) | The MBSR group had significantly fewer relapses than the OAT only group (p<0.05). | 8 |
| *Note.* CBT = Cognitive Behavioural Therapy, MBSR = Mindfulness-Based Stress Reduction, OAT = Opioid Agonist Treatment | | | | | | | |
