# Supplementary material for: Evaluating comparative effectiveness of psychosocial interventions adjunctive to opioid agonist therapy for opioid use disorder: A systematic review with network meta-analyses
Source: PLoS One. 2020 Dec 28;15(12):e0244401. doi: 10.1371/journal.pone.0244401 (PMC7769275; doi:10.1371/journal.pone.0244401)
Supplement: S24 Text — (DOCX) [file pone.0244401.s025.docx]

| **S24 Text: Overview of Findings by Study, *Quality of Life*** | | | | |  |  |  |
| --- | --- | --- | --- | --- | --- | --- | --- |
| **Author, Year** | **Outcome Description** | **Control Group:** N | **Control Group:** Mean (SD) | **Intervention Group:** N | **Intervention Group:** Mean (SD) | **Author Reported Conclusions** | **Final Timepoint (Weeks)** |
| Karow, 2010 | The Modular System for Quality of Life (HRQOL), self-rating scale for the assessment of HRQOL, was used. Items are rated from1 = “very bad” to 7 = “very good”. The core module measures six areas of HRQOL: physical health, vitality, psychosocial QOL, affective QOL, material satisfaction, and spare time QOL. Core QOL is measured by an additional item. Higher values indicating higher HRQOL. | C + Ed: 224 | 49.3 (8.6) | MI: 231 | 47.0 (8.6) | Not Reported | 52 |

*Note.* C = Counselling, Ed = Education, MI = Motivational Interviewing.
