# Supplementary material for: Evaluating comparative effectiveness of psychosocial interventions adjunctive to opioid agonist therapy for opioid use disorder: A systematic review with network meta-analyses
Source: PLoS One. 2020 Dec 28;15(12):e0244401. doi: 10.1371/journal.pone.0244401 (PMC7769275; doi:10.1371/journal.pone.0244401)
Supplement: S25 Text — (DOCX) [file pone.0244401.s026.docx]

**S25 Text: Included Studies**

1. Nyamathi AM, Nandy K, Greengold B, et al. Effectiveness of intervention on improvement of drug use among methadone maintained adults. J Addict Dis. 2011;30(1):6-16.
2. Day E, Copello A, Seddon JL, et al. A pilot feasibility randomised controlled trial of an adjunct brief social network intervention in opiate substitution treatment services. BMC Psychiatry. 2018;18(1):8.
3. Pashaei T, Shojaeizadeh D, Foroushani AR, et al. Effectiveness of relapse prevention cognitive-behavioral model in opioid-dependent patients participating in the methadone maintenance treatment in Iran. Iranian journal of public health. 2013;42(8):896.
4. Jiang H, Du J, Wu F, et al. Efficacy of contingency management in improving retention and compliance to methadone maintenance treatment: a random controlled study. Shanghai Arch Psychiatry. 2012;24(1):11-19.
5. Pan S, Jiang H, Du J, et al. Efficacy of cognitive behavioral therapy on opiate use and retention in methadone maintenance treatment in China: a randomised trial. PloS one. 2015;10(6):e0127598.
6. Stein MD, Herman DS, Moitra E, et al. A preliminary randomized controlled trial of a distress tolerance treatment for opioid dependent persons initiating buprenorphine. Drug Alcohol Depend. 2015;147:243-250.
7. Hosseinzadeh Asl N, Barahmand U. Effectiveness of mindfulness-based cognitive therapy for co-morbid depression in drug-dependent males. Arch Psychiatr Nurs. 2014;28(5):314-318.
8. Otto MW, Hearon BA, McHugh RK, et al. A randomized, controlled trial of the efficacy of an interoceptive exposure-based CBT for treatment-refractory outpatients with opioid dependence. J Psychoactive Drugs. 2014;46(5):402-411.
9. Marsch LA, Guarino H, Acosta M, et al. Web-based behavioral treatment for substance use disorders as a partial replacement of standard methadone maintenance treatment. J Subst Abuse Treat. 2014;46(1):43-51.
10. Chen W, Hong Y, Zou X, McLaughlin MM, Xia Y, Ling L. Effectiveness of prize-based contingency management in a methadone maintenance program in China. Drug and alcohol dependence. 2013;133(1):270-274.
11. Ling W, Hillhouse M, Ang A, Jenkins J, Fahey J. Comparison of behavioral treatment conditions in buprenorphine maintenance. Addiction. 2013;108(10):1788-1798.
12. Gu J, Lau JT, Xu H, et al. A randomized controlled trial to evaluate the relative efficacy of the addition of a psycho-social intervention to standard-of-care services in reducing attrition and improving attendance among first-time users of methadone maintenance treatment in China. AIDS and Behavior. 2013;17(6):2002-2010.
13. Moore BA, Fazzino T, Barry DT, et al. The Recovery Line: A pilot trial of automated, telephone-based treatment for continued drug use in methadone maintenance. J Subst Abuse Treat. 2013;45(1):63-69.
14. Fiellin DA, Barry DT, Sullivan LE, et al. A randomized trial of cognitive behavioral therapy in primary care-based buprenorphine. The American journal of medicine. 2013;126(1):74. e11-74. e17.
15. Fiellin DA, Pantalon MV, Chawarski MC, et al. Counseling plus buprenorphine–naloxone maintenance therapy for opioid dependence. New England Journal of Medicine. 2006;355(4):365-374.
16. Tetrault JM, Moore BA, Barry DT, et al. Brief versus extended counseling along with buprenorphine/naloxone for HIV-infected opioid dependent patients. Journal of Substance Abuse Treatment. 2012;43(4):433-439.
17. Tuten M, Svikis DS, Keyser-Marcus L, O'Grady KE, Jones HE. Lessons learned from a randomized trial of fixed and escalating contingency management schedules in opioid-dependent pregnant women. Am J Drug Alcohol Abuse. 2012;38(4):286-292.
18. Miotto K, Hillhouse M, Donovick R, et al. Comparison of buprenorphine treatment for opioid dependence in three settings. Journal of addiction medicine. 2012;6(1):68.
19. Kelly SM, Schwartz RP, O’Grady KE, Gandhi D, Jaffe JH. Impact of Methadone With v. Without Drug Abuse Counseling on HIV Risk: 4-and 12-Month Findings from a Clinical Trial. Journal of addiction medicine. 2012;6(2):145.
20. Schwartz RP, Kelly SM, O'Grady KE, Gandhi D, Jaffe JH. Randomized trial of standard methadone treatment compared to initiating methadone without counseling: 12‐month findings. Addiction. 2012;107(5):943-952.
21. Hser Y-I, Li J, Jiang H, et al. Effects of a randomized contingency management intervention on opiate abstinence and retention in methadone maintenance treatment in China. Addiction. 2011;106(10):1801-1809.
22. Chawarski MC, Mazlan M, Schottenfeld RS. Behavioral drug and HIV risk reduction counseling (BDRC) with abstinence-contingent take-home buprenorphine: A pilot randomized clinical trial. Drug and Alcohol Dependence. 2008;94(1-3):281-284.
23. Chawarski MC, Zhou W, Schottenfeld RS. Behavioral drug and HIV risk reduction counseling (BDRC) in MMT programs in Wuhan, China: a pilot randomized clinical trial. Drug Alcohol Depend. 2011;115(3):237-239.
24. Karow A, Reimer J, Schafer I, Krausz M, Haasen C, Verthein U. Quality of life under maintenance treatment with heroin versus methadone in patients with opioid dependence. Drug Alcohol Depend. 2010;112(3):209-215.
25. Czuchry M, Newbern-McFarland D, Dansereau DF. Visual representation tools for improving addiction treatment outcomes. J Psychoactive Drugs. 2009;41(2):181-187.
26. Chopra MP, Landes RD, Gatchalian KM, et al. Buprenorphine medication versus voucher contingencies in promoting abstinence from opioids and cocaine. Experimental and clinical psychopharmacology. 2009;17(4):226.
27. Epstein DH, Schmittner J, Umbricht A, Schroeder JR, Moolchan ET, Preston KL. Promoting abstinence from cocaine and heroin with a methadone dose increase and a novel contingency. Drug Alcohol Depend. 2009;101(1-2):92-100.
28. Bickel WK, Marsch LA, Buchhalter AR, Badger GJ. Computerized behavior therapy for opioid-dependent outpatients: A randomized controlled trial. Experimental and Clinical Psychopharmacology. 2008;16(2):132-143.
29. Ghitza UE, Epstein DH, Preston KL. Contingency management reduces injection-related HIV risk behaviors in heroin and cocaine using outpatients. Addictive behaviors. 2008;33(4):593-604.
30. Ball SA. Comparing individual therapies for personality disordered opioid dependent patients. Journal of personality disorders. 2007;21(3):305-321.
31. Brooner RK, Kidorf MS, King VL, Stoller KB, Neufeld KJ, Kolodner K. Comparing adaptive stepped care and monetary-based voucher interventions for opioid dependence. Drug Alcohol Depend. 2007;88 Suppl 2:S14-23.
32. Sullivan LE, Barry D, Moore BA, et al. A trial of integrated buprenorphine/naloxone and HIV clinical care. Clinical infectious diseases : an official publication of the Infectious Diseases Society of America. 2006;43 Suppl 4:S184-190.
33. Poling J, Oliveto A, Petry N, et al. Six-month trial of bupropion with contingency management for cocaine dependence in a methadone-maintained population. Archives of general psychiatry. 2006;63(2):219-228.
34. Scherbaum N, Kluwig J, Specka M, et al. Group psychotherapy for opiate addicts in methadone maintenance treatment–a controlled trial. European Addiction Research. 2005;11(4):163-171.
35. Oliveto A, Poling J, Sevarino KA, et al. Efficacy of dose and contingency management procedures in LAAM-maintained cocaine-dependent patients. Drug and alcohol dependence. 2005;79(2):157-165.
36. Schottenfeld RS, Chawarski MC, Pakes JR, Pantalon MV, Carroll KM, Kosten TR. Methadone versus buprenorphine with contingency management or performance feedback for cocaine and opioid dependence. American Journal of Psychiatry. 2005;162(2):340-349.
37. Silverman K, Robles E, Mudric T, Bigelow GE, Stitzer ML. A randomized trial of long-term reinforcement of cocaine abstinence in methadone-maintained patients who inject drugs. Journal of Consulting and Clinical Psychology. 2004;72(5):839.
38. Kosten T, Oliveto A, Feingold A, et al. Desipramine and contingency management for cocaine and opiate dependence in buprenorphine maintained patients. Drug and Alcohol Dependence. 2003;70(3):315-325.
39. Pollack MH, Penava SA, Bolton E, et al. A novel cognitive-behavioral approach for treatment-resistant drug dependence. Journal of substance abuse treatment. 2002;23(4):335-342.
40. Preston KL, Umbricht A, Epstein DH. Abstinence reinforcement maintenance contingency and one-year follow-up. Drug and alcohol dependence. 2002;67(2):125-137.
41. Linehan MM, Dimeff LA, Reynolds SK, et al. Dialectical behavior therapy versus comprehensive validation therapy plus 12-step for the treatment of opioid dependent women meeting criteria for borderline personality disorder. Drug and Alcohol Dependence. 2002;67(1):13-26.
42. Petry NM, Martin B. Low-cost contingency management for treating cocaine-and opioid-abusing methadone patients. Journal of consulting and clinical psychology. 2002;70(2):398.
43. Gross A, Marsch LA, Badger GJ, Bickel WK. A comparison between low-magnitude voucher and buprenorphine medication contingencies in promoting abstinence from opioids and cocaine. Exp Clin Psychopharmacol. 2006;14(2):148-156.
44. Downey KK, Helmus TC, Schuster CR. Treatment of heroin-dependent poly-drug abusers with contingency management and buprenorphine maintenance. Experimental and Clinical Psychopharmacology. 2000;8(2):176-184.
45. Preston KL, Umbricht A, Epstein DH. Methadone dose increase and abstinence reinforcement for treatment of continued heroin use during methadone maintenance. Archives of General Psychiatry. 2000;57(4):395-404.
46. Catalano RF, Gainey RR, Fleming CB, Haggerty KP, Johnson NO. An experimental intervention with families of substance abusers: one-year follow-up of the focus on families project. Addiction. 1999;94(2):241-254.
47. Chutuape MA, Silverman K, Stitzer M. Contingent reinforcement sustains post-detoxification abstinence from multiple drugs: a preliminary study with methadone patients. Drug and Alcohol Dependence. 1999;54(1):69-81.
48. Avants SK, Margolin A, Sindelar JL, et al. Day treatment versus enhanced standard methadone services for opioid-dependent patients: a comparison of clinical efficacy and cost. American Journal of Psychiatry. 1999;156(1):27-33.
49. O’Connor P, Oliveto A, Shi J, et al. A randomized trial of buprenorphine maintenance for heroin dependence in a primary care clinic for substance users versus a methadone clinic. The American journal of medicine. 1998;105(2):100-105.
50. Abbott PJ, Weller SB, Delaney HD, Moore BA. Community reinforcement approach in the treatment of opiate addicts. Am J Drug Alcohol Abuse. 1998;24(1):17-30.
51. Joe GW, Dansereau DF, Pitre U, Simpson DD. Effectiveness of node-link mapping enhanced counseling for opiate addicts: A 12-month posttreatment follow-up. The Journal of nervous and mental disease. 1997;185(5):306-313.
52. Iguchi MY, Belding MA, Morral AR, Lamb RJ, Husband SD. Reinforcing operants other than abstinence in drug abuse treatment: An effective alternative for reducing drug use. Journal of Consulting and Clinical Psychology. 1997;65(3):421.
53. O'Neill K, Baker A, Cooke M, Collins E, Heather N, Wodak1 A. Evaluation of a cognitive‐behavioural intervention for pregnant injecting drug users at risk of HIV infection. Addiction. 1996;91(8):1115-1126.
54. Woody GE, McLellan AT, Luborsky L, O'Brien CP. Twelve-month follow-up of psychotherapy for opiate dependence. The American journal of psychiatry. 1987.
55. Woody GE, McLellan AT, Luborsky L, O'Brien CP. Psychotherapy in community methadone programs: a validation study. The American journal of psychiatry. 1995.
56. McLellan AT, Arndt IO, Metzger DS, Woody GE, O'Brien CP. The effects of psychosocial services in substance abuse treatment. Jama. 1993;269(15):1953-1959.
57. Rounsaville BJ, Glazer W, Wilber CH, Weissman MM, Kleber HD. Short-term interpersonal psychotherapy in methadone-maintained opiate addicts. Archives of general psychiatry. 1983;40(6):629-636.
58. Amini-Lari M, Alammehrjerdi Z, Ameli F, et al. Cognitive-Behavioral Therapy for Opiate Users in Methadone Treatment: A Multicenter Randomized Controlled Trial. Iranian Journal of Psychiatry and Behavioral Sciences. 2017;11(2).
59. Abrahms JL. A cognitive-behavioral versus nondirective group treatment program for opioid-addicted persons: an adjunct to methadone maintenance. The International journal of the addictions. 1979;14(4):503-511.
60. Carroll KM, Chang G, Behr H, Clinton B, Kosten TR. Improving treatment outcome in pregnant, methadone-maintained women: results from a randomized clinical trial. American Journal on Addictions. 1995;4(1):56-59.
61. Fals-Stewart W, O'Farrell TJ, Birchler GR. Behavioral couples therapy for male methadone maintenance patients: Effects on drug-using behavior and relationship adjustment. Behavior Therapy. 2001;32(2):391-411.
62. Milby JB, Garrett C, English C, Fritschi O, Clarke C. Take-home methadone: contingency effects on drug-seeking and productivity of narcotic addicts. Addictive behaviors. 1978;3(3-4):215-220.
63. Rowan-Szal GA, Joe GW, Hiller ML, Simpson DD. Increasing early engagement in methadone treatment. Journal of Maintenance in the Addictions. 1997;1(1):49-61.
64. Moore BA, Buono FD, Lloyd DP, Printz DM, Fiellin DA, Barry DT. A randomized clinical trial of the Recovery Line among methadone treatment patients with ongoing illicit drug use. Journal of substance abuse treatment. 2019;97:68-74.
65. Barry DT, Beitel M, Cutter CJ, et al. An evaluation of the feasibility, acceptability, and preliminary efficacy of cognitive-behavioral therapy for opioid use disorder and chronic pain. Drug and alcohol dependence. 2019;194:460-467.
66. Kidorf M, Brooner RK, Leoutsakos J-M, Peirce J. Treatment initiation strategies for syringe exchange referrals to methadone maintenance: A randomized clinical trial. Drug and alcohol dependence. 2018;187:343-350.
67. Liu P, Song R, Zhang Y, et al. Educational and behavioral counseling in a methadone maintenance treatment program in China: a randomized controlled trial. Frontiers in psychiatry. 2018;9:113.
68. Yaghubi M, Zargar F, Akbari H. Comparing effectiveness of mindfulness-based relapse prevention with treatment as usual on impulsivity and relapse for methadone-treated patients: a randomized clinical trial. Addiction & health. 2017;9(3):156.
69. Salehi L, Alizadeh L. Efficacy of a cognitive-behavioral relapse prevention model in the treatment of opioid dependence in Iran: A randomized clinical trial. Shiraz E-Medical Journal. 2018;19(5).
70. Christensen DR, Landes RD, Jackson L, et al. Adding an Internet-delivered treatment to an efficacious treatment package for opioid dependence. J Consult Clin Psychol. 2014;82(6):964-972.
71. Jaffray M, Matheson C, Bond CM, et al. Does training in motivational interviewing for community pharmacists improve outcomes for methadone patients? A cluster randomised controlled trial. Int J Pharm Pract. 2014;22(1):4-12.

| 1. Shi JM, Henry SP, Dwy SL, Orazietti SA, Carroll KM. Randomized pilot trial of Web-based cognitive-behavioral therapy adapted for use in office-based buprenorphine maintenance. Substance abuse. 2019 Apr 3;40(2):132-5. |
| --- |
