# Supplementary material for: Evaluating comparative effectiveness of psychosocial interventions adjunctive to opioid agonist therapy for opioid use disorder: A systematic review with network meta-analyses
Source: PLoS One. 2020 Dec 28;15(12):e0244401. doi: 10.1371/journal.pone.0244401 (PMC7769275; doi:10.1371/journal.pone.0244401)
Supplement: S26 Text — (DOCX) [file pone.0244401.s027.docx]

**S26 Text: Excluded Studies From Full Text Screening (With Reasons For Exclusion)**

**Not Published in English or French (n=10)**

1. Gu, J., Zhao, Y., Zhong, Y., Xu, C. j., Pan, W. l., Fan, L. r., … & Wang, M. (2012). [Effectiveness of intervention services provided by social workers in methadone maintenance treatment clinics]. *Zhonghua yu fang yi xue za zhi [Chinese journal of preventive medicine].* 46(11), 999-1003.
2. Heidari, Z., Taremian, F., & Khalatbari, J. (2017). The effect of modified alpha-theta neurofeedback protocol on instant craving in opioid users*. J Adv Med Biomed Res*, 25(109), 130-139.
3. Hemmat, A., Bytamar, J.M., Pirzeh, R., Dadashi, M. The effectiveness of group therapy based on acceptance and commitment to reduce obsessive-compulsive use of substance, anxiety and depression of addicts under methadone treatment. *Journal of Zanjan University of Medical Sciences and Health Services*. 2018/// 26(117), 109.
4. Hemmat, A., Dadashi, M., Momtazi, S., Zenoozian, S., Bytamar, J.M. The Effectiveness of Group Therapy Based on Acceptance and Commitment to Reduce Craving and Improve the Quality of Life of Addicts Undergoing Methadone Treatment . *J Adv Med Biomed Res*. 2018; 26 (116) :61-74
5. Krupitsky, E. M., Zvartau, E. E., Blokhina, E. A., Verbitskaya, E. V., Wahlgren, V. Y., Tsoy-Podosenin, M. V., ... & Tiurina, A. A. (2018). Anhedonia, depression, anxiety, and craving in opioid dependent patients stabilized on oral naltrexone or naltrexone implant. *Zhurnal nevrologii i psikhiatrii imeni SS Korsakova,* 118(1. Vyp. 2), 26-33.
6. Kuhn, S., Schu, M., Vogt, I., Schmid, M., Simmedinger, R., Schlanstedt, G., … & Haasen, C. (2007). Psychosocial care in the German model project on heroin-maintenance therapy for opiate dependence*. Sucht*. 53(5), 278-287.
7. Radfar, M., Ghavami, H., Namazpoor, J., & Khalkhali, H. R. (2019). Lapse and craving prevention in methadone maintenance treatment, applying continuous care model: a randomized clinical trial. *Anadolu Psikiyatri Dergisi*. *20*(2), 117-124.
8. Sheidow, A. J., & Henggeler, S. W. (2008). [Multisystemic therapy with substance using adolescents: a clinical and research overview]. *Praxis der Kinderpsychologie und Kinderpsychiatrie.* 57(5), 401-419.
9. Wang, J., Jiang, H.-F., Du, J., Dong, A.-Z., Wang, J.-G., Fa, J.-M., … & Sun, H.-M. (2015). Randomized control trial on improving effect of methadone maintenance treatment by enhancing motivation. *Journal of Shanghai Jiaotong University (Medical Science).* 35(10), 1497-1501.
10. Wang, N.-S., Li, R.-Q., Hou, F., & Ju, F. (2004). Effects of psychological rehabilitation therapy on self-esteem and defense style in heroin addicts. *Chinese Journal of Clinical Rehabilitation.* 8(30), 6598-6599.

**Conference Abstract Only (n=19)**

1. Arechiga, G., Bonilla, J., Mendoza, I., Correa, J., and Ruiz, Y. (2019). Naltrexone implants improve therapeutic adherence with multiple substance use disorder. 50th Annual Conference of the American Society of Addiction Medicine, ASAM 2019. United States, 3(13) 13.
2. Bohnert ASB, Walton M. (2017). Acceptability And Feasibility Of An Intervention For Overdose And HIV Risk During Addictions Treatment For Patients With Prescription Opioid Misuse. Drug And Alcohol Dependence. 0376-8716. (171). 22.
3. Esmaeili, A., Yaghoubi, R., and Dastgerdi, R. (2018). Evaluate the effectiveness of cognitive behavioral group psychotherapy on psychological well-being of under treatment addict patient. Annals of Tropical Medicine and Public Health. 1755-6783. 65.
4. Fanucchi, L., Walsh, S. L., and Lofwall, M. R. (2018). Are long hospital stays needed for opioid use disorder and injection-related infections?. Journal of addiction medicine. 3 (12).2.
5. Gandhi, J. S. (2019). In Person v/s computerized counselling with a multilingual interactive software in SUD. 71st Annual Conference of Indian Psychiatric Society, ANCIPS 2019. India. 9 Supplement 3 (61). 488.
6. Hammond, C. J., Fishman, M., Hendrickson, B., Wenzel, K., Horner, M., Rahman, N., Allick, A., Rathod, K., Findling, R., and Matson, P. A.. (2019). 50th Annual Conference of the American Society of Addiction Medicine, ASAM 2019. United States, 13(3). 24
7. Jaffray, M. (2011). A Cluster Randomised Controlled Trial Of Enhanced Pharmacy Services (EPS) To Improve Outcomes For Patients On Methadone Maintenance therapy (MMT). International Journal of Pharmacy Practice. 0961-7671 (19) 4.
8. Jaiswal, R. (2018). Evaluating Motivational Enhancement Therapy Versus Life Skill Training Along With Pharmacotherapy In Management Of Opioid Use Disorder. Indian Journal Of Psychiatry. 5. (60). 155.
9. Jalali, A., Rahmati, M., Salari, N., and Behrouzi, M. K. (2018). Group Spiritual Intervention And Happiness Of Help-Seekers Receiving Methadone Maintenance Treatment. Annals Of Tropical Medicine And Public Health. 1755-6783 (12). S726.
10. Kim SJ, Marsch LA. (2015). Can Persons With A History Of Multiple Addiction Treatment Episodes And Chronic Relapse Benefit From Technology-Delivered Behavior Therapy?. Drug And Alcohol Dependence. 0376-8716. (156). 111.
11. Ling, W., Nadipelli, V. R., Solem, C. T., Ronquest, N. A., Yeh, Y. C., Learned, S. M., ... & Heidbreder, C. (2020). Effects Of Monthly Buprenorphine Extended-Release Injections On Patient-Centered Outcomes: A Long-Term Study. Journal of Substance Abuse Treatment, 110, 1-8.
12. Litwin AH, Agyemang L. (2017). The PREVAIL Study: Intensive Models Of HCV Care For People Who Inject Drugs. Journal Of Hepatology. 1 (66). 72.
13. Martin, C. E., Terplan, M., O'Grady, K., and Jones, H. E. (2017). Pregnancy Intention And Opioid Use Disorder Treatment Retention In The Mother Study. Journal Of Addiction Medicine. 3 (11).12.
14. Muench, S. C., Akiyama, M. J., Heo, M., and Litwin, A. H. (2018). Effect Of Direct Acting Antivirals On Health-Related Quality Of Life In Patients Who Inject Drugs. Gastroenterology. 6 (154).
15. Shrestha, R., & Copenhaver, M. M. (2018). Viral Suppression Among HIV-Infected Methadone-Maintained Patients: The Role Of Ongoing Injection Drug Use And Adherence To Antiretroviral Therapy (ART). *Addictive Behaviors*, *85*, 88-93.
16. Solomon SS, McFall AM Srikrishnan AK Lucas GM Anand S Kumar MS Balakrishnan. (2018). Integrating HCV And HIV Services Improves HCV Testing And Awareness Among People Who Inject Drugs In India: Findings From A Cluster Randomized Trial. Journal Of Viral Hepatitis. 1352-0504 (25) 118.
17. Wenzel, K. R., Burgower, R., Wildberger, J., Fishman, M., and Vo, H. (2019). Youth Opioid Recovery Support Intervention: Home Delivery Of Extended Release Naltrexone. 50th Annual Conference Of The American Society Of Addiction Medicine, ASAM 2019. United States. 3(13). 42.
18. Wildberger, J. (2019). Relationship Between Injectable Naltrexone And IOP Utilization On Opioid Relapse In Youth. Journal Of Addiction Medicine. 3 (13). 36.
19. Wilson, M. (2017). Receptivity To An Online Pain Self-Management Program Among People With Persistent Pain In Opioid Addiction Treatment. Journal of Pain. 4 (18). 37.

**Not an RCT (n=129)**

1. 28th Annual Meeting and Symposium of the American Academy of Addiction Psychiatry, AAAP 2018. American Journal on Addictions. Conference:(4).
2. Ainscough, T. S., Brose, L. S., Strang, J., & McNeill, A. (2017). Contingency management for tobacco smoking during opioid addiction treatment: a randomised pilot study. *BMJ open.* 7(9), e017467.
3. Amass, L., Ling, W., Freese, T. E., Reiber, C., Annon, J. J., Cohen, A. J., … & Horton, T. (2004). Bringing buprenorphine-naloxone detoxification to community treatment providers: the NIDA Clinical Trials Network field experience. *The American journal on addictions.* 13 Suppl 1, S42-S66.
4. Arani, F. D., Rostami, R., & Nostratabadi, M. (2010). Effectiveness of neurofeedback training as a treatment for opioid-dependent patients. *Clinical EEG and neuroscience.* 41(3), 170-177.
5. Baldus, C., Mokros, L., Daubmann, A., Arnaud, N., Holtmann, M., Thomasius, R., & Legenbauer, T. (2018). Treatment effectiveness of a mindfulness-based inpatient group psychotherapy in adolescent substance use disorder-study protocol for a randomized controlled trial. *Trials*, *19*(1), 706.
6. Bandstra, E. S. (2012). Maternal Opioid Treatment: Human Experimental Research (MOTHER) Study: Maternal, Fetal And Neonatal Outcomes From Secondary Analyses. *Addiction*. (107).1.
7. Bazazi, A. R., Wickersham, J. A., Wegman, M. P., Culbert, G. J., Pillai, V., Shrestha, R., … & Altice, F. L. (2017). Design and implementation of a factorial randomized controlled trial of methadone maintenance therapy and an evidence-based behavioral intervention for incarcerated people living with HIV and opioid dependence in Malaysia. *Contemporary clinical trials.* 59, 1-12.
8. Boden, M. T., Kimerling, R., Kulkarni, M., Bonn-Miller, M. O., Weaver, C., & Trafton, J. (2014). Coping among military veterans with PTSD in substance use disorder treatment. *Journal of substance abuse treatment*, *47*(2), 160-167.
9. Businelle, M. S., Parker, J. D., May, R. K., Kendzor, D. E., & Burke, R. S. (2008). Effects of contingency management on substance abuse continuing care participation. *Addictive Disorders and their Treatment.* 7(2), 99-107.
10. Callahan, E., Rawson, R., Glazer, M., McCleave, B., & Arias, R. (1976). Comparison of two naltrexone treatment programs: naltrexone alone versus naltrexone plus behavior therapy. *NIDA research monograph.* 150-157.
11. Chandra, D. K., Bazazi, A. R., Nahaboo Solim, M. A., Kamarulzaman, A., Altice, F. L., & Culbert, G. J. (2019). Retention in clinical trials after prison release: results from a clinical trial with incarcerated men with HIV and opioid dependence in Malaysia. *HIV research & clinical practice*. 20(1), 12-23.
12. Comer, S. D., Sullivan, M. A., & Walker, E. A. (2005). Comparison of intravenous buprenorphine and methadone self-administration by recently detoxified heroin-dependent individuals. *The Journal of pharmacology and experimental therapeutics*. 315(3), 1320-1330.
13. Cooperman, N. A., Heo, M., Berg, K. M., Li, X., Litwin, A. H., Nahvi, S., & Arnsten, J. H. (2012). Impact of adherence counseling dose on antiretroviral adherence and HIV viral load among HIV-infected methadone maintained drug users. *AIDS care*. 24(7), 828-835.
14. Craine, N., Hickman, M., Parry, J. V., Smith, J., Walker, A. M., Russell, D., ... & Lyons, M. (2009). Incidence of hepatitis C in drug injectors: the role of homelessness, opiate substitution treatment, equipment sharing, and community size. *Epidemiology & Infection*, 137(9), 1255-1265.
15. Crits-Christoph, P., Lundy, C., Stringer, M., Gallop, R., & Gastfriend, D. R. (2015). Extended-Release Naltrexone for Alcohol and Opioid Problems in Missouri Parolees and Probationers. *Journal of substance abuse treatment.* 56, 54-60.
16. Crump, M. T. & Milling, R. N. (1996). The efficacy of substance abuse education among dual-diagnosis patients. *Journal of substance abuse treatment*. 13(2), 141-144.
17. Cruz, R., Sanusi, M. T., Wahbi, R., Cruz, A., Clark, M., Hostetler, D., Higgins-Biddle, M., and Alford, D. P. (2019). Addressing Low Receipt Of Lifesaving Medications For Opioid Use Disorders In Minority Populations: Is Engagement In Primary Care Through Peer Recovery Coaching The Answer?. *Journal of General Internal Medicine*. 2 (34). 700.
18. de Jong, C. A. J., Roozen, H. G., Van Rossum, L. G. M., Krabbe, P. F. M., & Kerkhof, A. J. F. M. (2007). High abstinence rates in heroin addicts by a new comprehensive treatment approach. *American Journal on Addictions.* 16(2), 124-130.
19. Desrosiers, A., Blokhina, E., Krupitsky, E., Zvartau, E., Schottenfeld, R., & Chawarski, M. (2017). Psychiatric symptoms, quality of life, and HIV status among people using opioids in Saint Petersburg, Russia. *Drug and alcohol dependence*. 172, 60-65.
20. Dimeff, L. A. & Linehan, M. M. (2008). Dialectical behavior therapy for substance abusers. *Addiction science & clinical practice*. 4(2), 39-47.
21. Ducharme, L. J., Knudsen, H. K., & Roman, P. M. (2006). Evidence-based treatment for opiate-dependent clients: availability, variation, and organizational correlates. *The American journal of drug and alcohol abuse.* 32(4), 569-576.
22. Ducharme, L. J., Knudsen, H. K., Roman, P. M., & Johnson, J. A. (2007). Innovation adoption in substance abuse treatment: exposure, trialability, and the Clinical Trials Network*. Journal of substance abuse treatment.* 32(4), 321-329.
23. ED Management : The Monthly Update on Emergency Department Management. (2016). ED-based Counseling Sessions Reduce Risky Opioid Use Among Certain Patients*. ED management : the monthly update on emergency department management*. 28(7), 81-83.
24. El Hage, C., Ghabrash, M. F., Dubreucq, S., Brissette, S., Lespérance, F., Lespérance, P., ... & Jutras-Aswad, D. (2018). A pilot, open-label, 8-week study evaluating desvenlafaxine for treatment of major depression in methadone-maintained individuals with opioid use disorder. *International clinical psychopharmacology*, *33*(5), 268-273.
25. Elahei Roudposhti, N., Jalali, A., Khaledi Paveh, B., & Salari, N. (2018). Cognitive-behavioral counseling and self-perception of male clients receiving methadone maintenance treatment. *Alcoholism Treatment Quarterly*, 36(3), 419-427.
26. Ergelen, M., Yalçın, M., & Bilici, R. (2018). The comparison of violence, and the relationship with childhood trauma in Turkish men with alcohol, opiate, and synthetic cannabinoid use disorder. *Neuropsychiatric disease and treatment*, *14*, 3169.
27. Etemadi-Chardah, N., Matinpour, B., & Heshmati, R. (2017). Effectiveness of Transactional Analysis Group Therapy on Addiction Intensity of Woman Patients Treated with Methadone. *Addiction & health*, *9*(3), 146.
28. Evenson, R. C., Binner, P. R., Cho, D. W., Schicht, W. W., & Topolski, J. M. (1998). An outcome study of Missouri's CSTAR alcohol and drug abuse programs. *Journal of substance abuse treatment.* 15(2), 143-150.
29. Fahmy, R., Wasfi, M., Mamdouh, R., Moussa, K., Wahba, A., Wittemann, M., ... & Wolf, R. C. (2018). Mindfulness-based interventions modulate structural network strength in patients with opioid dependence. *Addictive behaviors*, *82*, 50-56.
30. Farabee, D., Hillhouse, M., Condon, T., McCrady, B., McCollister, K., & Ling, W. (2016). Injectable pharmacotherapy for opioid use disorders (IPOD). *Contemporary clinical trials.* 49, 70-77.
31. Finch, A. J., Tanner-Smith, E., Hennessy, E., & Moberg, D. P. (2018). Recovery high schools: Effect of schools supporting recovery from substance use disorders. *American Journal of Drug and Alcohol Abuse.* 44(2), 175-184.
32. Ford, J.H, Osborne, E.L., Assefa, M.T., McIlvaine, A.M., King, A.M., Campbell, K., McGovern, M.P. (2018). Using NIATx strategies to implement integrated services in routine care: a study protocol. *BMC health services research*. 18(1), 431.
33. Gerra, G., Saenz, E., Busse, A., Maremmani, I., Ciccocioppo, R., Zaimovic, A., … & Somaini, L. (2011). Supervised daily consumption, contingent take-home incentive and non-contingent take-home in methadone maintenance. *Progress in Neuro-Psychopharmacology and Biological Psychiatry.* 35(2), 483-489.
34. Gonzalez, A. M., Romero, R. J., Ojeda-Vaz, M. M., & Rabaza, J. R. (2015). Intravenous acetaminophen in bariatric surgery: effects on opioid requirements. *The Journal of surgical research.* 195(1), 99-104.
35. Graham, K., Annis, H. M., Brett, P. J., & Venesoen, P. (1996). A controlled field trial of group versus individual cognitive-behavioural training for relapse prevention. *Addiction.* 91(8), 1127-1139.
36. Greenwald, M. K. (2008). Opioid abstinence reinforcement delays heroin lapse during buprenorphine dose tapering. *Journal of applied behavior analysis.* 41(4), 603-607.
37. Greenwald, M. K. (2010). Effects of experimental Unemployment, Employment and Punishment analogs on opioid seeking and consumption in heroin-dependent volunteers. *Drug and alcohol dependence.* 111(1-2), 64-73.
38. Griffith, J. D., Rowan-Szal, G. A., Roark, R. R., & Simpson, D. D. (2000). Contingency management in outpatient methadone treatment: a meta-analysis (DARE structured abstract). *Drug and alcohol dependence.* 58, 55-66.
39. Grow, J. C., Collins, S. E., Harrop, E. N., & Marlatt, G. A. (2015). Enactment of home practice following mindfulness-based relapse prevention and its association with substance-use outcomes*. Addictive behaviors*. 40, 16-20.
40. Guydish, J., Campbell, B. K., Manuel, J. K., Delucchi, K. L., Le, T., Peavy, K. M., & McCarty, D. (2014). Does treatment fidelity predict client outcomes in 12-Step Facilitation for stimulant abuse? *Drug and alcohol dependence*. 134(1), 330-336.
41. Haasen, C., Schulte, B., Vanderplasschen, W., Verthein, U., Schafer, I., & Reimer, J. (2009). Predictive value of regular cocaine use among opioid-dependent patients for long-term outcome: A 4-year follow-up study. *Addictive Disorders and their Treatment*. 8(2), 74-79.
42. Hagedorn, H. J., Stetler, C. B., Bangerter, A., Noorbaloochi, S., Stitzer, M. L., & Kivlahan, D. (2014). An implementation-focused process evaluation of an incentive intervention effectiveness trial in substance use disorders clinics at two Veterans Health Administration medical centers. *Addiction science & clinical practice*. 9, 12.
43. Hassamal, S., Haglund, M., Wittnebel, K., & Danovitch, I. (2016). A preoperative interdisciplinary biopsychosocial opioid reduction program in patients on chronic opioid analgesia prior to spine surgery: A preliminary report and case series. *Scandinavian journal of pain*. 13, 27-31.
44. Hayes, S. C., Wilson, K. G., Gifford, E. V., Bissett, R., Piasecki, M., Batten, S. V., … & Gregg, J. (2004). A preliminary trial of twelve-step facilitation and acceptance and commitment therapy with polysubstance-abusing methadone-maintained opiate addicts. *Behavior Therapy.* 35(4), 667-688.
45. Hensold, T. C., Guercio, J. M., Grubbs, E. E., Upton, J. C., & Faw, G. (2006). A personal intervention substance abuse treatment approach: Substance abuse treatment in a least restrictive residential model. *Brain injury.* 20(4), 369-381.
46. Hill, K. P., Bennett, H. E., Griffin, M. L., Connery, H. S., Fitzmaurice, G. M., Subramaniam, … & Weiss, R. D. (2013). Association of cannabis use with opioid outcomes among opioid-dependent youth. *Drug and alcohol dependence.* 132(1-2), 342-345.
47. Hollonds, G. B., Oei, T. P., & Turecek, L. R. (1980). An evaluation of a behaviour therapy programme as an intervention treatment for the fear of withdrawal with heroin-dependent persons. *Drug and alcohol dependence.* 5(2), 153-160.
48. Holtyn, A. F., Koffarnus, M. N., DeFulio, A., Sigurdsson, S. O., Strain, E. C., Schwartz, R. P., & Silverman, K. (2014). Employment-based abstinence reinforcement promotes opiate and cocaine abstinence in out-of-treatment injection drug users. *Journal of applied behavior analysis.* 47(4), 681-693.
49. Huber, D. L., Sarrazin, M. V., Vaughn, T., & Hall, J. A. (2003). Evaluating the impact of case management dosage. *Nursing research.* 52(5), 276-288.
50. Hruschak, V., Cochran, G., & Wasan, A. D. (2018). Psychosocial interventions for chronic pain and comorbid prescription opioid use disorders: A narrative review of the literature. *Journal of opioid management*, *14*(5), 345-358.
51. Imani, S., Atef V., Mohammad K., Gharraee, B., Habibi, M., Bowen, S., & Noroozi, A. (2015). Comparing Mindfulness-Based Group Therapy With Treatment as Usual for Opioid Dependents: A Pilot Randomized Clinical Trial Study Protocol. *Iranian journal of psychiatry and behavioral sciences.* 9(1), e216.
52. Imani, S., Atef Vahid, M. K., Gharraee, B., Noroozi, A., Habibi, M., & Bowen, S. (2015). Effectiveness of Mindfulness-Based Group Therapy Compared to the Usual Opioid Dependence Treatment. *Iranian journal of psychiatry.* 10(3), 175-184.
53. Jalali, A., Behrouzi, M. K., Salari, N., Bazrafshan, M. R., & Rahmati, M. (2019). The effectiveness of group spiritual intervention on self-esteem and happiness among men undergoing methadone maintenance treatment. *Current Drug Research Reviews Formerly: Current Drug Abuse Reviews*, *11*(1), 67-72.
54. Jalali, A., Yekzaman, M., Bazrafshan, M. R., Salari, N., & Rahmati, M. (2018). Investigating the effect of family counseling on the acceptance and support of patients under methadone maintenance treatment. *Shiraz E Medical Journal*. 19(5).
55. Jarvis, B. P., Holtyn, A. F., Berry, M. S., Subramaniam, S., Umbricht, A., Fingerhood, M., ... & Silverman, K. (2018). Predictors of induction onto extended-release naltrexone among unemployed heroin-dependent adults. Journal of substance abuse treatment, 85, 38-44.
56. Jeal, N., Patel, R., Redmond, N. M., Kesten, J. M., Ramsden, S., Macleod, J., ... & Horwood, J. (2018). Drug use in street sex workers (DUSSK) study protocol: a feasibility and acceptability study of a complex intervention to reduce illicit drug use in drug-dependent female street sex workers. *BMJ open*, *8*(11), e022728.
57. Keegan, J., Lavenduski, C., & Schooff, K. (1976). Comments and findings from a naltrexone double blind study. *NIDA research monograph.* 74-76.
58. Kelly, S. M., O'Grady, K. E., Jaffe, J. H., Gandhi, D., & Schwartz, R. P. (2013). Improvements in outcomes in methadone patients on probation/parole regardless of counseling early in treatment. *Journal of addiction medicine.* 7(2), 133-138.
59. Khodabandeh, F., Kahani, S., Shadnia, S., & Abdollahi, M. (2012). Comparison of the efficacy of methadone maintenance therapy vs. narcotics anonymous in the treatment of opioid addiction: A 2-year survey. *International Journal of Pharmacology.* 8(5), 445-449.
60. Kidorf, M., Stitzer, M. L., Brooner, R. K., & Goldberg, J. (1994). Contingent methadone take-home doses reinforce adjunct therapy attendance of methadone maintenance patients. *Drug and alcohol dependence.* 36(3), 221-226.
61. Kidorf, M., King, V. L., Peirce, J., Gandotra, N., Ghazarian, S., & Brooner, R. K. (2015). Substance use and response to psychiatric treatment in methadone-treated outpatients with comorbid psychiatric disorder. *Journal of substance abuse treatment.* 51, 64-69.
62. Kiluk, B. D., Nich, C., & Carroll, K. M. (2010). Neurocognitive indicators predict results of an informed-consent quiz among substance-dependent treatment seekers entering a randomized clinical trial. *Journal of studies on alcohol and drugs.* 71(5), 704-712.
63. Kosten, T., Poling, J., & Oliveto, A. (2003). Effects of reducing contingency management values on heroin and cocaine use for buprenorphine‐and desipramine‐treated patients. *Addiction*, *98*(5), 665-671.
64. Lasser, K. E., Shanahan, C., Parker, V., Beers, D., Xuan, Z., Heymann, O., … & Liebschutz, J. M. (2016). A Multicomponent Intervention to Improve Primary Care Provider Adherence to Chronic Opioid Therapy Guidelines and Reduce Opioid Misuse: A Cluster Randomized Controlled Trial Protocol. *Journal of substance abuse treatment.* 60, 101-109.
65. Lerner, A., Sigal, M., Bacalu, A., & Gelkopf, M. (1992). Short term versus long term psychotherapy in opioid dependence: a pilot study. *The Israel journal of psychiatry and related sciences.* 29(2), 114-119.
66. Li, L., Comulada, W. S., Lin, C., Hsieh, J., Luo, S., & Wu, Z. (2017). Factors related to client satisfaction with methadone maintenance treatment in China. *Journal of substance abuse treatment.* 77, 201-206.
67. Li, L., Wu, Z., Liang, L. J., Lin, C., Zhang, L., Guo, S., … & Li, J. (2013). An intervention targeting service providers and clients for methadone maintenance treatment in China: a cluster-randomized trial. *Addiction.* 108(2), 356-366.
68. Luijks, M. J. A., Bevaart, F., Zijlmans, J., Van Duin, L., Marhe, R., Doreleijers, T. A., ... & Popma, A. (2017). A multimodal day treatment program for multi-problem young adults: study protocol for a randomized controlled trial. *Trials*, *18*(1), 225.
69. Lussier, J. P., Heil, S. H., Mongeon, J. A., Badger, G. J., & Higgins, S. T. (2006). A meta-analysis of voucher-based reinforcement therapy for substance use disorders (Provisional abstract). *Addiction*. 101, 192-203.
70. Marsden, J., Stillwell, G., Hellier, J., Brown, A. M., Byford, S., Kelleher, M., … & Mitcheson, L. (2017). Effectiveness of adjunctive, personalised psychosocial intervention for non-response to opioid agonist treatment: Study protocol for a pragmatic randomised controlled trial. *Contemporary clinical trials.* 53, 36-43.
71. Maumus-Robert, S., Micallef, J., Mansiaux, Y., Mallaret, M., Lapeyre-Mestre, M., Authier, N., & Pariente, A. (2018, June). Off-label use of oral morphine sulfate for opioid maintenance purpose in France: results from the 2009-2015 UTOPIA study. In Fundamental & Clinical Pharmacology (Vol. 32, Pp. 41-41).NJ USA: Wiley.
72. Mattick, R. P., Ward, J., & Hall, W. (1998). The role of counselling and psychological therapy. 265-304.
73. Matto, H. C., Strolin, J. S., & Mogro-Wilson, C. (2008). A pilot study of a dual processing substance user treatment intervention with adults. *Substance use & misuse.* 43(3-4), 285-294.
74. McDermott, K. A., Griffin, M. L., Connery, H. S., Hilario, E. Y., Fiellin, D. A., Fitzmaurice, G. M., & Weiss, R. D. (2015). Initial response as a predictor of 12-week buprenorphine-naloxone treatment response in a prescription opioid-dependent population. *The Journal of clinical psychiatry.* 76(2), 189-194.
75. McFall, A.M., Solomon, S.S., Lucas, G.M., Srikrishnan, A.K., Kumar, M.S., Anand, S., Vasudevan, C.K., Celentano, D.D., Mehta, S.H. (2017). High HIV incidence among PWID and MSM attending integrated care centers in India. *Topics in Antiviral Medicine*. 1 (25). 375.
76. Metrebian, N., Weaver, T., Pilling, S., Hellier, J., Byford, S., Shearer, J., ... & Bowden-Jones, O. (2018). Positive reinforcement targeting abstinence in substance misuse (PRAISe): Study protocol for a Cluster RCT & process evaluation of contingency management. *Contemporary clinical trials*, *71*, 124-132.
77. Middleton, L. (2016). HIV testing in the community: Responding to the Glasgow outbreak. *Journal of the International AIDS Society.* 19(Supplement 7), 227-228.
78. Mills, K. L., Barrett, E. L., Merz, S., Rosenfeld, J., Ewer, P. L., Sannibale, C., … & Teesson, M. (2016). Integrated Exposure-Based Therapy for Co-Occurring Post Traumatic Stress Disorder (PTSD) and Substance Dependence: Predictors of Change in PTSD Symptom Severity. *Journal of clinical medicine.* 5(11), 1-20.
79. Monico, L. B., Gryczynski, J., Mitchell, S. G., Schwartz, R. P., O'Grady, K. E., & Jaffe, J. H. (2015). Buprenorphine Treatment and 12-step Meeting Attendance: Conflicts, Compatibilities, and Patient Outcomes. *Journal of substance abuse treatment.* 57, 89-95.
80. Monico, L. B., Gryczynski, J., Schwartz, R. P., Jaffe, J. H., O’Grady, K. E., & Mitchell, S. G. (2018). Treatment outcomes among a cohort of African American buprenorphine patients: Follow-up at 12 months. *The American journal of drug and alcohol abuse*, 44(6), 604-610.
81. Moore, B. A., Barry, D. T., Sullivan, L. E., O'Connor, P. G., Cutter, C. J., Schottenfeld, R. S., & Fiellin, D. A. (2012). Counseling and directly observed medication for primary care buprenorphine maintenance: a pilot study. *Journal of addiction medicine.* 6(3), 205-211.
82. Moore, S. K., Guarino, H., & Marsch, L. A. (2014). "This is not who I want to be:" experiences of opioid-dependent youth before, and during, combined buprenorphine and behavioral treatment. *Substance use & misuse.* 49(3), 303-314.
83. Murray-Lillibridge, K., Barry, J., Reagan, S., O'Flanagan, D., Sayers, G., Bergin, C., … & Fischer, M. (2006). Epidemiological findings and medical, legal, and public health challenges of an investigation of severe soft tissue infections and deaths among injecting drug users - Ireland, 2000. *Epidemiology and Infection.* 134(4), 894-901.
84. Navidian, A., Kermansaravi, F., Tabas, E. E., & Saeedinezhad, F. (2016). Efficacy of Group Motivational Interviewing in the Degree of Drug Craving in the Addicts Under the Methadone Maintenance Treatment (MMT) in South East of Iran. *Archives of psychiatric nursing.* 30(2), 144-149.
85. Nielsen, S., Hillhouse, M., Mooney, L., Ang, A., & Ling, W. (2015). Buprenorphine pharmacotherapy and behavioral treatment: comparison of outcomes among prescription opioid users, heroin users and combination users. *Journal of substance abuse treatment.* 48(1), 70-76.
86. Nosyk, B., Bray, J. W., Wittenberg, E., Aden, B., Eggman, A. A., Weiss, R. D., … & Schackman, B. R. (2015). Short term health-related quality of life improvement during opioid agonist treatment. *Drug and alcohol dependence.* 157, 121-128.
87. Pani, P. P., Pirastu, R., Ricci, A., & Gessa, G. L. (1996). Prohibition of take-home dosages: negative consequences on methadone maintenance treatment. *Drug and alcohol dependence.* 41(1), 81-84.
88. Pantalon, M. V., Fiellin, D. A., O'Connor, P. G., Chawarski, M. C., Pakes, J. R., & Schottenfeld, R. S. (2004). Counseling requirements for buprenorphine maintenance in primary care: Lessons learned from a preliminary study in a methadone maintenance program. *Addictive Disorders and their Treatment.* 3(2), 71-76.
89. Parpouchi, M., Moniruzzaman, A., Rezansoff, S. N., Russolillo, A., & Somers, J. M. (2018). The effect of housing first on adherence to methadone maintenance treatment. *International Journal of Drug Policy*, *56*, 73-80.
90. Peles, E., Sason, A., Schreiber, S., & Adelson, M. (2017). Newborn birth-weight of pregnant women on methadone or buprenorphine maintenance treatment: A national contingency management approach trial. *The American journal on addictions*. 26(2), 167-175.
91. Premkumar, A., Grobman, W., Terplan, M., & Miller, E. (2019). Methadone, buprenorphine, or detoxification for management of perinatal opioid use disorder: a cost-effectiveness analysis. *American Journal of Obstetrics and Gynecology*, *220*(1).
92. Reimer, J., Schmidt, C. S., Schulte, B., Gansefort, D., Golz, J., Gerken, G., … & Backmund, M. (2013). Psychoeducation improves hepatitis C virus treatment during opioid substitution therapy: a controlled, prospective multicenter trial. *Clinical infectious diseases : an official publication of the Infectious Diseases Society of America.* 57 Suppl 2, S97-104.
93. Robbins, M. S., Alonso, E., Horigian, V. E., Bachrach, K., Burlew, K., Carrion, I. S., … & Szapocznik, Jose. (2010). Transporting clinical research to community settings: designing and conducting a multisite trial of brief strategic family therapy. *Addiction science & clinical practice.* 5(2), 54-61.
94. Robbins, M. S., Szapocznik, J., Horigian, V. E., Feaster, D. J., Puccinelli, M., Jacobs, P., … & Brigham, G. (2009). Brief strategic family therapy for adolescent drug abusers: a multi-site effectiveness study. *Contemporary clinical trials.* 30(3), 269-278.
95. Rose, M. (2018). Guideline: In adults with opioid use disorders, a stepped treatment approach is recommended. *Annals of internal medicine*, 168(12), JC62-JC62.
96. Ross, J., Teesson, M., Lejuez, C., Mills, K., Kaye, S., Brady, K., … & Masters, S. L. (2016). The efficacy of behavioural activation treatment for co-occurring depression and substance use disorder (the activate study): a randomized controlled trial. *BMC psychiatry.* 16, 221.
97. Saunders, B., Wilkinson, C., & Phillips, M. (1995). The impact of a brief motivational intervention with opiate users attending a methadone programme. *Addiction (Abingdon, England).* 90(3), 415-424.
98. Schottenfeld, R. S., Pantalon, M. V., Chawarski, M. C., & Pakes, J. (2000). Community reinforcement approach for combined opioid and cocaine dependence. Patterns of engagement in alternate activities. *Journal of substance abuse treatment.* 18(3), 255-261.
99. Schroeder, J. R., Gupman, A. E., Epstein, D. H., Umbricht, A., & Preston, K. L. (2003). Do noncontingent vouchers increase drug use? *Experimental and clinical psychopharmacology.* 11(3), 195-201.
100. Schwartz, R. P., Kelly, S. M., Mitchell, S. G., Dunlap, L., Zarkin, G. A., Sharma, A., … & Jaffe, J. H. (2016). Interim methadone and patient navigation in jail: Rationale and design of a randomized clinical trial. *Contemporary clinical trials.* 49, 21-28.
101. Scott, C. K., Dennis, M. L., & Gustafson, D. H. (2017). Using smartphones to decrease substance use via self-monitoring and recovery support: study protocol for a randomized control trial. *Trials*, *18*(1), 374.
102. Seraganian, P., Brown, T. G., & Tremblay, J. (2003). Randomization in a substance abuse treatment study: participants who consent vs those who do not. *Canadian journal of psychiatry. Revue canadienne de psychiatrie.* 48(6), 388-394.
103. Setodji, C. M., Watkins, K. E., Hunter, S. B., McCullough, C., Stein, B. D., Osilla, K. C., & Ober, A. J. (2018). Initiation and engagement as mechanisms for change caused by collaborative care in opioid and alcohol use disorders. *Drug and alcohol dependence*, *192*, 67-73.
104. Sigurdsson, S. O., DeFulio, A., Long, L., & Silverman, K. (2011). Propensity to work among chronically unemployed adult drug users. *Substance use & misuse.* 46(5), 599-607.
105. Skinner, M. L., Haggerty, K. P., Fleming, C. B., & Catalano, R. F. (2009). Predicting functional resilience among young-adult children of opiate-dependent parents. *The Journal of adolescent health : official publication of the Society for Adolescent Medicine.* 44(3), 283-290.
106. Smyth, B. P., Ducray, K., & Cullen, W. (2018). Changes in psychological well‐being among heroin‐dependent adolescents during psychologically supported opiate substitution treatment. *Early intervention in psychiatry*, 12(3), 417-425.
107. Socias, M. E., Ahamad, K., Le Foll, B., Lim, R., Bruneau, J., Fischer, B., ... & Jutras-Aswad, D. (2018). The OPTIMA study, buprenorphine/naloxone and methadone models of care for the treatment of prescription opioid use disorder: Study design and rationale. *Contemporary clinical trials*, *69*, 21-27.
108. Sorensen, J. L., London, J., Heitzmann, C., Gibson, D. R., Morales, E. S., Dumontet, R., & Acree, M. (1994). Psychoeducational group approach: HIV risk reduction in drug users. *AIDS education and prevention : official publication of the International Society for AIDS Education.* 6(2), 95-112.
109. Sun, B., Kim, H., Lupulescu-Mann, N., Charlesworth, C., Hartung, D., Deyo, R., & McConnell, K. J. (2016). Impact of hospital mandates on emergency department opioid "best practices" prescribing and pain-related visits. *Annals of emergency medicine.* 68(4 Supplement 1), S51.
110. Tabi, K., Choi, F., Mithani, Z., Nikoo, M., Jang, K., & Krausz, M. (2019). History of parenting instability and lifetime suicidal behavior in people who inject drugs. *Psychiatry research*. 280, 112493.
111. Tait, R. J., & Hulse, G. K. (2003). A systematic review of the effectiveness of brief interventions with substance using adolescents by type of drug (DARE structured abstract). *Drug and alcohol review.* 22, 337-346.
112. Tuten, M., Shadur, J. M., Stitzer, M., & Jones, H. E. (2017). A Comparison of Reinforcement Based Treatment (RBT) versus RBT plus Recovery Housing (RBTRH). *Journal of substance abuse treatment.* 72, 48-55.
113. Van Dorn, R. A., Desmarais, S. L., Rade, C. B., Burris, E. N., Cuddeback, G. S., Johnson, K. L., ... & Mueser, K. T. (2017). Jail-to-community treatment continuum for adults with co-occurring substance use and mental disorders: study protocol for a pilot randomized controlled trial. *Trials*, *18*(1), 365.
114. Wang, K., Fu, H., Longfield, K., Modi, S., Mundy, G., & Firestone, R. (2014). Do community-based strategies reduce HIV risk among people who inject drugs in China? A quasi-experimental study in Yunnan and Guangxi provinces. *Harm reduction journal.* 11, 15.
115. Wang, Z., Chen, S., Chen, J., Xu, C., Chen, Z., Zhuang, W., ... & JIANG, H. (2018). A Community-Based Addiction Rehabilitation Electronic System to Improve Treatment Outcomes in Drug Abusers: Protocol for a Randomized Controlled Trial. *Frontiers in Psychiatry*, *9*, 556.
116. Wasserman, D. A., Stewart, A. L., & Delucchi, K. L. (2001). Social support and abstinence from opiates and cocaine during opioid maintenance treatment. *Drug and alcohol dependence.* 65(1), 65-75.
117. Watkins, K. E., Hunter, S. B., Hepner, K. A., Paddock, S. M., De La Cruz, E., Zhou, A. J., & Gilmore, J. (2011). An effectiveness trial of group cognitive behavioral therapy for patients with persistent depressive symptoms in substance abuse treatment. *Archives of general psychiatry.* 68(6), 577-584.
118. Watkins, K. E., Hunter, S., Hepner, K., Paddock, S., Zhou, A., & De La Cruz, E. (2012). Group cognitive-behavioral therapy for clients with major depression in residential substance abuse treatment. *Psychiatric Services.* 63(6), 608-611.
119. Watkins, K. E., Cuellar, A. E., Hepner, K. A., Hunter, S. B., Paddock, S. M., Ewing, B. A., & de la Cruz, E. (2014). The cost-effectiveness of depression treatment for co-occurring disorders: a clinical trial. *Journal of substance abuse treatment.* 46(2), 128-133.
120. Weiss, R., Griffin, M. L., McDermott, K., McHugh, K., Karakula, S. L., & Fitzmaurice, G. (2017). Pain severity and subsequent opioid use during buprenorphine-naloxone treatment of prescription opioid-dependent patients with chronic pain. *Drug and alcohol dependence.* 171, e215-e216.
121. Weiss, R. D., Potter, J. S., Provost, S. E., Huang, Z., Jacobs, P., Hasson, A., … & Ling, W.. (2010). A multi-site, two-phase, Prescription Opioid Addiction Treatment Study (POATS): rationale, design, and methodology. *Contemporary clinical trials.* 31(2), 189-199.
122. Wells, E. A., Donovan, D. M., Daley, D. C., Doyle, S. R., Brigham, G., Garrett, S. B., … & Walker, R. (2014). Is level of exposure to a 12-step facilitation therapy associated with treatment outcome? *Journal of substance abuse treatment.* 47(4), 265-274.
123. Winters, K. C., Stinchfield, R., Latimer, W. W., & Lee, S. (2007). Long-term outcome of substance-dependent youth following 12-step treatment. *Journal of substance abuse treatment.* 33(1), 61-69.
124. White, B., Dore, G. J., Lloyd, A. R., Rawlinson, W. D., & Maher, L. (2014). Opioid substitution therapy protects against hepatitis C virus acquisition in people who inject drugs: the HITS‐c study. *Medical Journal of Australia*, 201(6), 326-329.
125. Wong, M. C., Isaacson, K. B., & Morris, S. (2018). Opioid Use After Laparoscopic Hysterectomy: Prescribing Practices and Preoperative Predictors [5Q]. Obstetrics & Gynecology, 131, 185S.
126. Woody, G.E. Extended vs short-term buprenorphine-naloxone for treatment of opioid-addicted youth: a randomized trial (Errata corrige). JAMA. 319(14), 1461.
127. Worley, M. J., Heinzerling, K. G., Shoptaw, S., & Ling, W. (2015). Pain volatility and prescription opioid addiction treatment outcomes in patients with chronic pain. *Experimental and clinical psychopharmacology.* 23(6), 428-435.
128. Yousefi, H., Ghaderi Rammazi, M., Abolghasemi, A., & Divsalar, K. (2019). Effects of Self-Control Training on Emotional Wellbeing and Opioid Craving Among Men with Opioid Use Disorder. Iranian *Journal of Psychiatry and Behavioral Sciences*. 13(1).
129. Zhu, Y., Evans, E. A., Mooney, L. J., Saxon, A. J., Kelleghan, A., Yoo, C., & Hser, Y. I. (2018). Correlates of long-term opioid abstinence after randomization to methadone versus buprenorphine/naloxone in a multi-site trial. *Journal of Neuroimmune Pharmacology*, *13*(4), 488-497.

**Not a Study with Results for Individuals with Problematic Opioid Use (n=277)**

1. Acevedo, A., Lee, M. T., Garnick, D. W., Horgan, C. M., Ritter, G. A., Panas, L., … & Bean-Mortinson, J. (2018). Agency-level financial incentives and electronic reminders to improve continuity of care after discharge from residential treatment and detoxification. *Drug and alcohol dependence.* 183, 192-200.
2. Aharonovich, E., Stohl, M., Cannizzaro, D., & Hasin, D. (2017). HealthCall delivered via smartphone to reduce co-occurring drug and alcohol use in HIV-infected adults: a randomized pilot trial. *Journal of substance abuse treatment*, *83*, 15-26.
3. Ahmad-Abadi, F. K., Maarefvand, M., Aghaei, H., Hosseinzadeh, S., Abbasi, M., & Khubchandani, J. (2017). Effectiveness of Satir-informed family-therapy on the codependency of drug dependents’ family members in Iran: a randomized controlled trial. *Journal of evidence-informed social work*, *14*(4), 301-310.
4. Alterman, A. I., Koppenhaver, J. M., Mulholland, E., Ladden, L. J., & Baime, M. J. (2004). Pilot trial of effectiveness of mindfulness meditation for substance abuse patients. *Journal of Substance Use.* 9(6), 259-268.
5. Azrin, N. H., McMahon, P. T., Donohue, B., Besalel, V. A., Lapinski, K. J., Kogan, E. S., … & Galloway, E. (1994). Behavior therapy for drug abuse: a controlled treatment outcome study. *Behaviour research and therapy.* 32(8), 857-866.
6. Babaie, E., & Razeghi, N. (2013). Comparing the effects of methadone maintenance treatment, therapeutic community, and residential rehabilitation on quality of life and mental health of drug addicts. *Addiction & health.* 5(1-2), 16-20.
7. Back, S. E., McCauley, J. L., Korte, K. J., Gros, D. F., Leavitt, V., Gray, K. M., … & Kalivas, P. W. (2016). A Double-Blind, Randomized, Controlled Pilot Trial of N-Acetylcysteine in Veterans With Posttraumatic Stress Disorder and Substance Use Disorders. *The Journal of clinical psychiatry.* 77(11), e1439-e1446.
8. Badour, C. L., Flanagan, J. C., Gros, D. F., Killeen, T., Pericot-Valverde, I., Korte, K. J., … & Back, S. E. (2017). Habituation of distress and craving during treatment as predictors of change in PTSD symptoms and substance use severity. *Journal of consulting and clinical psychology.* 85(3), 274-281.
9. Baer, J. S., Garrett, S. B., Beadnell, B., Wells, E. A., & Peterson, P. L. (2007). Brief motivational intervention with homeless adolescents: evaluating effects on substance use and service utilization. *Psychology of addictive behaviors : journal of the Society of Psychologists in Addictive Behaviors.* 21(4), 582-586.
10. Baer, J. S., Wells, E. A., Rosengren, D. B., Hartzler, B., Beadnell, B., & Dunn, C. (2009). Agency context and tailored training in technology transfer: a pilot evaluation of motivational interviewing training for community counselors. *Journal of substance abuse treatment.* 37(2), 191-202.
11. Baker, A., Lewin, T., Reichler, H., Clancy, R., Carr, V., Garrett, R., … & Terry, M. (2002). Evaluation of a motivational interview for substance use within psychiatric in-patient services. *Addiction (Abingdon, England).* 97(10), 1329-1337.
12. Ball, S. A., Maccarelli, L. M., LaPaglia, D. M., & Ostrowski, M. J. (2011). Randomized trial of dual-focused vs. single-focused individual therapy for personality disorders and substance dependence. *The Journal of nervous and mental disease.* 199(5), 319-328.
13. Ball, S. A., Martino, S., Nich, C., Frankforter, T. L., Van Horn, D., Crits-Christoph, P., … & National Institute on Drug Abuse Clinical Trials Network. (2007). Site matters: multisite randomized trial of motivational enhancement therapy in community drug abuse clinics. *Journal of consulting and clinical psychology.* 75(4), 556-567.
14. Banerjee, K., Howard, M., Mansheim, K., & Beattie, M. (2007). Comparison of Health Realization and 12-Step treatment in women's residential substance abuse treatment programs. *The American journal of drug and alcohol abuse.* 33(2), 207-215.
15. Barry, D., Sullivan, B., & Petry, N. M. (2009). Comparable efficacy of contingency management for cocaine dependence among African American, Hispanic, and White methadone maintenance clients. *Psychology of addictive behaviors: journal of the Society of Psychologists in Addictive Behaviors.* 23(1), 168-174.
16. Bassett, S. S., Stein, L. A. R., Rossi, J. S., & Martin, R. A. (2016). Evaluating Measures of Fidelity for Substance Abuse Group Treatment With Incarcerated Adolescents. *Journal of substance abuse treatment.* 66, 9-15.
17. Begun, A. L., Rose, S. J., & Lebel, T. P. (2011). Intervening with women in jail around alcohol and substance abuse during preparation for community reentry. *Alcoholism Treatment Quarterly.* 29(4), 453-478.
18. Bell, M. D., Laws, H. B., & Petrakis, I. B. (2017). A randomized controlled trial of cognitive remediation and work therapy in the early phase of substance use disorder recovery for older veterans: Neurocognitive and substance use outcomes. *Psychiatric rehabilitation journal.* 40(1), 94-102.
19. Berlin, L. J., Shanahan, M., & Appleyard, C. K. (2014). Promoting supportive parenting in new mothers with substance-use problems: A pilot randomized trial of residential treatment plus an attachment-based parenting program. *Infant Mental Health Journal.* 35(1), 81-85.
20. Berman, A. H., Forsberg, L., Durbeej, N., Kallmen, H., & Hermansson, U. (2010). Single-session motivational interviewing for drug detoxification inpatients: effects on self-efficacy, stages of change and substance use. *Substance use & misuse.* 45(3), 384-402.
21. Binswanger, I. A., Whitley, E., Haffey, P. R., Mueller, S. R., & Min, S. J. (2015). A patient navigation intervention for drug-involved former prison inmates. *Substance abuse.* 36(1), 34-41.
22. Boden, M. T., Kimerling, R., Jacobs-Lentz, J., Bowman, D., Weaver, C., Carney, D., … & Trafton, J. A. (2012). Seeking Safety treatment for male veterans with a substance use disorder and post-traumatic stress disorder symptomatology. *Addiction (Abingdon, England).* 107(3), 578-586.
23. Bohnert, A. S. B., Bonar, E. E., Cunningham, R., Greenwald, M. K., Thomas, L., Chermack, S., … & Walton, M. (2016). A pilot randomized clinical trial of an intervention to reduce overdose risk behaviors among emergency department patients at risk for prescription opioid overdose. *Drug and alcohol dependence.* 163, 40-47.
24. Bortolon, C. B., Moreira, T. D. C., Signor, L., Guahyba, B. L., Figueiró, L. R., Ferigolo, M., & Barros, H. M. T. (2017). Six-month outcomes of a randomized, motivational tele-intervention for change in the codependent behavior of family members of drug users. *Substance use & misuse*, *52*(2), 164-174.
25. Bowen, S., Chawla, N., Collins, S. E., Witkiewitz, K., Hsu, S., Grow, J., … & Marlatt, A. (2009). Mindfulness-based relapse prevention for substance use disorders: A pilot efficacy trial. *Substance abuse.* 30(4), 295-305.
26. Bowen, S., Witkiewitz, K., Clifasefi, S. L., Grow, J., Chawla, N., Hsu, S. H., … & Larimer, M. E. (2014). Relative efficacy of mindfulness-based relapse prevention, standard relapse prevention, and treatment as usual for substance use disorders: a randomized clinical trial. *JAMA Psychiatry.* 71(5), 547-556.
27. Braucht, G. N., Reichardt, C. S., Geissler, L. J., Bormann, C. A., Kwiatkowski, C. F., & Kirby, M. W. J. (1995). Effective services for homeless substance abusers. *Journal of addictive diseases.* 14(4), 87-109.
28. Brown, S. A., Glasner-Edwards, S. V., Tate, S. R., McQuaid, J. R., Chalekian, J., & Granholm, E. (2006). Integrated cognitive behavioral therapy versus twelve-step facilitation therapy for substance-dependent adults with depressive disorders. *Journal of psychoactive drugs.* 38(4), 449-460.
29. Burduli, E., Skalisky, J., Hirchak, K., Orr, M. F., Foote, A., Granbois, A., ... & McPherson, S. M. (2018). Contingency management intervention targeting co-addiction of alcohol and drugs among American Indian adults: Design, methodology, and baseline data. *Clinical Trials*, *15*(6), 587-599.
30. Calsyn, D. A., Hatch-Maillette, M., Tross, S., Doyle, S. R., Crits-Christoph, P., Song, Y. S., … & Berns, S. B. (2009). Motivational and skills training HIV/sexually transmitted infection sexual risk reduction groups for men. *Journal of substance abuse treatment.* 37(2), 138-150.
31. Campbell, A. N. C., Nunes, E. V., Matthews, A. G., Stitzer, M., Miele, G. M., Polsky, D., … & Ghitza, U. E. (2014). Internet-delivered treatment for substance abuse: A multisite randomized controlled trial. *American Journal of Psychiatry.* 171(6), 683-690.
32. Campbell, A. N. C., Nunes, E. V., Pavlicova, M., Hatch-Maillette, M., Hu, M.-C., Bailey, G. L., … & Greenfield, S. F. (2015). Gender-based outcomes and acceptability of a computer-assisted psychosocial intervention for substance use disorders. *Journal of substance abuse treatment.* 53, 9-15.
33. Campbell, A., Nunes, E. V., & Pavlicova, M. (2017). Clinician involvement with internet-delivered treatment and association to outcomes. *Drug and alcohol dependence.* 171, e32.
34. Campbell, B. K., Fuller, B. E., Lee, E. S., Tillotson, C., Woelfel, T., Jenkins, L., … & McCarty, D. (2009). Facilitating outpatient treatment entry following detoxification for injection drug use: a multisite test of three interventions. *Psychology of addictive behaviors : journal of the Society of Psychologists in Addictive Behaviors.* 23(2), 260-270.
35. Capone, C., & McGovern, M. P. (2016). Recruitment and retention of Iraq and Afghanistan veterans in integrated CBT for co-occurring SUD and PTSD. *Alcoholism: Clinical and Experimental Research.* 40(SUPPL. 1), 271A.
36. Carroll, K. M., Ball, S. A., Martino, S., Nich, C., Babuscio, T. A., & Rounsaville, B. J. (2009). Enduring effects of a computer-assisted training program for cognitive behavioral therapy: a 6-month follow-up of CBT4CBT. *Drug and alcohol dependence.* 100(1-2), 178-181.
37. Carroll, K. M., Ball, S. A., Martino, S., Nich, C., Babuscio, T. A., Nuro, K. F., … & Rounsaville, B. J. (2008). Computer-assisted delivery of cognitive-behavioral therapy for addiction: a randomized trial of CBT4CBT. *The American journal of psychiatry.* 165(7), 881-888.
38. Carroll, K. M., Ball, S. A., Nich, C., Martino, S., Frankforter, T. L., Farentinos, C., … & National Institute on Drug Abuse Clinical Trials Network. (2006). Motivational interviewing to improve treatment engagement and outcome in individuals seeking treatment for substance abuse: a multisite effectiveness study. *Drug and alcohol dependence.* 81(3), 301-312.
39. Carroll, K. M., Martino, S., Ball, S. A., Nich, C., Frankforter, T., Anez, L. M., … & Farentinos, C. (2009). A multisite randomized effectiveness trial of motivational enhancement therapy for Spanish-speaking substance users. *Journal of consulting and clinical psychology.* 77(5), 993-999.
40. Carroll, K. M., Nich, C., Frankforter, T. L., Yip, S. W., Kiluk, B. D., DeVito, E. E., & Sofuoglu, M. (2018). Accounting for the uncounted: physical and affective distress in individuals dropping out of oral naltrexone treatment for opioid use disorder. *Drug and alcohol dependence*, *192*, 264-270.
41. Chutuape, M. A., Katz, E. C., & Stitzer, M. L. (2001). Methods for enhancing transition of substance dependent patients from inpatient to outpatient treatment. *Drug and alcohol dependence.* 61(2), 137-143.
42. Chutuape, M. A., Silverman, K., & Stitzer, M. L. (1999). Use of methadone take-home contingencies with persistent opiate and cocaine abusers. *Journal of substance abuse treatment.* 16(1), 23-30.
43. Clark, R. E., Teague, G. B., Ricketts, S. K., Bush, P. W., Xie, H., McGuire, … & Zubkoff, M. (1998). Cost-effectiveness of assertive community treatment versus standard case management for persons with co-occurring severe mental illness and substance use disorders. *Health services research.* 33(5 Pt 1), 1285-1308.
44. Coffey, S. F., Schumacher, J. A., Nosen, E., Littlefield, A. K., Henslee, A. M., Lappen, A., & Stasiewicz, P. R. (2016). Trauma-focused exposure therapy for chronic posttraumatic stress disorder in alcohol and drug dependent patients: A randomized controlled trial. *Psychology of addictive behaviors: journal of the Society of Psychologists in Addictive Behaviors.* 30(7), 778-790.
45. Comulada, W. S., Weiss, R. E., Cumberland, W., & Rotheram-Borus, M. J. (2007). Reductions in drug use among young people living with HIV. *The American journal of drug and alcohol abuse.* 33(3), 493-501.
46. Condelli, W. S., Koch, M. A., & Fletcher, B. (2000). Treatment refusal/attrition among adults randomly assigned to programs at a drug treatment campus: The New Jersey Substance Abuse Treatment Campus, Seacaucus, NJ. *Journal of substance abuse treatment.* 18(4), 395-407.
47. Conrod, P. J., Stewart, S. H., Pihl, R. O., Cote, S., Fontaine, V., & Dongier, M. (2000). Efficacy of brief coping skills interventions that match different personality profiles of female substance abusers. *Psychology of addictive behaviors: journal of the Society of Psychologists in Addictive Behaviors.* 14(3), 231-242.
48. Cornish, J. W., Metzger, D., Woody, G. E., Wilson, D., McLellan, A. T., Vandergrift, B., & O'Brien, C. P. (1997). Naltrexone pharmacotherapy for opioid dependent federal probationers. *Journal of substance abuse treatment.* 14(6), 529-534.
49. Corrigan, J. D., Bogner, J., Lamb-Hart, G., Heinemann, A. W., & Moore, D. (2005). Increasing substance abuse treatment compliance for persons with traumatic brain injury. *Psychology of addictive behaviors: journal of the Society of Psychologists in Addictive Behaviors.* 19(2), 131-139.
50. Courbasson, C., Nishikawa, Y., & Dixon, L. (2012). Outcome of dialectical behaviour therapy for concurrent eating and substance use disorders. *Clinical psychology & psychotherapy.* 19(5), 434-449.
51. Coviello, D. M., Zanis, D. A., Wesnoski, S. A., & Alterman, A. I. (2006). The effectiveness of outreach case management in re-enrolling discharged methadone patients. *Drug and alcohol dependence.* 85(1), 56-65.
52. Cusack, K. J., Morrissey, J. P., Cuddeback, G. S., Prins, A., & Williams, D. M. (2010). Criminal justice involvement, behavioral health service use, and costs of forensic assertive community treatment: a randomized trial. *Community mental health journal.* 46(4), 356-363.
53. Dakof, G. A., Cohen, J. B., Henderson, C. E., Duarte, E., Boustani, M., Blackburn, A., … & Hawes, S. (2010). A randomized pilot study of the Engaging Moms Program for family drug court. *Journal of substance abuse treatment.* 38(3), 263-274.
54. Dakof, G. A., Henderson, C. E., Rowe, C. L., Boustani, M., Greenbaum, P. E., Wang, W., … & Liddle, H. A. (2015). A randomized clinical trial of family therapy in juvenile drug court. *Journal of family psychology: JFP : journal of the Division of Family Psychology of the American Psychological Association.* 29(2), 232-241.
55. Daley, M., Shepard, D. S., Tompkins, C., Dunigan, R., Reif, S., Perloff, J., … & Horgan, C. (2011). Randomized trial of enhanced profiling in substance abuse treatment. *Administration and policy in mental health.* 38(2), 96-104.
56. Davis, P., & Abou-Saleh, M. T. (2008). Developing an enhanced counseling intervention for the primary prevention of hepatitis C among injecting drug users. *Addictive Disorders and their Treatment.* 7(2), 65-75.
57. de Haan, H., Joosten, E., Wijdeveld, T., Boswinkel, P., van der Palen, J., & De Jong, C. (2012). Alexithymia is not a stable personality trait in patients with substance use disorders. *Psychiatry research.* 198(1), 123-129.
58. Delgadillo, J., Gore, S., Ali, S., Ekers, D., Gilbody, S., Gilchrist, G., … & Hughes, E. (2015). Feasibility randomized controlled trial of cognitive and behavioral interventions for depression symptoms in patients accessing drug and alcohol treatment. *Journal of substance abuse treatment.* 55, 6-14.
59. Donovan, D. M., Daley, D. C., Brigham, G. S., Hodgkins, C. C., Perl, H. I., Garrett, S. B., … & Zammarelli, L. (2013). Stimulant abuser groups to engage in 12-step: a multisite trial in the National Institute on Drug Abuse Clinical Trials Network. *Journal of substance abuse treatment.* 44(1), 103-114.
60. Drapkin, M. L., Tate, S. R., McQuaid, J. R., & Brown, S. A. (2008). Does initial treatment focus influence outcomes for depressed substance abusers? *Journal of substance abuse treatment.* 35(3), 343-350.
61. Drebing, C. E., Van Ormer, E. A., Mueller, L., Hebert, M., Penk, W. E., Petry, N. M., … & Rounsaville, B. (2007). Adding contingency management intervention to vocational rehabilitation: outcomes for dually diagnosed veterans. *Journal of rehabilitation research and development.* 44(6), 851-865.
62. Easton, C. J., Crane, C. A., & Mandel, D. (2017). A Randomized Controlled Trial Assessing the Efficacy of Cognitive Behavioral Therapy for Substance-Dependent Domestic Violence Offenders: An Integrated Substance Abuse-Domestic Violence Treatment Approach (SADV). *Journal of Marital and Family Therapy.* 1-16.
63. Eisen, M., Keyser-Smith, J., Dampeer, J., & Sambrano, S. (2000). Evaluation of substance use outcomes in demonstration projects for pregnant and postpartum women and their infants: findings from a quasi-experiment. *Addictive behaviors.* 25(1), 123-129.
64. Fals-Stewart, W., & Schafer, J. (1992). The treatment of substance abusers diagnosed with obsessive-compulsive disorder: an outcome study. *Journal of substance abuse treatment*. 9(4), 365-370.
65. Fals-Stewart, W., Birchler, G. R., & O'Farrell, T. J. (1996). Behavioral couples therapy for male substance-abusing patients: effects on relationship adjustment and drug-using behavior. *Journal of consulting and clinical psychology.* 64(5), 959-972.
66. Fals-Stewart, W., O'Farrell, T. J., & Birchler, G. R. (1997). Behavioral couples therapy for male substance-abusing patients: a cost outcomes analysis. *Journal of consulting and clinical psychology.* 65(5), 789-802.
67. Fals-Stewart, W., & Lam, W. K. K. (2010). Computer-assisted cognitive rehabilitation for the treatment of patients with substance use disorders: a randomized clinical trial. *Experimental and clinical psychopharmacology.* 18(1), 87-98.
68. Field, C. A., Adinoff, B., Harris, T. R., Ball, S. A., & Carroll, K. M. (2009). Construct, concurrent and predictive validity of the URICA: data from two multi-site clinical trials. *Drug and alcohol dependence.* 101(1-2), 115-123.
69. Fisher, M. S. S., & Bentley, K. J. (1996). Two group therapy models for clients with a dual diagnosis of substance abuse and personality disorder. *Psychiatric services (Washington, D.C.).* 47(11), 1244-1250.
70. Fletcher, J. B., Dierst-Davies, R., & Reback, C. J. (2014). Contingency management voucher redemption as an indicator of delayed gratification. *Journal of substance abuse treatment.* 47(1), 73-77.
71. Fletcher, J. B., Shoptaw, S., Peck, J. A., & Reback, C. J. (2014). Contingency Management Reduces Symptoms of Psychological and Emotional Distress among Homeless, Substance-dependent Men Who Have Sex with Men. *Mental health and substance use: dual diagnosis.* 7(4), 420-430.
72. Ford, J. D., Hawke, J., Alessi, S., Ledgerwood, D., & Petry, N. (2007). Psychological trauma and PTSD symptoms as predictors of substance dependence treatment outcomes. *Behaviour research and therapy.* 45(10), 2417-2431.
73. Ford II, J. H., Stumbo, S. P., & Robinson, J. M. (2018). Assessing long-term sustainment of clinic participation in NIATx200: Results and a new methodological approach. *Journal of substance abuse treatment*, *92*, 51-63.
74. French, M. T., Zavala, S. K., McCollister, K. E., Waldron, H. B., Turner, C. W., & Ozechowski, T. J. (2008). Cost-effectiveness analysis of four interventions for adolescents with a substance use disorder. *Journal of substance abuse treatment.* 34(3), 272-281.
75. Friedmann, P. D., Green, T. C., Taxman, F. S., Harrington, M., Rhodes, A. G., Katz, E., … & Step'n Out Research Group of CJ-DATS. (2012). Collaborative behavioral management among parolees: drug use, crime and re-arrest in the Step'n Out randomized trial. *Addiction.* 107(6), 1099-1108.
76. Garland, E. L., Bryan, C. J., Finan, P. H., Thomas, E. A., Priddy, S. E., Riquino, M. R., & Howard, M. O. (2017). Pain, hedonic regulation, and opioid misuse: Modulation of momentary experience by Mindfulness-Oriented Recovery Enhancement in opioid-treated chronic pain patients. *Drug and alcohol dependence.* 173 Suppl 1, S65-S72.
77. Gilchrist, G., Swan, D., Shaw, A., Keding, A., Towers, S., Craine, N., … & Watson, J. (2017). The acceptability and feasibility of a brief psychosocial intervention to reduce blood-borne virus risk behaviours among people who inject drugs: a randomised control feasibility trial of a psychosocial intervention (the PROTECT study) versus treatment as usual. *Harm reduction journal.* 14(14), 1-15.
78. Glasner-Edwards, S., Tate, S. R., McQuaid, J. R., Cummins, K., Granholm, E., & Brown, S. A. (2007). Mechanisms of action in integrated cognitive-behavioral treatment versus twelve-step facilitation for substance-dependent adults with comorbid major depression. *Journal of studies on alcohol and drugs.* 68(5), 663-672.
79. Godley, M. D., Godley, S. H., Dennis, M. L., Funk, R. R., & Passetti, L. L. (2007). The effect of assertive continuing care on continuing care linkage, adherence and abstinence following residential treatment for adolescents with substance use disorders. *Addiction (Abingdon, England).* 102(1), 81-93.
80. Godley, M. D., Godley, S. H., Dennis, M. L., Funk, R. R., Passetti, L. L., & Petry, N. M. (2014). A randomized trial of assertive continuing care and contingency management for adolescents with substance use disorders. *Journal of consulting and clinical psychology.* 82(1), 40-51.
81. Godley, S. H., Garner, B. R., Passetti, L. L., Funk, R. R., Dennis, M. L., & Godley, M. D. (2010). Adolescent outpatient treatment and continuing care: main findings from a randomized clinical trial. *Drug and alcohol dependence.* 110(1-2), 44-54.
82. Godley, M. D., Passetti, L. L., Hunter, B. D., Greene, A. R., & White, W. L. (2019). A randomized trial of volunteer recovery support for adolescents (VRSA) following residential treatment discharge. *Journal of Substance Abuse Treatment*. 98, 15-25.
83. Gorgels, W. J. M. J., Oude V., R. C., Mol, A. J. J., van De Lisdonk, E. H., Mulder, J., van Den, H. H., … & Zitman, F. G. (2007). Consequences of a benzodiazepine discontinuation programme in family practice on psychotropic medication prescription to the participants. *Family practice.* 24(5), 504-510.
84. Goti, J., Diaz, R., Serrano, L., Gonzalez, L., Calvo, R., Gual, A., & Castro, J. (2010). Brief intervention in substance-use among adolescent psychiatric patients: a randomized controlled trial. *European child & adolescent psychiatry.* 19(6), 503-511.
85. Gottheil, E., Thornton, C. C., & Weinstein, S. P. (1997). Treatment structure, client coping methods, and response to brief individual counseling: preliminary findings in a substance dependent sample. *Journal of addictive diseases.* 16(3), 51-65.
86. Gottheil, E., Thornton, C., & Weinstein, S. (2002). Effectiveness of high versus low structure individual counseling for substance abuse. *The American journal on addictions.* 11(4), 279-290.
87. Greenfield, B. L., Roos, C., Hagler, K. J., Stein, E., Bowen, S., & Witkiewitz, K. A. (2018). Race/ethnicity and racial group composition moderate the effectiveness of mindfulness-based relapse prevention for substance use disorder. *Addictive behaviors*, *81*, 96-103.
88. Greenfield, S. F., Trucco, E. M., McHugh, R. K., Lincoln, M., & Gallop, R. J. (2007). The Women's Recovery Group Study: a Stage I trial of women-focused group therapy for substance use disorders versus mixed-gender group drug counseling. *Drug and alcohol dependence.* 90(1), 39-47.
89. Gryczynski, J., Carswell, S. B., O'Grady, K. E., Mitchell, S. G., & Schwartz, R. P. (2018). Gender and ethnic differences in primary care patients' response to computerized vs. in-person brief intervention for illicit drug misuse. *Journal of substance abuse treatment.* 84, 50-56.
90. Haller, M., Norman, S. B., Cummins, K., Trim, R. S., Xu, X., Cui, R., … & Tate, S. R. (2016). Integrated Cognitive Behavioral Therapy Versus Cognitive Processing Therapy for Adults With Depression, Substance Use Disorder, and Trauma. *Journal of substance abuse treatment.* 62, 38-48.
91. Hanlon, T. E., McCabe, O. L., Savage, C., & Kurland, A. A. (1975). A controlled comparison of cyclazocine and naloxone treatment of the paroled narcotic addict. *International pharmacopsychiatry.* 10(4), 240-250.
92. Hawkins, J. D., Catalano, R. F. J., Gillmore, M. R., & Wells, E. A. (1989). Skills training for drug abusers: generalization, maintenance, and effects on drug use. *Journal of consulting and clinical psychology. 57*(4), 559-563.
93. Henggeler, S. W., Pickrel, S. G., & Brondino, M. J. (1999). Multisystemic treatment of substance-abusing and dependent delinquents: outcomes, treatment fidelity, and transportability. *Mental health services research.* 1(3), 171-184.
94. Henggeler, S. W., Pickrel, S. G., Brondino, M. J., & Crouch, J. L. (1996). Eliminating (almost) treatment dropout of substance abusing or dependent delinquents through home-based multisystemic therapy. *The American journal of psychiatry.* 153(3), 427-428.
95. Hien, D. A., Campbell, A. N. C., Killeen, T., Hu, M. C., Hansen, C., Jiang, H., … & Nunes, E. V. (2010). The impact of trauma-focused group therapy upon HIV sexual risk behaviors in the NIDA Clinical Trials Network "Women and trauma" multi-site study. *AIDS and behavior.* 14(2), 421-430.
96. Hien, D. A., Smith, K. Z., Owens, M., López-Castro, T., Ruglass, L. M., & Papini, S. (2018). Lagged effects of substance use on PTSD severity in a randomized controlled trial with modified prolonged exposure and relapse prevention. *Journal of consulting and clinical psychology.* 86(10), 810.
97. Hien, D. A., Wells, E. A., Jiang, H., Suarez-Morales, L., Campbell, A. N. C., Cohen, L. R., … & Nunes, E. V. (2009). Multisite randomized trial of behavioral interventions for women with co-occurring PTSD and substance use disorders. *Journal of consulting and clinical psychology.* 77(4), 607-619.
98. Ho, S. B., Brau, N., Cheung, R., Liu, L., Sanchez, C., Sklar, M., … & G., Erik J. (2015). Integrated Care Increases Treatment and Improves Outcomes of Patients With Chronic Hepatitis C Virus Infection and Psychiatric Illness or Substance Abuse. *Clinical gastroenterology and hepatology.* 13(11), 2005-3.
99. Hogue, A., & Dauber, S. (2013). Assessing fidelity to evidence-based practices in usual care: the example of family therapy for adolescent behavior problems. *Evaluation and program planning.* 37, 21-30.
100. Hops, H., Ozechowski, T. J., Waldron, H. B., Davis, B., Turner, C. W., Brody, J. L., & Barrera, M. (2011). Adolescent health-risk sexual behaviors: effects of a drug abuse intervention. *AIDS and behavior.* 15(8), 1664-1676.
101. Hubbard, R. L., Leimberger, J. D., Haynes, L., Patkar, A. A., Holter, J., Liepman, M. R., … & Hasson, A. (2007). Telephone Enhancement of Long-term Engagement (TELE) in continuing care for substance abuse treatment: A NIDA Clinical Trials Network (CTN) study. *American Journal on Addictions.* 16(6), 495-502.
102. Humphreys, K., & Moos, R. H. (2007). Encouraging posttreatment self-help group involvement to reduce demand for continuing care services: two-year clinical and utilization outcomes. *Alcoholism, clinical and experimental research.* 31(1), 64-68.
103. Hunter, S. B., Ramchand, R., Griffin, B. A., Suttorp, M. J., McCaffrey, D., & Morral, Andrew. (2012). The effectiveness of community-based delivery of an evidence-based treatment for adolescent substance use. *Journal of substance abuse treatment.* 43(2), 211-220.
104. Ilgen, M. A., Bohnert, A. S., Chermack, S., Conran, C., Jannausch, M., Trafton, J., & Blow, F. C. (2016). A randomized trial of a pain management intervention for adults receiving substance use disorder treatment. *Addiction*, *111*(8), 1385-1393.
105. Ingram, J. A., & Salzberg, H. C. (1990). Effects of in vivo behavioral rehearsal on the learning of assertive behaviors with a substance abusing population. *Addictive behaviors.* 15(2), 189-194.
106. Jaffee, W. B., Bailey, G. L., Lohman, M., Riggs, P., McDonald, L., & Weiss, R. D. (2009). Methods of recruiting adolescents with psychiatric and substance use disorders for a clinical trial. *The American journal of drug and alcohol abuse.* 35(5), 381-384.
107. Jalali, F., Hashemi, S. F., Hasani, A., & Fakoor Sharghi, N. (2017). The Effectiveness of Cognitive Group Therapy Based on Schema-Focused Approach on Self-Esteem and Emotion Regulation in Drug Addicted Prisoners Under the Methadone Maintenance Treatment (MMT). *Journal of Groups in Addiction & Recovery*, *12*(4), 284-295.
108. Jason, L. A., Olson, B. D., Ferrari, J. R., & Lo Sasso, A. T. (2006). Communal housing settings enhance substance abuse recovery. *American journal of public health.* 96(10), 1727-1729.
109. Jason, L. A., Olson, B. D., Ferrari, J. R., Majer, J. M., Alvarez, J., & Stout, J. (2007). An examination of main and interactive effects of substance abuse recovery housing on multiple indicators of adjustment. *Addiction (Abingdon, England).* 102(7), 1114-1121.
110. Kaminer, Y., Burleson, J. A., & Goldberger, R. (2002). Cognitive-behavioral coping skills and psychoeducation therapies for adolescent substance abuse. *The Journal of nervous and mental disease.* 190(11), 737-745.
111. Katz, E. C., Brown, B. S., Schwartz, R. P., King, S. D., Weintraub, E., & Barksdale, W. (2007). Impact of role induction on long-term drug treatment outcomes. *Journal of addictive diseases.* 26(2), 81-90.
112. Kelly, J. F., Kaminer, Y., Kahler, C. W., Hoeppner, B., Yeterian, J., Cristello, J. V., & Timko, C. (2017). A pilot randomized clinical trial testing integrated 12‐Step facilitation (iTSF) treatment for adolescent substance use disorder. *Addiction*, *112*(12), 2155-2166.
113. Kelley, M. L., & Fals-Stewart, W. (2002). Couples- versus individual-based therapy for alcohol and drug abuse: effects on children's psychosocial functioning. *Journal of consulting and clinical psychology.* 70(2), 417-427.
114. Kidorf, M. S. (2018, June). Mobilizing drug-free support and modifying social network ties using close network supports. In *Alcoholism-Clinical And Experimental Research* (Vol. 42, pp. 292A-292A). USA: Wiley.
115. Kikkert, M., Goudriaan, A., De Waal, M., Peen, J., & Dekker, J. (2018). Effectiveness of Integrated Dual Diagnosis Treatment (IDDT) in severe mental illness outpatients with a co-occurring substance use disorder. *Journal of Substance Abuse Treatment*, *95*, 35-42.
116. Killeen, T., Hien, D., Campbell, A., Brown, C., Hansen, C., Jiang, H., … & Nunes, E. (2008). Adverse events in an integrated trauma-focused intervention for women in community substance abuse treatment. *Journal of substance abuse treatment.* 35(3), 304-311.
117. Kiluk, B. D., Nich, C., & Carroll, K. M. (2011). Relationship of cognitive function and the acquisition of coping skills in computer assisted treatment for substance use disorders. *Drug and alcohol dependence.* 114(2-3), 169-176.
118. Kiluk, B. D., Nich, C., Babuscio, T., & Carroll, K. M. (2010). Quality versus quantity: acquisition of coping skills following computerized cognitive-behavioral therapy for substance use disorders. *Addiction (Abingdon, England).* 105(12), 2120-2127.
119. Kiluk, B. D., Nich, C., Buck, M. B., Devore, K. A., Frankforter, T. L., LaPaglia, D. M., ... & Carroll, K. M. (2018). Randomized clinical trial of computerized and clinician-delivered CBT in comparison with standard outpatient treatment for substance use disorders: primary within-treatment and follow-up outcomes. *American Journal of Psychiatry*, *175*(9), 853-863.
120. Kiluk, B. D., Nich, C., Buck, M. B., Devore, K. A., Frankforter, T. L., LaPaglia, D. M., ... & Carroll, K. M. (2018). Randomized clinical trial of computerized and clinician-delivered CBT in comparison with standard outpatient treatment for substance use disorders: primary within-treatment and follow-up outcomes. *American Journal of Psychiatry.* 175(9), 853-863.
121. Kingree, J. B., & Thompson, M. (2000). Mutual help groups, perceived status benefits, and well-being: a test with adult children of alcoholics with personal substance abuse problems. *American journal of community psychology.* 28(3), 325-342.
122. Kropp, F., Winhusen, T., Lewis, D., Hague, D., & Somoza, E. (2010). Increasing prenatal care and healthy behaviors in pregnant substance users. *Journal of psychoactive drugs.* 42(1), 73-81.
123. Lancaster, C. L., Gros, D. F., Mullarkey, M. C., Badour, C. L., Killeen, T. K., Brady, K. T., & Back, S. E. (2020). Does trauma-focused exposure therapy exacerbate symptoms among patients with comorbid PTSD and substance use disorders?. *Behavioural and cognitive psychotherapy*. 48(1), 38-53.
124. Lash, S. J., Burden, J. L., Parker, J. D., Stephens, R. S., Budney, A. J., Horner, R. D., … & Grambow, S. C. (2013). Contracting, prompting and reinforcing substance use disorder continuing care. *Journal of substance abuse treatment.* 44(4), 449-456.
125. Lash, S. J., Gilmore, J. D., Burden, J. L., Weaver, K. R., Blosser, S. L., & Finney, M. L. (2005). The impact of contracting and prompting substance abuse treatment entry: a pilot trial. *Addictive behaviors.* 30(3), 415-422.
126. Lash, S. J., Stephens, R. S., Burden, J. L., Grambow, S. C., DeMarce, J. M., Jones, M. E., … & Horner, R. D. (2007). Contracting, prompting, and reinforcing substance use disorder continuing care: a randomized clinical trial. *Psychology of addictive behaviors: journal of the Society of Psychologists in Addictive Behaviors.* 21(3), 387-397.
127. Law, F. M., & Guo, G. J. (2017). Choice and hope: a preliminary study of the effectiveness of choice-based reality therapy in strengthening hope in recovery for women convicted of drug offences in Taiwan. *International journal of offender therapy and comparative criminology*, *61*(3), 310-333.
128. Law, F. M., & Guo, G. J. (2012). Hope and recovery from substance abuse for female drug offenders in Taiwan. *International journal of offender therapy and comparative criminology.* 56(8), 1258-1282.
129. Law, F. M., & Guo, G. J. (2014). Who is in charge of your recovery? The effectiveness of reality therapy for female drug offenders in Taiwan. *International journal of offender therapy and comparative criminology.* 58(6), 672-696.
130. Law, F. M., & Guo, G. J. (2015). The impact of reality therapy on self-efficacy for substance-involved female offenders in Taiwan. *International journal of offender therapy and comparative criminology.* 59(6), 631-653.
131. Ledgerwood, D. M., Alessi, S. M., Hanson, T., Godley, M. D., & Petry, N. M. (2008). Contingency management for attendance to group substance abuse treatment administered by clinicians in community clinics. *Journal of applied behavior analysis.* 41(4), 517-526.
132. Lee, M. T., Acevedo, A., Garnick, D. W., Horgan, C. M., Panas, L., Ritter, G. A., & Campbell, K. M. (2018). Impact of agency receipt of incentives and reminders on engagement and continuity of care for clients with co-occurring disorders. *Psychiatric services*, *69*(7), 804-811.
133. Lehman, A. F., Herron, J. D., Schwartz, R. P., & Myers, C. P. (1993). Rehabilitation for adults with severe mental illness and substance use disorders. A clinical trial. *The Journal of nervous and mental disease*. 181(2), 86-90.
134. Lehman, W. E. K., Rowan, G. A., Greener, J. M., Joe, G. W., Yang, Y., & Knight, K. (2015). Evaluation of WaySafe: A Disease-Risk Reduction Curriculum for Substance-Abusing Offenders. *Journal of substance abuse treatment.* 58, 25-32.
135. LePage, J. P., & Garcia-Rea, E. A. (2012). Lifestyle coaching's effect on 6-month follow-up in recently homeless substance dependent veterans: a randomized study. *Psychiatric rehabilitation journal.* 35(5), 396-402.
136. Levesque, A., Campbell, A. N. C., Pavlicova, M., Hu, M.-C., Walker, R., McClure, E. A., … & Nunes, E. V. (2017). Coping strategies as a mediator of internet-delivered psychosocial treatment: Secondary analysis from a NIDA CTN multisite effectiveness trial. *Addictive behaviors.* 65, 74-80.
137. Li, L., Hien, N. T., Lin, C., Tuan, N. A., Tuan, L. A., Farmer, S. C., & Detels, R. (2014). An intervention to improve mental health and family well-being of injecting drug users and family members in Vietnam. *Psychology of addictive behaviors: journal of the Society of Psychologists in Addictive Behaviors.* 28(2), 607-613.
138. Li, L., Liang, L. J., Lin, C., Feng, N., Cao, W., & Wu, Z. (2019). An intervention to improve provider-patient interaction at methadone maintenance treatment in China. *Journal of substance abuse treatment.* 99, 149-155.
139. Liang, D., Han, H., Du, J., Zhao, M., & Hser, Y. I. (2018). A pilot study of a smartphone application supporting recovery from drug addiction. *Journal of substance abuse treatment*, *88*, 51-58.
140. Liddle, H. A., Dakof, G. A., Rowe, C. L., Henderson, C., Greenbaum, P., Wang, W., & Alberga, L. (2018). Multidimensional Family Therapy as a community-based alternative to residential treatment for adolescents with substance use and co-occurring mental health disorders. *Journal of substance abuse treatment*, *90*, 47-56.
141. Linehan, M. M., Schmidt, H., Dimeff, L. A., Craft, J. C., Kanter, J., & Comtois, K. A. (1999). Dialectical behavior therapy for patients with borderline personality disorder and drug-dependence. *The American journal on addictions.* 8(4), 279-292.
142. Lowe, J., Liang, H., Riggs, C., Henson, J., & Elder, T. (2012). Community partnership to affect substance abuse among Native American adolescents. *The American journal of drug and alcohol abuse.* 38(5), 450-455.
143. Lydecker, K. P., Tate, S. R., Cummins, K. M., McQuaid, J., Granholm, E., & Brown, S. A. (2010). Clinical Outcomes of an Integrated Treatment for Depression and Substance Use Disorders. *Psychology of Addictive Behaviors.* 24(3), 453-465.
144. Majer, J. M., Jason, L. A., Aase, D. M., Droege, J. R., & Ferrari, J. R. (2013). Categorical 12-step involvement and continuous abstinence at 2 years. *Journal of substance abuse treatment.* 44(1), 46-51.
145. Manning, V., Best, D., Faulkner, N., Titherington, E., Morinan, A., Keaney, F., … & Strang, J. (2012). Does active referral by a doctor or 12-Step peer improve 12-Step meeting attendance? Results from a pilot randomised control trial. *Drug and alcohol dependence.* 126(1-2), 131-137.
146. Manuel, J. I., Covell, N. H., Jackson, C. T., & Essock, S. M. (2011). Does assertive community treatment increase medication adherence for people with co-occurring psychotic and substance use disorders? *Journal of the American Psychiatric Nurses Association.* 17(1), 51-56.
147. Manuel, J. K., Austin, J. L., Miller, W. R., McCrady, B. S., Tonigan, J. S., Meyers, R. J., … & Bogenschutz, M. P. (2012). Community Reinforcement and Family Training: a pilot comparison of group and self-directed delivery. *Journal of substance abuse treatment.* 43(1), 129-136.
148. Marcus, M. T., Schmitz, J., Moeller, G., Liehr, P., Cron, S. G., Swank, P., … & Granmayeh, L. K. (2009). Mindfulness-based stress reduction in therapeutic community treatment: a stage 1 trial. *The American journal of drug and alcohol abuse.* 35(2), 103-108.
149. Marlowe, D. B., Festinger, D. S., Dugosh, K. L., Arabia, P. L., & Kirby, K. C. (2008). An effectiveness trial of contingency management in a felony preadjudication drug court. *Journal of applied behavior analysis*. 41(4), 565-577.
150. Marsh, J. C., D'Aunno, T. A., & Smith, B. D. (2000). Increasing access and providing social services to improve drug abuse treatment for women with children. *Addiction (Abingdon, England).* 95(8), 1237-1247.
151. Marsiglia, F. F., Wu, S., Ayers, S., & Weide, A. (2019). Randomized effectiveness trial of a parent and youth combined intervention on the substance use norms of Latino middle school students. *Journal of substance abuse treatment*, *97*, 75-83.
152. Martino, S., Carroll, K. M., Nich, C., & Rounsaville, B. J. (2006). A randomized controlled pilot study of motivational interviewing for patients with psychotic and drug use disorders. *Addiction (Abingdon, England).* 101(10), 1479-1492.
153. Masson, C. L., Sorensen, J. L., Phibbs, C. S., & Okin, R. L. (2004). Predictors of medical service utilization among individuals with co-occurring HIV infection and substance abuse disorders. *AIDS care*. 16(6), 744-755.
154. McHugo, G. J., & Fallot, R. D. (2011). Multisite randomized trial of behavioral interventions for women with Co-occurring PTSD and substance use disorders. *Journal of Dual Diagnosis*. 7(4), 280-284.
155. Merchant, R. C., Zhang, Z., Zhang, Z., Liu, T., & Baird, J. R. (2018). Lack of efficacy in a randomised trial of a brief intervention to reduce drug use and increase drug treatment services utilisation among adult emergency department patients over a 12-month period. *Emerg Med J*, *35*(5), 282-288.
156. Meshberg-Cohen, S., Svikis, D., & McMahon, T. J. (2014). Expressive writing as a therapeutic process for drug-dependent women. *Substance abuse.* 35(1), 80-88.
157. Meyers, R. J., Miller, W. R., Smith, J. E., & Tonigan, J. S. (2002). A randomized trial of two methods for engaging treatment-refusing drug users through concerned significant others. *Journal of consulting and clinical psychology*. 70(5), 1182-1185.
158. Milby, J. B., Clarke, C., Toro, C., Thornton, S., & Rickert, D. (1980). Effectiveness of urine surveillance as an adjunct to outpatient psychotherapy for drug abusers. *The International journal of the addictions.* 15(7), 993-1001.
159. Miller, W. R., Forcehimes, A., O'Leary, M. J., & LaNoue, M. D. (2008). Spiritual direction in addiction treatment: two clinical trials. *Journal of substance abuse treatment.* 35(4), 434-442.
160. Mills, K. L., Teesson, M., Back, S. E., Brady, K. T., Baker, A. L., Hopwood, S., … & Ewer, P. L. (2012). Integrated exposure-based therapy for co-occurring posttraumatic stress disorder and substance dependence: a randomized controlled trial. *JAMA.* 308(7), 690-699.
161. Mitrani, V. B., Weiss-Laxer, N. S., Ow, C. E., Burns, M. J., Ross-Russell, S., & Feaster, D. J. (2009). Examining family networks of HIV+ women in drug recovery: challenges and opportunities. *Families, systems & health : the journal of collaborative family healthcare.* 27(3), 267-283.
162. Morgenstern, J., Blanchard, K. A., McCrady, B. S., McVeigh, K. H., Morgan, T. J., & Pandina, R. J. (2006). Effectiveness of intensive case management for substance-dependent women receiving temporary assistance for needy families. *American journal of public health*. 96(11), 2016-2023.
163. Morgenstern, J., Bux, Jr, Labouvie, E., Morgan, T., Blanchard, K. A., & Muench, F. (2003). Examining mechanisms of action in 12-Step community outpatient treatment. *Drug and alcohol dependence.* 72(3), 237-247.
164. Morgenstern, J., Blanchard, K. A., Kahler, C., Barbosa, K. M., McCrady, B. S., & McVeigh, K. H. (2008). Testing mechanisms of action for intensive case management. *Addiction (Abingdon, England).* 103(3), 469-477.
165. Morgenstern, J., Hogue, A., Dauber, S., Dasaro, C., & McKay, J. R. (2009). A practical clinical trial of coordinated care management to treat substance use disorders among public assistance beneficiaries. *Journal of consulting and clinical psychology*. 77(2), 257-269.
166. Mueser, K. T., Glynn, S. M., Cather, C., Zarate, R., Fox, L., Feldman, J., … & Clark, R. E. (2009). Family intervention for co-occurring substance use and severe psychiatric disorders: participant characteristics and correlates of initial engagement and more extended exposure in a randomized controlled trial. *Addictive behaviors.* 34(10), 867-877.
167. Mullins, S. M., Suarez, M., Ondersma, S. J., & Page, M. C. (2004). The impact of motivational interviewing on substance abuse treatment retention: a randomized control trial of women involved with child welfare. *Journal of substance abuse treatment.* 27(1), 51-58.
168. Myers, M. G., & Prochaska, J. J. (2008). Does smoking intervention influence adolescent substance use disorder treatment outcomes? *Substance abuse*. 29(2), 81-88.
169. Najavits, L. M., Enggasser, J., Brief, D., & Federman, E. (2018). A randomized controlled trial of a gender‐focused addiction model versus 12‐step facilitation for women veterans. *The American journal on addictions*, *27*(3), 210-216.
170. North, C. S., Pollio, D. E., Sims, O. T., Jain, M. K., & Hong, B. A. (2018). Prospective longitudinal substance use patterns in patients preparing for hepatitis C treatment. *Journal of dual diagnosis*, *14*(1), 60-69.
171. Ober, A. J., Watkins, K. E., McCullough, C. M., Setodji, C. M., Osilla, K., & Hunter, S. B. (2018). Patient predictors of substance use disorder treatment initiation in primary care. *Journal of substance abuse treatment*, *90*, 64-72.
172. O'Connell, M. J., Kasprow, W. J., & Rosenheck, R. A. (2012). Differential impact of supported housing on selected subgroups of homeless veterans with substance abuse histories. *Psychiatric services (Washington, D.C.).* 63(12), 1195-1205.
173. O'Farrell, T. J., Murphy, M., Alter, J., & Fals-Stewart, W. (2007). Brief family treatment intervention to promote aftercare among male substance abusing patients in inpatient detoxification: A quasi-experimental pilot study. *Addictive behaviors.* 32(8), 1681-1691.
174. Ohana, D., Maayan, R., Delayahu, Y., Roska, P., Ponizovsky, A. M., Weizman, A., … & Yechiam, E. (2016). Effect of dehydroepiandrosterone add-on therapy on mood, decision making and subsequent relapse of polydrug users. *Addiction biology.* 21(4), 885-894.
175. Olmstead, T. A., & Petry, N. M. (2009). The cost-effectiveness of prize-based and voucher-based contingency management in a population of cocaine- or opioid-dependent outpatients. *Drug and alcohol dependence*. 102(1-3), 108-115.
176. Olmstead, T. A., Ostrow, C. D., & Carroll, K. M. (2010). Cost-effectiveness of computer-assisted training in cognitive-behavioral therapy as an adjunct to standard care for addiction. *Drug and alcohol dependence*. 110(3), 200-207.
177. Ondersma, S. J., Winhusen, T., & Lewis, D. F. (2012). Pre-treatment change in a randomized trial with pregnant substance-abusing women in community-based outpatient treatment. *Contemporary clinical trials*. 33(5), 1074-1079.
178. Ondersma, S. J., Winhusen, T., Erickson, S. J., Stine, S. M., & Wang, Y. (2009). Motivation Enhancement Therapy with pregnant substance-abusing women: does baseline motivation moderate efficacy? *Drug and alcohol dependence*. 101(1-2), 74-79.
179. Palfai, T. P., Cheng, D. M., Bernstein, J. A., Palmisano, J., Lloyd-Travaglini, C. A., Goodness, T., & Saitz, R. (2016). Is the quality of brief motivational interventions for drug use in primary care associated with subsequent drug use? *Addictive behaviors.* 56, 8-14.
180. Pantalon, M. V., Murphy, M. K., Barry, D. T., Lavery, M., & Swanson, A. J. (2014). Predictors and moderators of aftercare appointment-keeping following brief motivational interviewing among patients with psychiatric disorders or dual diagnosis. *Journal of Dual Diagnosis*. 10(1), 44-51.
181. Paris, M., Silva, M., Añez-Nava, L., Jaramillo, Y., Kiluk, B. D., Gordon, M. A., ... & Carroll, K. M. (2018). Culturally adapted, web-based cognitive behavioral therapy for spanish-speaking individuals with substance use disorders: A randomized clinical trial. *American journal of public health*, *108*(11), 1535-1542.
182. Parthasarathy, S., Mertens, J., Moore, C., & Weisner, C. (2003). Utilization and cost impact of integrating substance abuse treatment and primary care. *Medical Care*. 41(3), 357-367.
183. Penk, W., Drebing, C. E., Rosenheck, R. A., Krebs, C., Van Ormer, A., & Mueller, L. (2010). Veterans Health Administration Transitional work experience vs. job placement in veterans with co-morbid substance use and non-psychotic psychiatric disorders. *Psychiatric rehabilitation journal.* 33(4), 297-307.
184. Petitjean, S. A., Dursteler-Macfarland, K. M., Krokar, M. C., Strasser, J., Mueller, S. E., Degen, B., … & Farronato, N. S. (2014). A randomized, controlled trial of combined cognitive-behavioral therapy plus prize-based contingency management for cocaine dependence. *Drug and alcohol dependence.* 145, 94-100.
185. Petry, N. M., Alessi, S. M., Carroll, K. M., Hanson, T., MacKinnon, S., Rounsaville, B., & Sierra, S. (2006). Contingency management treatments: Reinforcing abstinence versus adherence with goal-related activities. *Journal of consulting and clinical psychology*. 74(3), 592-601.
186. Petry, N. M., Kolodner, K. B., Li, R., Peirce, J. M., Roll, J. M., Stitzer, M. L., & Hamilton, J. A. (2006). Prize-based contingency management does not increase gambling. *Drug and alcohol dependence*. 83(3), 269-273.
187. Petry, N. M., Rash, C. J., & Alessi, S. M. (2016). A randomized controlled trial of brief interventions for problem gambling in substance abuse treatment patients. *Journal of consulting and clinical psychology.* 84(10), 874-886.
188. Philips, B., Wennberg, P., Konradsson, P., & Franck, J. (2018). Mentalization-based treatment for concurrent borderline personality disorder and substance use disorder: A randomized controlled feasibility study. *European addiction research*, *24*(1), 1-8.
189. Pitre, U., Dansereau, D. F., Newbern, D., & Simpson, D. D. (1998). Residential drug abuse treatment for probationers. Use of node-link mapping to enhance participation and progress. *Journal of substance abuse treatment.* 15(6), 535-543.
190. Porter, L. S., Porter, B. O., McCoy, V., Bango-Sanchez, V., Kissel, B., Williams, M., & Nunnewar, S. (2015). Blended Infant Massage-Parenting Enhancement Program on Recovering Substance-Abusing Mothers' Parenting Stress, Self-Esteem, Depression, Maternal Attachment, and Mother-Infant Interaction. *Asian nursing research.* 9(4), 318-327.
191. Price, C. J., Thompson, E. A., Crowell, S., & Pike, K. (2019). Longitudinal effects of interoceptive awareness training through mindful awareness in body-oriented therapy (MABT) as an adjunct to women’s substance use disorder treatment: a randomized controlled trial. *Drug and alcohol dependence.* 198, 140-149.
192. Price, C. J., Wells, E. A., Donovan, D. M., & Rue, T. (2012). Mindful awareness in body-oriented therapy as an adjunct to women's substance use disorder treatment: a pilot feasibility study. *Journal of substance abuse treatment.* 43(1), 94-107.
193. Putnins, S. I., Griffin, M. L., Fitzmaurice, G. M., Dodd, D. R., & Weiss, R. D. (2012). Poor sleep at baseline predicts worse mood outcomes in patients with co-occurring bipolar disorder and substance dependence. *The Journal of clinical psychiatry.* 73(5), 703-708.
194. Raes, V., de Jong, C. A. J., De Bacquer, D., Broekaert, E., & De Maeseneer, J. (2011). The effect of using assessment instruments on substance-abuse outpatients' adherence to treatment: a multi-centre randomised controlled trial. *BMC health services research*. 11, 123.
195. Rafia, R., Dodd, P. J., Brennan, A., Meier, P. S., Hope, V. D., Ncube, F., … & Strang, J. (2016). An economic evaluation of contingency management for completion of hepatitis B vaccination in those on treatment for opiate dependence. *Addiction (Abingdon, England).* 111(9), 1616-1627.
196. Rash, C. J., & Petry, N. M. (2015). Contingency management treatments are equally efficacious for both sexes in intensive outpatient settings. *Experimental and clinical psychopharmacology.* 23(5), 369-376.
197. Rash, C. J., Alessi, S. M., & Petry, N. M. (2017). Substance Abuse Treatment Patients in Housing Programs Respond to Contingency Management Interventions. *Journal of substance abuse treatment*. 72, 97-102.
198. Reback, C. J., Peck, J. A., Fletcher, J. B., Nuno, M., & Dierst-Davies, R. (2012). Lifetime substance use and HIV sexual risk behaviors predict treatment response to contingency management among homeless, substance-dependent MSM. *Journal of psychoactive drugs.* 44(2), 166-172.
199. Reid, M. S., Fallon, B., Sonne, S., Flammino, F., Nunes, E. V., Jiang, H., … & Rotrosen, J. (2008). Smoking cessation treatment in community-based substance abuse rehabilitation programs. *Journal of substance abuse treatment.* 35(1), 68-77.
200. Robabeh, S. M., Jafar, M. M. M., Sharareh, H. M., Maryam, H. R. M., & Masoumeh, E. M. (2015). The Effect of Cognitive Behavior Therapy in Insomnia due to Methadone Maintenance Therapy: A Randomized Clinical Trial. *Iranian journal of medical sciences.* 40(5), 396-403.
201. Robbins, M. S., Feaster, D. J., Horigian, V. E., Rohrbaugh, M., Shoham, V., Bachrach, K., … & Szapocznik, J. (2011). Brief strategic family therapy versus treatment as usual: Results of a multisite randomized trial for substance using adolescents. *Journal of consulting and clinical psychology.* 79(6), 713-727.
202. Robbins, M. S., Szapocznik, J., Dillon, F. R., Turner, C. W., Mitrani, V. B., & Feaster, D. J. (2008). The efficacy of structural ecosystems therapy with drug-abusing/dependent African American and Hispanic American adolescents. *Journal of family psychology: JFP: journal of the Division of Family Psychology of the American Psychological Association (Division 43).* 22(1), 51-61.
203. Robles, R. R., Reyes, J. C., Colon, H. M., Sahai, H., Marrero, C. A., Matos, T. D., … & Shepard, E. W. (2004). Effects of combined counseling and case management to reduce HIV risk behaviors among Hispanic drug injectors in Puerto Rico: a randomized controlled study. *Journal of substance abuse treatment.* 27(2), 145-152.
204. Rohde, P., Turner, C. W., Waldron, H. B., Brody, J. L., & Jorgensen, J. (2018). Depression change profiles in adolescents treated for comorbid depression/substance abuse and profile membership predictors. *Journal of Clinical Child & Adolescent Psychology*. 47(4), 595-607.
205. Roos, C. R., Bowen, S., & Witkiewitz, K. (2017). Baseline patterns of substance use disorder severity and depression and anxiety symptoms moderate the efficacy of mindfulness-based relapse prevention. *Journal of consulting and clinical psychology*. 85(11), 1041-1051.
206. Roos, C. R., Stein, E., Bowen, S., & Witkiewitz, K. (2019). Individual gender and group gender composition as predictors of differential benefit from mindfulness-based relapse prevention for substance use disorders. *Mindfulness*. 10(8), 1560-1567.
207. Rowe, C. L., Liddle, H. A., Greenbaum, P. E., & Henderson, C. E. (2004). Impact of psychiatric comorbidity on treatment of adolescent drug abusers. *Journal of substance abuse treatment.* 26(2), 129-140.
208. Ruglass, L. M., Lopez-Castro, T., Papini, S., Killeen, T., Back, S. E., & Hien, D. A. (2017). Concurrent treatment with prolonged exposure for co-occurring full or subthreshold posttraumatic stress disorder and substance use disorders: A randomized clinical trial. *Psychotherapy and psychosomatics*, *86*(3), 150-161.
209. Rush, B. R., Dennis, M. L., Scott, C. K., Castel, S., & Funk, R. R. (2008). The interaction of co-occurring mental disorders and recovery management checkups on substance abuse treatment participation and recovery. *Evaluation Review*. 32(1), 7-38.
210. Saal, S., Forschner, L., Kemmann, D., Zlatosch, J., & Kallert, T. W. (2016). Is employment-focused case management effective for patients with substance use disorders? Results from a controlled multi-site trial in Germany covering a 2-years-period after inpatient rehabilitation. *BMC psychiatry*. 16, 279.
211. Sacks, J. Y., McKendrick, K., & Hamilton, Z. (2012). A randomized clinical trial of a therapeutic community treatment for female inmates: outcomes at 6 and 12 months after prison release. *Journal of addictive diseases.* 31(3), 258-269.
212. Sadeghi, H., Ebrahimi, L., & Vatandoust, La. (2015). Effectiveness of Hope Therapy Protocol on Depression and Hope in Amphetamine Users. *International journal of high risk behaviors & addiction.* 4(4), e21905.
213. Saleh, S. S., Vaughn, T., Hall, J., Levey, S., Fuortes, L., & Uden-Holmen, T. (2002). Effectiveness of case management in substance abuse treatment. Care management journals: *Journal of case management; The journal of long term home health care.* 3(4), 172-177.
214. Santa Ana, E. J., Carroll, K. M., Anez, L., Paris, Jr, Ball, S. A., Nich, C., … & Martino, S. (2009). Evaluating motivational enhancement therapy adherence and competence among Spanish-speaking therapists. *Drug and alcohol dependence.* 103(1-2), 44-51.
215. Santa Ana, E. J., Wulfert, E., & Nietert, P. J. (2007). Efficacy of group motivational interviewing (GMI) for psychiatric inpatients with chemical dependence. *Journal of consulting and clinical psychology.* 75(5), 816-822.
216. Santisteban, D. A., Mena, M. P., Muir, J., McCabe, B. E., Abalo, C., & Cummings, A. M. (2015). The efficacy of two adolescent substance abuse treatments and the impact of comorbid depression: results of a small randomized controlled trial. *Psychiatric rehabilitation journal.* 38(1), 55-64.
217. Schumm, J. A., O'Farrell, T. J., Murphy, M. M., & Muchowski, P. (2018). Partner violence among drug-abusing women receiving behavioral couples therapy versus individually-based therapy. *Journal of substance abuse treatment*, *92*, 1-10.
218. Schwartz, R. P., Jaffe, J. H., Highfield, D. A., Callaman, J. M., & O'Grady, K. E. (2007). A randomized controlled trial of interim methadone maintenance: 10-Month follow-up*. Drug and alcohol dependence*. 86(1), 30-36.
219. Shorey, R. C., Elmquist, J., Gawrysiak, M. J., Strauss, C., Haynes, E., Anderson, S., & Stuart, G. L. (2017). A randomized controlled trial of a mindfulness and acceptance group therapy for residential substance use patients. *Substance use & misuse*, *52*(11), 1400-1410.
220. Shulman, M., Campbell, A., Pavlicova, M., Hu, M. C., Aharonovich, E., & Nunes, E. V. (2018). Cognitive functioning and treatment outcomes in a randomized controlled trial of internet‐delivered drug and alcohol treatment. *The American journal on addictions*, *27*(6), 509-515.
221. Silverman, M. J. (2016). Effects of a Single Lyric Analysis Intervention on Withdrawal and Craving With Inpatients on a Detoxification Unit: A Cluster-Randomized Effectiveness Study. *Substance use & misuse.* 51(2), 241-249.
222. Sinadinovic, K., Wennberg, P., & Berman, A. H. (2012). Targeting problematic users of illicit drugs with Internet-based screening and brief intervention: a randomized controlled trial. *Drug and alcohol dependence.* 126(1-2), 42-50.
223. Slesnick, N., & Erdem, G. (2013). Efficacy of ecologically-based treatment with substance-abusing homeless mothers: substance use and housing outcomes. *Journal of substance abuse treatment.* 45(5), 416-425.
224. Slesnick, N., Guo, X., Brakenhoff, B., & Bantchevska, D. (2015). A comparison of three interventions for homeless youth evidencing substance use disorders: results of a randomized clinical trial. *Journal of substance abuse treatment.* 54, 1-13.
225. Sloboda, Z., Stephens, R. C., Stephens, P. C., Grey, S. F., Teasdale, B., Hawthorne, R. D., … & Marquette, J. F. (2009). The Adolescent Substance Abuse Prevention Study: A randomized field trial of a universal substance abuse prevention program. *Drug and alcohol dependence.* 102(1-3), 1-10.
226. Smith, D. C., Hall, J. A., Williams, J. K., An, H., & Gotman, N. (2006). Comparative efficacy of family and group treatment for adolescent substance abuse. *The American journal on addictions.* 15 Suppl 1, 131-136.
227. Solomon, S. S., Solomon, S., McFall, A. M., Srikrishnan, A. K., Anand, S., Verma, V., ... & Kumar, M. S. (2019). Integrated HIV testing, prevention, and treatment intervention for key populations in India: a cluster-randomised trial. *The Lancet HIV.* 6(5), e283-e296.
228. Stein, M. D., Herman, D. S., & Anderson, B. J. (2009). A trial to reduce hepatitis C seroincidence in drug users. *Journal of addictive diseases.* 28(4), 389-398.
229. Stitzer, M., Calsyn, D., Matheson, T., Sorensen, J., Gooden, L., & Metsch, L. (2017). Development of a Multi-Target Contingency Management Intervention for HIV Positive Substance Users. *Journal of substance abuse treatment.* 72, 66-71.
230. Stitzer, M. L., Gukasyan, N., Matheson, T., Sorensen, J. L., Feaster, D. J., Duan, R., ... & Metsch, L. R. (2019). Enhancing patient navigation with contingent financial incentives for substance use abatement in persons with HIV and substance use. Psychology of Addictive Behaviors. 34, 23-30.
231. Stitzer, M. L., Petry, N. M., & Peirce, J. (2010). Motivational incentives research in the National Drug Abuse Treatment Clinical Trials Network. *Journal of substance abuse treatment.* 38 Suppl 1, S61-S69.
232. Stover, C. S. (2015). Fathers for Change for Substance Use and Intimate Partner Violence: Initial Community Pilot. *Family process.* 54(4), 600-609.
233. Sugarman, D. E., Nich, C., & Carroll, K. M. (2010). Coping strategy use following computerized cognitive-behavioral therapy for substance use disorders. *Psychology of addictive behaviors: journal of the Society of Psychologists in Addictive Behaviors.* 24(4), 689-695.
234. Svikis, D. S., Lee, J. H., Haug, N. A., & Stitzer, M. L. (1997). Attendance incentives for outpatient treatment: effects in methadone- and nonmethadone-maintained pregnant drug dependent women. *Drug and alcohol dependence.* 48(1), 33-41.
235. Svikis, D. S., Keyser-Marcus, L., Stitzer, M., Rieckmann, T., Safford, L., Loeb, P., … & Zweben, J. (2012). Randomized multi-site trial of the Job Seekers' Workshop in patients with substance use disorders. *Drug and alcohol dependence.* 120(1-3), 55-64.
236. Tait, R. J., Hulse, G. K., Robertson, S. I., & Sprivulis, P. C. (2005). Emergency department-based intervention with adolescent substance users: 12-month outcomes. *Drug and alcohol dependence.* 79(3), 359-363.
237. Tantirangsee, N., Assanangkornchai, S., & Marsden, J. (2015). Effects of a brief intervention for substance use on tobacco smoking and family relationship functioning in schizophrenia and related psychoses: a randomised controlled trial. *Journal of substance abuse treatment.* 51, 30-37.
238. Tate, S. R., Mrnak-Meyer, J., Shriver, C. L., Atkinson, J. H., Robinson, S. K., & Brown, S. A. (2011). Predictors of treatment retention for substance-dependent adults with co-occurring depression. *American Journal on Addictions.* 20(4), 357-365.
239. Thornton, C. C., Gottheil, E., Weinstein, S. P., & Kerachsky, R. S. (1998). Patient-treatment matching in substance abuse. Drug addiction severity. *Journal of substance abuse treatment.* 15(6), 505-511.
240. Thornton, C., Gottheil, E., Patkar, A., & Weinstein, S. (2003). Coping styles and response to high versus low-structure individual counseling for substance abuse. The *American journal on addictions.* 12(1), 29-42.
241. Thurstone, C., Riggs, P. D., Salomonsen-Sautel, S., & Mikulich-Gilbertson, S. K. (2010). Randomized, controlled trial of atomoxetine for attention-deficit/hyperactivity disorder in adolescents with substance use disorder. *Journal of the American Academy of Child and Adolescent Psychiatry.* 49(6), 573-582.
242. Thylstrup, B., Schroder, S., & Hesse, M. (2015). Psycho-education for substance use and antisocial personality disorder: A randomized trial. *BMC psychiatry.* 15(1), 283.
243. Thylstrup, B., Schroder, S., Fridell, M., & Hesse, M. (2017). Did you get any help? A post-hoc secondary analysis of a randomized controlled trial of psychoeducation for patients with antisocial personality disorder in outpatient substance abuse treatment programs. *BMC psychiatry.* 17(1), 7.
244. Thylstrup, B., & Hesse, M. (2016). Impulsive lifestyle counseling to prevent dropout from treatment for substance use disorders in people with antisocial personality disorder: A randomized study. *Addictive behaviors.* 57, 48-54.
245. Timko, C., & DeBenedetti, A. (2007). A randomized controlled trial of intensive referral to 12-step self-help groups: one-year outcomes. *Drug and alcohol dependence.* 90(2-3), 270-279.
246. Timko, C., DeBenedetti, A., & Billow, R. (2006). Intensive referral to 12-Step self-help groups and 6-month substance use disorder outcomes. *Addiction (Abingdon, England).* 101(5), 678-688.
247. Timko, C., Harris, A. H., Jannausch, M., & Ilgen, M. (2019). Randomized controlled trial of telephone monitoring with psychiatry inpatients with co-occurring substance use and mental health disorders. *Drug and alcohol dependence.* 194, 230-237.
248. Tofighi, B., Campbell, A. N. C., Pavlicova, M., Hu, M. C., Lee, J. D., & Nunes, E. V. (2016). Recent internet use and associations with clinical outcomes among patients entering addiction treatment involved in a web-delivered psychosocial intervention study. *Journal of Urban Health*, *93*(5), 871-883.
249. Tracy, K., Burton, M., Nich, C., & Rounsaville, B. (2011). Utilizing peer mentorship to engage high recidivism substance-abusing patients in treatment. *The American journal of drug and alcohol abuse.* 37(6), 525-531.
250. Tuten, M., Fitzsimons, H., Chisolm, M. S., Nuzzo, P. A., & Jones, H. E. (2012). Contingent incentives reduce cigarette smoking among pregnant, methadone-maintained women: results of an initial feasibility and efficacy randomized clinical trial. *Addiction (Abingdon, England).* 107(10), 1868-1877.
251. Vaezazizi, L. M., Campbell, A. N., Pavlicova, M., Hu, M. C., & Nunes, E. V. (2019). Understanding site variability in a multisite clinical trial of a technology-delivered psychosocial intervention for substance use disorders. *Journal of substance abuse treatment.* 105, 64-70.
252. Vaughan Sarrazin, M. S., & Hall, J. A. (2004). Impact of Iowa case management on provisions of social support for substance abuse clients*. Care Management Journals.* 5(1), 3-11.
253. Vederhus, J. K., Timko, C., Kristensen, O., Hjemdahl, B., & Clausen, T. (2014). Motivational intervention to enhance post-detoxification 12-Step group affiliation: a randomized controlled trial. *Addiction (Abingdon, England).* 109(5), 766-773.
254. Vujanovic, A. A., Smith, L. J., Green, C. E., Lane, S. D., & Schmitz, J. M. (2018). Development of a novel, integrated cognitive-behavioral therapy for co-occurring posttraumatic stress and substance use disorders: A pilot randomized clinical trial. *Contemporary clinical trials*, *65*, 123-129.
255. Warden, D., Riggs, P. D., Min, S.-J., Mikulich-Gilbertson, S. K., Tamm, L., Trello-Rishel, K., & Winhusen, T. (2012). Major depression and treatment response in adolescents with ADHD and substance use disorder. *Drug and alcohol dependence.* 120(1-3), 214-219.
256. Walitzer, K. S., Dermen, K. H., Barrick, C., & Shyhalla, K. (2015). Modeling the innovation–decision process: dissemination and adoption of a motivational interviewing preparatory procedure in addiction outpatient clinics. *Journal of substance abuse treatment*, *57*, 18-29.
257. Washington, O. G. (2001). Using brief therapeutic interventions to create change in self-efficacy and personal control of chemically dependent women. *Archives of psychiatric nursing.* 15(1), 32-40.
258. Watkins, K. E., Ober, A. J., Lamp, K., Lind, M., Setodji, C., Osilla, K. C., … & Pincus, H. A. (2017). Collaborative care for opioid and alcohol use disorders in primary care: The SUMMIT randomized clinical trial. *JAMA internal medicine.* 177(10), 1480-1488.
259. Watson, J., Toner, P., Day, D. B., Brady, L. M., Fairhurst, C., Renwick, C., ... & Cocks, K. (2017). Youth social behaviour and network therapy (Y-SBNT): adaptation of a family and social network intervention for young people who misuse alcohol and drugs-a randomised controlled feasibility trial. *Health technology assessment (Winchester, England)*, *21*(15), 1.
260. Webster, J. M., Staton-Tindall, M., Dickson, M. F., Wilson, J. F., & Leukefeld, C. G. (2014). Twelve-month employment intervention outcomes for drug-involved offenders. *The American journal of drug and alcohol abuse.* 40(3), 200-205.
261. Weinstock, J., Alessi, S. M., & Petry, N. M. (2007). Regardless of psychiatric severity the addition of contingency management to standard treatment improves retention and drug use outcomes. *Drug and alcohol dependence.* 87(2-3), 288-296.
262. Weiss, R. D., Griffin, M. L., Kolodziej, M. E., Greenfield, S. F., Najavits, L. M., Daley, D. C., … & Hennen, J. A. (2007). A randomized trial of integrated group therapy versus group drug counseling for patients with bipolar disorder and substance dependence. *American Journal of Psychiatry.* 164(1), 100-107.
263. Welsh, W. N., Knudsen, H. K., Knight, K., Ducharme, L., Pankow, J., Urbine, T., … & Friedmann, P. D. (2016). Effects of an Organizational Linkage Intervention on Inter-Organizational Service Coordination Between Probation/Parole Agencies and Community Treatment Providers. *Administration and policy in mental health.* 43(1), 105-121.
264. Winhusen, T., Kropp, F., Babcock, D., Hague, D., Erickson, S. J., Renz, C., … & Somoza, E. (2008). Motivational enhancement therapy to improve treatment utilization and outcome in pregnant substance users. *Journal of substance abuse treatment.* 35(2), 161-173.
265. Winters, J., Fals-Stewart, W., O'Farrell, T. J., Birchler, G. R., & Kelley, M. L. (2002). Behavioral couples therapy for female substance-abusing patients: Effects on substance use and relationship adjustment. *Journal of Consulting and Clinical Psychology, 70*(2), 344-355.
266. Witkiewitz, K., Warner, K., Sully, B., Barricks, A., Stauffer, C., Thompson, B. L., & Luoma, J. B. (2014). Randomized trial comparing mindfulness-based relapse prevention with relapse prevention for women offenders at a residential addiction treatment center. *Substance use & misuse.* 49(5), 536-546.
267. Witkiewitz, K., & Bowen, S. (2010). Depression, craving, and substance use following a randomized trial of mindfulness-based relapse prevention. *Journal of consulting and clinical psychology.* 78(3), 362-374.
268. Wolitzky-Taylor, K., Krull, J., Rawson, R., Roy-Byrne, P., Ries, R., & Craske, M. G. (2018). Randomized clinical trial evaluating the preliminary effectiveness of an integrated anxiety disorder treatment in substance use disorder specialty clinics. *Journal of consulting and clinical psychology*, *86*(1), 81.
269. Wolitzky-Taylor, K., Niles, A. N., Ries, R., Krull, J. L., Rawson, R., Roy-Byrne, P., & Craske, M. (2018). Who needs more than standard care? Treatment moderators in a randomized clinical trial comparing addiction treatment alone to addiction treatment plus anxiety disorder treatment for comorbid anxiety and substance use disorders. *Behaviour research and therapy*, *107*, 1-9.
270. Woodruff, S. I., Clapp, J. D., Eisenberg, K., McCabe, C., Hohman, M., Shillington, A. M., … & Gareri, J. (2014). Randomized clinical trial of the effects of screening and brief intervention for illicit drug use: the Life Shift/Shift Gears study. *Addiction science & clinical practice.* 9, 8.
271. Worley, M. J., Trim, R. S., Roesch, S. C., Mrnak-Meyer, J., Tate, S. R., & Brown, S. A. (2012). Comorbid depression and substance use disorder: longitudinal associations between symptoms in a controlled trial. *Journal of substance abuse treatment.* 43(3), 291-302.
272. Wusthoff, L. E., Waal, H., & Grawe, R. W. (2014). The effectiveness of integrated treatment in patients with substance use disorders co-occurring with anxiety and/or depression--a group randomized trial. *BMC psychiatry*. 14, 67.
273. Xu, X., Yonkers, K. A., & Ruger, J. P. (2017). Economic evaluation of a behavioral intervention versus brief advice for substance use treatment in pregnant women: Results from a randomized controlled trial. *BMC pregnancy and childbirth*. 17(1), 83.
274. Zhang, J., & Slesnick, N. (2018). Substance use and social stability of homeless youth: A comparison of three interventions. *Psychology of Addictive Behaviors*, *32*(8), 873.
275. Ziaee, S. S., Fadardi, J. S., Cox, W. M., & Yazdi, S. A. A. (2016). Effects of attention control training on drug abusers' attentional bias and treatment outcome. *Journal of consulting and clinical psychology*. 84(10), 861-873.
276. Zimmermann, G., Riere, J., Favrat, B., Krenz, S., Besson, J., & Zullino, D. F. (2006). Additional effect of hypnosis in an in-patient detoxification program: Results of a pilot clinical trial. German *Journal of Psychiatry*. 9(1), 22-26.
277. Zlotnick, C., Johnson, J., & Najavits, L. M. (2009). Randomized Controlled Pilot Study of Cognitive-Behavioral Therapy in a Sample of Incarcerated Women With Substance Use Disorder and PTSD. *Behavior Therapy*. 40(4), 325-336.

**Individuals Not on Opioid Agonist Therapy (n=146)**

1. Ahmadi, J., Jahromi, M. S., & Ehsaei, Z. (2018). The effectiveness of different singly administered high doses of buprenorphine in reducing suicidal ideation in acutely depressed people with co-morbid opiate dependence: a randomized, double-blind, clinical trial. *Trials*, *19*(1), 462.
2. Ahmadi J, Jahromi MS, Ghahremani D, London ED. Single high-dose buprenorphine for opioid craving during withdrawal. *Trials [Electronic Resource]*. 2018/12/10/ 19(1), 675, 2018.
3. Ahmadpanah, M., Mirzaei Alavijeh, M., Allahverdipour, H., Jalilian, F., Haghighi, M., Afsar, A., & Gharibnavaz, H. (2013). Effectiveness of Coping Skills Education Program to Reduce Craving Beliefs among Addicts Referred To Addiction Centers in Hamadan: A Randomized Controlled Trial. *Iranian journal of public health.* 42(10), 1139-1144.
4. Alessi, S. M., Hanson, T., Wieners, M., & Petry, N. M. (2007). Low-cost contingency management in community clinics: delivering incentives partially in group therapy. *Experimental and clinical psychopharmacology.* 15(3), 293-300.
5. Azkhosh, M., Farhoudianm, A., Saadati, H., Shoaee, F., & Lashani, L. (2016). Comparing Acceptance and Commitment Group Therapy and 12-Steps Narcotics Anonymous in Addict's Rehabilitation Process: A Randomized Controlled Trial. Iran*ian journal of psychiatry*. 11(4), 244-249.
6. Bale, R. N., Van Stone, W. W., Kuldau, J. M., Engelsing, T. M., & Zarcone, V. P. (1973). Methadone treatment versus therapeutic communities: preliminary results of a randomized study in progress. *Proceedings.National Conference on Methadone Treatment.* 2, 1027-1034.
7. Ball, S. A., Nich, C., Rounsaville, B. J., Eagan, D., & Carroll, K. M. (2004). Millon Clinical Multiaxial Inventory-III subtypes of opioid dependence: validity and matching to behavioral therapies. *Journal of consulting and clinical psychology*. 72(4), 698-711.
8. Banta-Green, C. J., Coffin, P. O., Merrill, J. O., Sears, J. M., Dunn, C., Floyd, A. S., … & Donovan, D. M. (2018). Impacts of an opioid overdose prevention intervention delivered subsequent to acute care. *Injury prevention.* 0, 1-8.
9. Barnett, P. G., Masson, C. L., Sorensen, J. L., Wong, W., & Hall, S. (2006). Linking opioid-dependent hospital patients to drug treatment: Health care use and costs 6 months after randomization. *Addiction (Abingdon, England).* 101(12), 1797-1804.
10. Beattie, A., Marques, E. M., Barber, M., Greenwood, R., Ingram, J., Ayres, R., ... & Hickman, M. (2015). Script in a Day intervention for individuals who are injecting opioids: a feasibility randomized control trial. *Journal of Public Health*, *38*(4), 712-721.
11. Bernstein, E., Ashong, D., Heeren, T., Winter, M., Bliss, C., Madico, G., & Bernstein, J. (2012). The impact of a brief motivational intervention on unprotected sex and sex while high among drug-positive emergency department patients who receive STI/HIV VC/T and drug treatment referral as standard of care. *AIDS and behavior*. 16(5), 1203-1216.
12. Bernstein, J., Bernstein, E., Tassiopoulos, K., Heeren, T., Levenson, S., & Hingson, R. (2005). Brief motivational intervention at a clinic visit reduces cocaine and heroin use. *Drug and alcohol dependence.* 77(1), 49-59.
13. Bickel, W. K., Amass, L., Higgins, S. T., Badger, G. J., & Esch, R. A. (1997). Effects of adding behavioral treatment to opioid detoxification with buprenorphine. *Journal of consulting and clinical psychology*. 65(5), 803-810.
14. Bisaga, A., Mannelli, P., Yu, M., Nangia, N., Graham, C. E., Tompkins, D. A., ... & Sullivan, M. A. (2018). Outpatient transition to extended-release injectable naltrexone for patients with opioid use disorder: A phase 3 randomized trial. *Drug and alcohol dependence*, *187*, 171-178.
15. Booth, R. E., Corsi, K. F., & Mikulich, S. K. (2003). Improving entry to methadone maintenance among out-of-treatment injection drug users. *Journal of substance abuse treatment*. 24(4), 305-311.
16. Booth, R. E., Crowley, T. J., & Zhang, Y. (1996). Substance abuse treatment entry, retention and effectiveness: out-of-treatment opiate injection drug users. *Drug and alcohol dependence*. 42(1), 11-20.
17. Brigham, G. S., Slesnick, N., Winhusen, T. M., Lewis, D. F., Guo, X., & Somoza, E. (2014). A randomized pilot clinical trial to evaluate the efficacy of Community Reinforcement and Family Training for Treatment Retention (CRAFT-T) for improving outcomes for patients completing opioid detoxification. *Drug and alcohol dependence*. 138, 240-243.
18. Brinkley-Rubinstein, L., McKenzie, M., Macmadu, A., Larney, S., Zaller, N., Dauria, E., & Rich, J. (2018). A randomized, open label trial of methadone continuation versus forced withdrawal in a combined US prison and jail: Findings at 12 months post-release. *Drug and alcohol dependence*, *184*, 57-63.
19. Brooks, A. C., Comer, S. D., Sullivan, M. A., Bisaga, A., Carpenter, K. M., Raby, W. M., … & Nunes, E. V. (2010). Long-acting injectable versus oral naltrexone maintenance therapy with psychosocial intervention for heroin dependence: a quasi-experiment. *The Journal of clinical psychiatry*. 71(10), 1371-1378.
20. Brooner, R. K., Kidorf, M. S., King, V. L., Stoller, K. B., Peirce, J. M., Bigelow, G. E., & Kolodner, K. (2004). Behavioral contingencies improve counseling attendance in an adaptive treatment model. *Journal of Substance Abuse Treatment*, *27*(3), 223-232.
21. Calsyn, D. A., Campbell, A. N. C., Crits-Christoph, P., Doyle, S. R., Tross, S., Hatch-Maillette, M. A., & Mandler, R. (2010). Men in methadone maintenance versus psychosocial outpatient treatment: differences in sexual risk behaviors and intervention effectiveness from a multisite HIV prevention intervention trial. *Journal of addictive diseases*. 29(3), 370-382.
22. Carpenter, K. M., Jiang, H., Sullivan, M. A., Bisaga, A., Comer, S. D., Raby, W. N., … & Nunes, E. V. (2009). Betting on Change: Modeling Transitional Probabilities to Guide Therapy Development for Opioid Dependence. *Psychology of Addictive Behaviors*. 23(1), 47-55.
23. Carroll, K. M., Ball, S. A., Nich, C., O'Connor, P. G., Eagan, D. A., Frankforter, … & Rounsaville, B. J. (2001). Targeting behavioral therapies to enhance naltrexone treatment of opioid dependence: efficacy of contingency management and significant other involvement. *Archives of general psychiatry*. 58(8), 755-761.
24. Carroll, K. M., Sinha, R., Nich, C., Babuscio, T., & Rounsaville, B. J. (2002). Contingency management to enhance naltrexone treatment of opioid dependence: a randomized clinical trial of reinforcement magnitude. *Experimental and clinical psychopharmacology*. 10(1), 54-63.
25. Chen, J. Y., Yu, J. C., Cao, J. P., Xiao, Y., Gu, H., Zhong, R. L., ... & Wang, Z. Z. (2019). Abstinence Following a Motivation-Skill-Desensitization-Mental Energy Intervention for Heroin Dependence: A Three-year Follow-up Result of a Randomized Controlled Trial. *Current Medical Science.* 39(3), 472-482.
26. Cochran, G., Stitzer, M., Campbell, A. N. C., Hu, M. C., Vandrey, R., & Nunes, E. V. (2015). Web-based treatment for substance use disorders: differential effects by primary substance. *Addictive behaviors*. 45, 191-194.
27. Coffin, P. O., Santos, G. M., Matheson, T., Behar, E., Rowe, C., Rubin, T., … & Vittinghoff, E. (2017). Behavioral intervention to reduce opioid overdose among high-risk persons with opioid use disorder: A pilot randomized controlled trial. *PloS one.* 12(10), e0183354.
28. Collins, E. D., Kleber, H. D., Whittington, R. A., & Heitler, N. E. (2005). Anesthesia-assisted vs buprenorphine- or clonidine-assisted heroin detoxification and naltrexone induction: a randomized trial. *JAMA*. 294(8), 903-913.
29. Corrigan, J. D., & Bogner, J. (2007). Interventions to promote retention in substance abuse treatment. *Brain injury*. 21(4), 343-356.
30. Coviello, D. M., Cornish, J. W., Lynch, K. G., Alterman, A. I., & O'Brien, C. P. (2010). A randomized trial of oral naltrexone for treating opioid-dependent offenders. *The American journal on addictions*. 19(5), 422-432.
31. Dawe, S., Powell, J., Richards, D., Gossop, M., Marks, I., Strang, J., & Gray, J. A. (1993). Does post-withdrawal cue exposure improve outcome in opiate addiction? A controlled trial. *Addiction (Abingdon, England).* 88(9), 1233-1245.
32. de Quiros Aragon, M. B., Labrador, F. J., & de Arce, F. (2005). Evaluation of a group cue-exposure treatment for opiate addicts. *The Spanish journal of psychology.* 8(2), 229-237.
33. DeFulio, A., Everly, J. J., Leoutsakos, J. M., Umbricht, A., Fingerhood, M., Bigelow, G. E., & Silverman, K. (2012). Employment-based reinforcement of adherence to an FDA approved extended release formulation of naltrexone in opioid-dependent adults: a randomized controlled trial. *Drug and alcohol dependence*. 120(1-3), 48-54.
34. Des Jarlais, D. C., Casriel, C., Friedman, S. R., & Rosenblum, A. (1992). AIDS and the transition to illicit drug injection--results of a randomized trial prevention program. *British journal of addiction.* 87(3), 493-498.
35. Du, J., Fan, C., Jiang, H., Sun, H., Li, X., & Zhao, M. (2014). Biofeedback combined with cue-exposure as a treatment for heroin addicts. *Physiology & behavior.* 130, 34-39.
36. Dunn, K. E., DeFulio, A., Everly, J. J., Donlin, W. D., Aklin, W. M., Nuzzo, P. A., … & Silverman, K. (2013). Employment-based reinforcement of adherence to oral naltrexone treatment in unemployed injection drug users. *Experimental and clinical psychopharmacology.* 21(1), 74-83.
37. Dunn, K. E., Fingerhood, M., Wong, C. J., Svikis, D. S., Nuzzo, P., & Silverman, K. (2014). Employment-based abstinence reinforcement following inpatient detoxification in HIV-positive opioid and/or cocaine-dependent patients. *Experimental and clinical psychopharmacology.* 22(1), 75-85.
38. Dunn, K. E., Saulsgiver, K. A., Patrick, M. E., Heil, S. H., Higgins, S. T., & Sigmon, S. C. (2013). Characterizing and improving HIV and hepatitis knowledge among primary prescription opioid abusers. *Drug and alcohol dependence*. 133(2), 625-632.
39. Dunn, K. E., Yepez-Laubach, C., Nuzzo, P. A., Fingerhood, M., Kelly, A., Berman, S., & Bigelow, G. E. (2017). Randomized controlled trial of a computerized opioid overdose education intervention. *Drug and alcohol dependence.* 173 Suppl 1, S39-S47.
40. Dunn, K., DeFulio, A., Everly, J. J., Donlin, W. D., Aklin, W. M., Nuzzo, P. A., … & Silverman, K. (2015). Employment-based reinforcement of adherence to oral naltrexone in unemployed injection drug users: 12-month outcomes. *Psychology of addictive behaviors : journal of the Society of Psychologists in Addictive Behaviors.* 29(2), 270-276.
41. Everly, J. J., DeFulio, A., Koffarnus, M. N., Leoutsakos, J. M., Donlin, W. D., Aklin, W. M., … & Silverman, K. (2011). Employment-based reinforcement of adherence to depot naltrexone in unemployed opioid-dependent adults: a randomized controlled trial. *Addiction (Abingdon, England)*. 106(7), 1309-1318.
42. Fahmy, R., Wasfi, M., Mamdouh, R., Moussa, K., Wahba, A., Wittemann, M., … & Wolf, R. C. (2018). Mindfulness-based interventions modulate structural network strength in patients with opioid dependence. *Addictive behaviors*. 82, 50-56.
43. Fals-Stewart, W., & O'Farrell, T. J. (2003). Behavioral family counseling and naltrexone for male opioid-dependent patients. *Journal of consulting and clinical psychology*. 71(3), 432-442.
44. Flemmen, G., Unhjem, R., & Wang, E. (2014). High-intensity interval training in patients with substance use disorder. *BioMed research international*. 2014, 616935.
45. Galanter, M., Dermatis, H., Glickman, L., Maslansky, R., Sellers, M. B., Neumann, E., & Rahman-Dujarric, C. (2004). Network therapy: decreased secondary opioid use during buprenorphine maintenance. *Journal of Substance Abuse Treatment*, *26*(4), 313-318.
46. Garland, E. L., Froeliger, B., & Howard, M. O. (2015). Neurophysiological evidence for remediation of reward processing deficits in chronic pain and opioid misuse following treatment with Mindfulness-Oriented Recovery Enhancement: exploratory ERP findings from a pilot RCT. *Journal of behavioral medicine*. 38(2), 327-336.
47. Gilbert, L., Hunt, T., Primbetova, S., Terlikbayeva, A., Chang, M., Wu, E., … & El-Bassel, N. (2018). Reducing opioid overdose in Kazakhstan: A randomized controlled trial of a couple-based integrated HIV/HCV and overdose prevention intervention "Renaissance". *The International journal on drug policy*. 54, 105-113.
48. Gilchrist, G., Swan, D., Shaw, A., Keding, A., Towers, S., Craine, N., … & Watson, J. (2017). Preventing blood-borne virus infection in people who inject drugs in the UK: systematic review, stakeholder interviews, psychosocial intervention development and feasibility randomised controlled trial. *Health technology assessment (Winchester, England).* 21(72), 1-312.
49. Goldstein, M. F., Deren, S., Kang, S. Y., Des Jarlais, D. C., & Magura, S. (2002). Evaluation of an alternative program for MMTP drop-outs: impact on treatment re-entry. *Drug and alcohol dependence*. 66(2), 181-187.
50. Gordon, M. S., Blue, T. R., Couvillion, K., Schwartz, R. P., O’Grady, K. E., Fitzgerald, T. T., & Vocci, F. J. (2018). Initiating buprenorphine treatment prior to versus after release from prison: Arrest outcomes. *Drug and alcohol dependence*, *188*, 232-238.
51. Griffin, M. L., McDermott, K. A., McHugh, R. K., Fitzmaurice, G. M., Jamison, R. N., & Weiss, R. D. (2016). Longitudinal association between pain severity and subsequent opioid use in prescription opioid dependent patients with chronic pain. *Drug and alcohol dependence.* 163, 216-221.
52. Gruber, K., Chutuape, M. A., & Stitzer, M. L. (2000). Reinforcement-based intensive outpatient treatment for inner city opiate abusers: a short-term evaluation. *Drug and alcohol dependence*. 57(3), 211-223.
53. Haro, G., Ramirez, N., Lopez, N., Barea, J., Mateu, C., & Cervera, G. (2006). Effectiveness of a step-stage psychotherapeutic approach between hospital detoxification and outpatient treatment of drug dependencies. *Addictive Disorders and their Treatment*. 5(2), 87-98.
54. Higgins, S. T., Stitzer, M. L., Bigelow, G. E., & Liebson, I. A. (1986). Contingent methadone delivery: effects on illicit-opiate use. *Drug and alcohol dependence*. 17(4), 311-322.
55. Holtyn, A. F., Koffarnus, M. N., DeFulio, A., Sigurdsson, S. O., Strain, E. C., Schwartz, R. P., … & Silverman, K. (2014). The therapeutic workplace to promote treatment engagement and drug abstinence in out-of-treatment injection drug users: a randomized controlled trial. *Preventive medicine*. 68, 62-70.
56. Hser, Y. I., Fu, L., Wu, F., Du, J., & Zhao, M. (2013). Pilot trial of a recovery management intervention for heroin addicts released from compulsory rehabilitation in China. *Journal of substance abuse treatment*. 44(1), 78-83.
57. Iguchi, M. Y., Stitzer, M. L., Bigelow, G. E., & Liebson, I. A. (1988). Contingency management in methadone maintenance: Effects of reinforcing and aversive consequences on illicit polydrug use. *Drug & Alcohol Dependence*, *22*(1), 1-7.
58. Ives, W. R., Lounsbury, J. W., & Tornatzky, L. G. (1976). An experimental comparison of two community-based drug abuse treatment programs. *Journal of community psychology*. 4(3), 253-258.
59. Jaiswal, R., & Gupta, S. (2018, February). Evaluating motivational enhancement therapy versus life skill training along with pharmacotherapy in management of opioid use disorder. In *Indian Journal Of Psychiatry* (Vol. 60, No. 5, Pp. 155-156). B-9, Kanara Business Centre, Off Link Rd, Ghaktopar-E, Mumbai, 400075, India: Medknow Publications & Media Pvt Ltd.
60. Jenaabadi, H., & Jahangir, A. H. (2017). Comparing the effectiveness of mindfulness-based group therapy and methadone maintenance therapy on psychological symptoms (obsession, interpersonal sensitivity, depression, anxiety, and aggression) among opioid-dependent patients. *Shiraz E* *Medical Journal*. 18(6), e45224.
61. Jones, H. E., Tuten, M., & O'Grady, K. E. (2011). Treating the partners of opioid-dependent pregnant patients: feasibility and efficacy. *The American journal of drug and alcohol abuse.* 37(3), 170-178.
62. Jones, H. E., Wong, C. J., Tuten, M., & Stitzer, M. L. (2005). Reinforcement-based therapy: 12-month evaluation of an outpatient drug-free treatment for heroin abusers. *Drug and alcohol dependence*. 79(2), 119-128.
63. Katz, E. C., Brown, B. S., Schwartz, R. P., O'Grady, K. E., King, S. D., & Gandhi, D. (2011). Transitioning opioid-dependent patients from detoxification to long-term treatment: efficacy of intensive role induction. *Drug and alcohol dependence*. 117(1), 24-30.
64. Katz, E. C., Brown, B. S., Schwartz, R. P., Weintraub, E., Barksdale, W., & Robinson, R. (2004). Role induction: a method for enhancing early retention in outpatient drug-free treatment. *Journal of consulting and clinical psychology*. 72(2), 227-234.
65. Katz, E. C., Chutuape, M. A., Jones, H. E., & Stitzer, M. L. (2002). Voucher reinforcement for heroin and cocaine abstinence in an outpatient drug-free program. *Experimental and clinical psychopharmacology*. 10(2), 136-143.
66. Katz, E. C., Chutuape, M. A., Jones, H., Jasinski, D., Fingerhood, M., & Stitzer, M. (2004). Abstinence incentive effects in a short-term outpatient detoxification program. *Experimental and clinical psychopharmacology*. 12(4), 262-268.
67. Kidorf, M., & Stitzer, M. L. (1996). Contingent use of take-homes and split-dosing to reduce illicit drug use of methadone patients. *Behavior Therapy*, *27*(1), 41-51.
68. Kidorf, M., Disney, E., King, V., Kolodner, K., Beilenson, P., & Brooner, R. K. (2005). Challenges in motivating treatment enrollment in community syringe exchange participants. *Journal of urban health: bulletin of the New York Academy of Medicine*. 82(3), 456-467.
69. Kidorf, M., King, V. L., Gandotra, N., Kolodner, K., & Brooner, R. K. (2012). Improving treatment enrollment and re-enrollment rates of syringe exchangers: 12-month outcomes. *Drug and alcohol dependence*. 124(1-2), 162-166.
70. Kidorf, M., King, V. L., Neufeld, K., Peirce, J., Kolodner, K., & Brooner, R. K. (2009). Improving substance abuse treatment enrollment in community syringe exchangers. *Addiction (Abingdon, England*). 104(5), 786-795.
71. Kinlock, T. W., Gordon, M. S., Schwartz, R. P., O'Grady, K., Fitzgerald, T. T., & Wilson, M. (2007). A randomized clinical trial of methadone maintenance for prisoners: results at 1-month post-release. *Drug and alcohol dependence*. 91(2-3), 220-227.
72. Kirtadze, I., Otiashvili, D., O'Grady, K. E., & Jones, H. E. (2012). Behavioral treatment + naltrexone reduces drug use and legal problems in the Republic of Georgia. The *American journal of drug and alcohol abuse*. 38(2), 171-175.
73. Krook, A. L., Brors, O., Dahlberg, J., Grouff, K., Magnus, P., Roysamb, E., & Waal, H. (2002). A placebo-controlled study of high dose buprenorphine in opiate dependents waiting for medication-assisted rehabilitation in Oslo, Norway. *Addiction (Abingdon, England*). 97(5), 533-542.
74. Krupitsky, E., Bisaga, A., Nangia, N., Sullivan, M., Akerman, S., Silverman, B., & Nunes, E. (2018, June). Extended-Release Naltrexone Decreases Opioid Craving Across the 4-Week Dosing Interval. In *American Journal On Addictions* (Vol. 27, No. 4, pp. 324-325). USA: WILEY.
75. Krupitsky, E. M., Burakov, A. M., Dunaevsky, I. V., Romanova, T. N., Slavina, T. Y., & Grinenko, A. Y. (2007). Single versus repeated sessions of ketamine-assisted psychotherapy for people with heroin dependence. *Journal of psychoactive drugs*. 39(1), 13-19.
76. Krupitsky, E. M., Zvartau, E. E., Masalov, D. V., Tsoi, M. V., Burakov, A. M., Egorova, V. Y., … & Woody, G. E. (2004). Naltrexone for heroin dependence treatment in St. Petersburg, Russia. *Journal of substance abuse treatment*. 26(4), 285-294.
77. Krupitsky, E., Nunes, E. V., Ling, W., Illeperuma, A., Gastfriend, D. R., & Silverman, B. L. (2011). Injectable extended-release naltrexone for opioid dependence: a double-blind, placebo-controlled, multicentre randomised trial. *Lancet.* 377(9776), 1506-1513.
78. Kwiatkowski, C. F., Booth, R. E., & Lloyd, L. V. (2000). The effects of offering free treatment to street-recruited opioid injectors. *Addiction (Abingdon, England)*. 95(5), 697-704.
79. Ledgerwood, D. M., & Petry, N. M. (2006). Does contingency management affect motivation to change substance use? *Drug and alcohol dependence*. 83(1), 65-72.
80. Lee, J. D., Friedmann, P. D., Kinlock, T. W., Nunes, E. V., Boney, T. Y., Hoskinson, R. A. J., … & O'Brien, C. P. (2016). Extended-Release Naltrexone to Prevent Opioid Relapse in Criminal Justice Offenders. *The New England journal of medicine*. 374(13), 1232-1242.
81. Ling, W., Amass, L., Shoptaw, S., Annon, J. J., Hillhouse, M., Babcock, D., … & Buprenorphine Study Protocol Group. (2005). A multi-center randomized trial of buprenorphine-naloxone versus clonidine for opioid detoxification: findings from the National Institute on Drug Abuse Clinical Trials Network. *Addiction (Abingdon, England).* 100(8), 1090-1100.
82. Lofwall, M. R., Walsh, S. L., Nunes, E. V., Bailey, G. L., Sigmon, S. C., Kampman, K. M., ... & Oosman, S. (2018). Weekly and monthly subcutaneous buprenorphine depot formulations vs daily sublingual buprenorphine with naloxone for treatment of opioid use disorder: A randomized clinical trial. *JAMA internal medicine*, *178*(6), 764-773.
83. Maarefvand, M., Eghlima, M., Rafiey, H., Rahgozar, M., Tadayyon, N., Deilamizadeh, A., & Ekhtiari, H. (2015). Community-based relapse prevention for opiate dependents: a randomized community controlled trial. *Community mental health journal*. 51(1), 21-29.
84. Marissen, M. A. E., Franken, I. H. A., Blanken, P., van den Brink, W., & Hendriks, V. M. (2007). Cue exposure therapy for the treatment of opiate addiction: results of a randomized controlled clinical trial. *Psychotherapy and Psychosomatics*. 76(2), 97-105.
85. Marissen, M. A. E., Franken, I. H. A., Waters, A. J., Blanken, P., van den Brink, W., & Hendriks, V. M. (2006). Attentional bias predicts heroin relapse following treatment. *Addiction (Abingdon, England).* 101(9), 1306-1312.
86. Marsch, L. A., Bickel, W. K., Badger, G. J., Stothart, M. E., Quesnel, K. J., Stanger, C., & Brooklyn, J. (2005). Comparison of pharmacological treatments for opioid-dependent adolescents: a randomized controlled trial. *Archives of general psychiatry*. 62(10), 1157-1164.
87. Martino, S., Paris, M. Jr, Anez, L., Nich, C., Canning-Ball, M., Hunkele, K., … & Carroll, K. M. (2016). The Effectiveness and Cost of Clinical Supervision for Motivational Interviewing: A Randomized Controlled Trial. *Journal of substance abuse treatment.* 68, 11-23.
88. Masson, C. L., Barnett, P. G., Sees, K. L., Delucchi, K. L., Rosen, A., Wong, W., & Hall, S. M. (2004). Cost and cost-effectiveness of standard methadone maintenance treatment compared to enriched 180-day methadone detoxification. *Addiction (Abingdon, England).* 99(6), 718-726.
89. McCaul, M. E., Stitzer, M. L., Bigelow, G. E., & Liebson, I. A. (1984). Contingency management interventions: effects on treatment outcome during methadone detoxification. *Journal of applied behavior analysis.* 17(1), 35-43.
90. Min, Z., Xu, L., Chen, H., Ding, X., Yi, Z., and Mingyuang, Z. (2011). A pilot assessment of relapse prevention for heroin addicts in a Chinese rehabilitation center. *The American journal of drug and alcohol abuse.* 37(3), 141-147.
91. Morie, K. P., Nich, C., Hunkele, K., Potenza, M. N., & Carroll, K. M. (2015). Alexithymia level and response to computer-based training in cognitive behavioral therapy among cocaine-dependent methadone maintained individuals. *Drug and alcohol dependence*. 152, 157-163.
92. Nunes, E., Bisaga, A., Krupitsky, E., Silverman, B., Akerman, S., & Sullivan, M. (2018, June). Effects of Extended-Release Naltrexone Extend Beyond Exogenous Opioid Blockade: Clinical Trial Observations. In *American Journal on Addictions* (Vol. 27, No. 4, Pp. 322-323). USA: Wiley.
93. Nunes, E. V., Rothenberg, J. L., Sullivan, M. A., Carpenter, K. M., & Kleber, H. D. (2006). Behavioral therapy to augment oral naltrexone for opioid dependence: a ceiling on effectiveness? *The American journal of drug and alcohol abuse*. 32(4), 503-517.
94. Nuttbrock, L. A., Rahav, M., Rivera, J. J., Ng-Mak, D. S., & Link, B. G. (1998). Outcomes of homeless mentally ill chemical abusers in community residences and a therapeutic community. *Psychiatric services (Washington, D.C.).* 49(1), 68-76.
95. Otiashvili, D., Kirtadze, I., O'Grady, K. E., & Jones, H. E. (2012). Drug use and HIV risk outcomes in opioid-injecting men in the Republic of Georgia: behavioral treatment + naltrexone compared to usual care. *Drug and alcohol dependence.* 120(1-3), 14-21.
96. Perneger, T. V., Giner, F., del Rio, M., & Mino, A. (1998). Randomised trial of heroin maintenance programme for addicts who fail in conventional drug treatments. *Bmj*, *317*(7150), 13-18.
97. Petry, N. M., Alessi, S. M., Marx, J., Austin, M., & Tardif, M. (2005). Vouchers versus prizes: contingency management treatment of substance abusers in community settings. *Journal of consulting and clinical psychology.* 73(6), 1005-1014.
98. Petry, N. M., Roll, J. M., Rounsaville, B. J., Ball, S. A., Stitzer, M., Peirce, J. M., … & Carroll, K. M. (2008). Serious adverse events in randomized psychosocial treatment studies: safety or arbitrary edicts? *Journal of consulting and clinical psychology*. 76(6), 1076-1082.
99. Petry, N. M., Weinstock, J., & Alessi, S. M. (2011). A randomized trial of contingency management delivered in the context of group counseling. *Journal of consulting and clinical psychology.* 79(5), 686-696.
100. Piotrowski, N. A., Tusel, D. J., Sees, K. L., Reilly, P. M., Banys, P., Meek, P., & Hall, S. M. (1999). Contingency contracting with monetary reinforcers for abstinence from multiple drugs in a methadone program. *Experimental and Clinical Psychopharmacology*, *7*(4), 399.
101. Potter, J. S., Dreifuss, J. A., Marino, E. N., Provost, S. E., Dodd, D. R., Rice, L. S., … & Weiss, R. D. (2015). The multi-site prescription opioid addiction treatment study: 18-month outcomes. *Journal of substance abuse treatment.* 48(1), 62-69.
102. Preston, K. L., Silverman, K., Umbricht, A., DeJesus, A., Montoya, I. D., & Schuster, C. R. (1999). Improvement in naltrexone treatment compliance with contingency management. *Drug and alcohol dependence.* 54(2), 127-135.
103. Raby, W. N., Carpenter, K. M., Rothenberg, J., Brooks, A. C., Jiang, H., Sullivan, M., … & Nunes, E. V. (2009). Intermittent marijuana use is associated with improved retention in naltrexone treatment for opiate-dependence. *The American journal on addictions.* 18(4), 301-308.
104. Raheb, G., Khaleghi, E., Moghanibashi-Mansourieh, A., Farhoudian, A., & Teymouri, R. (2016). Effectiveness of social work intervention with a systematic approach to improve general health in opioid addicts in addiction treatment centers. *Psychology research and behavior management.* 9, 309-315.
105. Rawson, R. A., Mann, A. J., Tennant, F. S. J., & Clabough, D. (1983). Efficacy of psychotherapeutic counselling during 21-day ambulatory heroin detoxification. *Drug and alcohol dependence.* 12(2), 197-200.
106. Reid, M. S., Jiang, H., Fallon, B., Sonne, S., Rinaldi, P., Turrigiano, E., … & Nunes, E. V. (2011). Smoking cessation treatment among patients in community-based substance abuse rehabilitation programs: exploring predictors of outcome as clues toward treatment improvement. *The American journal of drug and alcohol abuse.* 37(5), 472-478.
107. Robles, E., Stitzer, M. L., Strain, E. C., Bigelow, G. E., & Silverman, K. (2002). Voucher-based reinforcement of opiate abstinence during methadone detoxification. *Drug and alcohol dependence.* 65(2), 179-189.
108. Romijn, C. M., Platt, J. J., & Schippers, G. M. (1990). Family therapy for Dutch drug abusers: replication of an American study. *The International journal of the addictions*. 25(10), 1127-1149.
109. Rosenblum, A., Magura, S., Kayman, D. J., & Fong, C. (2005). Motivationally enhanced group counseling for substance users in a soup kitchen: a randomized clinical trial. *Drug and alcohol dependence*. 80(1), 91-103.
110. Ruger, J. P., Chawarski, M., Mazlan, M., Ng, N., & Schottenfeld, R. (2012). Cost-effectiveness of buprenorphine and naltrexone treatments for heroin dependence in Malaysia. *PloS one*. 7(12), e50673.
111. Saitz, R., Cheng, D. M., Winter, M., Kim, T. W., Meli, S. M., Allensworth-Davies, D., … & Samet, J. H. (2013). Chronic care management for dependence on alcohol and other drugs: the AHEAD randomized trial. *JAMA.* 310(11), 1156-1167.
112. Sannibale, C., Hurkett, P., van den Bossche, E., O'Connor, D., Zador, D., Capus, C., … & McKenzie, M. (2003). Aftercare attendance and post-treatment functioning of severely substance dependent residential treatment clients. *Drug and alcohol review*. 22(2), 181-190.
113. Saunders, E. C., McGovern, M. P., Lambert-Harris, C., Meier, A., McLeman, B., & Xie, H. (2015). The impact of addiction medications on treatment outcomes for persons with co-occurring PTSD and opioid use disorders. *The American journal on addictions.* 24(8), 722-731.
114. Schottenfeld, R. S., Chawarski, M. C., & Mazlan, M. (2008). Maintenance treatment with buprenorphine and naltrexone for heroin dependence in Malaysia: a randomised, double-blind, placebo-controlled trial. *Lancet*. 371(9631), 2192-2200.
115. Schwartz, R. P., Highfield, D. A., Jaffe, J. H., Brady, J. V., Butler, C. B., Rouse, C. O., … & Battjes, R. J. (2006). A randomized controlled trial of interim methadone maintenance. *Archives of general psychiatry.* 63(1), 102-109.
116. Schwartz, R. P., Kelly, S. M., O'Grady, K. E., Gandhi, D., & Jaffe, J. H. (2011). Interim methadone treatment compared to standard methadone treatment: 4-month findings. *Journal of substance abuse treatment.* 41(1), 21-29.
117. Secades-Villa, R., Fernande-Hermida, J. Ramon, & Arnaez-Montaraz, C. (2004). Motivational interviewing and treatment retention among drug user patients: a pilot study. *Substance use & misuse*. 39(9), 1369-1378.
118. Sees, K. L., Delucchi, K. L., Masson, C., Rosen, A., Clark, H. W., Robillard, H., … & Hall, S. M. (2000). Methadone maintenance vs 180-day psychosocially enriched detoxification for treatment of opioid dependence: a randomized controlled trial. *JAMA*. 283(10), 1303-1310.
119. Shava, E., Lipira, L. E., Beauchamp, G. G., Donnell, D. J., Lockman, S., Ruan, Y., & Shao, Y. (2018). Risky Sexual Behavior Among Individuals Receiving Buprenorphine/Naloxone Opiate Dependency Treatment: HIV Prevention Trials Network (HPTN) 058. *Journal of acquired immune deficiency syndromes (1999)*, *78*(3), 300.
120. Shojaei Ghalehney, Z., Ilbeigi, S., Arshadi, H. R., & Afshari, R. (2018). Superiority of Buprenorphine over Suboxone in Preventing Addiction Relapse in Opioid Addicts under Maintenance Therapy: A Double-Blind Clinical Trial. *Asia Pacific Journal of Medical Toxicology*, *7*(1), 1-6.
121. Sigmon, S. C., Ochalek, T. A., Hruska, B., Heil, S. H., Higgins, S., Rose, G., & Moore, B. A. (2017). Interim buprenorphine treatment for reducing illicit opioid use during treatment delays. *Drug and alcohol dependence*. 171, e190-e191.
122. Soares III, W. E., Wilson, D., Gordon, M. S., Lee, J. D., Nunes, E. V., O’Brien, C. P., ... & Friedmann, P. D. (2019). Incidence of future arrests in adults involved in the criminal justice system with opioid use disorder receiving extended release naltrexone compared to treatment as usual. *Drug and alcohol dependence*, *194*, 482-486.
123. Solli, K., Opheim, A., Kunoe, N., & Tanum, L. (2018). The Effectiveness of Injectable Extended Release Naltrexone versus daily Buprenorphine-Naloxone for Opioid Dependence: A Randomized Clinical trial.(P2. 092).
124. Sorensen, J. L., Masson, C. L., Delucchi, K., Sporer, K., Barnett, P. G., Mitsuishi, F., … & Hall, S. M. (2005). Randomized trial of drug abuse treatment-linkage strategies. *Journal of consulting and clinical psychology.* 73(6), 1026-1035.
125. Stitzer, M. L., Iguchi, M. Y., & Felch, L. J. (1992). Contingent take-home incentive: effects on drug use of methadone maintenance patients. *Journal of consulting and clinical psychology*. 60(6), 927-934.
126. Stotts, A. L., Green, C., Masuda, A., Grabowski, J., Wilson, K., Northrup, T. F., … & Schmitz, J. M. (2012). A stage I pilot study of acceptance and commitment therapy for methadone detoxification. *Drug and alcohol dependence*. 125(3), 215-222.
127. Strang, J., Marks, I., Dawe, S., Powell, J., Gossop, M., Richards, D., & Gray, J. (1997). Type of hospital setting and treatment outcome with heroin addicts. Results from a randomised trial. *The British journal of psychiatry : the journal of mental science.* 171, 335-339.
128. Strathdee, S. A., Ricketts, E. P., Huettner, S., Cornelius, L., Bishai, D., Havens, J. R., … & Latkin, C. A. (2006). Facilitating entry into drug treatment among injection drug users referred from a needle exchange program: Results from a community-based behavioral intervention trial. *Drug and alcohol dependence*. 83(3), 225-232.
129. Streck, J. M., Ochalek, T. A., Badger, G. J., & Sigmon, S. C. (2018). Interim buprenorphine treatment during delays to comprehensive treatment: Changes in psychiatric symptoms. *Experimental and clinical psychopharmacology*, *26*(4), 403.
130. Streck, J. M., Ochalek, T. A., Hruska, B., Pusey, J. D., & Sigmon, S. C. (2017). Improvement in psychiatric symptoms during interim buprenorphine treatment. *Drug and alcohol dependence*. 171, e198.
131. Sullivan, M, A., Bisaga, A., Glass, A., Mishlen, K., Pavlicova, M., Carpenter, K. M., … & Nunes, E. V. (2015). Opioid use and dropout in patients receiving oral naltrexone with or without single administration of injection naltrexone. *Drug and alcohol dependence*. 147, 122-129.
132. Sullivan, M. A., Bisaga, A., Pavlicova, M., Carpenter, K. M., Choi, C. J., Mishlen, K., ... & Nunes, E. V. (2019). A randomized trial comparing extended-release injectable suspension and oral naltrexone, both combined with behavioral therapy, for the treatment of opioid use disorder. *American Journal of Psychiatry.* 176(2), 129-137.
133. Sullivan, M., Mannelli, P., Yu, M., Nangia, N., Graham, C., Webster, I., ... & Silverman, B. (2018, June). Outpatient Transition to Extended-release Naltrexone in Patients With Opioid-use Disorder. In *American Journal on Addictions* (Vol. 27, No. 4, pp. 323-323). USA: WILEY.
134. Tanum, L., Solli, K., Latif, Z.-E.-H., Benth, J. Š., Opheim, A., Krajci, P., & Kunoe, N. (2018). S273. The Effectiveness of Injectable Extended Release Naltrexone Versus Daily Buprenorphine-Naloxone for Opioid Dependence in Short and Long Term Treatment. *Biological Psychiatry*, *83*(9). doi: 10.1016/j.biopsych.2018.02.1165
135. Tavakolian, E., & Abolghasemi, A. (2016). Effects of cognitive restructuring training on neurocognitive functions in opioid addicts. *Archives of Psychiatry and Psychotherapy*. 18(1), 14-21.
136. Tucker, T., Ritter, A., Maher, C., & Jackson, H. (2004). A randomized control trial of group counseling in a naltrexone treatment program. *Journal of substance abuse treatment.* 27(4), 277-288.
137. Tuten, M., DeFulio, A., Jones, H. E., & Stitzer, M. (2012). Abstinence-contingent recovery housing and reinforcement-based treatment following opioid detoxification. *Addiction (Abingdon, England).* 107(5), 973-982.
138. Wang, X., Jiang, H., Zhao, M., Li, J., Gray, F., Sheng, L., ... & Hao, W. (2019). Treatment of opioid dependence with buprenorphine/naloxone sublingual tablets: A phase 3 randomized, double‐blind, placebo‐controlled trial. *Asia‐Pacific Psychiatry*, *11*(1), e12344.
139. Wechsberg, W. M., Krupitsky, E., Romanova, T., Zvartau, E., Kline, T. L., Browne, F. A., … & Jones, H. E. (2012). Double jeopardy--drug and sex risks among Russian women who inject drugs: initial feasibility and efficacy results of a small randomized controlled trial. S*ubstance abuse treatment, prevention, and policy.* 7, 1.
140. Weiss, R. D., Potter, J. S., Fiellin, D. A., Byrne, M., Connery, H. S., Dickinson, W., ... & Hasson, A. L. (2011). Adjunctive counseling during brief and extended buprenorphine-naloxone treatment for prescription opioid dependence: a 2-phase randomized controlled trial. *Archives of general psychiatry*, *68*(12), 1238-1246.
141. Yandoli, D., Eisler, I., Robbins, C., Mulleady, G., & Dare, C. (2002). A comparative study of family therapy in the treatment of opiate users in a London drug clinic. *Journal of Family Therapy*, *24*(4), 402-422.
142. Zanis, D. A., McLellan, A. T., Alterman, A. I., & Cnaan, R. A. (1996). Efficacy of enhanced outreach counseling to reenroll high-risk drug users 1 year after discharge from treatment. The *American journal of psychiatry*. 153(8), 1095-1096.
143. Zhao, M., Li, X., Wang, Z.-C., Xu, D., Zhang, Y., & Zhang, M.-Y. (2004). Effect of relapse prevention program on the anxiety and self-efficacy in heroin addict. *Chinese Journal of Clinical Rehabilitation.* 8(27), 5785-5787.
144. Zhong, N., Yuan, Y., Chen, H., Jiang, H., Du, J., Sun, H., … & Zhao, M. (2015). Effects of a Randomized Comprehensive Psychosocial Intervention Based on Cognitive Behavioral Therapy Theory and Motivational Interviewing Techniques for Community Rehabilitation of Patients With Opioid Use Disorders in Shanghai, China. *Journal of addiction medicine.* 9(4), 322-330.
145. Zhuang, S. M., An, S. H., & Zhao, Y. (2014). Effect of cognitive behavioural interventions on the quality of life in Chinese heroin-dependent individuals in detoxification: a randomised controlled trial. *Journal of clinical nursing*. 23(9-10), 1239-1248.
146. Ziedonis, D. M., Amass, L., Steinberg, M., Woody, G., Krejci, J., Annon, J. J., … & Ling, W. (2009). Predictors of outcome for short-term medically supervised opioid withdrawal during a randomized, multicenter trial of buprenorphine-naloxone and clonidine in the NIDA clinical trials network drug and alcohol dependence. *Drug and alcohol dependence*. 99(1-3), 28-36.

**Ineligible or Lack of Psychosocial Intervention Being Tested (n=120)**

1. Abbott, P. J., Moore, B., Delaney, H., & Weller, S. (1999). Retrospective analyses of additional services for methadone maintenance patients. *Journal of substance abuse treatment*. 17(1-2), 129-137.
2. Aklin, W. M., Wong, C. J., Hampton, J., Svikis, D. S., Stitzer, M. L., Bigelow, G. E., & Silverman, K. (2014). A therapeutic workplace for the long-term treatment of drug addiction and unemployment: eight-year outcomes of a social business intervention. *Journal of substance abuse treatment*. 47(5), 329-338.
3. Avants, S. K., Margolin, A., Usubiaga, M. H., & Doebrick, C. (2004). Targeting HIV-related outcomes with intravenous drug users maintained on methadone: a randomized clinical trial of a harm reduction group therapy. *Journal of substance abuse treatment*, *26*(2), 67-78.
4. Baker, A., Heather, N., Wodak, A., Dixon, J., & Holt, P. (1993). Evaluation of a cognitive-behavioural intervention for HIV prevention among injecting drug users. *AIDS (London, England).* 7(2), 247-256.
5. Baker, J. G., Rounds, J. B., & Carson, C. A. (1995). Monitoring in methadone maintenance treatment. *The International journal of the addictions*. 30(9), 1177-1185.
6. Barnett, P. G., Sorensen, J. L., Wong, W., Haug, N. A., & Hall, S. M. (2009). Effect of incentives for medication adherence on health care use and costs in methadone patients with HIV. *Drug and alcohol dependence.* 100(1-2), 115-121.
7. Blanken, P., Hendriks, V. M., Huijsman, I. A., van Ree, J. M., & van den Brink, W. (2016). Efficacy of cocaine contingency management in heroin-assisted treatment: Results of a randomized controlled trial. *Drug and alcohol dependence*. 164, 55-63.
8. Brooner, R. K., Kidorf, M., King, V. L., & Stoller, K. (1998). Preliminary evidence of good treatment response in antisocial drug abusers1. *Drug and Alcohol Dependence*, *49*(3), 249-260.
9. Brooner, R. K., Kidorf, M. S., King, V. L., Peirce, J., Neufeld, K., Stoller, K., & Kolodner, K. (2013). Managing psychiatric comorbidity within versus outside of methadone treatment settings: a randomized and controlled evaluation. *Addiction (Abingdon, England).* 108(11), 1942-1951.
10. Brown, R., Gassman, M., Hetzel, S., & Berger, L. (2013). Community-based treatment for opioid dependent offenders: a pilot study. *The American journal on addictions*. 22(5), 500-502.
11. Carpenter, K. M., Smith, J. L., Aharonovich, E., & Nunes, E. V. (2008). Developing therapies for depression in drug dependence: results of a stage 1 therapy study. *The American journal of drug and alcohol abuse.* 34(5), 642-652.
12. Chutuape, M. A., Silverman, K., & Stitzer, M. L. (2001). Effects of urine testing frequency on outcome in a methadone take-home contingency program. *Drug and alcohol dependence*, *62*(1), 69-76.
13. Copenhaver, M. M., Lee, I. C., & Baldwin, P. (2013). A randomized controlled trial of the community-friendly health recovery program (CHRP) among high-risk drug users in treatment. *AIDS and behavior*. 17(9), 2902-2913.
14. Coviello, D. M., Zanis, D. A., Wesnoski, S. A., & Domis, S. W. (2009). An integrated drug counseling and employment intervention for methadone clients. *Journal of psychoactive drugs*, *41*(2), 189-197.
15. Coviello, D. M., Zanis, D. A., & Lynch, K. (2004). Effectiveness of vocational problem-solving skills on motivation and job-seeking action steps. *Substance use & misuse*. 39(13-14), 2309-2324.
16. Dale, S. K., Traeger, L., O'Cleirigh, C., Bedoya, C. A., Pinkston, M., Wilner, J. G., … & Safren, S. A. (2016). Baseline Substance Use Interferes with Maintenance of HIV Medication Adherence Skills. *AIDS Patient Care and STDs*. 30(5), 215-220.
17. Dawe, S., & Harnett, P. (2007). Reducing potential for child abuse among methadone-maintained parents: results from a randomized controlled trial. *Journal of substance abuse treatment.* 32(4), 381-390.
18. DeFulio, A. & Silverman, K. (2011). Employment-based abstinence reinforcement as a maintenance intervention for the treatment of cocaine dependence: post-intervention outcomes. *Addiction (Abingdon, England).* 106(5), 960-967.
19. Dehghani-Arani, F., Rostami, R., & Nadali, H. (2013). Neurofeedback training for opiate addiction: improvement of mental health and craving. Ap*plied psychophysiology and biofeedback*. 38(2), 133-141.
20. Demaret, I., Quertemont, E., Litran, G., Magoga, C., Deblire, C., Dubois, N., … & Ansseau, M. (2015). Efficacy of Heroin-assisted Treatment In Belgium: A Randomised Controlled Trial. *European addiction research.* 21(4), 179-187.
21. Dhawan, A., Chopra, A., Jain, R., Yadav, D., and Vedamurthachar. (2015). Effectiveness of yogic breathing intervention on quality of life of opioid dependent users. *International journal of yoga.* 8(2), 144-147.
22. Dunn, K. E., Saulsgiver, K. A., & Sigmon, S. C. (2011). Contingency management for behavior change: applications to promote brief smoking cessation among opioid-maintained patients. *Experimental and clinical psychopharmacology*. 19(1), 20-30.
23. Dunn, K. E., Sigmon, S. C., Thomas, C. S., Heil, S. H., & Higgins, S. T. (2008). Voucher-based contingent reinforcement of smoking abstinence among methadone-maintained patients: a pilot study. *Journal of applied behavior analysis*. 41(4), 527-538.
24. Edelman, E J., Moore, B. A., Caffrey, S., Sikkema, K. J., Jones, E. S., Schottenfeld, R. S., … & Fiellin, L. E. (2013). HIV testing and sexual risk reduction counseling in office-based buprenorphine/naloxone treatment. *Journal of addiction medicine.* 7(6), 410-416.
25. El-Bassel, N., & Schilling, R. F. (1992). 15-month followup of women methadone patients taught skills to reduce heterosexual HIV transmission. *Public health reports (Washington, D.C.: 1974*). 107(5), 500-504.
26. Esmaeili, A., Khodadadi, M., Norozi, E., & Miri, M. R. (2018). Effectiveness of mindfulness-based cognitive group therapy on cognitive emotion regulation of patients under treatment with methadone. *Journal of Substance Use*. 23(1), 58-62.
27. Festinger, D. S., Dugosh, K. L., Kirby, K. C., & Seymour, B. L. (2014). Contingency management for cocaine treatment: Cash vs. vouchers. *Journal of substance abuse treatment.* 47(2), 168-174.
28. Fiellin, D. A., O'connor, P. G., Chawarski, M., Pakes, J. P., Pantalon, M. V., & Schottenfeld, R. S. (2001). Methadone maintenance in primary care: a randomized controlled trial. *Jama*, *286*(14), 1724-1731.
29. Gibson, A. E., Doran, C. M., Bell, J. R., Ryan, A., & Lintzeris, N. (2003). A comparison of buprenorphine treatment in clinic and primary care settings: a randomised trial. *The Medical journal of Australia*. 179(1), 38-42.
30. Gilbert, L., El-Bassel, N., Manuel, J., Wu, E., Go, H., Golder, S., ... & Sanders, G. (2006). An integrated relapse prevention and relationship safety intervention for women on methadone: testing short-term effects on intimate partner violence and substance use. *Violence and Victims*, *21*(5), 657.
31. Gordon, M. S., Kinlock, T. W., Couvillion, K. A., Schwartz, R. P., & O'Grady, K. (2012). A Randomized Clinical Trial of Methadone Maintenance for Prisoners: Prediction of Treatment Entry and Completion in Prison. *Journal of Offender Rehabilitation*. 51(4), 222-238.
32. Gordon, M. S., Kinlock, T. W., Schwartz, R. P., & O'Grady, K. E. (2008). A randomized clinical trial of methadone maintenance for prisoners: findings at 6 months post-release. *Addiction (Abingdon, England).* 103(8), 1333-1342.
33. Gordon, M. S., Kinlock, T. W., Schwartz, R. P., Fitzgerald, T. T., O'Grady, K. E., & Vocci, F. J. (2014). A randomized controlled trial of prison-initiated buprenorphine: prison outcomes and community treatment entry. *Drug and alcohol dependence*. 142, 33-40.
34. Greenwald, M. K. (2005). Opioid craving and seeking behavior in physically dependent volunteers: effects of acute withdrawal and drug reinforcement opportunity. *Experimental and clinical psychopharmacology.* 13(1), 3-14.
35. Greenwald, M. K., Schuh, K. J., Hopper, J. A., Schuster, C. R., & Johanson, C. E. (2002). Effects of buprenorphine sublingual tablet maintenance on opioid drug-seeking behavior by humans. *Psychopharmacology*. 160(4), 344-352.
36. Gruber, V. A., Delucchi, K. L., Kielstein, A., & Batki, S. L. (2008). A randomized trial of 6-month methadone maintenance with standard or minimal counseling versus 21-day methadone detoxification. *Drug and alcohol dependence*. 94(1-3), 199-206.
37. Haasen, C., Verthein, U., Eiroa-Orosa, F. J., Schafer, I., & Reimer, J. (2010). Is heroin-assisted treatment effective for patients with no previous maintenance treatment? Results from a German randomised controlled trial. *European addiction research*. 16(3), 124-130.
38. Haight, B., Andorn, A., Laffont, C., Young, M., Jones, A., Learned, S., & Heidbreder, C. (2017). RBP-6000 buprenorphine monthly depot demonstrates sustained clinical efficacy and safety in phase III opioid use disorder trials. *Neuropsychopharmacology : official publication of the American College of Neuropsychopharmacology*. 43(Supplement 1), S463-S464.
39. Harris, R. M., Bausell, R. B., Scott, D. E., Hetherington, S. E., & Kavanagh, K. H. (1998). An intervention for changing high-risk HIV behaviors of African American drug-dependent women. *Research in nursing & health*. 21(3), 239-250.
40. Haug, N. A., Svikis, D. S., & Diclemente, C. (2004). Motivational enhancement therapy for nicotine dependence in methadone-maintained pregnant women. *Psychology of addictive behaviors: journal of the Society of Psychologists in Addictive Behaviors*. 18(3), 289-292.
41. Havassy, B., Hargreaves, W. A., & De Barros, L. (1979). Self-regulation of dose in methadone maintenance with contingent privileges. *Addictive Behaviors*, *4*(1), 31-38.
42. Havens, J. R., Latkin, C. A., Pu, M., Cornelius, L. J., Bishai, D., Huettner, S., … & Strathdee, S. A. (2009). Predictors of opiate agonist treatment retention among injection drug users referred from a needle exchange program. *Journal of substance abuse treatment.* 36(3), 306-312.
43. Hojjat, S. K., Raufpoor, R., Khalili, M. N., Hamidi, M., Danesh, M., & Ziarat, H. M. (2016). Effects of Vocational Consultation on Relapse Rate and Hope among Drug Dependents in Bojnurd, Iran. *Electronic physician*. 8(1), 1711-1717.
44. Houston, C. C., & Miiby, J. B. (1983). Drug-seeking behavior and its mediation: effects of aversion therapy with narcotic addicts on methadone. *International journal of the addictions*, 18(8), 1171-1177.
45. Iguchi, M. Y., Lamb, R. J., Belding, M. A., Platt, J. J., Husband, S. D., & Morral, A. R. (1996). Contingent reinforcement of group participation versus abstinence in a methadone maintenance program. *Experimental and Clinical Psychopharmacology*, *4*(3), 315.
46. Jones, J. D., Bisaga, A., Metz, V. E., Manubay, J. M., Mogali, S., Ciccocioppo, R., ... & Comer, S. D. (2018). The PPARγ Agonist Pioglitazone Fails to Alter the Abuse Potential of Heroin, But Does Reduce Heroin Craving and Anxiety. *Journal of psychoactive drugs*, *50*(5), 390-401.
47. Jones, H. E., Haug, N., Silverman, K., Stitzer, M., & Svikis, D. (2001). The effectiveness of incentives in enhancing treatment attendance and drug abstinence in methadone-maintained pregnant women. *Drug and alcohol dependence*, *61*(3), 297-306.
48. Karuntzos, G. T., Caddell, J. M., & Dennis, M. L. (1994). Gender differences in vocational needs and outcomes for methadone treatment clients. *Journal of psychoactive drugs*. 26(2), 173-180.
49. Kennedy, A. P., Phillips, K. A., Epstein, D. H., Reamer, D. A., Schmittner, J., & Preston, K. L. (2013). A randomized investigation of methadone doses at or over 100 mg/day, combined with contingency management. *Drug and alcohol dependence*, *130*(1-3), 77-84.
50. Kidorf, M., Brooner, R. K., Gandotra, N., Antoine, D., King, V. L., Peirce, J., & Ghazarian, S. (2013). Reinforcing integrated psychiatric service attendance in an opioid-agonist program: a randomized and controlled trial. *Drug and alcohol dependence*, *133*(1), 30-36.
51. King, V. L., Brooner, R. K., Peirce, J. M., Kolodner, K., & Kidorf, M. S. (2014). A randomized trial of Web-based videoconferencing for substance abuse counseling. *Journal of substance abuse treatment*, *46*(1), 36-42.
52. King, V. L., Stoller, K. B., Kidorf, M., Kindbom, K., Hursh, S., Brady, T., & Brooner, R. K. (2009). Assessing the effectiveness of an Internet-based videoconferencing platform for delivering intensified substance abuse counseling. *Journal of substance abuse treatment*, *36*(3), 331-338.
53. Kinlock, T. W., Gordon, M. S., Schwartz, R. P., & O'Grady, K. E. (2008). A Study of Methadone Maintenance For Male Prisoners: 3-Month Postrelease Outcomes. *Criminal justice and behavior.* 35(1), 34-47.
54. Kinlock, T. W., Gordon, M. S., Schwartz, R. P., Fitzgerald, T. T., & O'Grady, K. E. (2009). A randomized clinical trial of methadone maintenance for prisoners: results at 12 months postrelease. *Journal of substance abuse treatment*. 37(3), 277-285.
55. Knealing, T. W., Wong, C. J., Diemer, K. N., Hampton, J., & Silverman, K. (2006). A randomized controlled trial of the therapeutic workplace for community methadone patients: A partial failure to engage. *Experimental and Clinical Psychopharmacology*, *14*(3), 350.
56. Larios, S. E., Masson, C. L., Shopshire, M. S., Hettema, J., Jordan, A. E., McKnight, C., … & Perlman, D. C. (2014). Education and counseling in the methadone treatment setting improves knowledge of viral hepatitis. *Journal of substance abuse treatment.* 46(4), 528-531.
57. Li, L., Wu, Z., Liang, L. J., Lin, C., Luo, S., Cao, X., ... & Rou, K. (2019). An intervention trial targeting methadone maintenance treatment providers to improve clients’ treatment retention in China. *Drug and alcohol dependence*, *194*, 143-150.
58. Liang, D., Han, H., Du, J., Zhao, M., & Hser, Y. I. (2018). A pilot study of a smartphone application supporting recovery from drug addiction. *Journal of substance abuse treatment*, *88*, 51-58.
59. Lucas, G. M., Beauchamp, G., Aramrattana, A., Shao, Y., Liu, W., Fu, L., … & Study Group. (2012). Short-term safety of buprenorphine/naloxone in HIV-seronegative opioid-dependent Chinese and Thai drug injectors enrolled in HIV Prevention Trials Network 058. *The International journal on drug policy.* 23(2), 162-165.
60. Magura, S., Blankertz, L., Madison, E. M., Friedman, E., & Gomez, A. (2007). An innovative job placement model for unemployed methadone patients: a randomized clinical trial. *Substance use & misuse*. 42(5), 811-828.
61. Margolin, A., Avants, S. K., & Arnold, R. (2005). Acupuncture and spirituality-focused group therapy for the treatment of HIV-positive drug users: a preliminary study. Jo*urnal of psychoactive drugs.* 37(4), 385-390.
62. Margolin, A., Beitel, M., Schuman-Olivier, Z., & Avants, S. K. (2006). A controlled study of a spirituality-focused intervention for increasing motivation for HIV prevention among drug users. AIDS education and prevention : official publication of the International Society for *AIDS Education*. 18(4), 311-322.
63. Masson, C. L., Delucchi, K. L., McKnight, C., Hettema, J., Khalili, M., Min, A., … & Perlman, D. C. (2013). A randomized trial of a hepatitis care coordination model in methadone maintenance treatment. *American journal of public health.* 103(10), e81-e88.
64. McHugh, R. K., Murray, H. W., Hearon, B. A., Pratt, E. M., Pollack, M. H., Safren, S. A., & Otto, M. W. (2013). Predictors of dropout from psychosocial treatment in opioid-dependent outpatients. *The American journal on addictions*. 22(1), 18-22.
65. Meade, C. S., Weiss, R. D., Fitzmaurice, G. M., Poole, S. A., Subramaniam, G. A., Patkar, A. A., … & Woody, G. E. (2010). HIV risk behavior in treatment-seeking opioid-dependent youth: results from a NIDA clinical trials network multisite study. *Journal of acquired immune deficiency syndromes* (1999). 55(1), 65-72.
66. Metzger, D. S., Donnell, D., Celentano, D. D., Jackson, J. B., Shao, Y., Aramrattana, A., … & Protocol Team. (2015). Expanding substance use treatment options for HIV prevention with buprenorphine-naloxone: HIV Prevention Trials Network 058. *Journal of acquired immune deficiency syndromes (1999).* 68(5), 554-561.
67. Mitchell, S. G., Gryczynski, J., Schwartz, R. P., O’Grady, K. E., Olsen, Y. K., & Jaffe, J. H. (2013). A randomized trial of intensive outpatient (IOP) vs. standard outpatient (OP) buprenorphine treatment for African Americans. *Drug and Alcohol Dependence*, *128*(3), 222-229.
68. Mitcheson, L., McCambridge, J., & Byrne, S. (2007). Pilot cluster-randomised trial of adjunctive motivational interviewing to reduce crack cocaine use in clients on methadone maintenance. *European addiction research*. 13(1), 6-10.
69. Montoya, I. D., Gorelick, D. A., Preston, K. L., Schroeder, J R., Umbricht, A., Cheskin, L. J., … & Fudala, P. J. (2004). Randomized trial of buprenorphine for treatment of concurrent opiate and cocaine dependence. *Clinical Pharmacology and Therapeutics*. 75(1), 34-48.
70. Mooney, M. E., Poling, J., Gonzalez, G., Gonsai, K., Kosten, T., & Sofuoglu, M. (2008). Preliminary study of buprenorphine and bupropion for opioid-dependent smokers. *The American journal on addictions*. 17(4), 287-292.
71. Moore, B. A., Buono, F. D., Printz, D., Lloyd, D. P., Fiellin, D. A., Cutter, C. J., ... & Barry, D. T. (2017). Customized recommendations and reminder text messages for automated, computer-based treatment during methadone. *Experimental and clinical psychopharmacology*, *25*(6), 485.
72. Neufeld, K. J., Kidorf, M. S., Kolodner, K., King, V. L., Clark, M., & Brooner, R. K. (2008). A behavioral treatment for opioid-dependent patients with antisocial personality. *Journal of Substance Abuse Treatment*, *34*(1), 101-111.
73. Nyamathi, A., Sinha, K., Greengold, B., Cohen, A., & Marfisee, M. (2010). Predictors of HAV/HBV vaccination completion among methadone maintenance clients. *Research in nursing & health.* 33(2), 120-132.
74. Oliveto, A., Poling, J., Mancino, M. J., Feldman, Z., Cubells, J. F., Pruzinsky, R., … & Kosten, T. R. (2011). Randomized, double blind, placebo-controlled trial of disulfiram for the treatment of cocaine dependence in methadone-stabilized patients. *Drug and alcohol dependence*. 113(2-3), 184-191.
75. Oviedo-Joekes, E., Guh, D., Brissette, S., Marchand, K., Marsh, D., Chettiar, J., … & Schechter, M. T. (2010). Effectiveness of diacetylmorphine versus methadone for the treatment of opioid dependence in women. *Drug and alcohol dependence*. 111(1-2), 50-57.
76. Peirce, J. M., Petry, N. M., Stitzer, M. L., Blaine, J., Kellogg, S., Satterfield, F., ... & Kirby, K. C. (2006). Effects of lower-cost incentives on stimulant abstinence in methadone maintenance treatment: A National Drug Abuse Treatment Clinical Trials Network study. *Archives of general psychiatry*, *63*(2), 201-208.
77. Petry, N. M., Alessi, S. M., & Ledgerwood, D. M. (2012). A randomized trial of contingency management delivered by community therapists. *Journal of consulting and clinical psychology*. 80(2), 286-298.
78. Piralishvili, G., Otiashvili, D., Sikharulidze, Z., Kamkamidze, G., Poole, S., & Woody, G. E. (2015). Opioid addicted buprenorphine injectors: drug use during and after 12-weeks of buprenorphine-naloxone or methadone in the Republic of Georgia. *Journal of substance abuse treatment*. 50, 32-37.
79. Pirnia, B., Soleimani, A. A., Malekanmehr, P., Pirnia, K., & Zahiroddin, A. (2018). Topiramate for the Treatment of Dually Dependent on Opiates and Cocaine: A Single-center Placebo-controlled Trial. *Iranian journal of public health*, *47*(9), 1345.
80. Preston, K. L., Ghitza, U. E., Schmittner, J. P., Schroeder, J. R., & Epstein, D. H. (2008). Randomized trial comparing two treatment strategies using prize‐based reinforcement of abstinence in cocaine and opiate users. *Journal of applied behavior analysis*. 41(4), 551-563.
81. Price, C. J., Merrill, J. O., McCarty, R. L., Pike, K. C., & Tsui, J. I. (2020). A pilot study of mindful body awareness training as an adjunct to office-based medication treatment of opioid use disorder. *Journal of Substance Abuse Treatment.* 108, 123-128.
82. Printz, D. M. B., Buono, F. D., Lloyd, D. P., Reichin, S., & Moore, B. A. (2017). Gender differences in timing of reminder messages for automated, CBT-based treatment for methadone. *Drug and alcohol dependence.* 171, e169.
83. Raisch, D. W., Campbell, H. M., Garnand, D. A., Jones, M. A., Sather, M. R., Naik, R., & Ling, W. (2012). Health-related quality of life changes associated with buprenorphine treatment for opioid dependence. *Quality of life research: an international journal of quality of life aspects of treatment, care and rehabilitation*. 21(7), 1177-1183.
84. Rass, O., Schacht, R. L., Buckheit, K., Johnson, M. W., Strain, E. C., & Mintzer, M. Z. (2015). A randomized controlled trial of the effects of working memory training in methadone maintenance patients. *Drug and alcohol dependence*. 156, 38-46.
85. Rawson, R. A., Huber, A., McCann, M., Shoptaw, S., Farabee, D., Reiber, C., & Ling, W. (2002). A comparison of contingency management and cognitive-behavioral approaches during methadone maintenance treatment for cocaine dependence. *Archives of general psychiatry.* 59(9), 817-824.
86. Rezapour, T., Hatami, J., Farhoudian, A., Sofuoglu, M., Noroozi, A., Daneshmand, R., … & Ekhtiari, H. (2017). Cognitive rehabilitation for individuals with opioid use disorder: A randomized controlled trial. *Neuropsychological rehabilitation.* 1-17.
87. Rhodes, G. L., Saules, K. K., Helmus, T. C., Roll, J., Beshears, R. S., Ledgerwood, D. M., & Schuster, C. R. (2003). Improving on-time counseling attendance in a methadone treatment program: a contingency management approach. *The American journal of drug and alcohol abuse.* 29(4), 759-773.
88. Ritter, A. J., Lintzeris, N., Clark, N., Kutin, J. J., Bammer, G., & Panjari, M. (2003). A randomized trial comparing levo-alpha acetylmethadol with methadone maintenance for patients in primary care settings in Australia. *Addiction (Abingdon, England).* 98(11), 1605-1613.
89. Roux, P., Michel, L., Cohen, J., Mora, M., Morel, A., Aubertin, J. F., … & ANRS Methaville study group. (2012). Methadone induction in primary care (ANRS-Methaville): a phase III randomized intervention trial. *BMC public health*. 12, 488.
90. Rowan-Szal, G., Joe, G. W., Chatham, L. R., & Simpson, D. D. (1994). A simple reinforcement system for methadone clients in a community-based treatment program. *Journal of substance abuse treatment*, *11*(3), 217-223.
91. Ruetsch, C., Tkacz, J., McPherson, T. L., & Cacciola, J. (2012). The effect of telephonic patient support on treatment for opioid dependence: outcomes at one year follow-up. *Addictive Behaviors*, *37*(5), 686-689.
92. Schacht, R. L., Brooner, R. K., King, V. L., Kidorf, M. S., & Peirce, J. M. (2017). Incentivizing attendance to prolonged exposure for PTSD with opioid use disorder patients: A randomized controlled trial. *Journal of consulting and clinical psychology*. 85(7), 689-701.
93. Schilling, R. F., El-Bassel, N., Schinke, S. P., Gordon, K., & Nichols, S. (1991). Building skills of recovering women drug users to reduce heterosexual AIDS transmission. Pu*blic health reports (Washington, D.C.: 1974).* 106(3), 297-304.
94. Schmitz, J. M., Rhoades, H. M., Elk, R., Creson, D., Hussein, I., & Grabowski, J. (1998). Medication take-home doses and contingency management. *Experimental and Clinical Psychopharmacology*, *6*(2), 162.
95. Schwartz, R. P., Kelly, S. M., Mitchell, S. G., Gryczynski, J., O'Grady, K. E., Gandhi, D., ... & Jaffe, J. H. (2017). Patient‐centered methadone treatment: a randomized clinical trial. *Addiction*, *112*(3), 454-464.
96. Schottenfeld, R. S., Chawarski, M. C., Sofuoglu, M., Chooi, W. T., Zaharim, N. M., Yasin, M. A. M., ... & Vicknasingam, B. K. (2018). Atomoxetine for amphetamine-type stimulant dependence during buprenorphine treatment: A randomized controlled trial. *Drug and alcohol dependence*, *186*, 130-137.
97. Schottenfeld, R. S., Pakes, J., O'Connor, P., Chawarski, M., Oliveto, A., & Kosten, T. R. (2000). Thrice-weekly versus daily buprenorphine maintenance. *Biological Psychiatry*. 47(12), 1072-1079.
98. Senay, E. C., Barthwell, A. G., Marks, R., Bokos, P., Gillman, D., White, R., & Pristach, C. A. (1993). Medical maintenance: a pilot study. *Journal of addictive diseases*, *12*(4), 59-76.
99. Shaffer, H. J., LaSalvia, T. A., & Stein, J. (1997). Comparing Hatha yoga with dynamic group psychotherapy for enhancing methadone maintenance treatment: a randomized clinical trial. *Alternative Therapies in Health and Medicine*, *3*, 57-67.
100. Shoptaw, S., Rotheram-Fuller, E., Yang, X., Frosch, D., Nahom, D., Jarvik, M. E., … & Ling, W. (2002). Smoking cessation in methadone maintenance. *Addiction (Abingdon, England).* 97(10), 1317-1325.
101. Sigmon, S. C., Dunn, K. E., Saulsgiver, K., Patrick, M. E., Badger, G. J., Heil, S. H., … & Higgins, S. T. (2013). A randomized, double-blind evaluation of buprenorphine taper duration in primary prescription opioid abusers*. JAMA Psychiatry.* 70(12), 1347-1354.
102. Sigmon, S. C., Miller, M. E., Meyer, A. C., Saulsgiver, K., Badger, G. J., Heil, S. H., & Higgins, S. T. (2016). Financial incentives to promote extended smoking abstinence in opioid-maintained patients: a randomized trial. *Addiction (Abingdon, England).* 111(5), 903-912.
103. Silverman, K., Wong, C. J., Umbricht-Schneiter, A., Montoya, I. D., Schuster, C. R., & Preston, K. L. (1998). Broad beneficial effects of cocaine abstinence reinforcement among methadone patients. *Journal of consulting and clinical psychology*. 66(5), 811-824.
104. Silverman, K., Svikis, D., Wong, C. J., Hampton, J., Stitzer, M. L., & Bigelow, G. E. (2002). A reinforcement-based therapeutic workplace for the treatment of drug abuse: three-year abstinence outcomes. *Experimental and Clinical Psychopharmacology*, *10*(3), 228.
105. Silverman, K., Wong, C. J., Needham, M., Diemer, K. N., Knealing, T., Crone‐Todd, D., ... & Kolodner, K. (2007). A randomized trial of employment‐based reinforcement of cocaine abstinence in injection drug users. *Journal of Applied Behavior Analysis*, *40*(3), 387-410.
106. Sofuoglu, M., Gonzalez, G., Poling, J., & Kosten, T. R. (2003). Prediction of treatment outcome by baseline urine cocaine results and self-reported cocaine use for cocaine and opioid dependence. *The American journal of drug and alcohol abuse*. 29(4), 713-727.
107. Sorensen, J. L., Haug, N. A., Delucchi, K. L., Gruber, V., Kletter, E., Batki, S. L., Tulsky, J. P., … & Hall, S. (2007). Voucher reinforcement improves medication adherence in HIV-positive methadone patients: a randomized trial. *Drug and alcohol dependence.* 88(1), 54-63.
108. Specka, M., Böning, A., Kluwig, J., Schifano, F., Banger, M., Lange, W., ... & Scherbaum, N. (2013). Can reinforcement-based interventions to reduce drug use successfully be adapted to routine opioid maintenance treatment?. *Annali dell'Istituto superiore di sanita*, *49*, 358-364.
109. Staines, G. L., Blankertz, L., Magura, S., Bali, P., Madison, E. M., Spinelli, M., … & Friedman, E.. (2004). Efficacy of the customized employment supports (CES) model of vocational rehabilitation for unemployed methadone patients: preliminary results. *Substance use & misuse*. 39(13-14), 2261-2285.
110. Stein, M. D., Weinstock, M. C., Herman, D. S., Anderson, B. J., Anthony, J. L., & Niaura, R. (2006). A smoking cessation intervention for the methadone-maintained. *Addiction (Abingdon, England).* 101(4), 599-607.
111. Strain, E. C., Stitzer, M. L., Liebson, I. A., & Bigelow, G. E. (1996). Buprenorphine versus methadone in the treatment of opioid dependence: self-reports, urinalysis, and addiction severity index. *Journal of clinical psychopharmacology*. 16(1), 58-67.
112. Subramaniam, G. A., Warden, D., Minhajuddin, A., Fishman, M. J., Stitzer, M. L., Adinoff, B., … & Woody, G. E. (2011). Predictors of abstinence: National Institute of Drug Abuse multisite buprenorphine/naloxone treatment trial in opioid-dependent youth. *Journal of the American Academy of Child and Adolescent Psychiatry.* 50(11), 1120-1128.
113. Thornton, P. I., Igleheart, H. C., & Silverman, L. H. (1987). Subliminal stimulation of symbiotic fantasies as an aid in the treatment of drug abusers. *The International journal of the addictions.* 22(8), 751-765.
114. Unger, A., Jagsch, R., Jones, H., Arria, A., Leitich, H., Rohrmeister, K., … & Fischer, G. (2011). Randomized controlled trials in pregnancy: scientific and ethical aspects. Exposure to different opioid medications during pregnancy in an intra-individual comparison. *Addiction (Abingdon, England)*. 106(7), 1355-1362.
115. van den Brink, W., Hendriks, V. M., Blanken, P., Koeter, M. W. J., van Zwieten, B. J., & van Ree, J. M. (2003). Medical prescription of heroin to treatment resistant heroin addicts: two randomised controlled trials. *BMJ (Clinical research ed.)*. 327(7410), 310.
116. Warden, D., Subramaniam, G. A., Carmody, T., Woody, G. E., Minhajuddin, A., Poole, S. A., … & Trivedi, M. H. (2012). Predictors of attrition with buprenorphine/naloxone treatment in opioid dependent youth. *Addictive behaviors*. 37(9), 1046-1053.
117. Weaver, T., Metrebian, N., Hellier, J., Pilling, S., Charles, V., Little, N., … & Strang, J. (2014). Use of contingency management incentives to improve completion of hepatitis B vaccination in people undergoing treatment for heroin dependence: a cluster randomised trial. *Lancet.* 384(9938), 153-163.
118. Wilcox, C. E., Bogenschutz, M. P., Nakazawa, M., & Woody, G. (2013). Concordance between self-report and urine drug screen data in adolescent opioid dependent clinical trial participants. *Addictive behaviors.* 38(10), 2568-2574.
119. Wilcox, C. E., Bogenschutz, M. P., Nakazawa, M., & Woody, G. E. (2012). Compensation effects on clinical trial data collection in opioid-dependent young adults. The *American journal of drug and alcohol abuse.* 38(1), 81-86.
120. Zanis, D. A., Coviello, D., Alterman, A. I., & Appling, S. E. (2001). A community-based trial of vocational problem-solving to increase employment among methadone patients. *Journal of substance abuse treatment.* 21(1), 19-26.

**No Eligible Outcomes (n=25)**

1. Acosta, M. C., Marsch, L. A., Xie, H., Guarino, H., & Aponte-Melendez, Y. (2012). A Web-Based Behavior Therapy Program Influences the Association Between Cognitive Functioning and Retention and Abstinence in Clients Receiving Methadone Maintenance Treatment. *Journal of Dual Diagnosis.* 8(4), 283-293.
2. Aghataher, A., & Mahani, K. N. (2014). The effect of rational emotive behavior group therapy on self-concept and depression of self-introduced drug abusers referred to ofogh addiction treatment center in Zarand (Kerman, Iran). *Biomedical and Pharmacology Journal*. 7(1), 317-323.
3. Avants, S. K., Margolin, A., Kosten, T. R., Rounsaville, B. J., & Schottenfeld, R. S. (1998). When is less treatment better? The role of social anxiety in matching methadone patients to psychosocial treatments. *Journal of consulting and clinical psychology*. 66(6), 924-931.
4. Batki, S. L., Gruber, V. A., Bradley, J. M., Bradley, M., & Delucchi, K. (2002). A controlled trial of methadone treatment combined with directly observed isoniazid for tuberculosis prevention in injection drug users. *Drug and alcohol dependence*. 66(3), 283-293.
5. Booth, R. E., Corsi, K. F., & Mikulich-Gilbertson, S. K. (2004). Factors associated with methadone maintenance treatment retention among street-recruited injection drug users. *Drug and alcohol dependence.* 74(2), 177-185.
6. Calsyn, D. A., Wells, E. A., Saxon, A. J., Jackson, T. R., Wrede, A. F., Stanton, V., & Fleming, C. (1994). Contingency management of urinalysis results and intensity of counseling services have an interactive impact on methadone maintenance treatment outcome. *Journal of addictive diseases*. 13(3), 47-63.
7. Gonzalez, G., Feingold, A., Oliveto, A., Gonsai, K., & Kosten, T. R. (2003). Comorbid major depressive disorder as a prognostic factor in cocaine-abusing buprenorphine-maintained patients treated with desipramine and contingency management. *The American journal of drug and alcohol abuse.* 29(3), 497-514.
8. Haggerty, K. P., Skinner, M., Fleming, C. B., Gainey, R. R., & Catalano, R. F. (2008). Long-term effects of the Focus on Families project on substance use disorders among children of parents in methadone treatment. *Addiction (Abingdon, England).* 103(12), 2008-2016.
9. Kang, S. Y., Magura, S., Blankertz, L., Madison, E., & Spinelli, M. (2006). Predictors of engagement in vocational counseling for methadone treatment patients. *Substance use & misuse*. 41(8), 1125-1138.
10. Kidorf, M., Stitzer, M. L., & Griffiths, R. R. (1995). Evaluating the reinforcement value of clinic-based privileges through a multiple choice procedure. *Drug and alcohol dependence*. 39(3), 167-172.
11. Kim, S. J., Marsch, L. A., Acosta, M. C., Guarino, H., & Aponte-Melendez, Y. (2016). Can persons with a history of multiple addiction treatment episodes benefit from technology delivered behavior therapy? A moderating role of treatment history at baseline. *Addictive behaviors*. 54, 18-23.
12. Kim, S. J., Marsch, L. A., Guarino, H., Acosta, M. C., & Aponte-Melendez, Y. (2015). Predictors of outcome from computer-based treatment for substance use disorders: Results from a randomized clinical trial. *Drug and alcohol dependence*. 157, 174-178.
13. Lones, C. E., Bond, G. R., McGovern, M. P., Carr, K., Leckron-Myers, T., Hartnett, T., & Becker, D. R. (2017). Individual Placement and Support (IPS) for Methadone Maintenance Therapy Patients: A Pilot Randomized Controlled Trial. *Administration and policy in mental health.* 44(3), 359-364.
14. Mancino, M. J., McGaugh, J., Feldman, Z., Poling, J., & Oliveto, A. (2010). Effect of PTSD diagnosis and contingency management procedures on cocaine use in dually cocaine- and opioid-dependent individuals maintained on LAAM: a retrospective analysis. *The American journal on addictions*. 19(2), 169-177.
15. McPherson, S., Brooks, O., Barbosa-Leiker, C., Lederhos, C., Lamp, A., Murphy, S., … & Roll, J. (2016). Examining Longitudinal Stimulant Use and Treatment Attendance as Parallel Outcomes in Two Contingency Management Randomized Clinical Trials. *Journal of substance abuse treatment.* 61, 18-25.
16. Moore, B. A., Fiellin, D. A., Cutter, C. J., Buono, F. D., Barry, D. T., Fiellin, L. E., … & Schottenfeld, R. S. (2016). Cognitive Behavioral Therapy Improves Treatment Outcomes for Prescription Opioid Users in Primary Care Buprenorphine Treatment. *Journal of substance abuse treatment*. 71, 54-57.
17. Newbern, D., Dansereau, D. F., Czuchry, M., & Simpson, D. (2005). Node-link mapping in individual counseling: Treatment impact on clients with ADHD-related behaviors. *Journal of psychoactive drugs.* 37(1), 93-103.
18. Schwartz, R. P., Alexandre, P. K., Kelly, S. M., O'Grady, K. E., Gryczynski, J., & Jaffe, J. H. (2014). Interim versus standard methadone treatment: a benefit-cost analysis. *Journal of substance abuse treatment*. 46(3), 306-314.
19. Simpson, D. D., Joe, G. W., Rowan-Szal, G. A., & Greener, J. M. (1997). Drug abuse treatment process components that improve retention. *Journal of substance abuse treatment*. 14(6), 565-572.
20. Sindelar, J. L., Olmstead, T. A., & Peirce, J. M. (2007). Cost-effectiveness of prize-based contingency management in methadone maintenance treatment programs. *Addiction (Abingdon, England).* 102(9), 1463-1471.
21. Weiss, R. (2017). Predictors of long-term outcome of opioid dependence: Implications for treatment planning. *American Journal on Addictions*. 26(3), 238.
22. Weiss, R. D., Griffin, M. L., Potter, J. S., Dodd, D. R., Dreifuss, J. A., Connery, H. S., & Carroll, K. M. (2014). Who benefits from additional drug counseling among prescription opioid-dependent patients receiving buprenorphine-naloxone and standard medical management? *Drug and alcohol dependence.* 140, 118-122.
23. Woody, G. E., McLellan, A. T., Luborsky, L., & O'Brien, C. P. (1985). Sociopathy and psychotherapy outcome. *Archives of general psychiatry*. 42(11), 1081-1086.
24. Woody, G. E., McLellan, A. T., Luborsky, L., O'Brien, C. P., Blaine, J., Fox, … & Beck, A. T. (1984). Severity of psychiatric symptoms as a predictor of benefits from psychotherapy: the Veterans Administration-Penn study. *The American journal of psychiatry*. 141(10), 1172-1177.
25. Ziaaddini, H., Ebrahim-Nejad, B., & Nakhaee, N. (2013). The Effectiveness of Group Therapy on the Family Functioning of Individuals under Methadone Treatment: A Clinical Trial. *Addiction & health.* 5(1-2), 1-6.

**Duplicate Sample (n=15)**

1. Czuchry, M., Dansereau, D. F., Dees, S. M., & Simpson, D. D. (1995). The use of node-link mapping in drug abuse counseling: the role of attentional factors. *Journal of psychoactive drugs.* 27(2), 161-166.
2. Dansereau, D. F., Joe, G. W., Dees, S. M., & Simpson, D. D. (1996). Ethnicity and the effects of mapping-enhanced drug abuse counseling. *Addictive behaviors*. 21(3), 363-376.
3. Dansereau, D. F., Joe, G. W., & Simpson, D. D. (1995). Attentional difficulties and the effectiveness of a visual representation strategy for counseling drug-addicted clients. *International Journal of the Addictions*, *30*(4), 371-386.
4. Dees, S. M., Dansereau, D. F., & Simpson, D. D. (1997). Mapping-enhanced drug abuse counseling: urinalysis results in the first year of methadone treatment. *Journal of substance abuse treatment.* 14(1), 45-54.
5. Dunlap, G.A., Zarkin, S., Orme, A., Meinhofer, S.M., Kelly, K.E., O’Grady, J., . . . & Schwartz, R.P. (2018). Re-engineering methadone-Cost-effectiveness analysis of a patient-centered approach to methadone treatment. *J Subst Abuse Treat*. 11(94), 81.
6. Ghitza, U. E., Epstein, D. H., Schmittner, J., Vahabzadeh, M., Lin, J. L., & Preston, K. L. (2007). Randomized trial of prize-based reinforcement density for simultaneous abstinence from cocaine and heroin. *Journal of Consulting and Clinical Psychology*, *75*(5), 765.
7. Hutchinson, M. L., Chisolm, M. S., Tuten, M., Leoutsakos, J. M., & Jones, H. E. (2012). The efficacy of escalating and fixed contingency management reinforcement on illicit drug use in opioid-dependent pregnant women. *Addictive Disorders & Their Treatment.* 11(3), 150-153.
8. Joe, G. W., Dansereau, D. F., & Simpson, D. D. (1994). Node-link mapping for counseling cocaine users in methadone treatment. *Journal of substance abuse*. 6(4), 393-406.
9. Knight, D. K., Dansereau, D. F., Joe, G. W., & Simpson, D. D. (1994). The role of node-link mapping in individual and group counseling. *The American journal of drug and alcohol abuse.* 20(4), 517-527.
10. Kraft, M. K., Rothbard, A. B., Hadley, T. R., McLellan, A. T., & Asch, D. A. (1997). Are supplementary services provided during methadone maintenance really cost-effective?. *American Journal of Psychiatry*, *154*(9), 1214-1219.
11. Rogers, R. E., Higgins, S. T., Silverman, K., Thomas, C. S., Badger, G. J., Bigelow, G., & Stitzer, M. (2008). Abstinence-contingent reinforcement and engagement in non-drug-related activities among illicit drug abusers. *Psychology of Addictive Behaviors*, *22*(4), 544.
12. Saxon, A. J., Wells, E. A., Fleming, C., Jackson, T. R., & Calsyn, D. A. (1996). Pre-treatment characteristics, program philosophy and level of ancillary services as predictors of methadone maintenance treatment outcome. *Addiction (Abingdon, England).* 91(8), 1197-1209.
13. Silverman, K., Svikis, D., Robles, E., Stitzer, M. L., & Bigelow, G. E. (2001). A reinforcement-based therapeutic workplace for the treatment of drug abuse: six-month abstinence outcomes. *Experimental and clinical psychopharmacology*. 9(1), 14-23.
14. Woody, G. E., Luborsky, L., McLellan, A. T., O'Brien, C. P., Beck, A. T., Blaine, J., … & Hole, A. (1983). Psychotherapy for opiate addicts. Does it help? A*rchives of general psychiatry.* 40(6), 639-645.
15. Woody, G. E., Luborsky, L., McLellan, A. T., O'Brien, C. P., Beck, A. T., Blaine, J., … & Hole, A. (1983). Psychotherapy for opiate addicts. *NIDA research monograph*. 43, 59-70.
