# Supplementary material for: Evaluating comparative effectiveness of psychosocial interventions adjunctive to opioid agonist therapy for opioid use disorder: A systematic review with network meta-analyses
Source: PLoS One. 2020 Dec 28;15(12):e0244401. doi: 10.1371/journal.pone.0244401 (PMC7769275; doi:10.1371/journal.pone.0244401)
Supplement: S27 Text — (DOCX) [file pone.0244401.s028.docx]

**S27 Text: Treatment Retention NMA – Model Fit Statistics and Findings from Sensitivity Analyses**

A summary of model fit statistics from random effects NMAs is presented in the table below. Both the primary unadjusted RE analysis as well as statistics from meta-regression analyses and sensitivity analyses are presented. For meta-regression analyses, comparisons with the primary unadjusted model considered DIC, reductions in the between study SD and the importance of regression coefficients. The consistency assumption of direct and indirect evidence was also inspected based upon DIC comparisons between consistency and inconsistency models as well as inspection of residual scatterplots.

| **NMA Model** | **# data points** | **Total residual deviance** | **DIC**  **(consistency model)** | **DIC**  **(inconsistency model)** | **SD** | **Significant regression coefficient?** |
| --- | --- | --- | --- | --- | --- | --- |
| Primary (unadjusted) | 100 | 104.2 | 544.1 | 546.2 | 0.2003 | NA |
| **Univariate NMA Meta-Regression Adjusting for:** | | | | | | |
| Control group risk | 100 | 104.7 | 547.5 | 549.4 | 0.2296 | 1 |
| Follow-up duration | 100 | 102.5 | 544.7 | 545.2 | 0.255 | 0 |
| Mean patient age | 100 | 104.7 | 546.1 | 548.5 | 0.21055 | 0 |
| % males | 100 | 104.5 | 544.3 | 547.9 | 0.1757 | 0 |
| **Sensitivity Analyses Excluding:** | | | | | | |
| Studies in potential predatory journals | 96 | 99.97 | 531.1 | 533.2 | 0.1694 | NA |

Model fit was judged adequate across all models based upon comparisons of the posterior total residual deviance and the numbers of unconstrained data points; interpretations were drawn upon the unadjusted model. There was no strong evidence of inconsistency in any of the models, as indicated by inspection of DIC values from consistency models and unrelated means models as well as a scatterplot of posterior deviance contributions from both models (provided below). The comparison-adjusted funnel plot provided no strong evidence of small study effects (next page). Treatment comparisons and SUCRA values were generally robust across analyses (see subsequent figures later in this Appendix).

**
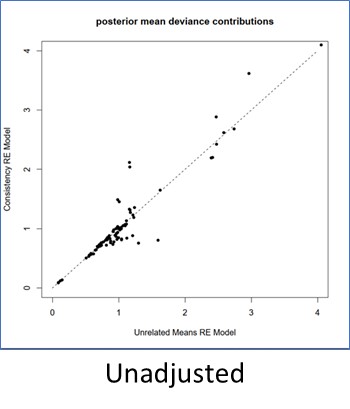
**

**Comparison Adjusted Funnel Plot (Treatment Retention NMA)**

Funnel plot to detect small-study effects—random-effects model—all Counselling or OAT Only comparisons. The red line represents the null hypothesis that the study-specific effect sizes do not differ from the respective comparison-specific pooled effect estimates. The blue regression line represents the change in the comparison-specific effect difference with the SE of the effect size.

**
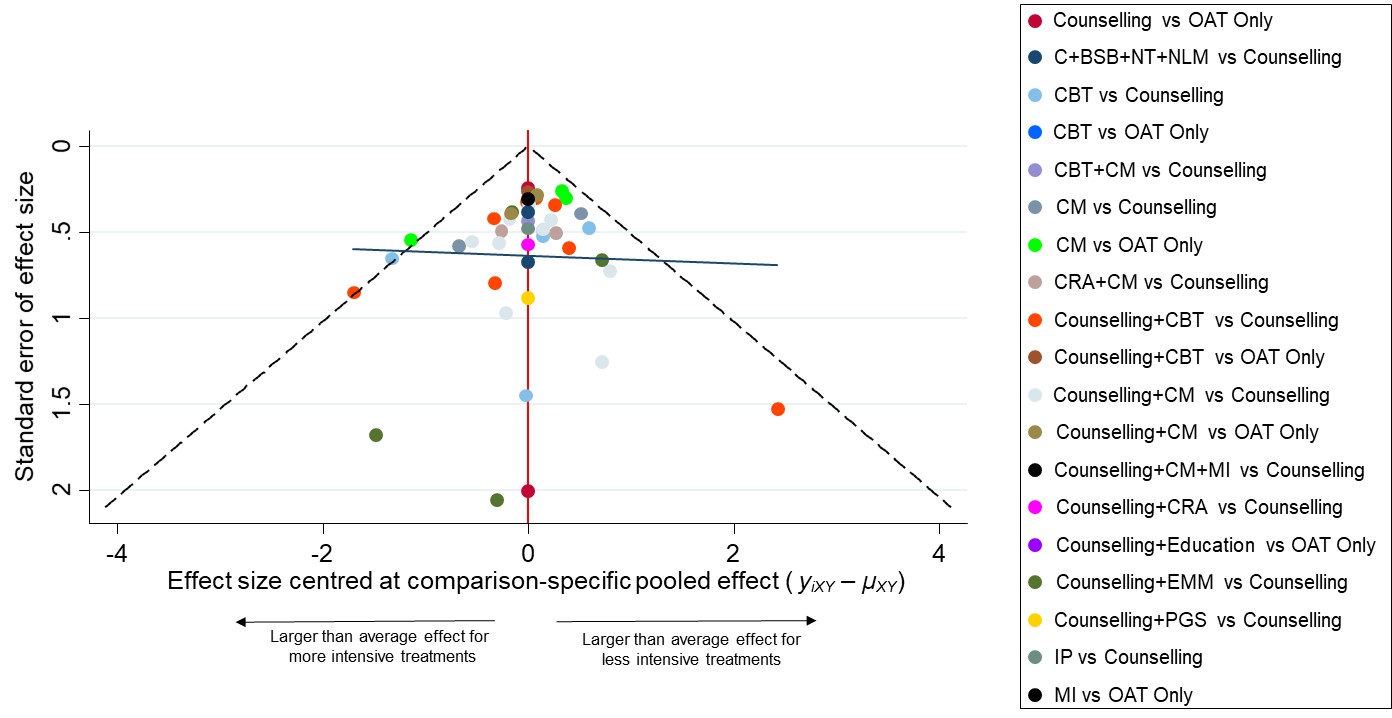
**

A summary of SUCRA values across all RE NMAs performed is presented below; ordering of treatments and magnitudes of SUCRA values per intervention were found to be of a high degree of similarity across analyses. The following page presents forest plots of treatment comparisons versus the reference group OAT only; full league tables have been omitted for brevity, but are available upon request from the authors.


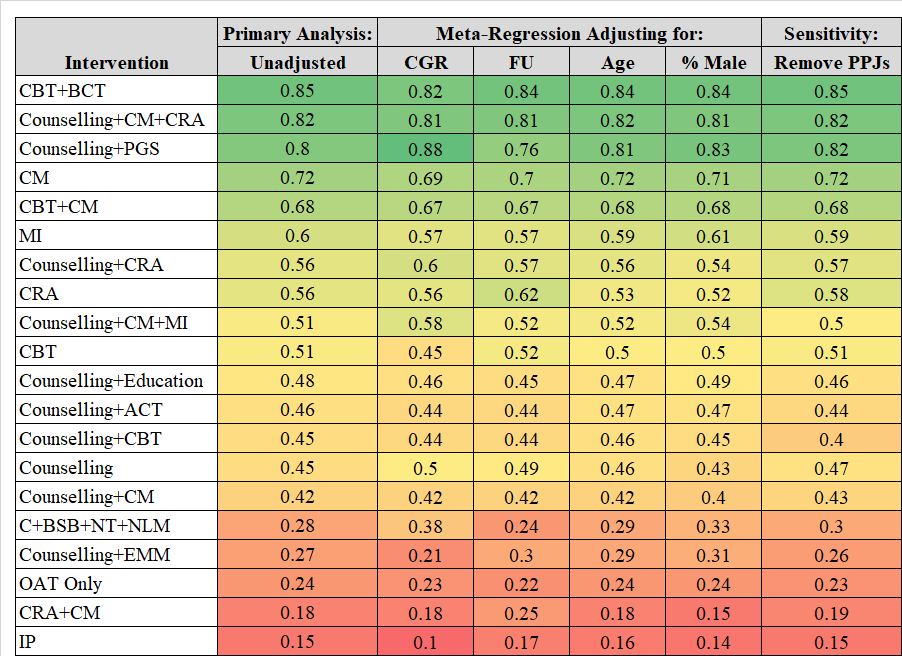


**Summary of Comparisons versus OAT Only Across Models**

| **Unadjusted Analysis (Primary)** | **Control-Group Risk Meta-Regression** |
| --- | --- |
| **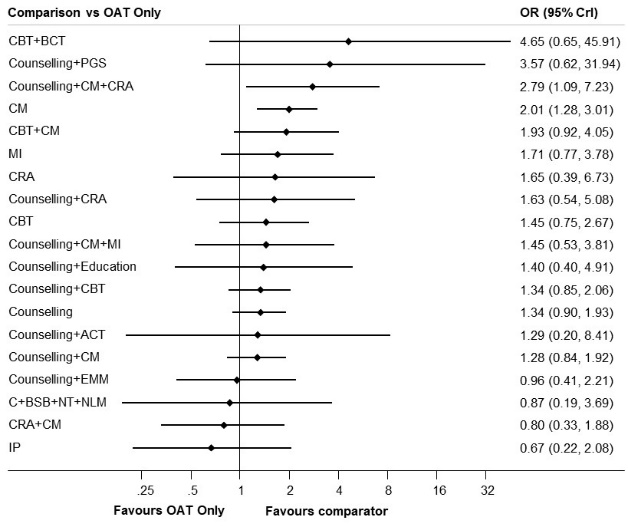** | **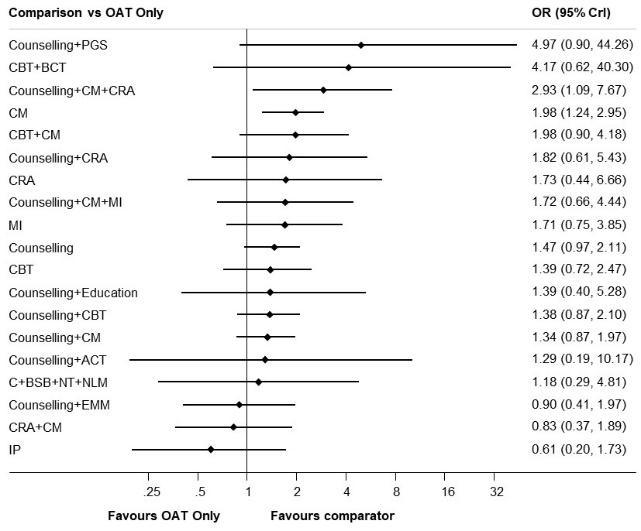** |
| **Meta-Regression for Follow-up Duration** | **Meta-Regression for Mean Participant Age** |
| **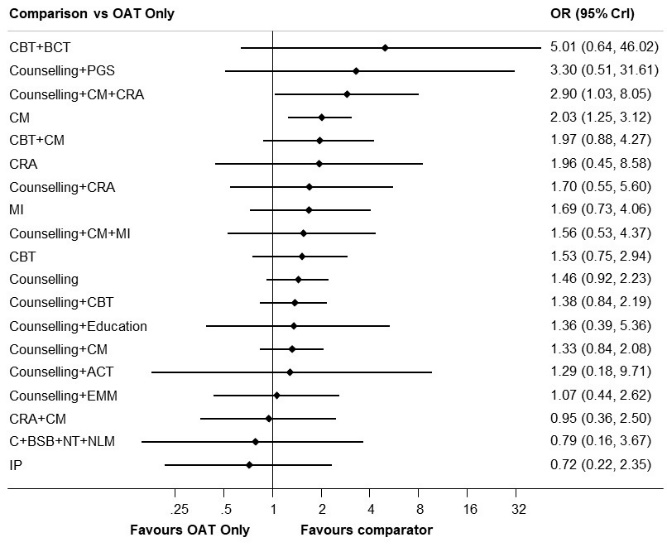** | **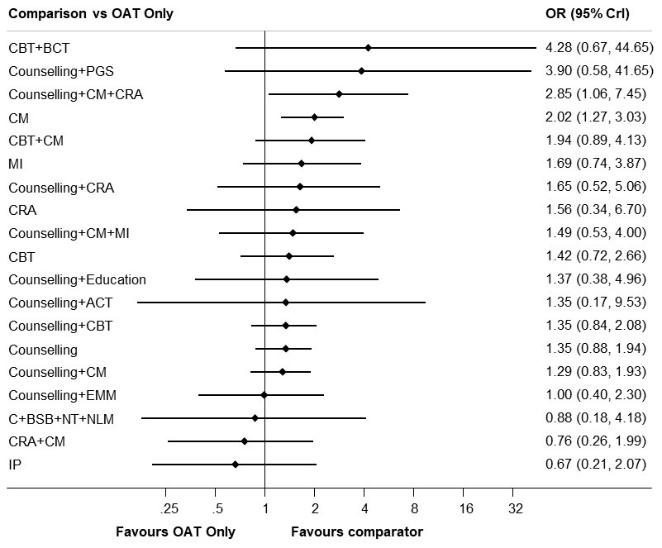** |
| **Meta-Regression for % Male Subjects** | **Sensitivity Analysis:**  **Removing RCTs from Potential Predatory Journals** |
| **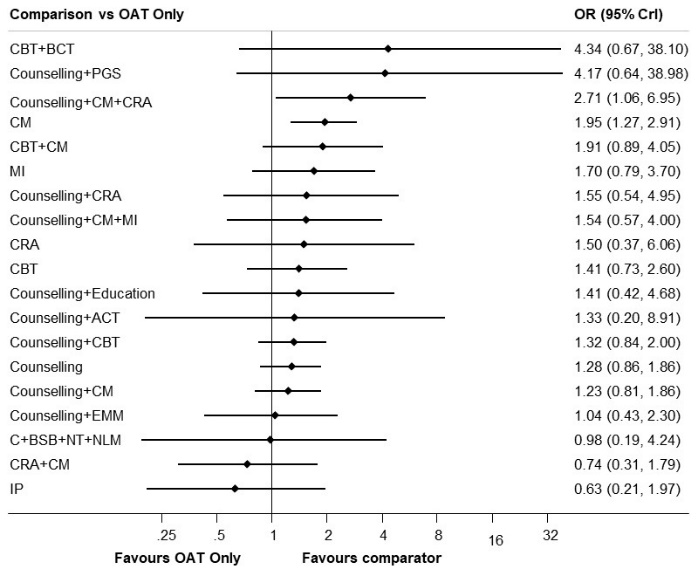** | **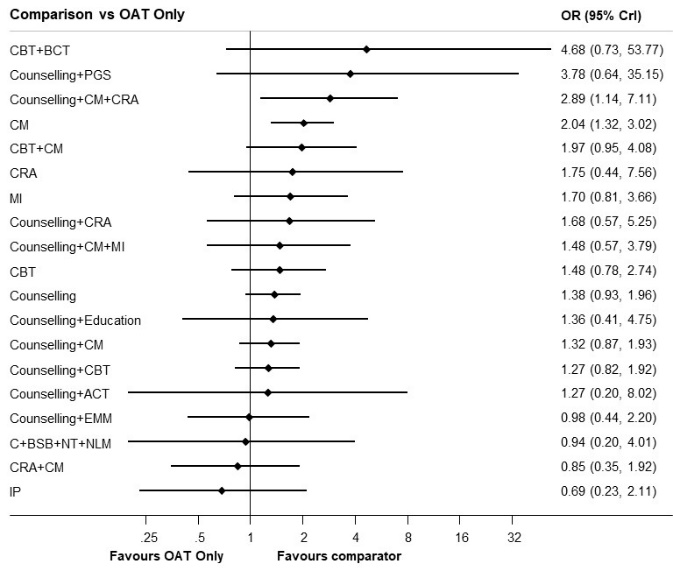** |
