# Supplementary material for: Evaluating comparative effectiveness of psychosocial interventions adjunctive to opioid agonist therapy for opioid use disorder: A systematic review with network meta-analyses
Source: PLoS One. 2020 Dec 28;15(12):e0244401. doi: 10.1371/journal.pone.0244401 (PMC7769275; doi:10.1371/journal.pone.0244401)
Supplement: S28 Text — (DOCX) [file pone.0244401.s029.docx]

**S28 Text: League Table: Unadjusted RE NMA, Treatment Retention**

**
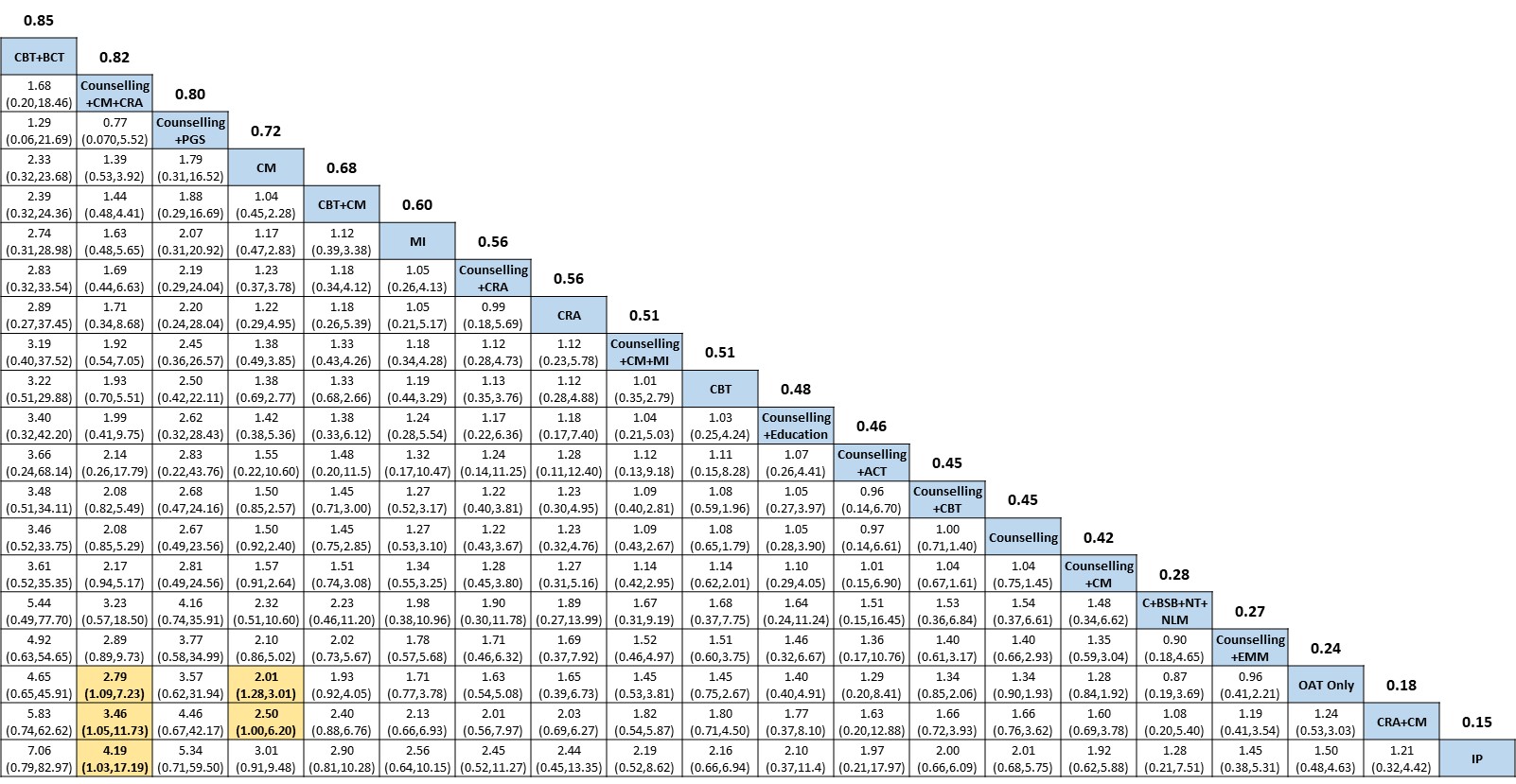
**

A league table summarizing all pairwise comparisons from the primary unadjusted RE NMA for treatment retention is shown. Treatments are ordered from upper left to lower right in order of highest (most preferable) to lowest (least preferable) SUCRA values, which are reported above the diagonal. For each pairwise comparison, the lower/right-most intervention operates as the reference group; for example, for the comparison between CM and OAT only, the estimate of 2.01 (95% CrI 1.28 to 3.01) suggests an important increase in retention with CM. Odds ratios with corresponding 95% credible intervals are shown, and comparisons excluding a possible null difference are shown in bold font with orange coloring.
