# Supplementary material for: Evaluating comparative effectiveness of psychosocial interventions adjunctive to opioid agonist therapy for opioid use disorder: A systematic review with network meta-analyses
Source: PLoS One. 2020 Dec 28;15(12):e0244401. doi: 10.1371/journal.pone.0244401 (PMC7769275; doi:10.1371/journal.pone.0244401)
Supplement: S29 Text — (DOCX) [file pone.0244401.s030.docx]

**S29 Text:**

**Narrative Summary of Findings for Secondary Outcomes with Data from 15 Studies or Fewer**

As noted within the main text of this review, for outcomes with data from 15 or fewer studies, we provided a summary table of key findings within a summary table while providing more detailed summaries in a dedicated appendix. These summaries are provided below; we also point readers to **S8 Text** through **S24 Text** for study-level data related to each outcome.

*Findings, Mental Health Symptoms*

Thirteen studies (18.1%) reported at least one measure of mental health symptoms.^40,42,43,45,68,71,73,76,77,88,92,95,97^ Twelve of these studies^40,42,43,45,68,71,73,76,88,92,95,97^ collected outcomes by self-report measures while one study^77^ included a clinician-rated instrument for depression. Most frequently, the measures collected included symptoms of depression (n = 9),^45,71,73,76,77,88,92,95,97^ general psychological functioning or psychiatric symptoms (n = 3),^40,42,43^ and symptoms of distress (n = 1)^68^ (see **S14 Text** for details). Among the nine studies that measured symptoms of depression, the majority (7/9, 77.8%)^45,71,73,76,77,88,95^ reported no statistically significant differences between control and treatment groups. Two studies^92,97^ conducted prior to 1990, however, reported that individuals receiving psychoanalytic supportive-expressive psychotherapy (with counselling),^92^ CBT alone or CBT with counselling experienced a significantly greater reduction in depression symptoms compared to the counselling group (*p* < 0.05).^97^ Among the one study that measured distress^68^ and the three studies that measured psychological functioning and psychiatric symptoms,^40,42,43^ no significant differences were found when comparing psychosocial interventions to OAT-only,^43^ counselling,^40,42^ or counselling plus 12-step facilitation therapy control groups^112^ (all *p* > 0.05) (see **S14 Text)**.

*Findings, Alcohol Use*

Eleven studies (15.3%)^42,43,58,80-83,88,92,95,104^ reported the use of alcohol using four measures, including the alcohol use composite score of the Addiction Severity Index (ASI; n = 8),^42,43,58,81,82,88,92,104^ Timeline Follow-back (n = 1),^80^ percentage of breath samples negative for alcohol (n = 1),^83^ and the self-reported current drinking patterns (n = 1),^95^ (see **S15 Text** for details). Of the eight studies that reported results from the ASI, five reported study means and standard deviations. The majority of studies that reported on alcohol use found no significant difference between the intervention and control groups (10/11 studies, 90.1%).^42,43,58,80-83,88,95,104^ One study^92^ conducted in 1987 that did describe a significant difference between groups found counselling plus CBT as well as counselling plus psychoanalytic supportive-expressive psychotherapy had significantly less alcohol use as compared to the counselling group that did not receive any additional psychosocial therapy (see **S15 Text**).

*Findings, Adherence to OAT*

Eleven studies (15.3%)^42,43,48-50,53,54,61,72,86,102^ reported adherence to OAT in five different formats, including the number of days OAT was received (n = 4),^48,53,54,102^ the number of days attending a methadone maintenance treatment clinic (n = 3),^42,50,102^ continuing to receive OAT at a specific follow-up timepoint (n = 2),^61,86^ the percentage of doses being taken as prescribed (n = 1),^49^ and attending treatment regularly as defined by the study (n = 1) (see **S16 Text** for details).^43^ Studies that reported adherence to OAT compared OAT-only^43,48,50,61^ or counselling^42,49,53,54,72,86,102^ to CM (alone or with counselling),^42,48,49^ CBT (alone or with counselling),^43,49,72,86,102^ CM plus CBT,^49^ or enhanced medical management (alone or with counselling or CM)^53,54,61^. The majority of studies found no significant difference between the intervention and control groups at follow-up (n = 9/11 studies, 81.8%).^42,43,49,53,54,61,72,86,102^ Two studies found a significant improvement in OAT adherence among individuals receiving counselling^50^ or CM^48^ as compared to OAT-only (see **S16 Text**).

*Findings, Self-reported Opioid Use*

Eleven studies (15.5%) reported opioid use through self-report measures or interview rather than through urinalysis or oral swabs.^42,51-53,59,78,83,84,92,96,109^ Self-reported use was based on the proportion of participants using opioids in the past 30 days,^109^ the number of times or days that opioids were used in the past 30 days,^42,59,84,92^ the frequency of use in the past week,^52,53^ and the frequency of use in the past day (see **S17 Text** for details).^51,78,83^ One of the seven self-reported opioid use outcomes was a structured interview that aimed to identify regular opiate use (i.e., at least once per week).^96^ The self-reported use of opioids between OAT-only^59,96,109^ and counselling control groups^42,51-53,78,83,84,92^ was compared to psychosocial intervention groups (e.g., motivational interviewing, CBT, and CM). Most studies (8/11, 72.7%)^42,51-53,59,83,84,109^ did not find a significant difference between groups regarding the self-reported use of opioids. Of the 11 studies reporting the outcome, seven studies reported raw data while the remaining four studies only reported p-values. Three studies reported statistically significant differences between groups, whereby CM (with counselling),^78^ CBT (alone or with counselling),^92,96^ and skills-based parental training^92^ resulted in significantly less self-reported opioid use as compared to the control group (see **S17 Text**).

*Findings, HIV/HCV Risk Behaviour*

Nine studies (12.5%)^54,57,60,61,67,88,89,91,105^ reported HIV/HCV risk behaviours using three measures, including the drug and sexual score from the HIV Risk-Taking Behaviour scale (n = 2),^67,91^ the AIDS Risk Inventory (n = 3),^54,60,61^ the Risk Assessment Battery (n = 1),^54^ and the AIDS Risk Assessment (n = 1),^57^ where among all measures lower scores represented fewer risk-taking behaviours (see **Appendix 18** for details). Six studies only reported p-value results for this outcome. When comparing OAT-only^57,61^ and counselling^54,60,67,88,89,91,105^ control groups to other psychosocial interventions (e.g., CBT and CM either alone or with counselling), no significant differences were found between groups for six studies (66.7%).^57,60,88,89,91,105^ Two^54,61^ of three studies^54,60,61^ that included enhanced medical management alone or with counselling reported significantly greater reductions in risk-taking behaviours as compared to OAT-only or counselling.^54,61^ One study^67^ that compared counselling to counselling and CM reported significantly greater reductions in the sexual risk-taking behaviours subscale within the counselling and CM group as compared to the counselling group, though non-significant differences between these groups were found for the drug risk-taking behavior subscale. (see **S18 Text**).

*Findings, Withdrawal Symptoms*

Seven studies (9.7%)^40,49,65,73,86,87,96^ reported withdrawal symptoms using six different outcome measures, including the Opioid Withdrawal Symptom Scale (n = 2),^73,76^ the Leeds Dependence Questionnaire (n = 1),^40^ the Clinical Opioid Withdrawal Scale (n = 1),^49^ the Substance-Dependent Severity Scale (n = 1),^65^ Severity of Dependence Scale (n = 1),^96^ and a 17-item self-report measure of withdrawal symptoms (n = 1),^87^ where higher scores indicated experiencing greater cravings (see **S19 Text** for details). Four studies only reported p-value results for this outcome. When comparing OAT-only and counselling control groups to other psychosocial interventions (e.g., CBT and CM either alone or with counselling), no significant differences were found between groups regarding withdrawal symptoms in any of the studies (n = 0/7)^40,49,65,73,86,87,96^ (see**S19 Text**).

*Findings, Adverse Events*

Four studies (5.6%) reported adverse events for each group studied.^49,58,75,102^ Adverse events were reported as the total number of events ^49,58,75^ and the number of “unanticipated negative events”^102^ (see **S20 Text** for details). The four studies compared counselling^49,75,102^ and OAT-only^58^ control groups to counselling and CBT,^49,102^ counselling plus CM,^49,58,75^ or CBT plus CM.^49^ The frequency of adverse events was measured for up to 52 weeks.^49,58^ As few as five events were reported at a follow-up time of 12 weeks,^102^ whereas up to 74 adverse events were reported when participants were followed for a year, although these events were deemed possibly or definitely due to OAT, as opposed to the psychosocial interventions.^49^ Studies did not test statistical significance for these values; however, all studies^49,58,75,102^ concluded that there were no substantive differences for adverse events between study groups (see **S20 Text**).

*Findings, Dropouts from Psychotherapy*

Two studies (2.8%) reported the number of individuals that withdrew from the psychotherapy component of the study but continued to receive OAT (see **S21 Text** for details).^46,95^ One study^43^ found minimal and non-statistically significant differences (*p* > 0.05) in dropout rates when comparing the number of participants that withdrew from the CBT group (9/41 participants, 22.0%) to the counselling group (9/37 participants, 24.3%). A second study^92^ compared dropouts from interpersonal psychotherapy (19/37 participants, 51.4%) to the counselling group (8/35 participants, 22.9%), where more than two times the participants in interpersonal psychotherapy withdrew from this intervention as compared to the counselling intervention (*p <* 0.05) (see **S21 Text**).

*Findings, Craving*

Two studies (2.8%) reported drug craving, where no significant differences were found between groups at the last time point collected (see **S22 Text** for details).^49,83^ In the first study (n = 202),^49^ counselling (mean = 19.3, standard deviation [SD] = 18.2), craving was measured on a visual analogue scale (maximum score possible not reported), and no significant differences (*p* > 0.05) were found between the following groups: counselling plus CBT (mean = 26.6, SD = 25.2), counselling plus CM (mean = 19.7, SD = 21.9), and CBT plus CM (mean = 19.9, SD = 21.2). The second study^83^ (n = 57) administered a questionnaire of cravings on a scale from 0 to 4, where higher scores indicated higher cravings for cocaine and heroin. No statistically significant difference was found (*p* > 0.05) between counselling (mean = 1.34, standard error of the mean [SEM] = 0.18) and counselling plus CM (mean = 1.49, SEM = 0.16). In both studies,^49,83^ the addition of CM and/or CBT did not significantly reduce measures of craving as compared to the control group (see **S22 Text**).

*Findings, Relapse*

Two studies (2.8%) described measuring the number of participants that relapsed using two distinct approaches to defining relapse prevention (see **S23 Text** for details).^41,106^ The first study^41^ included 92 individuals and defined relapse as the number of individuals who did not show up for their maintenance therapy, returned to drug use at least five consecutive days in a row, and had a positive urine test for morphine by week 28 of the study. Of 46 individuals in the CBT group, 17 relapsed (36.4%), as compared to 29 of 46 individuals in the OAT-only group (63.6%). A second study,^106^ that included 60 individuals, defined relapse as the number of individuals that had a positive urine test for morphine by week 8 of the study. Of 30 individuals in the mindfulness-based stress reduction group, 3 relapsed (10.0%), as compared to 10 of 30 (33.3%) individuals that relapsed in the OAT-only group. In both studies,^41,106^ the psychosocial intervention groups had a statistically significant and substantively smaller proportion of individuals relapse as compared to the OAT-only groups (see **S23 Text**).

*Findings, Quality of Life*

One study (1.4%) of 455 individuals reported change in health-related quality of life as measured by the Modular System for Quality of Life (MSQoL) (see **S24 Text** for details).^62^ Mean scores were similar between groups at the 52-week follow-up with a total score of 49.3 (SD = 8.6) for the MI group and 47.0 (SD = 8.6) for the psychoeducation plus counselling group out of a total possible score of 100.0, with higher values indicating higher health-related quality of life (see **S24 Text**).^62^
